# Inflammatory signature in acute-on-chronic liver failure includes increased expression of granulocyte genes *ELANE*, *MPO* and *CD177*

**DOI:** 10.1038/s41598-021-98086-6

**Published:** 2021-09-22

**Authors:** Rohini Saha, Sai Sanwid Pradhan, Shalimar, Prasenjit Das, Priyanka Mishra, Rohan Singh, Venketesh Sivaramakrishnan, Pragyan Acharya

**Affiliations:** 1 Department of Biochemistry, All India Institute of Medical Sciences, Lab 3002, 3rd floor Teaching Block, New Delhi, 110029 India; 2 Disease Biology Lab, Department of Biosciences, Sri Sathya Sai Institute of Higher Learning, Puttaparthi, Andhra Pradesh India; 3 Department of Gastroenterology, All India Institute of Medical Sciences, New Delhi, India; 4 Department of Pathology, All India Institute of Medical Sciences, New Delhi, India

**Keywords:** Biochemistry, Immunology

## Abstract

Acute-on-Chronic Liver Failure (ACLF) is associated with innate immune dysfunction and high short-term mortality. Neutrophils have been identified to influence prognosis in ACLF. Neutrophil biology is under-evaluated in ACLF. Therefore, we investigated neutrophil-specific genes and their association with ACLF outcomes. This is an observational study. Enriched granulocytes, containing neutrophils, isolated from study participants in three groups- ACLF(n = 10), chronic liver disease (CLD, n = 4) and healthy controls (HC, n = 4), were analysed by microarray. Differentially expressed genes were identified and validated by qRT-PCR in an independent cohort of ACLF, CLD and HC (n = 30, 15 and 15 respectively). The association of confirmed overexpressed genes with ACLF 28-day non-survivors was investigated. The protein expression of selected neutrophil genes was confirmed using flow cytometry and IHC. Differential gene expression analysis showed 1140 downregulated and 928 upregulated genes for ACLF versus CLD and 2086 downregulated and 1091 upregulated genes for ACLF versus HC. Significant upregulation of neutrophilic inflammatory signatures were found in ACLF compared to CLD and HC. Neutrophil enriched genes *ELANE, MPO* and *CD177* were highly upregulated in ACLF and their expression was higher in ACLF 28-day non-survivors. Elevated expression of CD177 protein on neutrophil surface in ACLF was confirmed by flow cytometry. IHC analysis in archival post mortem liver biopsies showed the presence of CD177^+^ neutrophils in the liver tissue of ACLF patients. Granulocyte genes *ELANE, MPO* and *CD177* are highly overexpressed in ACLF neutrophils as compared to CLD or HC. Further, this three-gene signature is highly overexpressed in ACLF 28-day non-survivors.

## Introduction

Acute-on-chronic liver failure (ACLF) is known to be driven by systemic inflammation, and is associated with multiple organ dysfunction leading to a very high short-term mortality^1^. Mortality for ACLF within a 28-day period is almost 50%^1^. Neutrophils form a major component of the innate immune system and the neutrophil-to-lymphocyte ratio (NLR) has been shown to be a predictor of mortality in ACLF^2–6^. Recent studies reveal certain characteristics of neutrophil phenotypes in ACLF such as reduced phagocytosis, increased expression of CXCR1/2, differentially expressed cell surface TLR, enhanced NET production^7–10^. However, in-depth molecular understanding of neutrophils and their relevance in ACLF outcomes is lacking. Therefore, the objective of the present study was to define the transcriptomic profiles of polymorphonuclear cells (PMN or granulocytes) derived from ACLF patients, to identify neutrophil specific gene signatures from the transcriptome and, to explore association between expression of these genes with ACLF 28-day mortality. Neutrophils are a major component of granulocyte preparations.We provide a detailed analysis of differentially expressed genes in ACLF derived granulocytes, that contain neutrophil enriched genes, versus those of chronic liver disease (CLD) patients and healthy controls (HC). Using a combination of experimental and analytical approaches, we identify *ELANE*, *MPO* and *CD177* as consistently upregulated genes in the granulocytes of ACLF versus CLD and HC. We also found that this three gene signature was higher in ACLF 28-day non-survivors as compared to survivors. In addition, we have analyzed the pathways that characterize the ACLF granulocytes as compared to CLD and HC.

## Materials and methods

### Study groups and sample collection

ACLF patients admitted to the Department of Gastroenterology, All India Institute of Medical Sciences New Delhi, were diagnosed as per the APASL (Asian Pacific Association for the Study of the Liver) criteria and recruited into this study^1^. The patient recruitment period was between April 2018 and November 2019. The grades of ACLF were defined according to the EASL definition and categorized as grade 1, 2, and 3 depending on the number of organ systems involved. ACLF 1: patients with renal failure (creatinine ≥ 2.0 mg/dl) or a non-renal organ failure plus renal dysfunction (creatinine between 1.5–1.9 mg/dl) and/or HE grade I–II. ACLF 2: Patients with 2 organ failures; ACLF 3: Patients with 3 or more organ failures^11^. Patients with hepatocellular carcinoma or portal vein thrombosis, age group of < 18 years and > 75 years, diabetes (defined on the basis of recent (within 3 months) available fasting blood sugar ≥ 126 mg/dl or random blood sugar ≥ 200 mg/dl; were excluded from the study. During the current admission a random blood sugar ≥ 200 mg/dl (11.1 mmol/l) was used to define diabetes); presence of prior renal, respiratory and/or cardiovascular disease were excluded. Patients with impaired blood glucose levels were excluded because hyperglycaemia even without diabetes has been shown to alter basal neutrophil metabolism and cause activation^12–14^. ACLF patient samples were collected on the day of admission, and follow-up of 28-days was done. After 48 hour of admission, based on the blood culture reports, ACLF patients were stratified as those with sepsis or without sepsis (sterile inflammation). After the 28-day follow up period, ACLF patients were stratified as survivors and non-survivors.

Chronic liver disease (CLD) patients who were within the age group > 18 years and < 75 years, treatment naïve, ambulatory, without any extra-hepatic complications, and no overt symptoms were recruited from the outpatient department. Acute decompensation of CLD but without ACLF (CLD-AD) was defined as complications of cirrhosis such as jaundice, ascites, hepatic encephalopathy and GI bleed, without associated organ failure^15^.

Age and gender-matched healthy volunteers with no recent infection or history of past chronic illness were recruited as study controls. The study has been approved by the All India Institute of Medical Sciences, New Delhi ethics committee [Reference No. IEC/473/9/2016 and, IEC/369/7/2016]. All procedures are as per the declaration of Helsinki. All patients included in the study were > 18 years of age and were recruited in the study after informed consent.

#### Sample processing

**Figure 1:**
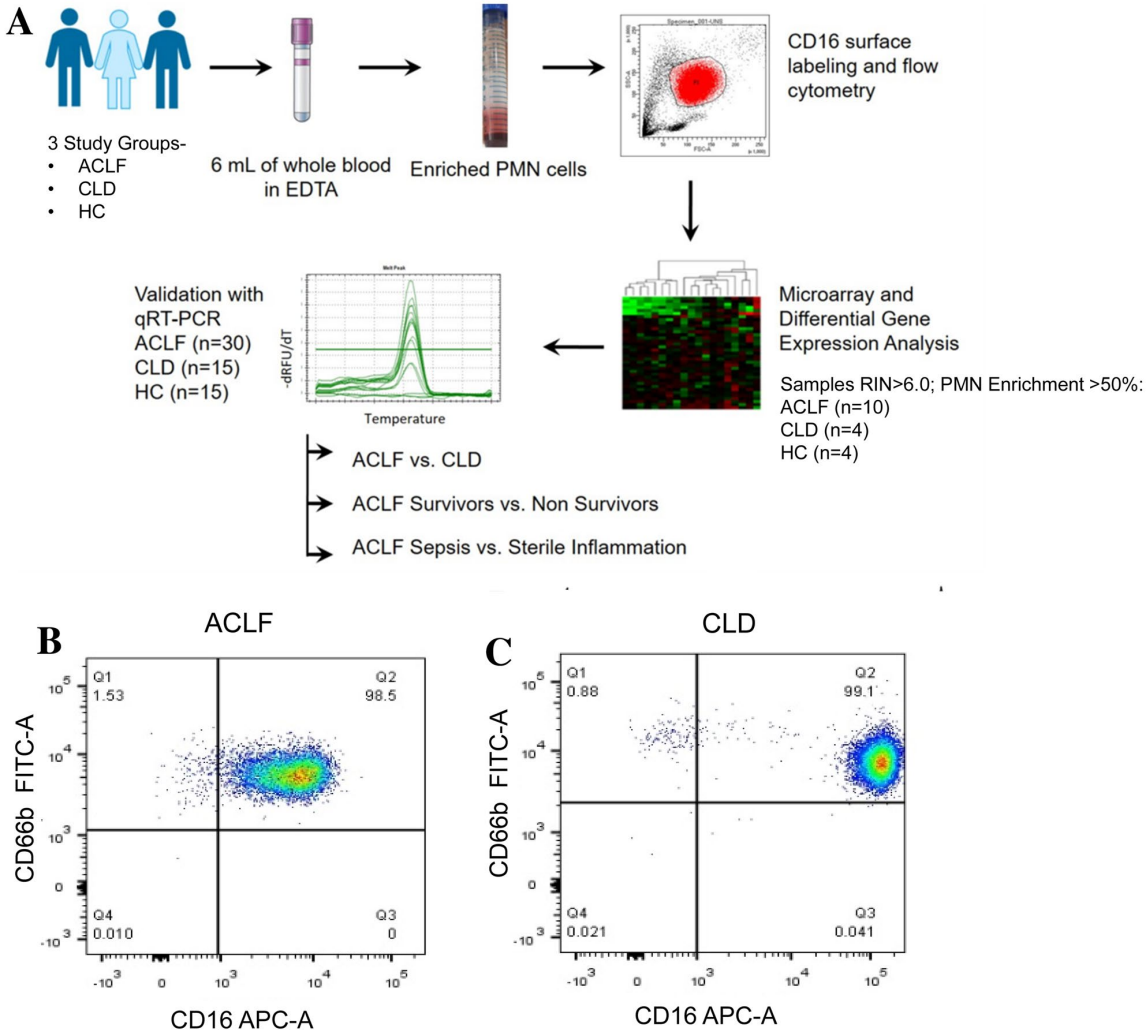
Overall study design and neutrophil enrichment. (**A**) Polymorphonuclear neutrophils (PMN) were enriched from the peripheral whole blood of participants. The isolated PMN were assessed for neutrophil enrichment by CD16 surface staining and analyzed by flow cytometry. RNA were extracted from enriched neutrophils and subjected to microarray analysis followed by quantitative RT-PCR validation of top differentially expressed genes. (**B**, **C**) Flow cytometry based evaluation of the enriched PMN population based on CD16 and CD66b surface staining of representative ACLF (**B**) and CLD (**C**) samples.

PMN were isolated from 6 ml of whole blood in EDTA vial within 2 h of collection, followed by centrifugation at 500×*g* for 10 min. Plasma was aseptically separated and stored at − 80 °C until further use. The remaining blood pellet used for PMN isolation as described below. Enriched PMN were used for all experiments. Overall workflow is described in Fig. 1A.

### PMN isolation and enrichment analysis by flow cytometry

PMN were isolated by modified Boyum's method of double gradient centrifugation^16^. Blood pellet containing the buffy coat was diluted with 2× volume of sterile 1× PBS (*VWR, USA, 97062-730*), at room temperature (RT). Ficoll-Hisep (*Himedia, INDIA, LSM 1077*) was layered over Granulosep (*Himedia, INDIA, LS004*) in a 2:3 ratio to prepare a double gradient in a 15 ml centrifuge tube. Whole blood was carefully layered on top followed by centrifugation at 300×*g* for 30 min at RT without brakes. The following phases were formed in order (top to bottom) after centrifugation: Diluted plasma, PBMC, Hisep, PMN, Granulosep, RBC pellet. Diluted plasma was discarded; PBMC layer was separated and the lower enriched PMN layer was collected. PMN cells were resuspended in sterile 1 × PBS and washed twice by centrifugation at 500×*g* for 10 min. Contaminating RBCs were removed by incubating the washed pellet in 1 × RBC lysis solution for 10 min at 4 °C, followed by two 1 × PBS washes. The final cell pellet was reconstituted in 1.2 ml filtered RPMI-1640 cell culture media (*Himedia, INDIA, AL171A*) supplemented with 2% heat-inactivated fetal bovine serum (FBS) (*Himedia, INDIA, RM10432)*. The enriched PMN cells were diluted 1:20 and counted by Trypan blue exclusion assay to score for live and dead cells (1 µl of reconstituted cells with 10 µl of 0.4% of Trypan blue and 9 µl of sterile 1× PBS). Total cells were counted, and 1 × 10^5^ cells were stained with respective antibodies. To assess PMN enrichment, anti-human CD14 (clone M5E2, *FITC conjugated, Cat No.301803, Biolegend, USA*) and anti-human CD16 (clone 3G8, *APC conjugated, Cat No 302011, Biolegend, USA*) were used and their respective stain index calculated by antibody dilutions. 5 × 10^4^ cells were acquired in BD LSR Fortessa X-20 Flow Cytometer. Unstained controls and single-stain tubes were prepared for each stained sample, and the acquisition was supported by BD FACSDiva software. PMN cells were gated using SSC versus FSC plot and single cells were gated as FSC height versus FSC area (Supplementary Fig. 1A-D). Neutrophils were selected as CD14^−^ (negative) CD16^+^ (positive) population. The percentage enrichment of samples is listed in the Supplementary Table 1.

### PMN RNA isolation and microarray

RNA isolated from 1 × 10^6^ enriched PMN cells was subjected to microarray to identify differentially expressed genes. Briefly, RNA was isolated from 1 × 10^6^ cells using the TRIzol (Invitrogen) and the manufacturer's protocol was followed. The cell suspension was pelletized and 1 ml TRIzol was added to solubilize the cells by vortexing for 30 s. Molecular biology grade chloroform (0.2 ml) was added and mixed until milky-white appearance was formed and incubated at room temperature (RT) for 10 min. Phase separation was done by centrifugation at 12,700×*g* for 15 min. The upper aqueous phase was removed carefully, and RNA was precipitated using 400 µl of ice-cold Isopropanol. The precipitate was washed in 75% ethanol before being air-dried and suspended in 30 µl of RNase free water. RNA quantity and quality were checked using Bioanalyzer.

Total RNA with satisfactory integrity (RNA integrity number or RIN >) and concentration, were labelled and cRNA was synthesized using Agilent Low Input Quick Amp Labelling Kit, One-Colour, without spike-in. This was followed by purification by RNeasy Mini Kit which was quantified with Nanodrop spectrophotometer (Thermofischer Scientific). The RNA yield and specific activity of Cy3 were calculated and samples meeting the manufacturer’s threshold were prepared for hybridization. 240 ng of labelled cRNA was hybridized using the Agilent Gene expression hybridization kit, onto SurePrint G3 Human gene expression v3 (Cat no. G4851C) chips, at 65 °C for 17 h as per the protocol. The chips were washed and scanned using the Agilent surescan microarray scanner.

### Gene expression data analysis

Microarray data have been recorded and prepared according to Minimum Information About a Microarray Experiment (MIAME) guidelines and raw data have been submitted at NCBI Gene Expression Omnibus (GEO) with the accession number GSE156382. Microarray data were extracted using the Agilent Feature extraction software. The individual sample feature extracted files (FEF) were converted, and data normalization was done using Genespring software followed by data analysis (Version 7.0). Briefly, the FEF files containing 56,000 probes individually, was exported to Genespring software, and sample order was chosen and grouping was done (ACLF, CLD, Healthy, ACLF sepsis, ACLF sterile inflammation). Data normalization was done using Percentile and background correction was based on median. The normalized data was subject to sequential filtering: Expression filter of 20–100 window, Filter on flags of Detected/Non detected, Filter on Error on CV < 50%, SD < 0.1, SD < 0.5. Statistical analysis was proceeded with the probe-set of CV < 50%. NetworkAnalyst (https://www.networkanalyst.ca/NetworkAnalyst/home.xhtml) was used for carrying out DEG analysis of the mentioned sample comparatives. NetworkAnalyst uses Limma workflow for analysing data from gene expression experiments^17^. The differentially expressed genes were then selected with threshold criteria of *p *value 0.05 and log FC > 1.0 and < − 0.5. The significant DEG were then tested for their biological pathway implication. All the gene sets from respective comparisons were taken individually for enrichment analysis. The enrichment analysis was done using ClueGO (a Cytoscape plugin) and Enrichr ^18–20^. The enrichment criteria for ClueGO was set for detailed network specificity at 5% genes for clustering and pathway significance of *p* value 0.05. For Enrichr, the significant pathways were selected at adjusted *p* value 0.05. KEGG enrichment analysis was used for overlapping of the DEG in both cases. The pathways output from ClueGO was used for visualization in Cytoscape. A network centrality analysis was done for the created pathway with CytoNCA. A custom style was created wherein genes involved in various pathways were highlighted based on their expression levels and, the size of individual nodes varying to their betweenness values, which helped identify the key elements (genes/pathways) that regulate the network created. Gene set enrichment analysis (GSEA) was additionally performed using the Molecular Signatures Database or MSigDB (http://www.gsea-msigdb.org/gsea/msigdb/index.jsp).

GO cellular component (GO_CC) enrichment analysis was done for DEG of ACLF versus HC and ACLF versus CLD using ClueGO, a Cytoscape plugin at *p* value ≤ 0.05. Additional analysis were carried out between our dataset and published eosinophil, and neutrophil datasets. The DEG profile of transcriptomics datasets were mined from literature for eosinophils (GSE65239) and neutrophils(GSE142254) , a neutrophil dataset of GSE ID-GSE153781 was also taken for analysis. The common DEG in ACLF with respect to HC and CLD and mentioned datasets were illustrated using Venn diagrams (https://www.molbiotools.com/listcompare.php)^21,22^.

The DGE analysis for Neutrophil dataset (GSE153781) was carried out using GREIN, an online interactive web platform used to analyse GEO RNA-seq data (http://www.ilincs.org/apps/grein/).

### Microarray validation and tissue-level expression by Quantitative Real-time PCR

Total PMN RNA was reverse transcribed into cDNA using verso cDNA synthesis kit (AB-1453A from Thermo Fisher Scientific). DNaseI treatment was inherent to this kit and prevented genomic DNA carryover into downstream reactions. PCR and qRT-PCR were performed with 500 ng of cDNA synthesized from total PMN RNA. Prior to qRT-PCR, temperature gradient standardization for all primer sets were done to select optimum annealing temperature. Validation of gene expression log fold change by qRT-PCR was performed for the top 8 differentially expressed genes in ACLF versus CLD using specific primers designed from IDT oligoanalyzer software. The upregulated genes chosen for validation were ELANE, MPO, CD177, OLFM4, and OLAH. The transcript for 18S rRNA was used as a reference gene. The genes ELANE, MPO and CD177 were selected on the basis of their reported specificity in neutrophils, since the neutrophil population is known to be expanded in ACLF, resulting in a high NLR^23–25^.

The primer sequence and qRT-PCR conditions are included as supplementary data as per Minimum Information for Publication of Quantitative Real-Time PCR Experiments (MIQE) guidelines (See Supplementary data_MIQE guidelines file). All graphical representations of log fold change gene expression were done using Graphpad Prism.

### Cell-surface CD177 staining and detection by flow cytometry

Peripheral blood was collected in EDTA vial, and 200 μl of blood was aliquoted for antibody staining. Briefly, 1 ml of RBC lysis buffer was added to 200 μl of whole blood, and mixed properly. A 10× red blood cell lysis buffer was prepared in-house using NH_4_Cl (0.155 M), KHCO_3_ (0.01 M) and EDTA (0.1 mM). The tubes were incubated for 10 min at 4 °C, and centrifuged at 300×*g* for 10 min. The cell pellets were washed twice with 1× PBS and suspended in 300 μl of PBS to obtain a single cell suspension. For cell surface staining, 100 μl of cell suspension was used, and the antibodies CD16 (1:100) (clone 3G8, *APC conjugated, Cat No 302011, Biolegend, USA*), CD66b (1:100) (clone G10F5, FITC conjugated, Cat No 305103, Biolegend, USA) and CD177 (1.5:100) (clone MEM-166, APC/Cyanine 7 conjugated, Cat No 315809, Biolegend, USA) was used. Unstained controls, stained samples, and fluorescence minus one controls were acquired on BD-LSR Fortessa flow cytometry machine. Using the FACS DIVA software, PMN gating was done based on FSC-A v/s SSC-A plot. Enriched and activated neutrophils were gated based on CD16^+^ CD66b^+^ (double positive) in a quadrant plot. CD177^+^ neutrophils were gated within these double positive cells.

### Dual colour immunohistochemistry for CD177 and CD16 in post-mortem liver biopsy

5-micron thick sections of formalin-fixed paraffin-embedded (FFPE) tissues were retrieved from the Department of Pathology, AIIMS New Delhi and taken on coated slides. Deparaffinization was done by dipping the slides in xylene for 5 min (2 changes), acetone for 2–3 min, alcohol for 2–3 min, and then under running/tap water. Antigen retrieval was performed with citrate buffer (pH = 6) in a microwave oven, at 100 degrees Celsius at 900 MW for 30 min. Tissue sections were then allowed to cool down to come to room temperature. The slides were washed three times with Tris buffer (pH 7.5). Endogenous peroxidase blocking was done with 4% Hydrogen peroxide in 96 ml of methanol for 20 min. Anti-CD177 (Invitrogen, pH 9, 1: 50), Rabbit anti-human antibody was incubated overnight at 2–4 degrees Celsius. Next day, three sequences of washings were given with Tris buffer (pH 7.5). Universal polymer-based secondary antibody (SkyTek Laboratories, USA) was incubated at room temperature for 30 min, and the reaction product was developed with 3, 3"-diaminobenzidine chromogen (1: 1). Appropriate positive and negative controls were used. Colour development was monitored under the microscope. Four subsequent sequences of washings with Tris buffer were given at 5 min intervals, followed by the addition of 200 µl of enhancer and incubation for 5 min at. After that the Mouse anti-CD16 antibody (Invitrogen, USA. 1: 50) was incubated at room temperature for 1 h.Three sequences of washings were given with TRIS buffer. Alkaline phosphatase tagged goat anti-mouse IgG H&L secondary antibody (Ab7069) was added in a dilution of 1:10 for 30 min at room temperature. Three sequences of washings were given in Tris buffer. A VECTOR^®^ Blue Alkaline Phosphatase chromogen was used to develop the colour of the reaction (Blue AP), prepared in Tris HCL, pH 8.5, with 5 min incubation at room temperature and monitoring under the microscope. The slides were then washed under running tap water for 3–5 min, counterstained with Neutral red before mounting with a glycerin solution. The dual-colour stained slides were photographed by using a BX43 Olympus microscope. The images taken at 2 × objective power were divided into multiple fields of vision (FOVs) having a diameter of 2 mm each. The procedure for manual cell counting the biopsy core was sequentially divided into multiple non-overlapping FOVs. A systematic eyeballing and counting of the cells were performed and noted. This was done to adjust the variable number of FOV available for each case due to varying biopsy core lengths. In the case of clustering and overlap of the stained cells, only those cells were counted whose nuclei were identified. Additionally, in each case, randomly in 5 FOVs the manual counting was crosschecked by the manual tagging and counting tool of `the Image Proplus 6.1 software. Sepsis was defined on the basis of blood culture reports within 48 h of admission, and the patients who did not have sepsis were classified as having “sterile inflammation”.

### Statistical analysis

All statistical analyses and graphical representations were done using Graphpad Prism software. Normally distributed continuous variables were expressed as Mean ± SD, and continuous variables with skewed distribution were expressed as Median (Interquartile Range). For two group comparisons, Unpaired T-test and Mann–Whitney Test were applied for categorical variables and non-normally distributed variables, respectively.

## Results

### Study participants, workflow and neutrophil enrichment

The study groups included in the microarray-based gene expression analysis were ACLF (n = 10), CLD (n =4 ); as well as Healthy controls (n = 4) (Table 1, Fig. 1A). ACLF is a dysfunction that occurs due to an acute injury over an underlying chronic liver disease (CLD). All the ACLF patients recruited into this study had organ failure scores of above 2 (CLIF-C- OF 2 and 3) indicating the presence of multiple organ dysfunction. Chronic etiologies in ACLF patients included alcohol (n = 4), autoimmune hepatitis (AIH; n = 3), cryptogenic causes (n = 3). Acute etiologies included alcohol (n = 2), AIH (n = 2), HEV + alcohol (n = 1), cryptogenic causes (n = 5). Etiologies for CLD(Compensated -diseased controls) patients included alcohol (n = 2) and viral hepatitis (n =23) (Table 1).

**Table 1:** Baseline Characteristics and comparison of ACLF, CLD and Healthy controls.

Variables	ACLF (n = 10)	CLD (n =4 )	HC (n = 4)
Age/ years (Mean ± S.D.)	44 (± 16)	33(± *9*)	44 (± 9)
Gender (male)	10	4	2
Etiology chronic	Alcohol (4), AIH (3), Others (2), Cryptogenic (1), Viral (0)	Viral (2), Alcoholic (2)	–
Etiology acute	Alcohol (2), Sepsis (3), AIH (1), AIH + sepsis (1), HEV + alcohol (1), Unknown causes (2)	–	–

ACLF=Acute-on-chronic liver failure, CLD=Chronic liver disease, HC=Healthy Control, AIH= Autoimmune hepatitis, HEV= Hepatitis E virus

**Figure 2:**
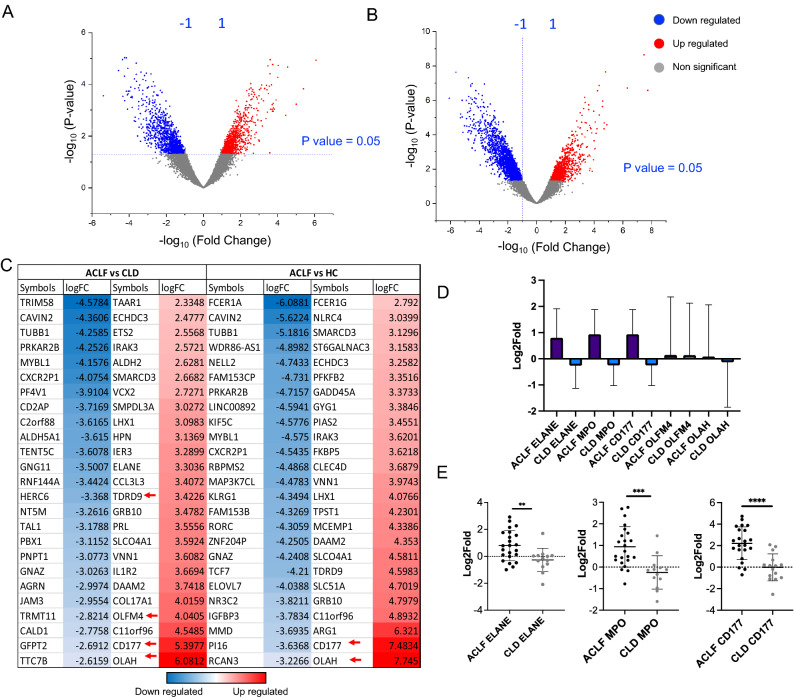
Total gene expression analysis and differentially expressed genes. (**A**) Volcano plots representing -log (p-value) vs log fold change of ACLF versus HC, ACLF versus CLD. (**B**) Two-way analysis for differential gene expression. Top 50 upregulated and downregulated genes represented as heatmap with respective log fold changes (**B**) ACLF versus HC and (**C**) ACLF versus CLD. (**D**) Validation of microarray gene expression trends by qRT-PCR. Y-axis represents Mean log fold change of differentially expressed genes in ACLF versus. CLD and X-axis represents gene names. Validation with qRT-PCR was performed for the following genes: *ELANE, MPO, CD177, OLFM4, OLAH*. Sample sizes were ACLF (n = 10), CLD (n = 4), HC (n = 4) for microarray and ACLF (n = 30), CLD (n = 15) and HC (n = 15) for qRT-PCR. (**E**) Pairwise analysis shows significant elevation of ELANE (*p* value = 0.0064), MPO (*p* value = 0.0003) and CD177 (*p* value < 0.0001) in ACLF versus CLD.

Neutrophil enrichment was determined by surface labelling of cells for the markers CD14 and CD16; followed by flow cytometric analysis (Supplementary Fig. 1A-D; Fig. 1B,C, Supplementary Table 1). Neutrophil enrichment was calculated as the percentage of CD16^+^ cells in the PMN/ granulocyte gate over total acquired cells. Purity of the neutrophil population was defined as the percentage of CD16^+^ cells over total PMN. The median values for PMN enrichment were: ACLF 80.60% (45.1–93); CLD 52.3% (41.2–60); HC 75.7% (68.5–82.7). The median values for CD16^+^ neutrophils as a percentage of total PMN were—ACLF 97.9% (67–99); CLD 84% (76–95) ; HC 96% (90–98-98.79) (Supplementary Table 1) . RNA isolated from enriched PMN/granulocytes were subjected to microarray analysis. Differential gene expression (DGE) and pathways analysis were performed only with the samples which showed > 50% enrichment and RIN score > 6.0 (Fig. 2A-E). The only exceptions were- 1 sample of ACLF (45.2%) and 2 of CLD (45.2% and 41.1%). These were included due to their high RIN value (> 7.0) suggesting good RNA quality. Since the aim of the experiment was to describe an overall gene expression profile of PMN cells and additional validation of selected genes using qRT-PCR was incorporated, these three samples were included so that robust transcriptome profiles could be obtained from which gene expression patterns could be further extracted and validated.

The qRT-PCR validation set included ACLF (n = 30), CLD (n = 15) and Healthy controls HC (n = 15) samples. These samples were age and gender matched to minimize bias, and the baseline clinical parameters were compared between ACLF and CLD (compensated) (Table 2). As expected, baseline parameters were significantly different from ACLF, due to the worsening of liver function and presence of organ failure in ACLF. We next estimated the Neutrophil to lymphocyte ratio (NLR) from the complete blood count (CBC) reports of these patients. The NLR median value was 8.2 in ACLF versus 1.9 in CLD patients (Supplementary Fig. 1E).

**Table 2:** Baseline characteristics of ACLF, CLD and Healthy controls used in qRT-PCR validation.

Variables	ACLF (n = 30)	CLD (n = 15)	Healthy control (n = 15)	p-value
Age (years)	46 ± 13	39 ± 10	35 ± 9	0.3706
Gender (No. of males)	21	10	8	0.479
Hemoglobin (gm/dL)	8.40 (6.90–10.03)	11.1 (9.9–13.8)	n.a	< 0.0001
TLC (count/µl)	12,831 ± 7455	4480 ± 3693	n.a	0.0043
Platelet × 1000/µL	76.50 (37.25–125.0)	n.a	n.a	n.a
INR	2.10 (1.80–3.62)	1.2 (1.0–2.41)	n.a	0.008
Creatinine (mg)	1.4 (1.0–2.22)	0.90 (0.7–1.0)	n.a	0.001
Sodium (mEq/L)	140 ± 9.6	n.a	n.a	n.a
Bilirubin (mg)	12.10 (4.27–24.28)	1.2 (0.4–2.0)	n.a	< 0.0001
AST(IU/L)	88.0 (64.25–146.8)	44.0 (28.0–67.25)	n.a	0.001
ALT (IU/L)	39.0 (27.75–59)	36.0 (22.0–48.0)	n.a	0.0.32
SAP (IU/L)	233.5 (189.5–293.0)	305.0 (240.0–529.5)	n.a	0.04
Albumin (gm)	2.78 ± 0.59	n.a	n.a	n.a
Neutrophil to lymphocyte ratio (NLR)	8.2(4.0–14.1)	1.90 (1.3–8.90)	n.a	< 0.0001

Acute on chronic liver failure (ACLF) n = 30, Chronic liver disease (CLD) n = 15, Healthy controls (HC) n = 15. Parameters which pass the Pearson Normality test are represented by Mean ± SD, and the rest are represented as Median (25th Quartile -75th Quartile). For samples with normal distribution, students T test was performed, and for samples with skewed distribution. Mann whitney test was performed.

Note: NLR has been calculated for 28 out of 30 ACLF patients, and 13 out of 15 CLD patients.

HB = Hemoglobin, TLC = Total leukocyte count, PT = Prothrombin time, INR = International normalized ratio, AST = Aspartate aminotransferase, ALT = Alanine aminotransferase, SAP = Serum Alkaline phosphatase.

### Differential gene expression patterns in ACLF, CLD and HC

Differential gene expression (DGE) analysis revealed 2068 (1140 downregulated and 928 upregulated) genes for ACLF versus CLD and 3177 (2086 downregulated and 1091 upregulated) genes for ACLF versus HC (Fig. 2A-C). CD177 was one of the highest upregulated genes in ACLF versus HC as well as ACLF versus CLD (Fig. 2C). Other genes found to be among the top 50 upregulated genes in ACLF were- ELANE, OLFM4 and OLAH (Fig. 2C; Supplementary Tables 2 and 3). MPO gene, a classical neutrophil activation marker was found to be upregulated in ACLF versus CLD with a Log2FC of 2.4 (Supplementary Table 2). Based on the neutrophil gene expression patterns reported in literature, and log-fold values in our microarray data set, 5 differentially expressed genes from ACLF versus CLD, were selected for validation using qRT-PCR which included ELANE, MPO, CD177, OLFM4 and OLAH (Fig. 2D). Overall trends for ACLF versus CLD were found to be conserved in the qRT-PCR analysis (Fig. 2D). ELANE, MPO and CD177 are three genes which have been linked to neutrophil pathogenicity in inflammatory disorders and were found to be significantly upregulated in ACLF PMN compared to CLD or HC (Fig. 2E).

### Association of PMN gene expression with short-term mortality in ACLF

**Figure 3:**
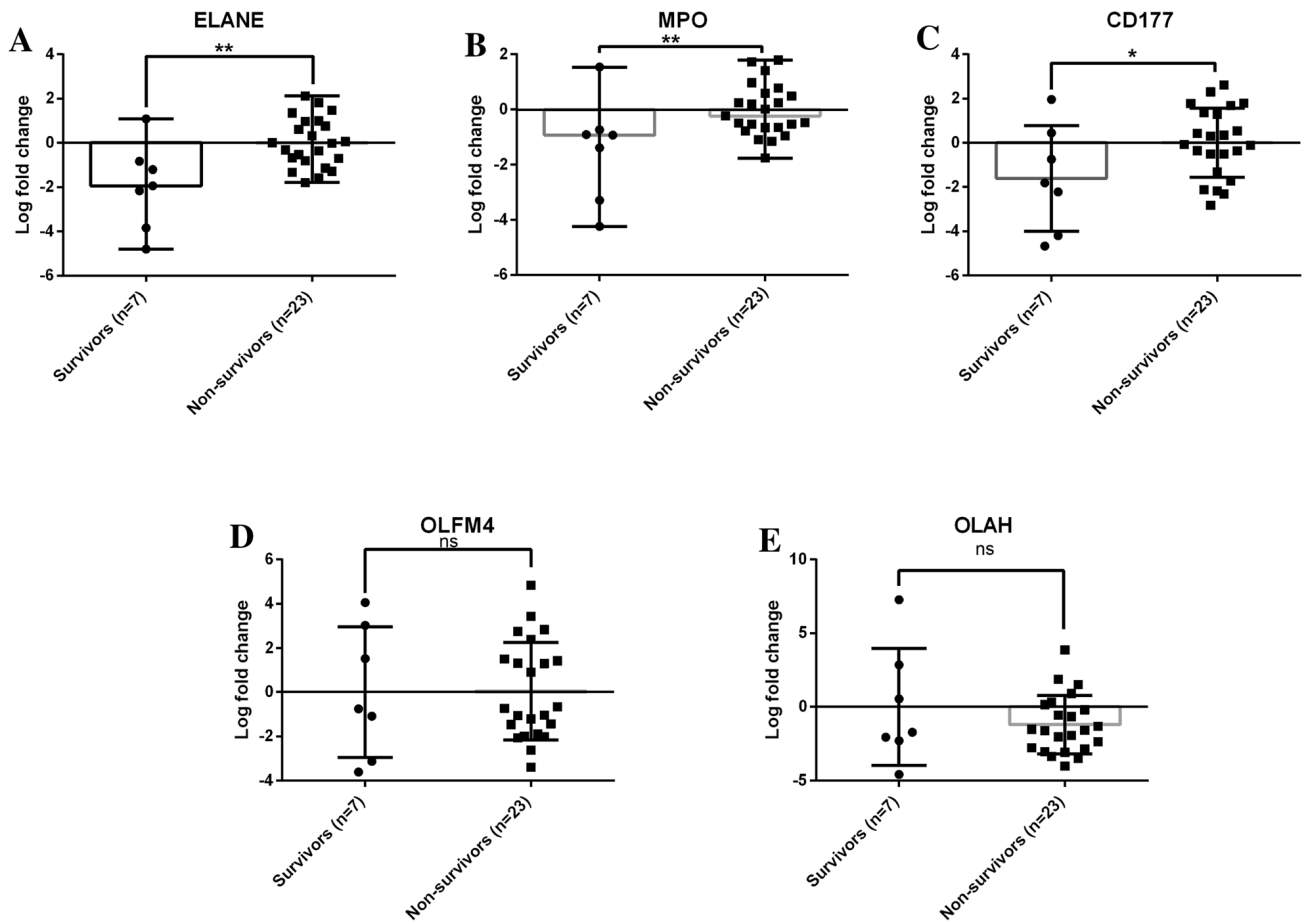
Differential gene expression of ELANE, MPO and CD177 in ACLF 28-day non-survivors versus survivors. Gene expression assessment of selected differentially expressed genes by qRT-PCR in ACLF 28-day non-survivors versus survivors. For the calculation of relative expression and log fold change, 18 s rRNA was taken as reference gene and survivors were taken as calibrator group. ELANE, MPO and CD177 were found to be elevated in 28-day non-survivors. Unpaired t-test was used for statistical testing; (**A**) ELANE (*p* value = 0.008), (**B**) MPO (*p* value = 0.007), (**C**) CD177 (*p* value = 0.04), (**D**) OLFM4 (*p* value = 0. 96), (**E**) OLAH (*p* value = 0.28). 0.004, 0.02, 0.04.

Since the high degree of mortality observed in ACLF is believed to be driven by highly inflammatory innate immune responses, gene expression levels obtained from qRT-PCR experiments for the genes ELANE, MPO, CD177, OLFM4, OLAH (upregulated in ACLF versus CLD or ACLF versus HC) were stratified based on 28-day mortality in ACLF (Fig. 3A-E). Mean gene expression values of the granulocyte genes ELANE, MPO and CD177 were significantly elevated in 28-day non-survivors as compared to survivors (Fig. 3A-C; *p* values 0.008, 0.007, 0.04 respectively for ELANE, MPO and CD177). OLFM4 and OLAH were comparable in both the groups amongst survivors and non-survivors with ACLF (Fig. 3D,E). Stratification based on sex showed comparable values of ELANE, MPO, CD177, OLFM4 and, OLAH in both males and females (Supplementary Fig. 3) in all the three groups (ACLF, CLD and HC).

Since sepsis is a major etiology as well as complication in ACLF, gene expression values were stratified into sepsis versus sterile inflammation (Supplementary Fig. 2). ELANE, MPO, CD177, OLFM4 and OLAH gene expression were found to be comparable in both the groups sepsis and sterile inflammation (Supplementary Fig. 2) indicating that neutrophil response to both bacterial infection and tissue injury involved upregulation of these genes.

A literature search for the functions of the above mentioned genes revealed that MPO is associated with inflammatory diseases such as cardiovascular disease and sepsis, whereas the role of the other neutrophil-enriched genes such as ELANE, CD177 and OLFM4 are relatively poorly described. ELANE, MPO and CD177 were among the highest expressed genes in transcriptome meta-analyses of sepsis whole blood transcriptomes (Supplementary Table 11). MPO and CD177 but not ELANE, have been found to be associated with increased ROS production and enhanced phagocytosis in neutrophils. The precise role of CD177, OLFM4 and OLAH in granulocyte functions, is unknown.

### CD177 surface level expression is elevated in ACLF

**Figure 4:**
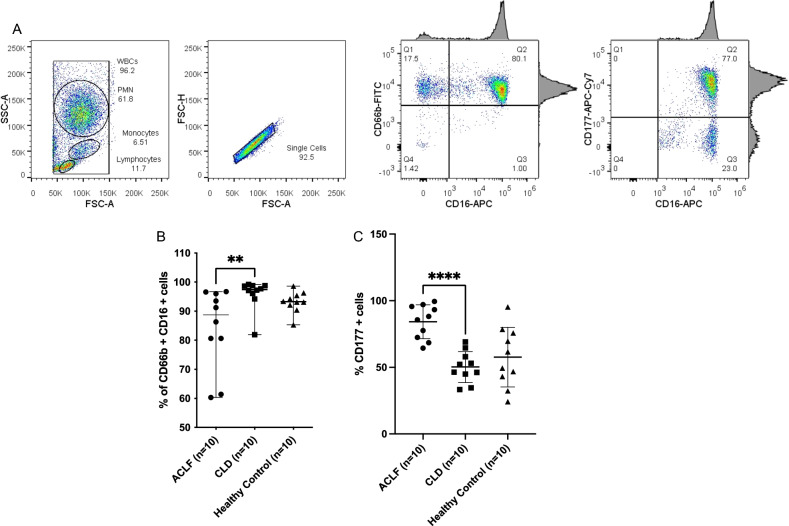
CD177 cell surface level expression is higher in ACLF versus CLD. (**A**) Gating strategy for assessment of CD17^+^ cells by flow cytometry. Neutrophil enrichment was determined by CD66b^+^ and CD16^+^positivity. The first plot represents an FSC versus SSC scatter plot, used for gating PMN cells. Singlets gating as determined by FSC-height v/s FSC-area plot. This is followed by a quadrant plot for CD66b-FITC versus CD16-APC, and double positive neutrophils are gated on Q2. The last quadrant plot represents CD177-APC-Cy7 versus CD16-APC. CD177 positive cells are gated. (**B**) CD66b^+^CD16^+^ positive neutrophil percentage across groups. The mean percentage of enrichment is significantly different across groups as determined by Kruskal Wallis test (**C**) CD177 positive cell percentage across study groups. The mean CD177 population was found to be significantly different across the three groups, as calculated by one-way ANOVA. Pairwise analysis for CD177 + cell percentages were performed for ACLF versus CLD. *p value ≤ 0.05; ** p value ≤ 0.005, ***p value ≤ 0.001.

CD177 is a neutrophil specific cell surface molecule which defines heterogeneous neutrophil populations. Since the CD177 gene was found to be highly overexpressed in ACLF neutrophils, we investigated the cell-surface protein level expression of CD177 in circulating neutrophils from ACLF patients. For this, the total percentage of CD16^+^ and CD66b^+^ neutrophils were estimated from the whole blood of ACLF, CLD and Healthy (n = 10 per group) samples. These enriched double positive (CD16^+^CD66b^+^) cells define mature and activated neutrophils in circulating blood. ACLF, CLD and HC groups included in the flow cytometric analysis of CD177, had median enrichment values of 81.6% (62.7–94.3), 97.4% (95.3–98.5) and 90.3% (84.1–94.1) % respectively. Figure 4A represents the gating strategy used for the assessment of CD16, CD66b and CD177. First, PMN were gated based on their characteristic position in the FSC-A/ SSC-A plot, followed by singlet gating. Singlets were assessed for two markers- CD16 (human FCγRIII) and CD66b. While CD16 is a marker present on most neutrophils, CD66b is an activation marker specific to mature neutrophils. A majority of the neutrophils in the CLD group were found to be CD66b + (almost 100%); ACLF showed a wider range and HC in our study population showed about 92% mature neutrophils (Fig. 4B). CD177^+^ cells were gated within the CD16^+^CD66b^+^ positive neutrophil population (lineage markers for granulocytes and neutrophils) for all samples. Percentage of CD177^+^ neutrophils as a proportion of total CD16^+^CD66b^+^ neutrophils were compared between the study groups. Percentage of CD177^+^ neutrophils in total CD16^+^CD66b^+^ neutrophils were found to be significantly higher in ACLF (~ 80%) as compared to CLD (~ 50%) (*p* value < 0.0001) and Healthy controls (~ 60%) (*p* value 0.005) (Fig. 4B,C). The values of CD16^+^CD66b^+^ enrichment and CD177 positivity for individual samples are provided in Supplementary Table 4.

### Presence of CD177 + neutrophils in ACLF and CLD-AD post-mortem liver biopsies

**Figure 5:**
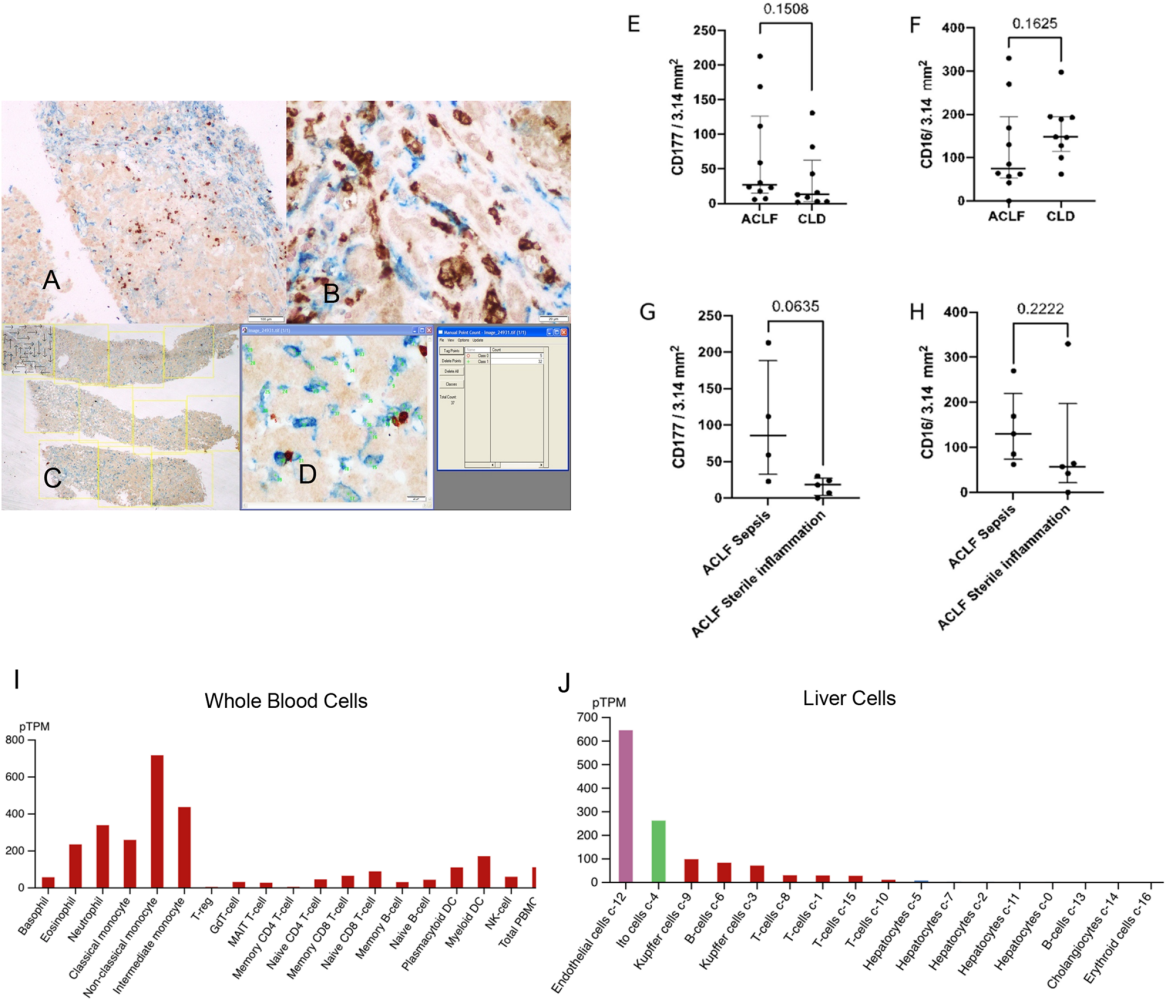
Immunohistochemistry of post-mortem biopsies obtained from ACLF and CLD-AD patients. (**A**, **B**) The dual-colour stained slides were photographed by using a BX43 Olympus microscope. The CD177 stained neutrophils were represented by brown stain (black arrows) and the CD16 stained neutrophils, monocytes, macrophages, and Kupffer cells were represented by blue stain (blue arrows) (A × 40, B × 400). (**C**) Method for observation of dual-colour stained slide under 2 × objective of the microscope and selection of non-overlapping FOVs followed by counting at × 40; (**D**) Method of using the manual tagging and counting tool of the Image Proplus 6.1 software [D × 40] (**E**) CD177^+^ Neutrophil count/ 3.14 mm^2^ in ACLF and CLD-AD patients; (**F**) CD16^+^ leukocytes in ACLF and CLD-AD patients; (**G**) CD177^+^ Neutrophil count/ 3.14 mm^2^ in ACLF patients with or without sepsis; (**H**) CD16^+^ leukocytes in ACLF and CLD-AD patients. Sample size for each group- ACLF (n = 10); CLD-AD (n = 9); ACLF sepsis (n = 4); ACLF sterile inflammation (n = 5). For 1 ACLF sample, categorization as sepsis/ sterile inflammation was not available). Non-parametric Mann Whitney Test was performed to determine *p* values (indicated on the top of the graphs). Graphs are plotted as Median with IQR. (**I**) Cell-type specific expression of CD177 binding partner PECAM1 in whole blood cells. (**J**) Cell-type specific expression of PECAM1 in liver cells. (**I**, **J**) Data have been taken from Human Protein Atlas.

Formalin fixed paraffin embedded (FFPE) liver biopsies from deceased ACLF and CLD-AD (CLD with acute decompensation but without ACLF) were retrospectively retrieved and subjected to dual colour IHC for CD16 and CD177 in order to investigate the tissue localization of the CD177^+^ neutrophil sub-population (Fig. 5A-D). A limitation of this experiment was that “normal” or “CLD” control liver biopsies are ethically non-permissible at our centre in the absence of clinical indication for a biopsy in live patients, and therefore, cannot be included as controls. The CD177 stained neutrophils were represented by brown stain (black arrows) and the CD16 stained neutrophils, monocytes, macrophages, and Kupffer cells were represented by blue stain (blue arrows) (Fig. 5A,B). The manual counts, post validation was averaged for each cell type in a case and were expressed per 3.14 mm^2^, that is the area of each FOV of 10 × objective in this microscope (Supplementary Table 5). These data show that CD177^+^ neutrophils are detectable in the liver tissue of the deceased patients in both the ACLF and CLD-AD groups and CD16^+^ leukocytes were present at comparable levels in both groups (Fig. 5E,F; *p* values 0.1508 and 0.1625 respectively). Stratification of ACLF samples based on the presence or absence of sepsis demonstrated higher numbers of CD177^+^ neutrophils in the ACLF sepsis biopsies although CD16^+^ neutrophils were comparable (Fig. 5G,H, *p* value 0.0635 and *p* value 0.2222 respectively).

In order to understand target cells with which CD177^+^ neutrophils may potentialy interact, cell specific expression data was extracted from human protein atlas for the well known CD177 binding partner PECAM1 (https://www.proteinatlas.org/ENSG00000261371-PECAM1/celltype)^26^. Among blood cells , classical and non-classical monocytes, neutrophils, eosinophils had high expression of PECAM1 and CD4 naïve T cells, naïve and memory CD8 T cells, B cells and dendritic cells had low expression of PECAM 1 (Fig. 5I). Within the liver, endothelial cells, hepatic stellate cells (Ito cells), Kupffer cells, B cells and T cells were found to express PECAM1 mRNA (Fig. 5J).

### Pathways analysis reveals several inflammatory modules within ACLF

Pathways analysis using the DEG from ACLF versus CLD and ACLF versus HC were performed using the algorithms ClueGO and Enrichr. ClueGO analysis showed 15 significant pathways for ACLF versus CLD and 12 significant pathways for ACLF versus HC (Supplementary Tables 6 and 7 respectively). Enrichr analysis showed 22 significant pathways for ACLF versus CLD and 18 significant pathways for ACLF versus HC (Supplementary Tables 8 and 9 respectively). Significant enriched pathways included upregulation of inflammatory signatures similar to those observed in well studied inflammatory diseases such as SLE, Asthma, Inflammatory Bowel Disease (IBD) and Rheumatoid Arthritis (RA) (Supplementary Fig. 4). The Gene Ontology Cellular Components (GO_CC) analysis revealed a significant upregulation of secretory, azurophilic and tertiary granule components in ACLF versus HC as well as ACLF versus CLD PMN (Supplementary Fig. 4). GSEA analysis using MSigDB revealed the upregulation of the neutrophil degranulation pathway in ACLF versus CLD, supporting the observation that neutrophil granule genes ELANE and MPO and related cell surface protein CD177 were highly upregulated in ACLF PMN (Supplementary Table 10).

**Figure 6:**
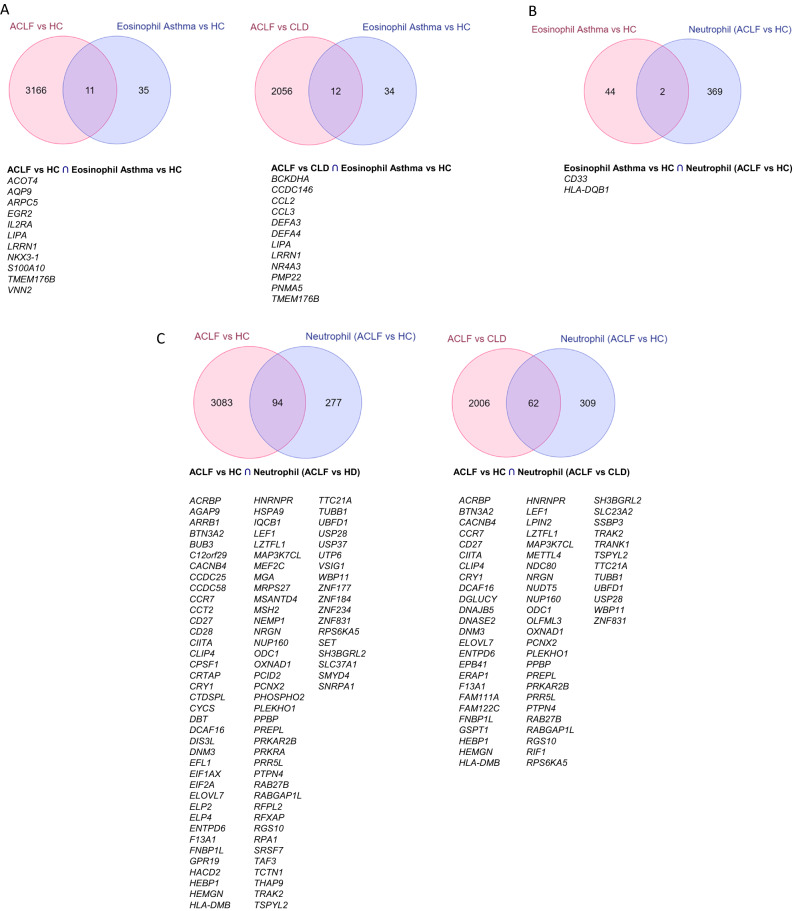
Comparative analysis of ACLF PMN DEG from our dataset with published eosinophil DEG from hypereosinophilia patients versus healthy controls and with recently published ACLF neutrophil DEG. (**A**) 11 of 3177 (0.34%) genes were common between ACLF versus HC (our dataset) and Eosinophil versus HC and 12 of 2068 genes (0.58%) were common between ACLF versus CLD (our dataset) and Eosinophil versus HC. (**B**) 2 of 371 genes (0.54%) were common between the DEG from a recently published ACLF neutrophil dataset (GSE142254) and the eosinophil dataset revealed an overlap of 2 of 371 genes (0.54%), which was comparable. (**C**) 94 genes were common in the comparative analysis of ACLF versus HC (our study) with the published ACLF neutrophil dataset (GSE142254; named as Neutrophil (ACLF vs HC)) and 62 were common between genes between ACLF versus CLD (our dataset) versus Neutrophil (ACLF vs HC).

**Figure 7:**
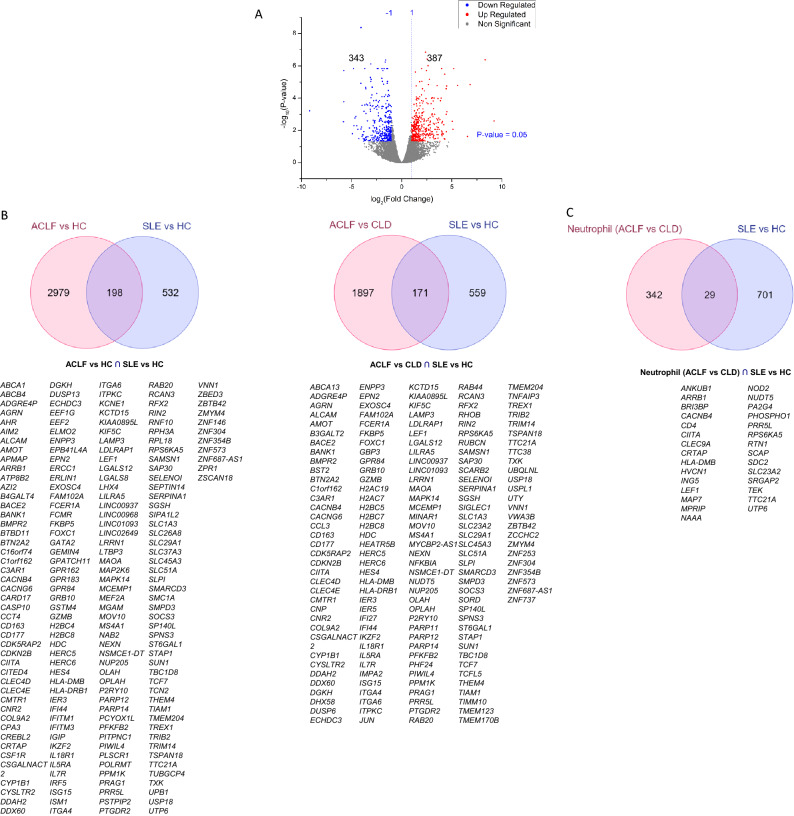
Comparative analysis of ACLF PMN DEG from our dataset with published enriched neutrophil dataset from systemic lupus erythematosus (SLE) patients (GSE153781). (**A**) Reanalysis of the GSE153781 showed 387 genes to be upregulated and 343 genes to be downregulated in the neutrophils of SLE versus HC. (**B**) 198 of 3177 genes and 171 of 2068 genes were common between ACLF versus HC (our dataset) and SLE versus HC; and ACLF versus CLD (our dataset) and SLE versus HC respectively. (**C**) Recently published ACLF neutrophil dataset (GSE142254) shared 29 of 371 genes with the SLE versus HC (GSE153781).

In order to assess the potential contamination of our ACLF PMN with eosinophils, a comparative analysis of our dataset was performed with published eosinophil dataset. The DEG profile of transcriptomics datasets were mined from literature for eosinophils^21^ (GSE65239). The common DEG in ACLF with respect to HC and CLD and mentioned datasets were illustrated using Venn diagrams (https://www.molbiotools.com/listcompare.php). The overlap between the DEG in ACLF versus HC (our dataset) and Eosinophil versus HC was 11 of 3177 genes (0.34%) and between ACLF versus CLD (our dataset) and Eosinophil versus HC, was 12 of 2068 genes (0.58%) (Fig. 6 A). Comparison between DEG from a recently published ACLF neutrophil dataset (GSE142254) and the eosinophil dataset revealed an overlap of 2 of 371 genes (0.54%), which was comparable (Fig. 6B). Comparative analysis of our ACLF dataset with the published ACLF neutrophil dataset (GSE142254; named as Neutrophil (ACLF vs HC)) revealed an overlap of 94 DEG between ACLF versus HC (our dataset) versus ACLF versus HC [Neutrophil (ACLF vs HC) in Fig. 6] and an overlap of 62 genes between ACLF versus CLD (our dataset) versus Neutrophil (ACLF vs HC) (Fig. 6 C). We further reanalyzed a submitted enriched neutrophil transcriptome dataset from SLE patients (GSE153781) and assessed the overlap between our DEG and the DEG of enriched neutrophils isolated from SLE (Fig. 7). Our analysis revealed that 387 genes were upregulated and 343 genes were downregulated in the neutrophils of SLE versus HC (Fig. 7A). A comparative analysis of our ACLF PMN dataset with SLE neutrophil DEG dataset revealed that 198 of 3177 genes and 171 of 2068 genes overlapped between ACLF versus HC (our dataset) and SLE versus HC and ACLF versus CLD (our dataset) and SLE versus HC respectively (Fig. 7 B). These genes included ELANE, MPO and CD177.We further assessed the overlap between recently published ACLF neutrophil dataset (GSE142254) and the SLE versus HC (GSE153781) and found that they shared 29 of 371 genes in common (Fig. 7C).

## Discussion

ACLF is driven by a poorly understood innate immune dysfunction which is linked to multiple organ failure and mortality^2–10^. An increase in neutrophil-to-lymphocyte (NLR) ratio has been reported as an important prognostic marker in ACLF. In fact, the NLR has been implicated as an important measurable prognostic parameter in several other inflammatory diseases such as sepsis, COVID-19 and kidney disease^27,28^). A recent study has explored the importance of NLR in all-cause mortality in USA and found a correlation between NLR and mortality^29^. Fundamentally, the NLR reflects the balance between systemic inflammation and targeted immune responses in the patient and possibly when systemic inflammation increases beyond a threshold, events leading to patient mortality occur. The association of NLR with mortality shows that neutrophil expansion is important in events leading to mortality. In our patient population also, NLR was found to be significantly higher in ACLF compared to the control groups CLD (Supplementary Fig. 1E). The gene expression analysis presented in our study, represents the transcriptomic landscape of the expanded population of granulocytes in ACLF of which neutrophils are an important component. Our primary findings are discussed below-

1. ***Overexpression of ELANE, MPO and CD177 in ACLF PMN.*** We found significantly higher overexpression of ELANE, MPO and CD177 genes in neutrophils from ACLF in comparison to neutrophils in CLD (compensated) and healthy controls (Fig. 2E). Over-expression of ELANE, MPO and CD177 in ACLF neutrophils was associated with higher 28-day mortality (Fig. 3A-C). Among the upregulated genes in ACLF , CD177 expression was highest (Fig. 2D). Such high CD177 gene upregulation was associated with significant increase in the corresponding neutrophil cell surface CD177 protein expression in ACLF compared to CLD and HC (~ 80% neutrophils in ACLF vs ~ 50% in CLD vs ~ 55% in healthy control; One way ANOVA p = 0.0002 and ACLF vs CLD *p* value < 0.0001) as captured by flow cytometric evaluation (Fig. 4C ). The expression of ELANE, MPO and CD177 was not influenced by sex or the presence of bacterial infection (Supplementary Figs. 1A-C, and Supplementary Fig. 2A-C). ELANE, MPO and CD177 are among the highest expressed genes in a variety of inflammatory disease^24,25,30–36^. Their high overexpression in ACLF patients as compared to controls, and their association with ACLF non-survivors suggests the involvement of neutrophil responses in ACLF pathogenesis leading to mortality. An independent study on ACLF blood immune signatures carried out in a European (French) population has also reported expansion of the CD177^+^ sub-population in ACLF^10^. This corroborates our observation in a genetically non-identical cohort of ACLF patients. Our study additionally shows that CD177 gene expression is associated with 28-day non-survivors. CD177^+^ neutrophils have been shown to be associated with increased ELANE and MPO gene expression, in agreement with our observations ^37^. Therefore ELANE, MPO and CD177 may form a three-gene signature that characterize a pathogenic neutrophil subset in neutrophil mediated pathogenesis in ACLF. Quantitative gene expression values of ELANE, MPO and CD177; or quantitative flowcytometric expression of CD177 on neutrophils may provide quantitative measures for evaluating the extent of inflammation and as a prognostic biomarker for short term mortality.
  Other genes found to be overexpressed in ACLF granulocytes were *OLFM4* (Olfactomedin 4) and *OLAH* (Oleoyl-ACP hydrolase). Although the precise mechanistic role of *OLFM4* in neutrophil is unknown, it has been shown to have a strong association with highly inflammatory conditions. *OLFM4* defines a neutrophil subset in healthy as well as diseased individuals ^38^. While *OLFM4* has not been shown to directly affect neutrophil functions such as phagocytosis or tissue migration, OLFM4+ neutrophil subsets have been found to be highly expanded and associated with mortality in inflammatory diseases with high mortality and morbidity such as sepsis, intestinal ischemia/ reperfusion injury ^39^ and, hemorrhagic shock. Our study reports the elevation of OLFM4 mRNA. *OLFM4* KO mice have been shown to have reduced ROS burst in neutrophils suggesting a role for *OLFM4* in promoting ROS mediated functions ^40^. However the role of OLAH in granulocyte function has not been described. *OLAH* (Oleoyl ACP hydrolase) catalyzes the release of fatty acids from fatty acid synthase enzyme. Among blood cells, it is neutrophil enriched (Supplementary Table 11). It can be postulated that the upregulation of *OLAH* in ACLF granulocytes might indicate upregulation of fatty acid release that can enter TCA cycle thereby generating high levels of ATP for neutrophil function. It has been shown that ATP generation through glycolysis as well as fatty acid metabolism through TCA cycle are important in ATP-requiring neutrophil functions such as NETosis ^41^. While the precise role of *OLAH* in neutrophil function still needs to be deciphered, we think that this enzyme may be involved in regulating energy production to facilitate neutrophil function.
2. ***CD177***^**+**^***Neutrophils are present in the liver tissue of deceased ACLF and CLD-AD patients.*** CD177 is a GPI-anchored cell surface glycoprotein which has been shown to bind the cell adhesion molecule PECAM-1 on endothelial cells^42^. CD177 participates in cell adhesion, tissue transmigration and neutrophil degranulation^43^. However, its exact role in tissue transmigration is controversial and it is not clear whether CD177^+^ neutrophils are capable of reaching target tissue sites, or not^44,45^. We retrieved archived FFPE liver biopsies from deceased ACLF and CLD-AD patients and carried out dual-colour IHC staining for CD16 and CD177 cell surface proteins. In the liver, CD16 is expressed by all cells of myeloid origin such as neutrophils, macrophages, Kupffer cells, and NK cells and CD177 is a neutrophil specific marker. Therefore CD16^+^ CD177^+^ double positives were scored as neutrophils in the liver biopsies (Fig. 5). Liver biopsies isolated from both deceased ACLF and CLD-AD patients were found to have CD177^+^ neutrophils suggesting that these cells are capable of tissue transmigration (Fig. 5). We also observed that in ACLF, patients with sepsis had greater number of CD177^+^ neutrophils in liver tissues as compared to patients without detectable sepsis (sterile inflammation) (Fig. 5G; *p* = 0.0635). Our observation is corroborated by recent studies carried out in periodontitis as a model for inflammation, that show that CD177^+^ neutrophils preferentially travel towards tissue sites with microbial inflammation as compared to tissue sites with sterile inflammation, although the levels of CD177^+^ neutrophils in circulation are elevated in both^44^. A substantial body of work suggests that the primary binding partner for CD177 on neutrophil surface is the platelet/ endothelial cell adhesion molecule 1 (PECAM1) ^46–48^. While PECAM1 has been established as a hallmark of endothelial cells, it was not clear if PECAM1 could also be expressed by other cell types. Hence, we investigated the expression of PECAM1 in the Human Protein Atlas database. We found that while endothelial cells had high expression of *PECAM1* mRNA, other cell types also expressed *PECAM1* mRNA at varying levels (Fig. 5I,J). Among blood cells , classical and non-classical monocytes, neutrophils, eosinophils had high expression of PECAM1 and CD4 naïve T cells, naïve and memory CD8 T cells, B cells and dendritic cells had low expression of PECAM 1 (Fig. 5I). Within the liver, endothelial cells, hepatic stellate cells (Ito cells), Kupffer cells, B cells and T cells were found to express *PECAM1* mRNA (Fig. 5J). While this is mRNA level expression that needs to be confirmed at the level of protein expression, the analysis suggests that non-endothelial cell types including hepatic stellate cells, B and T lymphocytes might be capable of synthesizing PECAM1 and therefore, may interact with CD177 + neutrophils. These interactions may further regulate inflammation or tissue injury within the liver. This is an area that needs further investigation and validation.
  The precise molecular function of the CD177^+^ neutrophils is not yet known. However, based on its presence in the liver tissue from our study, along with its reported ability to preferentially get recruited to septic tissues, adhesion to endothelial PECAM1 and, association with increased granule enzyme genes ELANE and MPO, we hypothesize that CD177^+^ neutrophils expressing high levels of cytotoxic enzymes ELANE and MPO reach the liver tissue in severe ACLF. However, the precise role of CD177^+^ neutrophils, and association with sepsis and the extensive cellular damage that is seen in ACLF, need further investigation^44^. Our study suggests that ACLF 28-day non-survivors have significantly higher levels of CD177^+^ neutrophils in circulation, indicating a role of the CD177^+^ neutrophil sub-population in ACLF pathogenesis, that needs to be further explored.
3. ***Upregulation of Inflammatory Pathways in ACLF PMN.*** Pathways analysis of overall transcriptomic signatures in ACLF versus CLD revealed the upregulation of inflammatory modules in ACLF PMN that resembled gene expression modules in several inflammatory diseases such as SLE, RA and IBD suggesting commonalities in PMN activation in these diseases (Supplementary Fig. 4). Neutrophil degranulation pathway was a part of the shared gene signature among the inflammatory diseases and may provide a basis for a common therapeutic target in inflammatory diseases that share this signature (Supplementary Table 10). Further, a comparative analysis of ACLF DEG from our dataset with ACLF DEG reported in the recently published study by Weiss et al., 2021 (GSE142254) revealed several shared DEG (Fig. 6C). These genes likely represent the most consistently expressed PMN genes which are differentially expressed in ACLF across study populations and ACLF definitions (Fig. 6C). We also found that our ACLF PMN dataset shared gene expression signatures with the transcriptome of enriched neutrophils from SLE patients that include the genes ELANE, MPO and CD177. This suggests that these three genes might constitute a damaging inflammatory gene expression signature that is common across diseases where inflammation mediated damage may be occurring.

A limitation of our study is the limited sample sizes included in various experiments. However, the observations made in the study, particularly in the context of ELANE, MPO and CD177, are supported by significant trends in spite of low sample sizes and therefore, cannot be ignored. We report novel associations between neutrophil-specific genes and ACLF outcomes. We also address the controversial question about the ability of CD177^+^ neutrophils to transmigrate to sites of tissue injury. Our study shows that they can indeed travel to the liver tissue in deceased ACLF and CLD-AD patients.

A major challenge in ACLF research is the heterogeneity of the disease in terms of variable clinical definitions of ACLF across continents, chronic and acute etiologies, presence or absence of sepsis and the involvement of multi-organ dysfunction. At the time of the acute super added liver injury, the functional liver reserve also vary which might influence the inflammatory and immune dysregulation. These variables are characteristic of ACLF and unfortunately cannot be avoided. This complicates studies on the pathogenesis of ACLF due the involvement of different kinds of PAMPS and DAMPS, interaction of different molecular and physiological processes^49^. Therefore, it is difficult to extrapolate findings from one study to another and this has been a major challenge in ACLF biomarker research^50,51^ . However, our observations which are made in an Indian cohort are corroborated by published studies carried out on genetically non-identical French population, particularly in the context of overexpression of neutrophil CD177^10^. This suggests that neutrophil specific CD177 expression may be a major conserved pathogenic signature in ACLF. CD177 expression has also been found to be high in ACLF whole blood transcriptome which suggests that whole blood gene expression profiling might be a good strategy for biomarker development^10^. While further investigations are needed to understand the mechanistic relevance of these genes and pathways in ACLF; our study provides a rational basis for further exploration of the ELANE, MPO, CD177 signature as potential biomarkers in predicting ACLF outcomes; as well as highlights these neutrophil granule genes and pathways as possible points of therapeutic intervention in ACLF.

## Supplementary Information

Supplementary Information 1.

Supplementary Information 2.

Supplementary Information 3.

Supplementary Information 4.

Supplementary Information 5.

Supplementary Information 6.

Supplementary Information 7.

Supplementary Information 8.

Supplementary Information 9.

Supplementary Information 10.

Supplementary Information 11.

Supplementary Information 12.

Supplementary Information 13.

Supplementary Information 14.

Supplementary Information 15.

Supplementary Information 16.

## Data Availability

GEO Accession Number for the Microarray Dataset: GSE156382. Reviewer access link: https://www.ncbi.nlm.nih.gov/geo/query/acc.cgi?acc=GSE156382 (token number: ojgjguqudjyrluf).
