# Supplementary material for: Inflammatory signature in acute-on-chronic liver failure includes increased expression of granulocyte genes ELANE, MPO and CD177
Source: Sci Rep. 2021 Sep 22;11:18849. doi: 10.1038/s41598-021-98086-6 (PMC8458283; doi:10.1038/s41598-021-98086-6)
Supplement: Supplementary file 1 — Supplementary Information 1. [file 41598_2021_98086_MOESM1_ESM.pptx]

## Slide 1
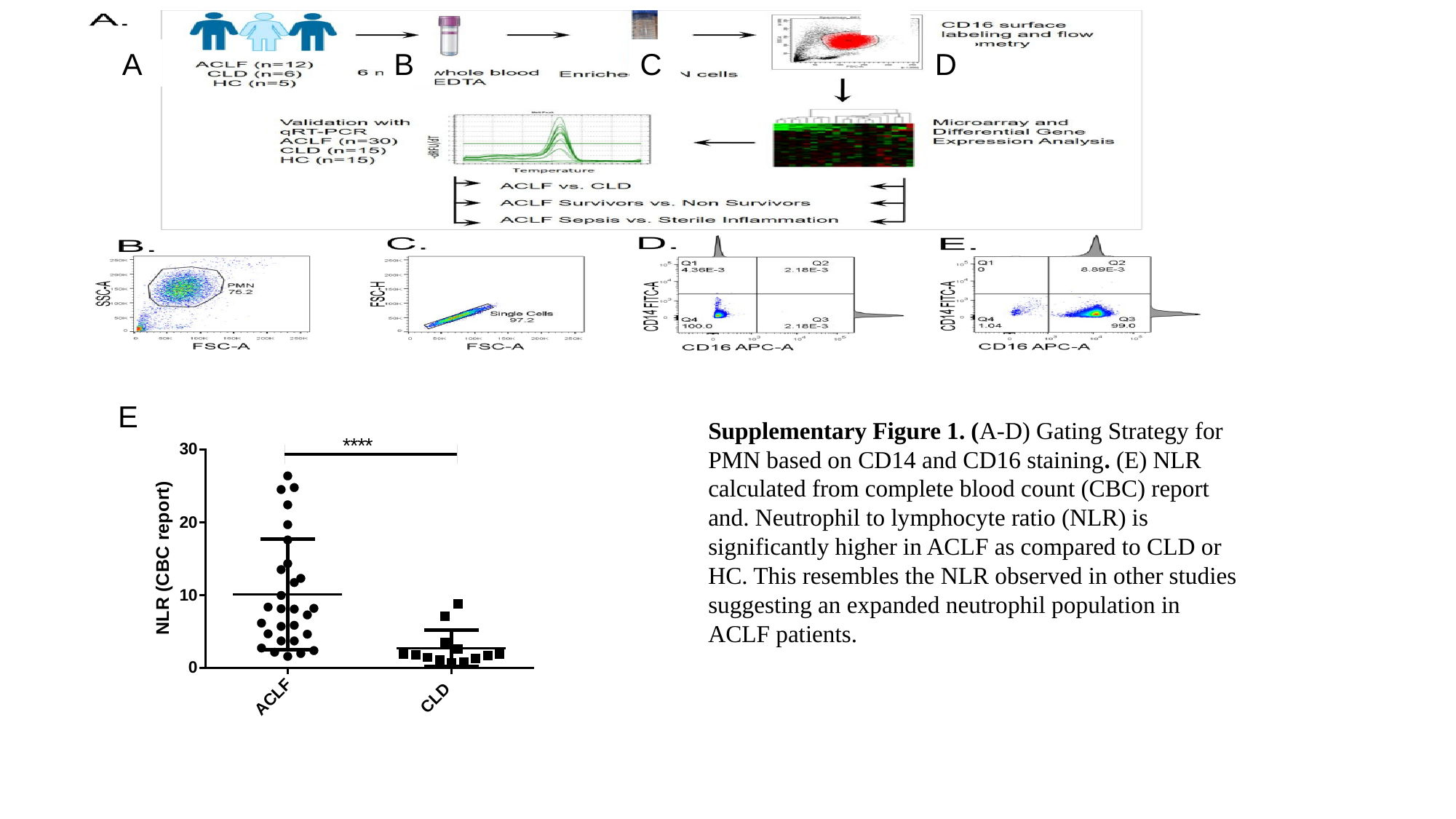

A
B
C
D
E
Supplementary Figure 1. (A-D) Gating Strategy for PMN based on CD14 and CD16 staining. (E) NLR calculated from complete blood count (CBC) report and. Neutrophil to lymphocyte ratio (NLR) is significantly higher in ACLF as compared to CLD or HC. This resembles the NLR observed in other studies suggesting an expanded neutrophil population in ACLF patients.
