# Supplementary material for: Inflammatory signature in acute-on-chronic liver failure includes increased expression of granulocyte genes ELANE, MPO and CD177
Source: Sci Rep. 2021 Sep 22;11:18849. doi: 10.1038/s41598-021-98086-6 (PMC8458283; doi:10.1038/s41598-021-98086-6)
Supplement: Supplementary file 2 — Supplementary Information 2. [file 41598_2021_98086_MOESM2_ESM.pptx]

## Slide 1
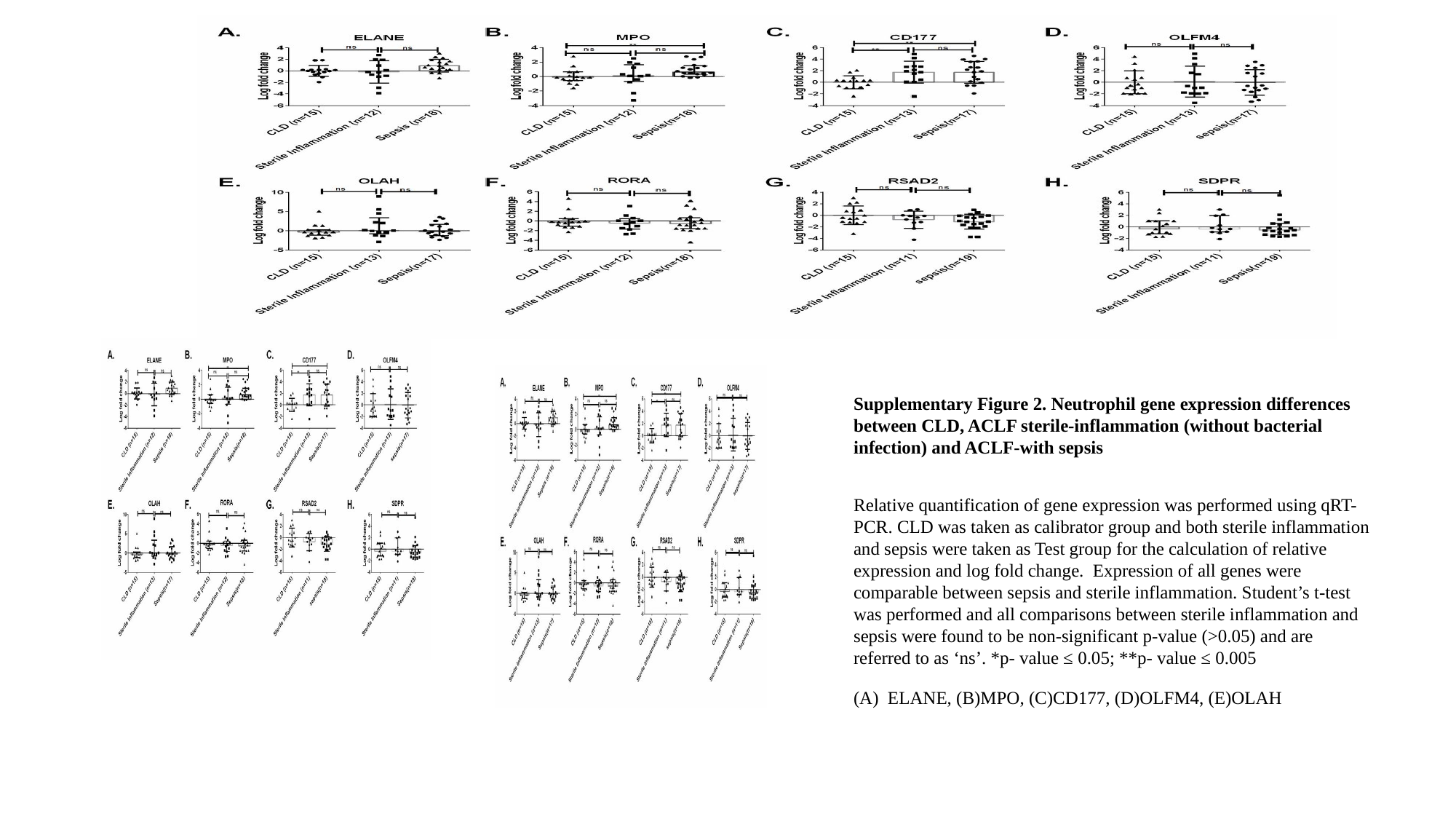

Supplementary Figure 2. Neutrophil gene expression differences between CLD, ACLF sterile-inflammation (without bacterial infection) and ACLF-with sepsis
Relative quantification of gene expression was performed using qRT-PCR. CLD was taken as calibrator group and both sterile inflammation and sepsis were taken as Test group for the calculation of relative expression and log fold change. Expression of all genes were comparable between sepsis and sterile inflammation. Student’s t-test was performed and all comparisons between sterile inflammation and sepsis were found to be non-significant p-value (>0.05) and are referred to as ‘ns’. *p- value ≤ 0.05; **p- value ≤ 0.005
(A) ELANE, (B)MPO, (C)CD177, (D)OLFM4, (E)OLAH
