# Supplementary material for: Inflammatory signature in acute-on-chronic liver failure includes increased expression of granulocyte genes ELANE, MPO and CD177
Source: Sci Rep. 2021 Sep 22;11:18849. doi: 10.1038/s41598-021-98086-6 (PMC8458283; doi:10.1038/s41598-021-98086-6)
Supplement: Supplementary file 3 — Supplementary Information 3. [file 41598_2021_98086_MOESM3_ESM.pptx]

## Slide 1
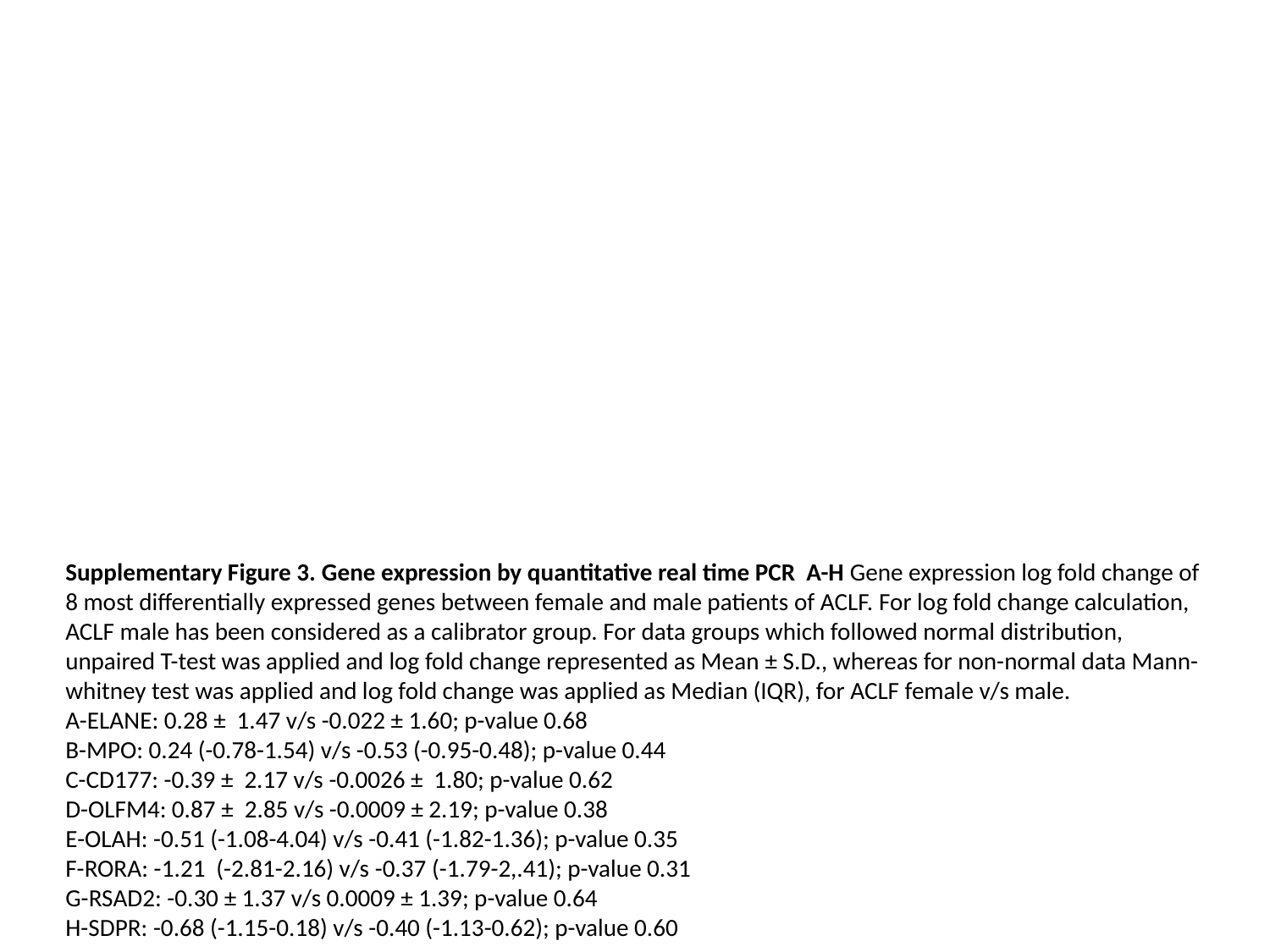

Supplementary Figure 3. Gene expression by quantitative real time PCR A-H Gene expression log fold change of 8 most differentially expressed genes between female and male patients of ACLF. For log fold change calculation, ACLF male has been considered as a calibrator group. For data groups which followed normal distribution, unpaired T-test was applied and log fold change represented as Mean ± S.D., whereas for non-normal data Mann-whitney test was applied and log fold change was applied as Median (IQR), for ACLF female v/s male.
A-ELANE: 0.28 ± 1.47 v/s -0.022 ± 1.60; p-value 0.68
B-MPO: 0.24 (-0.78-1.54) v/s -0.53 (-0.95-0.48); p-value 0.44
C-CD177: -0.39 ± 2.17 v/s -0.0026 ± 1.80; p-value 0.62
D-OLFM4: 0.87 ± 2.85 v/s -0.0009 ± 2.19; p-value 0.38
E-OLAH: -0.51 (-1.08-4.04) v/s -0.41 (-1.82-1.36); p-value 0.35
F-RORA: -1.21 (-2.81-2.16) v/s -0.37 (-1.79-2,.41); p-value 0.31
G-RSAD2: -0.30 ± 1.37 v/s 0.0009 ± 1.39; p-value 0.64
H-SDPR: -0.68 (-1.15-0.18) v/s -0.40 (-1.13-0.62); p-value 0.60
