# Supplementary material for: Inflammatory signature in acute-on-chronic liver failure includes increased expression of granulocyte genes ELANE, MPO and CD177
Source: Sci Rep. 2021 Sep 22;11:18849. doi: 10.1038/s41598-021-98086-6 (PMC8458283; doi:10.1038/s41598-021-98086-6)
Supplement: Supplementary file 4 — Supplementary Information 4. [file 41598_2021_98086_MOESM4_ESM.pptx]

## Slide 1
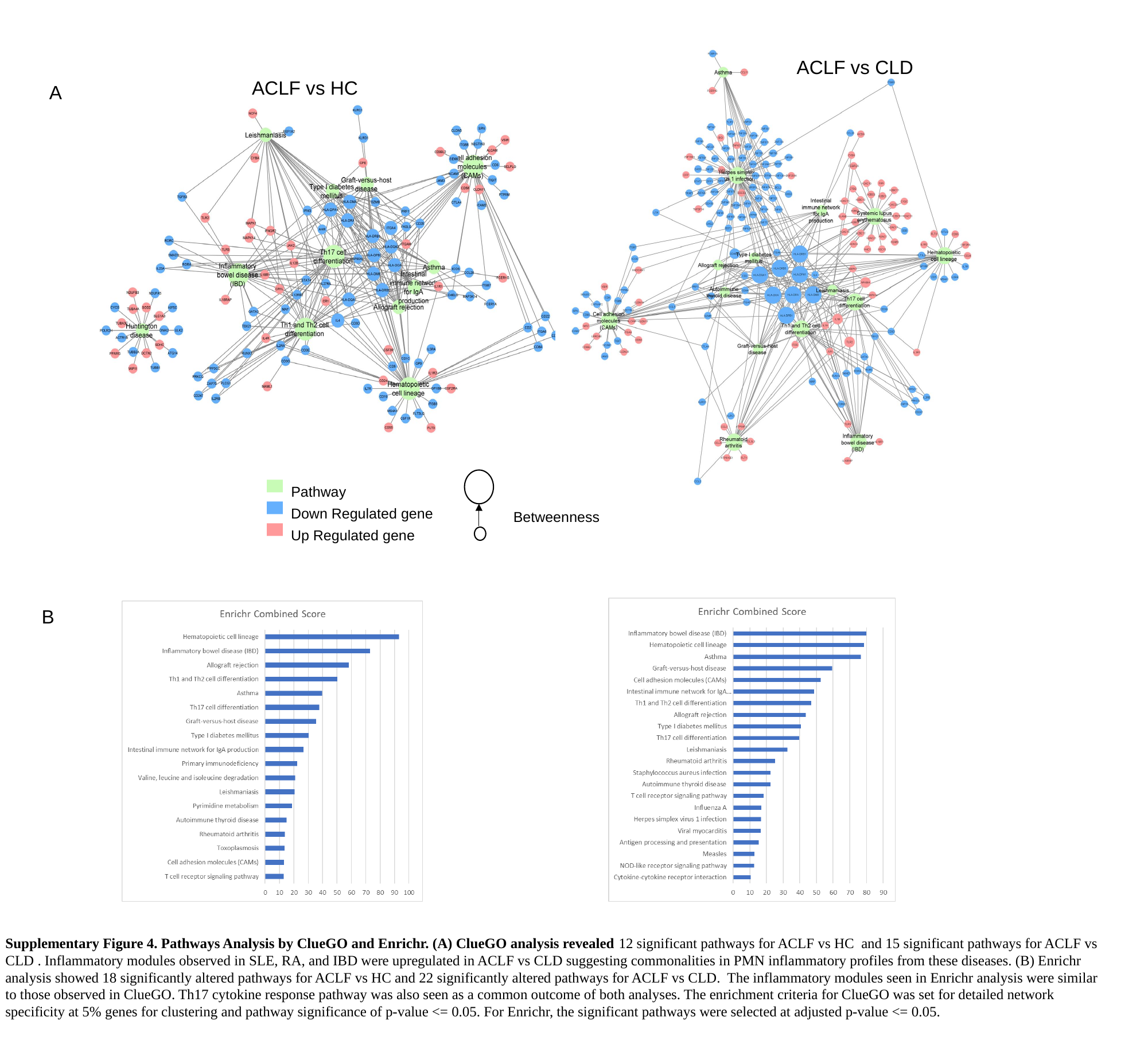

F
A
ACLF vs CLD
ACLF vs HC
A
Pathway
Down Regulated gene
Betweenness
Up Regulated gene
B
Supplementary Figure 4. Pathways Analysis by ClueGO and Enrichr. (A) ClueGO analysis revealed 12 significant pathways for ACLF vs HC and 15 significant pathways for ACLF vs CLD . Inflammatory modules observed in SLE, RA, and IBD were upregulated in ACLF vs CLD suggesting commonalities in PMN inflammatory profiles from these diseases. (B) Enrichr analysis showed 18 significantly altered pathways for ACLF vs HC and 22 significantly altered pathways for ACLF vs CLD. The inflammatory modules seen in Enrichr analysis were similar to those observed in ClueGO. Th17 cytokine response pathway was also seen as a common outcome of both analyses. The enrichment criteria for ClueGO was set for detailed network specificity at 5% genes for clustering and pathway significance of p-value <= 0.05. For Enrichr, the significant pathways were selected at adjusted p-value <= 0.05.
