# Supplementary material for: Inflammatory signature in acute-on-chronic liver failure includes increased expression of granulocyte genes ELANE, MPO and CD177
Source: Sci Rep. 2021 Sep 22;11:18849. doi: 10.1038/s41598-021-98086-6 (PMC8458283; doi:10.1038/s41598-021-98086-6)
Supplement: Supplementary file 5 — Supplementary Information 5. [file 41598_2021_98086_MOESM5_ESM.pptx]

## Slide 1
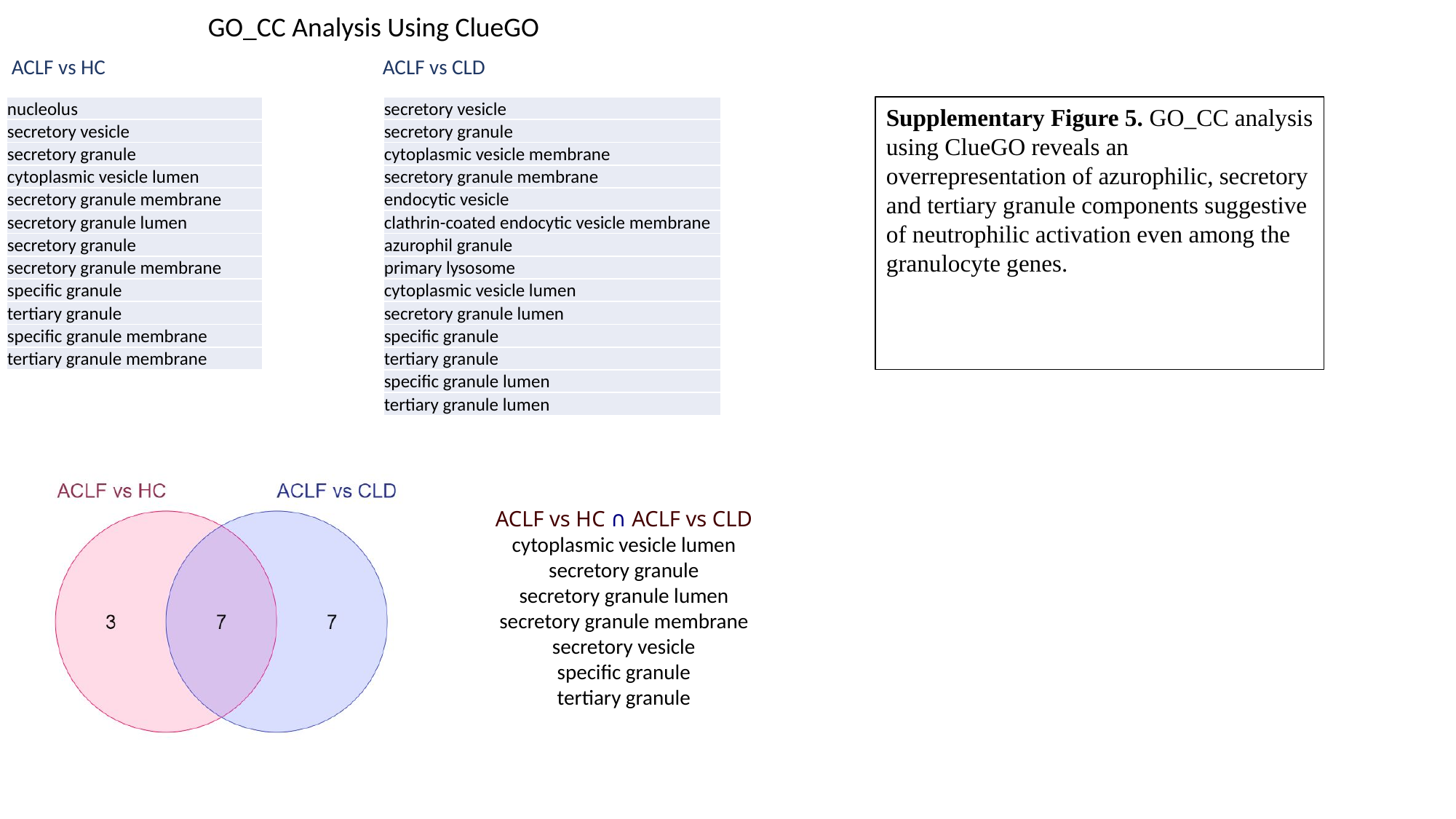

GO_CC Analysis Using ClueGO
ACLF vs HC
ACLF vs CLD
| nucleolus |
| --- |
| secretory vesicle |
| secretory granule |
| cytoplasmic vesicle lumen |
| secretory granule membrane |
| secretory granule lumen |
| secretory granule |
| secretory granule membrane |
| specific granule |
| tertiary granule |
| specific granule membrane |
| tertiary granule membrane |
| secretory vesicle |
| --- |
| secretory granule |
| cytoplasmic vesicle membrane |
| secretory granule membrane |
| endocytic vesicle |
| clathrin-coated endocytic vesicle membrane |
| azurophil granule |
| primary lysosome |
| cytoplasmic vesicle lumen |
| secretory granule lumen |
| specific granule |
| tertiary granule |
| specific granule lumen |
| tertiary granule lumen |
Supplementary Figure 5. GO_CC analysis using ClueGO reveals an overrepresentation of azurophilic, secretory and tertiary granule components suggestive of neutrophilic activation even among the granulocyte genes.
ACLF vs HC ∩ ACLF vs CLD
cytoplasmic vesicle lumen
secretory granule
secretory granule lumen
secretory granule membrane
secretory vesicle
specific granule
tertiary granule
