# Supplementary material for: Inflammatory signature in acute-on-chronic liver failure includes increased expression of granulocyte genes ELANE, MPO and CD177
Source: Sci Rep. 2021 Sep 22;11:18849. doi: 10.1038/s41598-021-98086-6 (PMC8458283; doi:10.1038/s41598-021-98086-6)
Supplement: Supplementary file 6 — Supplementary Information 6. [file 41598_2021_98086_MOESM6_ESM.docx]

**Supplementary Table 1.** **Flow cytometry analysis of PMN enrichment.** Whole blood was subjected to double gradient centrifugation, and PMN enriched pellet was used for antibody staining (CD14 and CD16) and acquired in BD Fourtesa Flow cytometer. According to the starting whole blood volume, number of cells were acquired (50,000 or 5000), and FSC-A v/s SSC-A was used for PMN gating. **%PMN enrichment** is referred to as the Percentage of cells in PMN gating over total cells acquired. **%CD16+** refers to the percentage of cells stained for CD16+CD14- inside the PMN gate.

| Sample ID | Total cells acquired | WBCs | | % PMN enrichment (as a % of WBCs) | % CD16+ |  |
| --- | --- | --- | --- | --- | --- | --- |
| ACLF_1 |  | | Flow data not available; microscopically evaluated enrichment = 79.0 | | |  |
| ACLF_2 | 50000 | 95.2 | | 89.5 | 97.9 |  |
| ACLF_3 | 50000 | 89.2 | | 70.3 | 99.7 |  |
| ACLF_4 | 50000 | 88.7 | | 83.7 | 95 |  |
| ACLF_5 | 50000 | 97.0 | | 45.1 | 74.6 |  |
| ACLF_6 | 50000 | 95.8 | | 93.5 | 67.1 |  |
| ACLF_7 | 50000 | 77.2 | | 81.0 | 97.9 |  |
| ACLF_8 | 50000 | 91.2 | | 88.8 | 99.4 |  |
| ACLF_9 | 50000 | 83.8 | | 80.2 | 98.2 |  |
| ACLF_10 | 50000 | 71.8 | | 78.8 | 97.5 |  |
|  |  |  | |  |  |  |
| CLD_1 |  | | Flow data not available; microscopically evaluated enrichment = 55.0 | | |  |
| CLD_2 |  | | Flow data not available; microscopically evaluated enrichment = 60.0 | | |  |
| CLD_3 | 5000 | 57.6 | | 41.2 | 84.2 |  |
| CLD_4 | 5000 | 65.4 | | 51.8 | 83.6 |  |
| CLD_5 | 5000 | 97.8 | | 52.8 | 76.0 |  |
| CLD_6 | 5000 | 47.0 | | 45.1 | 99.8 |  |
|  |  |  | |  |  |  |
| Healthy_1 | 50000 | 89.1 | | 68.5 | 97.3 |  |
| Healthy_2 | 50000 | 85.1 | | 76.7 | 90 |  |
| Healthy_3 | 5000 | 57.9 | | 74.7 | 98.6 |  |
| Healthy_4 | 5000 | 54.4 | | 82.7 | 94.3 |  |
| The median PMN enrichment was for ACLF ACLF 80.60 % (45.1-93); CLD 52.3 % (41.2-60); HC 75.7% (68.5-82.7). The median values for CD16^+^ neutrophils as a percentage of total PMN were - ACLF 97.9% (67-99) ; CLD 84% (76-95) ; HC 96 % (90-98-98.79). Note: Sample with no data available were prepared using the same enrichment protocol and proceeded with RNA isolation directly, due to low cell numbers for flow cytometry analysis. These were assessed using microscopy. | | | | | | |
