# Supplementary material for: Inflammatory signature in acute-on-chronic liver failure includes increased expression of granulocyte genes ELANE, MPO and CD177
Source: Sci Rep. 2021 Sep 22;11:18849. doi: 10.1038/s41598-021-98086-6 (PMC8458283; doi:10.1038/s41598-021-98086-6)
Supplement: Supplementary file 11 — Supplementary Information 11. [file 41598_2021_98086_MOESM11_ESM.docx]

**Supplementary Table 4:** **Whole blood neutrophil staining (CD16^+^CD66b^+^) and CD177^+^ positive percentage estimation**

Whole blood lysis was carried out and total leucocytes were acquired in flow cytometry. Number of cells acquired were noted in the given table. An initial gating encompassing all the whole blood cells, ‘WBCs’ was followed by PMN gating. PMN cells were gated based on FSC-SSC scatter plot, and the percentage of cells gated were recorded. Within PMN, single cell gating was done based on FSC-H vs FSC-A and the percentage of cells were recorded. A quadrant plot for CD66b-FITC stain vs CD16-APC stain was generated and the number of double positive cells were selected. CD177+ cells within the previous gating was recorded as percentage of CD177 positive cells. MFI (Median fluorescence Intensity) of CD177 positive cells were also recorded.

| Patient ID | Total Cells acquired | Total WBC (% of total) | PMN (% of total WBCs) | SINGLE CELL | CD16^+^CD66b^+^ (% of single cells) | CD177 (% of CD16^+^CD177^+^) |
| --- | --- | --- | --- | --- | --- | --- |
| ACLF_229 | 50000 | 24.2 | 57.5 | 95.4 | 96.6 | 64.4 |
| ACLF_231 | 10000 | 18.2 | 63.0 | 92.8 | 96.0 | 85.7 |
| ACLF_232 | 50000 | 20.4 | 69.7 | 92.8 | 80.6 | 77.6 |
| ACLF_234 | 50000 | 20.0 | 46 | 99.0 | 80.6 | 72.5 |
| ACLF_226 | 50000 | 68.4 | 78.5 | 98.4 | 86.3 | 99.5 |
| ACLF_225 | 10000 | 57.0 | 81.4 | 96.1 | 61.4 | 88.2 |
| ACLF_236 | 50000 | 12.2 | 25.8 | 95.4 | 93.5 | 68.5 |
| ACLF_227 | 15000 | 52.3 | 78.2 | 96.7 | 60.3 | 97.1 |
| ACLF_233 | 50000 | 32.9 | 74.2 | 92.6 | 91.2 | 93.3 |
| ACLF_228 | 60000 | 69.3 | 68.7 | 98.9 | 96.7 | 95.7 |
|  |  |  |  |  |  |  |
| HC_6579 | 500000 | 12.0 | 28.5 | 93.3 | 96.3 | 75.8 |
| HC_6580 | 500000 | 6.91 | 27.4 | 85.3 | 94.2 | 78.5 |
| HC_6581 | 500000 | 29.2 | 25.1 | 97.9 | 85.3 | 69.7 |
| HC_6582 | 500000 | 17.0 | 28.4 | 93.8 | 98.6 | 61.6 |
| HC_6583 | 500000 | 15.0 | 31.8 | 94.3 | 90.4 | 32.3 |
| HC_1 | 500000 | 8.87 | 30.4 | 95.2 | 93.1 | 43.1 |
| HC_2 | 500000 | 10.1 | 33.2 | 90.4 | 93.1 | 95.3 |
| HC_3 | 500000 | 9.61 | 22.4 | 94.6 | 95.4 | 46.8 |
| HC_4 | 500000 | 12.4 | 21.4 | 93.1 | 92.1 | 24.2 |
| HC_5 | 500000 | 7.49 | 15.9 | 93.7 | 93.4 | 49.4 |
|  |  |  |  |  |  |  |
| CLD_195 | 50000 | 19.5 | 50.4 | 92.2 | 96.1 | 69.1 |
| CLD_193 | 60000 | 12.4 | 50.7 | 85.6 | 94.2 | 46.4 |
| CLD_192 | 50000 | 41.8 | 67.6 | 96.8 | 99.2 | 52.2 |
| CLD_196 | 50000 | 20.9 | 42.6 | 94.7 | 97.1 | 44.9 |
| CLD_183 | 50000 | 10.5 | 48.5 | 93.2 | 98.8 | 46.2 |
| CLD_184 | 50000 | 11.3 | 27.3 | 84.6 | 97.7 | 53.1 |
| CLD_186 | 50000 | 41.7 | 57.8 | 94.2 | 98.6 | 64.6 |
| CLD_187 | 50000 | 32.3 | 21.2 | 92.1 | 81.9 | 33.3 |
| CLD_188 | 50000 | 12.5 | 48.1 | 89.7 | 98.8 | 58.0 |
| CLD_189 | 50000 | 17.1 | 35.0 | 88.2 | 97.2 | 34.6 |
