# Supplementary material for: Inflammatory signature in acute-on-chronic liver failure includes increased expression of granulocyte genes ELANE, MPO and CD177
Source: Sci Rep. 2021 Sep 22;11:18849. doi: 10.1038/s41598-021-98086-6 (PMC8458283; doi:10.1038/s41598-021-98086-6)
Supplement: Supplementary file 7 — Supplementary Information 7. [file 41598_2021_98086_MOESM7_ESM.pdf]

overlap

Overlap Results		
Collection(s):	CP	
# overlaps shown:	10	
# genesets in collections:	2922	
# genes in comparison (n):	2005	
# genes in universe (N):	40312	

Gene Set Name	# Genes in Gene Set (K)	Description	# Genes in Overlap (k)	k/K	p-value	FDR q-value
REACTOME_INNATE_IMMUNE_SYSTEM	1117	Innate Immune System	157	0.1406	3.38E-32	9.86E-29
REACTOME_NEUTROPHIL_DEGRANULATION	479	Neutrophil degranulation	91	0.1900	1.57E-28	2.3E-25
REACTOME_CYTOKINE_SIGNALING_IN_IMMUNE_SYSTEM	719	Cytokine Signaling in Immune system	114	0.1586	3.9E-28	3.8E-25
REACTOME_POST_TRANSLATIONAL_PROTEIN_MODIFICATION	1435	Post-translational protein modification	161	0.1122	3.37E-22	2.46E-19
REACTOME_SIGNALING_BY_GPCR	698	Signaling by GPCR	97	0.1390	6.44E-20	3.76E-17
REACTOME_RNA_POLYMERASE_II_TRANSCRIPTION	1374	RNA Polymerase II Transcription	149	0.1084	3.09E-19	1.51E-16
REACTOME_HEMOSTASIS	678	Hemostasis	93	0.1372	9.1E-19	3.8E-16
KEGG_CELL_ADHESION_MOLECULES_CAMS	133	Cell adhesion molecules (CAMs)	36	0.2707	3.29E-17	1.2E-14
KEGG_SYSTEMIC_LUPUS_ERYTHEMATOSUS	139	Systemic lupus erythematosus	36	0.2590	1.54E-16	5.01E-14
KEGG_CYTOKINE_CYTOKINE_RECEPTOR_INTERACTION	265	Cytokine-cytokine receptor interaction	50	0.1887	3.61E-16	1.06E-13

Gene/Gene Set Overlap Matrix

Entrez Gene Id	Gene Symbol	Gene Description	REACTOME_INNATE_IMMUNE_SYSTEM	REACTOME_NEUTROPHIL_DEGRANULATION	REACTOME_CYTOKINE_SIGNALING_IN_IMMUNE_SYSTEM	REACTOME_POST_TRANSLATIONAL_PROTEIN_MODIFICATION	REACTOME_SIGNALING_BY_GPCR	REACTOME_RNA_POLYMERASE_II_TRANSCRIPTION	REACTOME_HEMOSTASIS	KEGG_CELL_ADHESION_MOLECULES_CAMS	KEGG_SYSTEMIC_LUPUS_ERYTHEMATOSUS	KEGG_CYTOKINE_CYTOKINE_RECEPTOR_INTERACTION
5594	MAPK1	mitogen-activated protein kinase 1 [Source:HGNC Symbol;Acc:HGNC:6871]	REACTOME_INNATE_IMMUNE_SYSTEM	REACTOME_NEUTROPHIL_DEGRANULATION	REACTOME_CYTOKINE_SIGNALING_IN_IMMUNE_SYSTEM		REACTOME_SIGNALING_BY_GPCR	REACTOME_RNA_POLYMERASE_II_TRANSCRIPTION	REACTOME_HEMOSTASIS			
1432	MAPK14	mitogen-activated protein kinase 14 [Source:HGNC Symbol;Acc:HGNC:6876]	REACTOME_INNATE_IMMUNE_SYSTEM	REACTOME_NEUTROPHIL_DEGRANULATION	REACTOME_CYTOKINE_SIGNALING_IN_IMMUNE_SYSTEM		REACTOME_SIGNALING_BY_GPCR	REACTOME_RNA_POLYMERASE_II_TRANSCRIPTION	REACTOME_HEMOSTASIS			
5657	PRTN3	proteinase 3 [Source:HGNC Symbol;Acc:HGNC:9495]	REACTOME_INNATE_IMMUNE_SYSTEM	REACTOME_NEUTROPHIL_DEGRANULATION	REACTOME_CYTOKINE_SIGNALING_IN_IMMUNE_SYSTEM				REACTOME_HEMOSTASIS			
1511	CTSG	cathepsin G [Source:HGNC Symbol;Acc:HGNC:2532]	REACTOME_INNATE_IMMUNE_SYSTEM	REACTOME_NEUTROPHIL_DEGRANULATION	REACTOME_CYTOKINE_SIGNALING_IN_IMMUNE_SYSTEM						KEGG_SYSTEMIC_LUPUS_ERYTHEMATOSUS	
4318	MMP9	matrix metallopeptidase 9 [Source:HGNC Symbol;Acc:HGNC:7176]	REACTOME_INNATE_IMMUNE_SYSTEM	REACTOME_NEUTROPHIL_DEGRANULATION	REACTOME_CYTOKINE_SIGNALING_IN_IMMUNE_SYSTEM							
6283	S100A12	S100 calcium binding protein A12 [Source:HGNC Symbol;Acc:HGNC:10489]	REACTOME_INNATE_IMMUNE_SYSTEM	REACTOME_NEUTROPHIL_DEGRANULATION	REACTOME_CYTOKINE_SIGNALING_IN_IMMUNE_SYSTEM							
3934	LCN2	lipocalin 2 [Source:HGNC Symbol;Acc:HGNC:6526]	REACTOME_INNATE_IMMUNE_SYSTEM	REACTOME_NEUTROPHIL_DEGRANULATION	REACTOME_CYTOKINE_SIGNALING_IN_IMMUNE_SYSTEM							
54472	TOLLIP	toll interacting protein [Source:HGNC Symbol;Acc:HGNC:16476]	REACTOME_INNATE_IMMUNE_SYSTEM	REACTOME_NEUTROPHIL_DEGRANULATION	REACTOME_CYTOKINE_SIGNALING_IN_IMMUNE_SYSTEM							
684	BST2	bone marrow stromal cell antigen 2 [Source:HGNC Symbol;Acc:HGNC:1119]	REACTOME_INNATE_IMMUNE_SYSTEM	REACTOME_NEUTROPHIL_DEGRANULATION	REACTOME_CYTOKINE_SIGNALING_IN_IMMUNE_SYSTEM							
387	RHOA	ras homolog family member A [Source:HGNC Symbol;Acc:HGNC:667]	REACTOME_INNATE_IMMUNE_SYSTEM	REACTOME_NEUTROPHIL_DEGRANULATION		REACTOME_POST_TRANSLATIONAL_PROTEIN_MODIFICATION	REACTOME_SIGNALING_BY_GPCR		REACTOME_HEMOSTASIS			
1604	CD55	CD55 molecule (Cromer blood group) [Source:HGNC Symbol;Acc:HGNC:2665]	REACTOME_INNATE_IMMUNE_SYSTEM	REACTOME_NEUTROPHIL_DEGRANULATION		REACTOME_POST_TRANSLATIONAL_PROTEIN_MODIFICATION	REACTOME_SIGNALING_BY_GPCR					
5265	SERPINA1	serpin family A member 1 [Source:HGNC Symbol;Acc:HGNC:8941]	REACTOME_INNATE_IMMUNE_SYSTEM	REACTOME_NEUTROPHIL_DEGRANULATION		REACTOME_POST_TRANSLATIONAL_PROTEIN_MODIFICATION			REACTOME_HEMOSTASIS			
5768	QSOX1	quiescin sulfhydryl oxidase 1 [Source:HGNC Symbol;Acc:HGNC:9756]	REACTOME_INNATE_IMMUNE_SYSTEM	REACTOME_NEUTROPHIL_DEGRANULATION		REACTOME_POST_TRANSLATIONAL_PROTEIN_MODIFICATION			REACTOME_HEMOSTASIS			
1075	CTSC	cathepsin C [Source:HGNC Symbol;Acc:HGNC:2528]	REACTOME_INNATE_IMMUNE_SYSTEM	REACTOME_NEUTROPHIL_DEGRANULATION		REACTOME_POST_TRANSLATIONAL_PROTEIN_MODIFICATION						
5199	CFP	complement factor properdin [Source:HGNC Symbol;Acc:HGNC:8864]	REACTOME_INNATE_IMMUNE_SYSTEM	REACTOME_NEUTROPHIL_DEGRANULATION		REACTOME_POST_TRANSLATIONAL_PROTEIN_MODIFICATION						
58485	TRAPPC1	trafficking protein particle complex 1 [Source:HGNC Symbol;Acc:HGNC:19894]	REACTOME_INNATE_IMMUNE_SYSTEM	REACTOME_NEUTROPHIL_DEGRANULATION		REACTOME_POST_TRANSLATIONAL_PROTEIN_MODIFICATION						
683	BST1	bone marrow stromal cell antigen 1 [Source:HGNC Symbol;Acc:HGNC:1118]	REACTOME_INNATE_IMMUNE_SYSTEM	REACTOME_NEUTROPHIL_DEGRANULATION		REACTOME_POST_TRANSLATIONAL_PROTEIN_MODIFICATION						
8876	VNN 1.00	vanin 1 [Source:HGNC Symbol;Acc:HGNC:12705]	REACTOME_INNATE_IMMUNE_SYSTEM	REACTOME_NEUTROPHIL_DEGRANULATION		REACTOME_POST_TRANSLATIONAL_PROTEIN_MODIFICATION						
401258	RAB44	RAB44, member RAS oncogene family [Source:HGNC Symbol;Acc:HGNC:21068]	REACTOME_INNATE_IMMUNE_SYSTEM	REACTOME_NEUTROPHIL_DEGRANULATION		REACTOME_POST_TRANSLATIONAL_PROTEIN_MODIFICATION						
5473	PPBP	pro-platelet basic protein [Source:HGNC Symbol;Acc:HGNC:9240]	REACTOME_INNATE_IMMUNE_SYSTEM	REACTOME_NEUTROPHIL_DEGRANULATION			REACTOME_SIGNALING_BY_GPCR		REACTOME_HEMOSTASIS			KEGG_CYTOKINE_CYTOKINE_RECEPTOR_INTERACTION
719	C3AR1	complement C3a receptor 1 [Source:HGNC Symbol;Acc:HGNC:1319]	REACTOME_INNATE_IMMUNE_SYSTEM	REACTOME_NEUTROPHIL_DEGRANULATION			REACTOME_SIGNALING_BY_GPCR					
2358	FPR2	formyl peptide receptor 2 [Source:HGNC Symbol;Acc:HGNC:3827]	REACTOME_INNATE_IMMUNE_SYSTEM	REACTOME_NEUTROPHIL_DEGRANULATION			REACTOME_SIGNALING_BY_GPCR					
53831	GPR84	G protein-coupled receptor 84 [Source:HGNC Symbol;Acc:HGNC:4535]	REACTOME_INNATE_IMMUNE_SYSTEM	REACTOME_NEUTROPHIL_DEGRANULATION			REACTOME_SIGNALING_BY_GPCR					
56729	RETN	resistin [Source:HGNC Symbol;Acc:HGNC:20389]	REACTOME_INNATE_IMMUNE_SYSTEM	REACTOME_NEUTROPHIL_DEGRANULATION				REACTOME_RNA_POLYMERASE_II_TRANSCRIPTION				
965	CD58	CD58 molecule [Source:HGNC Symbol;Acc:HGNC:1688]	REACTOME_INNATE_IMMUNE_SYSTEM	REACTOME_NEUTROPHIL_DEGRANULATION					REACTOME_HEMOSTASIS	KEGG_CELL_ADHESION_MOLECULES_CAMS		
391	RHOG	ras homolog family member G [Source:HGNC Symbol;Acc:HGNC:672]	REACTOME_INNATE_IMMUNE_SYSTEM	REACTOME_NEUTROPHIL_DEGRANULATION					REACTOME_HEMOSTASIS			
2207	FCER1G	Fc fragment of IgE receptor Ig [Source:HGNC Symbol;Acc:HGNC:3611]	REACTOME_INNATE_IMMUNE_SYSTEM	REACTOME_NEUTROPHIL_DEGRANULATION					REACTOME_HEMOSTASIS			
226	ALDOA	aldolase, fructose-bisphosphate A [Source:HGNC Symbol;Acc:HGNC:414]	REACTOME_INNATE_IMMUNE_SYSTEM	REACTOME_NEUTROPHIL_DEGRANULATION					REACTOME_HEMOSTASIS			
5004	ORM1	orosomucoid 1 [Source:HGNC Symbol;Acc:HGNC:8498]	REACTOME_INNATE_IMMUNE_SYSTEM	REACTOME_NEUTROPHIL_DEGRANULATION					REACTOME_HEMOSTASIS			
5005	ORM2	orosomucoid 2 [Source:HGNC Symbol;Acc:HGNC:8499]	REACTOME_INNATE_IMMUNE_SYSTEM	REACTOME_NEUTROPHIL_DEGRANULATION					REACTOME_HEMOSTASIS			
967	CD63	CD63 molecule [Source:HGNC Symbol;Acc:HGNC:1692]	REACTOME_INNATE_IMMUNE_SYSTEM	REACTOME_NEUTROPHIL_DEGRANULATION					REACTOME_HEMOSTASIS			
1088	CEACAM8	CEA cell adhesion molecule 8 [Source:HGNC Symbol;Acc:HGNC:1820]	REACTOME_INNATE_IMMUNE_SYSTEM	REACTOME_NEUTROPHIL_DEGRANULATION					REACTOME_HEMOSTASIS			
4680	CEACAM6	CEA cell adhesion molecule 6 [Source:HGNC Symbol;Acc:HGNC:1818]	REACTOME_INNATE_IMMUNE_SYSTEM	REACTOME_NEUTROPHIL_DEGRANULATION					REACTOME_HEMOSTASIS			
57126	CD177	CD177 molecule [Source:HGNC Symbol;Acc:HGNC:30072]	REACTOME_INNATE_IMMUNE_SYSTEM	REACTOME_NEUTROPHIL_DEGRANULATION					REACTOME_HEMOSTASIS			
5547	PRCP	prolylcarboxypeptidase [Source:HGNC Symbol;Acc:HGNC:9344]	REACTOME_INNATE_IMMUNE_SYSTEM	REACTOME_NEUTROPHIL_DEGRANULATION					REACTOME_HEMOSTASIS			
1991	ELANE	elastase, neutrophil expressed [Source:HGNC Symbol;Acc:HGNC:3309]	REACTOME_INNATE_IMMUNE_SYSTEM	REACTOME_NEUTROPHIL_DEGRANULATION							KEGG_SYSTEMIC_LUPUS_ERYTHEMATOSUS	
2212	FCGR2A	Fc fragment of IgG receptor IIa [Source:HGNC Symbol;Acc:HGNC:3616]	REACTOME_INNATE_IMMUNE_SYSTEM	REACTOME_NEUTROPHIL_DEGRANULATION							KEGG_SYSTEMIC_LUPUS_ERYTHEMATOSUS	
1535	CYBA	cytochrome b-245 alpha chain [Source:HGNC Symbol;Acc:HGNC:2577]	REACTOME_INNATE_IMMUNE_SYSTEM	REACTOME_NEUTROPHIL_DEGRANULATION								
7097	TLR2	toll like receptor 2 [Source:HGNC Symbol;Acc:HGNC:11848]	REACTOME_INNATE_IMMUNE_SYSTEM	REACTOME_NEUTROPHIL_DEGRANULATION								
126014	OSCAR	osteoclast associated Ig-like receptor [Source:HGNC Symbol;Acc:HGNC:29960]	REACTOME_INNATE_IMMUNE_SYSTEM	REACTOME_NEUTROPHIL_DEGRANULATION								
27180	SIGLEC9	sialic acid binding Ig like lectin 9 [Source:HGNC Symbol;Acc:HGNC:10878]	REACTOME_INNATE_IMMUNE_SYSTEM	REACTOME_NEUTROPHIL_DEGRANULATION								
3903	LAIR1	leukocyte associated immunoglobulin like receptor 1 [Source:HGNC Symbol;Acc:H	REACTOME_INNATE_IMMUNE_SYSTEM	REACTOME_NEUTROPHIL_DEGRANULATION								
8778	SIGLEC5	sialic acid binding Ig like lectin 5 [Source:HGNC Symbol;Acc:HGNC:10874]	REACTOME_INNATE_IMMUNE_SYSTEM	REACTOME_NEUTROPHIL_DEGRANULATION								
4317	MMP8	matrix metallopeptidase 8 [Source:HGNC Symbol;Acc:HGNC:7175]	REACTOME_INNATE_IMMUNE_SYSTEM	REACTOME_NEUTROPHIL_DEGRANULATION								
6286	S100P	S100 calcium binding protein P [Source:HGNC Symbol;Acc:HGNC:10504]	REACTOME_INNATE_IMMUNE_SYSTEM	REACTOME_NEUTROPHIL_DEGRANULATION								
1992	SERPINB1	serpin family B member 1 [Source:HGNC Symbol;Acc:HGNC:3311]	REACTOME_INNATE_IMMUNE_SYSTEM	REACTOME_NEUTROPHIL_DEGRANULATION								
6282	S100A11	S100 calcium binding protein A11 [Source:HGNC Symbol;Acc:HGNC:10488]	REACTOME_INNATE_IMMUNE_SYSTEM	REACTOME_NEUTROPHIL_DEGRANULATION								
6590	SLPI	secretory leukocyte peptidase inhibitor [Source:HGNC Symbol;Acc:HGNC:11092]	REACTOME_INNATE_IMMUNE_SYSTEM	REACTOME_NEUTROPHIL_DEGRANULATION								
5273	SERPINB10	serpin family B member 10 [Source:HGNC Symbol;Acc:HGNC:8942]	REACTOME_INNATE_IMMUNE_SYSTEM	REACTOME_NEUTROPHIL_DEGRANULATION								
338339	CLEC4D	C-type lectin domain family 4 member D [Source:HGNC Symbol;Acc:HGNC:14554	REACTOME_INNATE_IMMUNE_SYSTEM	REACTOME_NEUTROPHIL_DEGRANULATION								
160364	CLEC12A	C-type lectin domain family 12 member A [Source:HGNC Symbol;Acc:HGNC:3171	REACTOME_INNATE_IMMUNE_SYSTEM	REACTOME_NEUTROPHIL_DEGRANULATION								
10875	FGL2	fibrinogen like 2 [Source:HGNC Symbol;Acc:HGNC:3696]	REACTOME_INNATE_IMMUNE_SYSTEM	REACTOME_NEUTROPHIL_DEGRANULATION								
116844	LRG1	leucine rich alpha-2-glycoprotein 1 [Source:HGNC Symbol;Acc:HGNC:29480]	REACTOME_INNATE_IMMUNE_SYSTEM	REACTOME_NEUTROPHIL_DEGRANULATION								
7130	TNFAIP6	TNF alpha induced protein 6 [Source:HGNC Symbol;Acc:HGNC:11898]	REACTOME_INNATE_IMMUNE_SYSTEM	REACTOME_NEUTROPHIL_DEGRANULATION								
4057	LTF	lactotransferrin [Source:HGNC Symbol;Acc:HGNC:6720]	REACTOME_INNATE_IMMUNE_SYSTEM	REACTOME_NEUTROPHIL_DEGRANULATION								
10555	AGPAT2	1-acylglycerol-3-phosphate O-acyltransferase 2 [Source:HGNC Symbol;Acc:HGNC	REACTOME_INNATE_IMMUNE_SYSTEM	REACTOME_NEUTROPHIL_DEGRANULATION								
9218	VAPA	VAMP associated protein A [Source:HGNC Symbol;Acc:HGNC:12648]	REACTOME_INNATE_IMMUNE_SYSTEM	REACTOME_NEUTROPHIL_DEGRANULATION								
383	ARG1	arginase 1 [Source:HGNC Symbol;Acc:HGNC:663]	REACTOME_INNATE_IMMUNE_SYSTEM	REACTOME_NEUTROPHIL_DEGRANULATION								
3848	KRT1	keratin 1 [Source:HGNC Symbol;Acc:HGNC:6412]	REACTOME_INNATE_IMMUNE_SYSTEM	REACTOME_NEUTROPHIL_DEGRANULATION								
81619	TSPAN14	tetraspanin 14 [Source:HGNC Symbol;Acc:HGNC:23303]	REACTOME_INNATE_IMMUNE_SYSTEM	REACTOME_NEUTROPHIL_DEGRANULATION								
7226	TRPM2	transient receptor potential cation channel subfamily M member 2 [Source:HGNC S	REACTOME_INNATE_IMMUNE_SYSTEM	REACTOME_NEUTROPHIL_DEGRANULATION								
2512	FTL	ferritin light chain [Source:HGNC Symbol;Acc:HGNC:3999]	REACTOME_INNATE_IMMUNE_SYSTEM	REACTOME_NEUTROPHIL_DEGRANULATION								
6556	SLC11A1	solute carrier family 11 member 1 [Source:HGNC Symbol;Acc:HGNC:10907]	REACTOME_INNATE_IMMUNE_SYSTEM	REACTOME_NEUTROPHIL_DEGRANULATION								
196527	ANO6	anoctamin 6 [Source:HGNC Symbol;Acc:HGNC:25240]	REACTOME_INNATE_IMMUNE_SYSTEM	REACTOME_NEUTROPHIL_DEGRANULATION								
121260	SLC15A4	solute carrier family 15 member 4 [Source:HGNC Symbol;Acc:HGNC:23090]	REACTOME_INNATE_IMMUNE_SYSTEM	REACTOME_NEUTROPHIL_DEGRANULATION								
3304	HSPA1B	heat shock protein family A (Hsp70) member 1B [Source:HGNC Symbol;Acc:HGNC	REACTOME_INNATE_IMMUNE_SYSTEM	REACTOME_NEUTROPHIL_DEGRANULATION								
80184	CEP290	centrosomal protein 290 [Source:HGNC Symbol;Acc:HGNC:29021]	REACTOME_INNATE_IMMUNE_SYSTEM	REACTOME_NEUTROPHIL_DEGRANULATION								
2992	GYG1	glycogenin 1 [Source:HGNC Symbol;Acc:HGNC:4699]	REACTOME_INNATE_IMMUNE_SYSTEM	REACTOME_NEUTROPHIL_DEGRANULATION								
3101	HK3	hexokinase 3 [Source:HGNC Symbol;Acc:HGNC:4925]	REACTOME_INNATE_IMMUNE_SYSTEM	REACTOME_NEUTROPHIL_DEGRANULATION								
5836	PYGL	glycogen phosphorylase L [Source:HGNC Symbol;Acc:HGNC:9725]	REACTOME_INNATE_IMMUNE_SYSTEM	REACTOME_NEUTROPHIL_DEGRANULATION								
8993	PGLYRP1	peptidoglycan recognition protein 1 [Source:HGNC Symbol;Acc:HGNC:8904]	REACTOME_INNATE_IMMUNE_SYSTEM	REACTOME_NEUTROPHIL_DEGRANULATION								
4353	MPO	myeloperoxidase [Source:HGNC Symbol;Acc:HGNC:7218]	REACTOME_INNATE_IMMUNE_SYSTEM	REACTOME_NEUTROPHIL_DEGRANULATION								
3240	HP	haptoglobin [Source:HGNC Symbol;Acc:HGNC:5141]	REACTOME_INNATE_IMMUNE_SYSTEM	REACTOME_NEUTROPHIL_DEGRANULATION								
1669	DEFA4	defensin alpha 4 [Source:HGNC Symbol;Acc:HGNC:2763]	REACTOME_INNATE_IMMUNE_SYSTEM	REACTOME_NEUTROPHIL_DEGRANULATION								
2352	FOLR3	folate receptor gamma [Source:HGNC Symbol;Acc:HGNC:3795]	REACTOME_INNATE_IMMUNE_SYSTEM	REACTOME_NEUTROPHIL_DEGRANULATION								
51125	GOLGA7	golgin A7 [Source:HGNC Symbol;Acc:HGNC:24876]	REACTOME_INNATE_IMMUNE_SYSTEM	REACTOME_NEUTROPHIL_DEGRANULATION								
978	CDA	cytidine deaminase [Source:HGNC Symbol;Acc:HGNC:1712]	REACTOME_INNATE_IMMUNE_SYSTEM	REACTOME_NEUTROPHIL_DEGRANULATION								
154664	ABCA13	ATP binding cassette subfamily A member 13 [Source:HGNC Symbol;Acc:HGNC:1	REACTOME_INNATE_IMMUNE_SYSTEM	REACTOME_NEUTROPHIL_DEGRANULATION								
1118	CHIT1	chitinase 1 [Source:HGNC Symbol;Acc:HGNC:1936]	REACTOME_INNATE_IMMUNE_SYSTEM	REACTOME_NEUTROPHIL_DEGRANULATION								
51316	PLAC8	placenta associated 8 [Source:HGNC Symbol;Acc:HGNC:19254]	REACTOME_INNATE_IMMUNE_SYSTEM	REACTOME_NEUTROPHIL_DEGRANULATION								
8836	GGH	gamma-glutamyl hydrolase [Source:HGNC Symbol;Acc:HGNC:4248]	REACTOME_INNATE_IMMUNE_SYSTEM	REACTOME_NEUTROPHIL_DEGRANULATION								
10562	OLFM4	olfactomedin 4 [Source:HGNC Symbol;Acc:HGNC:17190]	REACTOME_INNATE_IMMUNE_SYSTEM	REACTOME_NEUTROPHIL_DEGRANULATION								
199675	MCEMP1	mast cell expressed membrane protein 1 [Source:HGNC Symbol;Acc:HGNC:27291	REACTOME_INNATE_IMMUNE_SYSTEM	REACTOME_NEUTROPHIL_DEGRANULATION								
23071	ERP44	endoplasmic reticulum protein 44 [Source:HGNC Symbol;Acc:HGNC:18311]	REACTOME_INNATE_IMMUNE_SYSTEM	REACTOME_NEUTROPHIL_DEGRANULATION								
23593	HEBP2	heme binding protein 2 [Source:HGNC Symbol;Acc:HGNC:15716]	REACTOME_INNATE_IMMUNE_SYSTEM	REACTOME_NEUTROPHIL_DEGRANULATION								
441864	TARM1	T cell-interacting, activating receptor on myeloid cells 1 [Source:HGNC Symbol;Ac	REACTOME_INNATE_IMMUNE_SYSTEM	REACTOME_NEUTROPHIL_DEGRANULATION								
566	AZU1	azurocidin 1 [Source:HGNC Symbol;Acc:HGNC:913]	REACTOME_INNATE_IMMUNE_SYSTEM	REACTOME_NEUTROPHIL_DEGRANULATION								
64114	TMBIM1	transmembrane BAX inhibitor motif containing 1 [Source:HGNC Symbol;Acc:HGNC	REACTOME_INNATE_IMMUNE_SYSTEM	REACTOME_NEUTROPHIL_DEGRANULATION								
79930	DOK3	docking protein 3 [Source:HGNC Symbol;Acc:HGNC:24583]	REACTOME_INNATE_IMMUNE_SYSTEM	REACTOME_NEUTROPHIL_DEGRANULATION								
84418	CYSTM1	cysteine rich transmembrane module containing 1 [Source:HGNC Symbol;Acc:HGI	REACTOME_INNATE_IMMUNE_SYSTEM	REACTOME_NEUTROPHIL_DEGRANULATION								
932	MS4A3	membrane spanning 4-domains A3 [Source:HGNC Symbol;Acc:HGNC:7317]	REACTOME_INNATE_IMMUNE_SYSTEM	REACTOME_NEUTROPHIL_DEGRANULATION								
5721	PSME2	proteasome activator subunit 2 [Source:HGNC Symbol;Acc:HGNC:9569]	REACTOME_INNATE_IMMUNE_SYSTEM		REACTOME_CYTOKINE_SIGNALING_IN_IMMUNE_SYSTEM	REACTOME_POST_TRANSLATIONAL_PROTEIN_MODIFICATION		REACTOME_RNA_POLYMERASE_II_TRANSCRIPTION				
7334	UBE2N	ubiquitin conjugating enzyme E2 N [Source:HGNC Symbol;Acc:HGNC:12492]	REACTOME_INNATE_IMMUNE_SYSTEM		REACTOME_CYTOKINE_SIGNALING_IN_IMMUNE_SYSTEM	REACTOME_POST_TRANSLATIONAL_PROTEIN_MODIFICATION						
4792	NFKBIA	NFKB inhibitor alpha [Source:HGNC Symbol;Acc:HGNC:7797]	REACTOME_INNATE_IMMUNE_SYSTEM		REACTOME_CYTOKINE_SIGNALING_IN_IMMUNE_SYSTEM	REACTOME_POST_TRANSLATIONAL_PROTEIN_MODIFICATION						
4582	MUC1	mucin 1, cell surface associated [Source:HGNC Symbol;Acc:HGNC:7508]	REACTOME_INNATE_IMMUNE_SYSTEM		REACTOME_CYTOKINE_SIGNALING_IN_IMMUNE_SYSTEM	REACTOME_POST_TRANSLATIONAL_PROTEIN_MODIFICATION						
10392	NOD1	nucleotide binding oligomerization domain containing 1 [Source:HGNC Symbol;Ac	REACTOME_INNATE_IMMUNE_SYSTEM		REACTOME_CYTOKINE_SIGNALING_IN_IMMUNE_SYSTEM	REACTOME_POST_TRANSLATIONAL_PROTEIN_MODIFICATION						
23586	DDX58	DExD/H-box helicase 58 [Source:HGNC Symbol;Acc:HGNC:19102]	REACTOME_INNATE_IMMUNE_SYSTEM		REACTOME_CYTOKINE_SIGNALING_IN_IMMUNE_SYSTEM	REACTOME_POST_TRANSLATIONAL_PROTEIN_MODIFICATION						
3725	JUN	Jun proto-oncogene, AP-1 transcription factor subunit [Source:HGNC Symbol;Acc	REACTOME_INNATE_IMMUNE_SYSTEM		REACTOME_CYTOKINE_SIGNALING_IN_IMMUNE_SYSTEM			REACTOME_RNA_POLYMERASE_II_TRANSCRIPTION				
2353	FOS	Fos proto-oncogene, AP-1 transcription factor subunit [Source:HGNC Symbol;Acc	REACTOME_INNATE_IMMUNE_SYSTEM		REACTOME_CYTOKINE_SIGNALING_IN_IMMUNE_SYSTEM			REACTOME_RNA_POLYMERASE_II_TRANSCRIPTION				
5775	PTPN4	protein tyrosine phosphatase non-receptor type 4 [Source:HGNC Symbol;Acc:HGN	REACTOME_INNATE_IMMUNE_SYSTEM		REACTOME_CYTOKINE_SIGNALING_IN_IMMUNE_SYSTEM			REACTOME_RNA_POLYMERASE_II_TRANSCRIPTION				
2534	FYN	FYN proto-oncogene, Src family tyrosine kinase [Source:HGNC Symbol;Acc:HGNC	REACTOME_INNATE_IMMUNE_SYSTEM		REACTOME_CYTOKINE_SIGNALING_IN_IMMUNE_SYSTEM				REACTOME_HEMOSTASIS			
3553	IL1B	interleukin 1 beta [Source:HGNC Symbol;Acc:HGNC:5992]	REACTOME_INNATE_IMMUNE_SYSTEM		REACTOME_CYTOKINE_SIGNALING_IN_IMMUNE_SYSTEM							KEGG_CYTOKINE_CYTOKINE_RECEPTOR_INTERACTION
51191	HERC5	HECT and RLD domain containing E3 ubiquitin protein ligase 5 [Source:HGNC Sym	REACTOME_INNATE_IMMUNE_SYSTEM		REACTOME_CYTOKINE_SIGNALING_IN_IMMUNE_SYSTEM							
3162	HMOX1	heme oxygenase 1 [Source:HGNC Symbol;Acc:HGNC:5013]	REACTOME_INNATE_IMMUNE_SYSTEM		REACTOME_CYTOKINE_SIGNALING_IN_IMMUNE_SYSTEM							
596	BCL2	BCL2 apoptosis regulator [Source:HGNC Symbol;Acc:HGNC:990]	REACTOME_INNATE_IMMUNE_SYSTEM		REACTOME_CYTOKINE_SIGNALING_IN_IMMUNE_SYSTEM							
9252	RPS6KA5	ribosomal protein S6 kinase A5 [Source:HGNC Symbol;Acc:HGNC:10434]	REACTOME_INNATE_IMMUNE_SYSTEM		REACTOME_CYTOKINE_SIGNALING_IN_IMMUNE_SYSTEM							
1848	DUSP6	dual specificity phosphatase 6 [Source:HGNC Symbol;Acc:HGNC:3072]	REACTOME_INNATE_IMMUNE_SYSTEM		REACTOME_CYTOKINE_SIGNALING_IN_IMMUNE_SYSTEM							
11213	IRAK3	interleukin 1 receptor associated kinase 3 [Source:HGNC Symbol;Acc:HGNC:1702	REACTOME_INNATE_IMMUNE_SYSTEM		REACTOME_CYTOKINE_SIGNALING_IN_IMMUNE_SYSTEM							
9636	ISG15	ISG15 ubiquitin like modifier [Source:HGNC Symbol;Acc:HGNC:4053]	REACTOME_INNATE_IMMUNE_SYSTEM		REACTOME_CYTOKINE_SIGNALING_IN_IMMUNE_SYSTEM							
801	CALM1	calmodulin 1 [Source:HGNC Symbol;Acc:HGNC:1442]	REACTOME_INNATE_IMMUNE_SYSTEM			REACTOME_POST_TRANSLATIONAL_PROTEIN_MODIFICATION	REACTOME_SIGNALING_BY_GPCR	REACTOME_RNA_POLYMERASE_II_TRANSCRIPTION	REACTOME_HEMOSTASIS			
720	C4A	complement C4A (Rodgers blood group) [Source:HGNC Symbol;Acc:HGNC:1323]	REACTOME_INNATE_IMMUNE_SYSTEM			REACTOME_POST_TRANSLATIONAL_PROTEIN_MODIFICATION					KEGG_SYSTEMIC_LUPUS_ERYTHEMATOSUS	
118430	MUCL1	mucin like 1 [Source:HGNC Symbol;Acc:HGNC:30588]	REACTOME_INNATE_IMMUNE_SYSTEM			REACTOME_POST_TRANSLATIONAL_PROTEIN_MODIFICATION						
114548	NLRP3	NLR family pyrin domain containing 3 [Source:HGNC Symbol;Acc:HGNC:16400]	REACTOME_INNATE_IMMUNE_SYSTEM			REACTOME_POST_TRANSLATIONAL_PROTEIN_MODIFICATION						
7128	TNFAIP3	TNF alpha induced protein 3 [Source:HGNC Symbol;Acc:HGNC:11896]	REACTOME_INNATE_IMMUNE_SYSTEM			REACTOME_POST_TRANSLATIONAL_PROTEIN_MODIFICATION						
84282	RNF135	ring finger protein 135 [Source:HGNC Symbol;Acc:HGNC:21158]	REACTOME_INNATE_IMMUNE_SYSTEM			REACTOME_POST_TRANSLATIONAL_PROTEIN_MODIFICATION						
5588	PRKCQ	protein kinase C theta [Source:HGNC Symbol;Acc:HGNC:9410]	REACTOME_INNATE_IMMUNE_SYSTEM				REACTOME_SIGNALING_BY_GPCR	REACTOME_RNA_POLYMERASE_II_TRANSCRIPTION	REACTOME_HEMOSTASIS			
3710	ITPR3	inositol 1,4,5-trisphosphate receptor type 3 [Source:HGNC Symbol;Acc:HGNC:618	REACTOME_INNATE_IMMUNE_SYSTEM				REACTOME_SIGNALING_BY_GPCR		REACTOME_HEMOSTASIS			
5567	PRKACB	protein kinase cAMP-activated catalytic subunit beta [Source:HGNC Symbol;Acc:H	REACTOME_INNATE_IMMUNE_SYSTEM				REACTOME_SIGNALING_BY_GPCR		REACTOME_HEMOSTASIS			
10125	RASGRP1	RAS guanyl releasing protein 1 [Source:HGNC Symbol;Acc:HGNC:9878]	REACTOME_INNATE_IMMUNE_SYSTEM				REACTOME_SIGNALING_BY_GPCR		REACTOME_HEMOSTASIS			
58484	NLRC4	NLR family CARD domain containing 4 [Source:HGNC Symbol;Acc:HGNC:16412]	REACTOME_INNATE_IMMUNE_SYSTEM					REACTOME_RNA_POLYMERASE_II_TRANSCRIPTION				
5027	P2RX7	purinergic receptor P2X 7 [Source:HGNC Symbol;Acc:HGNC:8537]	REACTOME_INNATE_IMMUNE_SYSTEM						REACTOME_HEMOSTASIS			
3384	ICAM2	intercellular adhesion molecule 2 [Source:HGNC Symbol;Acc:HGNC:5345]	REACTOME_INNATE_IMMUNE_SYSTEM							KEGG_CELL_ADHESION_MOLECULES_CAMS		
714	C1QC	complement C1q C chain [Source:HGNC Symbol;Acc:HGNC:1245]	REACTOME_INNATE_IMMUNE_SYSTEM								KEGG_SYSTEMIC_LUPUS_ERYTHEMATOSUS	
917	CD3G	CD3g molecule [Source:HGNC Symbol;Acc:HGNC:1675]	REACTOME_INNATE_IMMUNE_SYSTEM									
919	CD247	CD247 molecule [Source:HGNC Symbol;Acc:HGNC:1677]	REACTOME_INNATE_IMMUNE_SYSTEM									
3824	KLRD1	killer cell lectin like receptor D1 [Source:HGNC Symbol;Acc:HGNC:6378]	REACTOME_INNATE_IMMUNE_SYSTEM									
975	CD81	CD81 molecule [Source:HGNC Symbol;Acc:HGNC:1701]	REACTOME_INNATE_IMMUNE_SYSTEM									
26052	DNM3	dynamin 3 [Source:HGNC Symbol;Acc:HGNC:29125]	REACTOME_INNATE_IMMUNE_SYSTEM									
3702	ITK	IL2 inducible T cell kinase [Source:HGNC Symbol;Acc:HGNC:6171]	REACTOME_INNATE_IMMUNE_SYSTEM									
10892	MALT1	MALT1 paracaspase [Source:HGNC Symbol;Acc:HGNC:6819]	REACTOME_INNATE_IMMUNE_SYSTEM									
22914	KLRK1	killer cell lectin like receptor K1 [Source:HGNC Symbol;Acc:HGNC:18788]	REACTOME_INNATE_IMMUNE_SYSTEM									
4153	MBL2	mannose binding lectin 2 [Source:HGNC Symbol;Acc:HGNC:6922]	REACTOME_INNATE_IMMUNE_SYSTEM									
55600	ITLN1	intelectin 1 [Source:HGNC Symbol;Acc:HGNC:18259]	REACTOME_INNATE_IMMUNE_SYSTEM									
26253	CLEC4E	C-type lectin domain family 4 member E [Source:HGNC Symbol;Acc:HGNC:14555	REACTOME_INNATE_IMMUNE_SYSTEM									
10095	ARPC1B	actin related protein 2/3 complex subunit 1B [Source:HGNC Symbol;Acc:HGNC:70	REACTOME_INNATE_IMMUNE_SYSTEM									
10810	WASF3	WASP family member 3 [Source:HGNC Symbol;Acc:HGNC:12734]	REACTOME_INNATE_IMMUNE_SYSTEM									
4651	MYO10	myosin X [Source:HGNC Symbol;Acc:HGNC:7593]	REACTOME_INNATE_IMMUNE_SYSTEM									
51284	TLR7	toll like receptor 7 [Source:HGNC Symbol;Acc:HGNC:15631]	REACTOME_INNATE_IMMUNE_SYSTEM									
4771	NF2	neurofibromin 2 [Source:HGNC Symbol;Acc:HGNC:7773]	REACTOME_INNATE_IMMUNE_SYSTEM									
10578	GNLY	granulysin [Source:HGNC Symbol;Acc:HGNC:4414]	REACTOME_INNATE_IMMUNE_SYSTEM									
2205	FCER1A	Fc fragment of IgE receptor Ia [Source:HGNC Symbol;Acc:HGNC:3609]	REACTOME_INNATE_IMMUNE_SYSTEM									
8992	ATP6V0E1	ATPase H+ transporting V0 subunit e1 [Source:HGNC Symbol;Acc:HGNC:863]	REACTOME_INNATE_IMMUNE_SYSTEM									
24145	PANX1	pannexin 1 [Source:HGNC Symbol;Acc:HGNC:8599]	REACTOME_INNATE_IMMUNE_SYSTEM									
79132	DHX58	DExH-box helicase 58 [Source:HGNC Symbol;Acc:HGNC:29517]	REACTOME_INNATE_IMMUNE_SYSTEM									
51295	ECSIT	ECSIT signaling integrator [Source:HGNC Symbol;Acc:HGNC:29548]	REACTOME_INNATE_IMMUNE_SYSTEM									
7098	TLR3	toll like receptor 3 [Source:HGNC Symbol;Acc:HGNC:11849]	REACTOME_INNATE_IMMUNE_SYSTEM									
115004	CGAS	cyclic GMP-AMP synthase [Source:HGNC Symbol;Acc:HGNC:21367]	REACTOME_INNATE_IMMUNE_SYSTEM									
11277	TREX1	three prime repair exonuclease 1 [Source:HGNC Symbol;Acc:HGNC:12269]	REACTOME_INNATE_IMMUNE_SYSTEM									
9533	POLR1C	RNA polymerase I and III subunit C [Source:HGNC Symbol;Acc:HGNC:20194]	REACTOME_INNATE_IMMUNE_SYSTEM									
7294	TXK	TXK tyrosine kinase [Source:HGNC Symbol;Acc:HGNC:12434]	REACTOME_INNATE_IMMUNE_SYSTEM									
7100	TLR5	toll like receptor 5 [Source:HGNC Symbol;Acc:HGNC:11851]	REACTOME_INNATE_IMMUNE_SYSTEM									
1668	DEFA3	defensin alpha 3 [Source:HGNC Symbol;Acc:HGNC:2762]	REACTOME_INNATE_IMMUNE_SYSTEM									
414325	DEFB103A	defensin beta 103A [Source:HGNC Symbol;Acc:HGNC:15967]	REACTOME_INNATE_IMMUNE_SYSTEM									
92747	BPIFB1	BPI fold containing family B member 1 [Source:HGNC Symbol;Acc:HGNC:16108]	REACTOME_INNATE_IMMUNE_SYSTEM									
725	C4BPB	complement component 4 binding protein beta [Source:HGNC Symbol;Acc:HGNC	REACTOME_INNATE_IMMUNE_SYSTEM									
55718	POLR3E	RNA polymerase III subunit E [Source:HGNC Symbol;Acc:HGNC:30347]	REACTOME_INNATE_IMMUNE_SYSTEM									
54941	RNF125	ring finger protein 125 [Source:HGNC Symbol;Acc:HGNC:21150]	REACTOME_INNATE_IMMUNE_SYSTEM									
2625	GATA3	GATA binding protein 3 [Source:HGNC Symbol;Acc:HGNC:4172]			REACTOME_CYTOKINE_SIGNALING_IN_IMMUNE_SYSTEM	REACTOME_POST_TRANSLATIONAL_PROTEIN_MODIFICATION		REACTOME_RNA_POLYMERASE_II_TRANSCRIPTION	REACTOME_HEMOSTASIS			
9021	SOCS3	suppressor of cytokine signaling 3 [Source:HGNC Symbol;Acc:HGNC:19391]			REACTOME_CYTOKINE_SIGNALING_IN_IMMUNE_SYSTEM	REACTOME_POST_TRANSLATIONAL_PROTEIN_MODIFICATION		REACTOME_RNA_POLYMERASE_II_TRANSCRIPTION				
332	BIRC5	baculoviral IAP repeat containing 5 [Source:HGNC Symbol;Acc:HGNC:593]			REACTOME_CYTOKINE_SIGNALING_IN_IMMUNE_SYSTEM	REACTOME_POST_TRANSLATIONAL_PROTEIN_MODIFICATION		REACTOME_RNA_POLYMERASE_II_TRANSCRIPTION				
6095	RORA	RAR related orphan receptor A [Source:HGNC Symbol;Acc:HGNC:10258]			REACTOME_CYTOKINE_SIGNALING_IN_IMMUNE_SYSTEM	REACTOME_POST_TRANSLATIONAL_PROTEIN_MODIFICATION		REACTOME_RNA_POLYMERASE_II_TRANSCRIPTION				
4088	SMAD3	SMAD family member 3 [Source:HGNC Symbol;Acc:HGNC:6769]			REACTOME_CYTOKINE_SIGNALING_IN_IMMUNE_SYSTEM	REACTOME_POST_TRANSLATIONAL_PROTEIN_MODIFICATION		REACTOME_RNA_POLYMERASE_II_TRANSCRIPTION				
7076	TIMP1	TIMP metallopeptidase inhibitor 1 [Source:HGNC Symbol;Acc:HGNC:11820]			REACTOME_CYTOKINE_SIGNALING_IN_IMMUNE_SYSTEM	REACTOME_POST_TRANSLATIONAL_PROTEIN_MODIFICATION			REACTOME_HEMOSTASIS			
8835	SOCS2	suppressor of cytokine signaling 2 [Source:HGNC Symbol;Acc:HGNC:19382]			REACTOME_CYTOKINE_SIGNALING_IN_IMMUNE_SYSTEM	REACTOME_POST_TRANSLATIONAL_PROTEIN_MODIFICATION						
23279	NUP160	nucleoporin 160 [Source:HGNC Symbol;Acc:HGNC:18017]			REACTOME_CYTOKINE_SIGNALING_IN_IMMUNE_SYSTEM	REACTOME_POST_TRANSLATIONAL_PROTEIN_MODIFICATION						
23165	NUP205	nucleoporin 205 [Source:HGNC Symbol;Acc:HGNC:18658]			REACTOME_CYTOKINE_SIGNALING_IN_IMMUNE_SYSTEM	REACTOME_POST_TRANSLATIONAL_PROTEIN_MODIFICATION						
9688	NUP93	nucleoporin 93 [Source:HGNC Symbol;Acc:HGNC:28958]			REACTOME_CYTOKINE_SIGNALING_IN_IMMUNE_SYSTEM	REACTOME_POST_TRANSLATIONAL_PROTEIN_MODIFICATION						
11274	USP18	ubiquitin specific peptidase 18 [Source:HGNC Symbol;Acc:HGNC:12616]			REACTOME_CYTOKINE_SIGNALING_IN_IMMUNE_SYSTEM	REACTOME_POST_TRANSLATIONAL_PROTEIN_MODIFICATION						
9306	SOCS6	suppressor of cytokine signaling 6 [Source:HGNC Symbol;Acc:HGNC:16833]			REACTOME_CYTOKINE_SIGNALING_IN_IMMUNE_SYSTEM	REACTOME_POST_TRANSLATIONAL_PROTEIN_MODIFICATION						
6352	CCL5	C-C motif chemokine ligand 5 [Source:HGNC Symbol;Acc:HGNC:10632]			REACTOME_CYTOKINE_SIGNALING_IN_IMMUNE_SYSTEM		REACTOME_SIGNALING_BY_GPCR					KEGG_CYTOKINE_CYTOKINE_RECEPTOR_INTERACTION
6364	CCL20	C-C motif chemokine ligand 20 [Source:HGNC Symbol;Acc:HGNC:10619]			REACTOME_CYTOKINE_SIGNALING_IN_IMMUNE_SYSTEM		REACTOME_SIGNALING_BY_GPCR					KEGG_CYTOKINE_CYTOKINE_RECEPTOR_INTERACTION
6348	CCL3	C-C motif chemokine ligand 3 [Source:HGNC Symbol;Acc:HGNC:10627]			REACTOME_CYTOKINE_SIGNALING_IN_IMMUNE_SYSTEM		REACTOME_SIGNALING_BY_GPCR					KEGG_CYTOKINE_CYTOKINE_RECEPTOR_INTERACTION
6356	CCL11	C-C motif chemokine ligand 11 [Source:HGNC Symbol;Acc:HGNC:10610]			REACTOME_CYTOKINE_SIGNALING_IN_IMMUNE_SYSTEM		REACTOME_SIGNALING_BY_GPCR					KEGG_CYTOKINE_CYTOKINE_RECEPTOR_INTERACTION
6347	CCL2	C-C motif chemokine ligand 2 [Source:HGNC Symbol;Acc:HGNC:10618]			REACTOME_CYTOKINE_SIGNALING_IN_IMMUNE_SYSTEM		REACTOME_SIGNALING_BY_GPCR					KEGG_CYTOKINE_CYTOKINE_RECEPTOR_INTERACTION
414062	CCL3L3	C-C motif chemokine ligand 3 like 3 [Source:HGNC Symbol;Acc:HGNC:30554]			REACTOME_CYTOKINE_SIGNALING_IN_IMMUNE_SYSTEM		REACTOME_SIGNALING_BY_GPCR					KEGG_CYTOKINE_CYTOKINE_RECEPTOR_INTERACTION
333932	H3C15	H3 clustered histone 15 [Source:HGNC Symbol;Acc:HGNC:20505]			REACTOME_CYTOKINE_SIGNALING_IN_IMMUNE_SYSTEM			REACTOME_RNA_POLYMERASE_II_TRANSCRIPTION	REACTOME_HEMOSTASIS		KEGG_SYSTEMIC_LUPUS_ERYTHEMATOSUS	
356	FASLG	Fas ligand [Source:HGNC Symbol;Acc:HGNC:11936]			REACTOME_CYTOKINE_SIGNALING_IN_IMMUNE_SYSTEM			REACTOME_RNA_POLYMERASE_II_TRANSCRIPTION				KEGG_CYTOKINE_CYTOKINE_RECEPTOR_INTERACTION
3977	LIFR	LIF receptor subunit alpha [Source:HGNC Symbol;Acc:HGNC:6597]			REACTOME_CYTOKINE_SIGNALING_IN_IMMUNE_SYSTEM			REACTOME_RNA_POLYMERASE_II_TRANSCRIPTION				KEGG_CYTOKINE_CYTOKINE_RECEPTOR_INTERACTION
3726	JUNB	JunB proto-oncogene, AP-1 transcription factor subunit [Source:HGNC Symbol;Acc:HGNC:6205]			REACTOME_CYTOKINE_SIGNALING_IN_IMMUNE_SYSTEM			REACTOME_RNA_POLYMERASE_II_TRANSCRIPTION				
6648	SOD2	superoxide dismutase 2 [Source:HGNC Symbol;Acc:HGNC:11180]			REACTOME_CYTOKINE_SIGNALING_IN_IMMUNE_SYSTEM			REACTOME_RNA_POLYMERASE_II_TRANSCRIPTION				
6097	RORC	RAR related orphan receptor C [Source:HGNC Symbol;Acc:HGNC:10260]			REACTOME_CYTOKINE_SIGNALING_IN_IMMUNE_SYSTEM			REACTOME_RNA_POLYMERASE_II_TRANSCRIPTION				
2162	F13A1	coagulation factor XIII A chain [Source:HGNC Symbol;Acc:HGNC:3531]			REACTOME_CYTOKINE_SIGNALING_IN_IMMUNE_SYSTEM				REACTOME_HEMOSTASIS			
959	CD40LG	CD40 ligand [Source:HGNC Symbol;Acc:HGNC:11935]			REACTOME_CYTOKINE_SIGNALING_IN_IMMUNE_SYSTEM					KEGG_CELL_ADHESION_MOLECULES_CAMS	KEGG_SYSTEMIC_LUPUS_ERYTHEMATOSUS	KEGG_CYTOKINE_CYTOKINE_RECEPTOR_INTERACTION
3122	HLA-DRA	major histocompatibility complex, class II, DR alpha [Source:HGNC Symbol;Acc:HGNC:4947]			REACTOME_CYTOKINE_SIGNALING_IN_IMMUNE_SYSTEM					KEGG_CELL_ADHESION_MOLECULES_CAMS	KEGG_SYSTEMIC_LUPUS_ERYTHEMATOSUS	
3123	HLA-DRB1	major histocompatibility complex, class II, DR beta 1 [Source:HGNC Symbol;Acc:HGNC:4948]			REACTOME_CYTOKINE_SIGNALING_IN_IMMUNE_SYSTEM					KEGG_CELL_ADHESION_MOLECULES_CAMS	KEGG_SYSTEMIC_LUPUS_ERYTHEMATOSUS	
3127	HLA-DRB5	major histocompatibility complex, class II, DR beta 5 [Source:HGNC Symbol;Acc:HGNC:4953]			REACTOME_CYTOKINE_SIGNALING_IN_IMMUNE_SYSTEM					KEGG_CELL_ADHESION_MOLECULES_CAMS	KEGG_SYSTEMIC_LUPUS_ERYTHEMATOSUS	

3126	HLA-DRB4	major histocompatibility complex, class II, DR beta 4 [Source:HGNC Symbol;Acc:HGNC:4952]			REACTOME_CYTOKINE_SIGNALING_IN_IMMUNE_SYSTEM					KEGG_CELL_ADHESION_MOLECULES_CAMS	KEGG_SYSTEMIC_LUPUS_ERYTHEMATOSUS	
3117	HLA-DQA1	major histocompatibility complex, class II, DQ alpha 1 [Source:HGNC Symbol;Acc:HGNC:4942]			REACTOME_CYTOKINE_SIGNALING_IN_IMMUNE_SYSTEM					KEGG_CELL_ADHESION_MOLECULES_CAMS	KEGG_SYSTEMIC_LUPUS_ERYTHEMATOSUS	
3113	HLA-DPA1	major histocompatibility complex, class II, DP alpha 1 [Source:HGNC Symbol;Acc:HGNC:4938]			REACTOME_CYTOKINE_SIGNALING_IN_IMMUNE_SYSTEM					KEGG_CELL_ADHESION_MOLECULES_CAMS	KEGG_SYSTEMIC_LUPUS_ERYTHEMATOSUS	
3115	HLA-DPB1	major histocompatibility complex, class II, DP beta 1 [Source:HGNC Symbol;Acc:HGNC:4940]			REACTOME_CYTOKINE_SIGNALING_IN_IMMUNE_SYSTEM					KEGG_CELL_ADHESION_MOLECULES_CAMS	KEGG_SYSTEMIC_LUPUS_ERYTHEMATOSUS	
4684	NCAM1	neural cell adhesion molecule 1 [Source:HGNC Symbol;Acc:HGNC:7656]			REACTOME_CYTOKINE_SIGNALING_IN_IMMUNE_SYSTEM					KEGG_CELL_ADHESION_MOLECULES_CAMS		
3552	IL1A	interleukin 1 alpha [Source:HGNC Symbol;Acc:HGNC:5991]			REACTOME_CYTOKINE_SIGNALING_IN_IMMUNE_SYSTEM							KEGG_CYTOKINE_CYTOKINE_RECEPTOR_INTERACTION
3565	IL4	interleukin 4 [Source:HGNC Symbol;Acc:HGNC:6014]			REACTOME_CYTOKINE_SIGNALING_IN_IMMUNE_SYSTEM							KEGG_CYTOKINE_CYTOKINE_RECEPTOR_INTERACTION
5008	OSM	oncostatin M [Source:HGNC Symbol;Acc:HGNC:8506]			REACTOME_CYTOKINE_SIGNALING_IN_IMMUNE_SYSTEM							KEGG_CYTOKINE_CYTOKINE_RECEPTOR_INTERACTION
2323	FLT3LG	fms related receptor tyrosine kinase 3 ligand [Source:HGNC Symbol;Acc:HGNC:3766]			REACTOME_CYTOKINE_SIGNALING_IN_IMMUNE_SYSTEM							KEGG_CYTOKINE_CYTOKINE_RECEPTOR_INTERACTION
4049	LTA	lymphotoxin alpha [Source:HGNC Symbol;Acc:HGNC:6709]			REACTOME_CYTOKINE_SIGNALING_IN_IMMUNE_SYSTEM							KEGG_CYTOKINE_CYTOKINE_RECEPTOR_INTERACTION
5617	PRL	prolactin [Source:HGNC Symbol;Acc:HGNC:9445]			REACTOME_CYTOKINE_SIGNALING_IN_IMMUNE_SYSTEM							KEGG_CYTOKINE_CYTOKINE_RECEPTOR_INTERACTION
8740	TNFSF14	TNF superfamily member 14 [Source:HGNC Symbol;Acc:HGNC:11930]			REACTOME_CYTOKINE_SIGNALING_IN_IMMUNE_SYSTEM							KEGG_CYTOKINE_CYTOKINE_RECEPTOR_INTERACTION
3554	IL1R1	interleukin 1 receptor type 1 [Source:HGNC Symbol;Acc:HGNC:5993]			REACTOME_CYTOKINE_SIGNALING_IN_IMMUNE_SYSTEM							KEGG_CYTOKINE_CYTOKINE_RECEPTOR_INTERACTION
3568	IL5RA	interleukin 5 receptor subunit alpha [Source:HGNC Symbol;Acc:HGNC:6017]			REACTOME_CYTOKINE_SIGNALING_IN_IMMUNE_SYSTEM							KEGG_CYTOKINE_CYTOKINE_RECEPTOR_INTERACTION
3575	IL7R	interleukin 7 receptor [Source:HGNC Symbol;Acc:HGNC:6024]			REACTOME_CYTOKINE_SIGNALING_IN_IMMUNE_SYSTEM							KEGG_CYTOKINE_CYTOKINE_RECEPTOR_INTERACTION
7850	IL1R2	interleukin 1 receptor type 2 [Source:HGNC Symbol;Acc:HGNC:5994]			REACTOME_CYTOKINE_SIGNALING_IN_IMMUNE_SYSTEM							KEGG_CYTOKINE_CYTOKINE_RECEPTOR_INTERACTION
1438	CSF2RA	colony stimulating factor 2 receptor subunit alpha [Source:HGNC Symbol;Acc:HGNC:2435]			REACTOME_CYTOKINE_SIGNALING_IN_IMMUNE_SYSTEM							KEGG_CYTOKINE_CYTOKINE_RECEPTOR_INTERACTION
8809	IL18R1	interleukin 18 receptor 1 [Source:HGNC Symbol;Acc:HGNC:5988]			REACTOME_CYTOKINE_SIGNALING_IN_IMMUNE_SYSTEM							KEGG_CYTOKINE_CYTOKINE_RECEPTOR_INTERACTION
8807	IL18RAP	interleukin 18 receptor accessory protein [Source:HGNC Symbol;Acc:HGNC:5989]			REACTOME_CYTOKINE_SIGNALING_IN_IMMUNE_SYSTEM							KEGG_CYTOKINE_CYTOKINE_RECEPTOR_INTERACTION
3560	IL2RB	interleukin 2 receptor subunit beta [Source:HGNC Symbol;Acc:HGNC:6009]			REACTOME_CYTOKINE_SIGNALING_IN_IMMUNE_SYSTEM							KEGG_CYTOKINE_CYTOKINE_RECEPTOR_INTERACTION
3595	IL12RB2	interleukin 12 receptor subunit beta 2 [Source:HGNC Symbol;Acc:HGNC:5972]			REACTOME_CYTOKINE_SIGNALING_IN_IMMUNE_SYSTEM							KEGG_CYTOKINE_CYTOKINE_RECEPTOR_INTERACTION
53833	IL20RB	interleukin 20 receptor subunit beta [Source:HGNC Symbol;Acc:HGNC:6004]			REACTOME_CYTOKINE_SIGNALING_IN_IMMUNE_SYSTEM							KEGG_CYTOKINE_CYTOKINE_RECEPTOR_INTERACTION
2322	FLT3	fms related receptor tyrosine kinase 3 [Source:HGNC Symbol;Acc:HGNC:3765]			REACTOME_CYTOKINE_SIGNALING_IN_IMMUNE_SYSTEM							KEGG_CYTOKINE_CYTOKINE_RECEPTOR_INTERACTION
8718	TNFRSF25	TNF receptor superfamily member 25 [Source:HGNC Symbol;Acc:HGNC:11910]			REACTOME_CYTOKINE_SIGNALING_IN_IMMUNE_SYSTEM							KEGG_CYTOKINE_CYTOKINE_RECEPTOR_INTERACTION
7293	TNFRSF4	TNF receptor superfamily member 4 [Source:HGNC Symbol;Acc:HGNC:11918]			REACTOME_CYTOKINE_SIGNALING_IN_IMMUNE_SYSTEM							KEGG_CYTOKINE_CYTOKINE_RECEPTOR_INTERACTION
939	CD27	CD27 molecule [Source:HGNC Symbol;Acc:HGNC:11922]			REACTOME_CYTOKINE_SIGNALING_IN_IMMUNE_SYSTEM							KEGG_CYTOKINE_CYTOKINE_RECEPTOR_INTERACTION
10148	EBI3	Epstein-Barr virus induced 3 [Source:HGNC Symbol;Acc:HGNC:3129]			REACTOME_CYTOKINE_SIGNALING_IN_IMMUNE_SYSTEM							
6773	STAT2	signal transducer and activator of transcription 2 [Source:HGNC Symbol;Acc:HGNC:11363]			REACTOME_CYTOKINE_SIGNALING_IN_IMMUNE_SYSTEM							
1052	CEBPD	CCAAT enhancer binding protein delta [Source:HGNC Symbol;Acc:HGNC:1835]			REACTOME_CYTOKINE_SIGNALING_IN_IMMUNE_SYSTEM							
5783	PTPN13	protein tyrosine phosphatase non-receptor type 13 [Source:HGNC Symbol;Acc:HGNC:9646]			REACTOME_CYTOKINE_SIGNALING_IN_IMMUNE_SYSTEM							
4128	MAOA	monoamine oxidase A [Source:HGNC Symbol;Acc:HGNC:6833]			REACTOME_CYTOKINE_SIGNALING_IN_IMMUNE_SYSTEM							
10538	BATF	basic leucine zipper ATF-like transcription factor [Source:HGNC Symbol;Acc:HGNC:958]			REACTOME_CYTOKINE_SIGNALING_IN_IMMUNE_SYSTEM							
3667	IRS1	insulin receptor substrate 1 [Source:HGNC Symbol;Acc:HGNC:6125]			REACTOME_CYTOKINE_SIGNALING_IN_IMMUNE_SYSTEM							
9173	IL1RL1	interleukin 1 receptor like 1 [Source:HGNC Symbol;Acc:HGNC:5998]			REACTOME_CYTOKINE_SIGNALING_IN_IMMUNE_SYSTEM							
4507	MTAP	methylthioadenosine phosphorylase [Source:HGNC Symbol;Acc:HGNC:7413]			REACTOME_CYTOKINE_SIGNALING_IN_IMMUNE_SYSTEM							
5610	EIF2AK2	eukaryotic translation initiation factor 2 alpha kinase 2 [Source:HGNC Symbol;Acc:HGNC:9437]			REACTOME_CYTOKINE_SIGNALING_IN_IMMUNE_SYSTEM							
3841	KPNA5	karyopherin subunit alpha 5 [Source:HGNC Symbol;Acc:HGNC:6398]			REACTOME_CYTOKINE_SIGNALING_IN_IMMUNE_SYSTEM							
2635	GBP3	guanylate binding protein 3 [Source:HGNC Symbol;Acc:HGNC:4184]			REACTOME_CYTOKINE_SIGNALING_IN_IMMUNE_SYSTEM							
4261	CIITA	class II major histocompatibility complex transactivator [Source:HGNC Symbol;Acc:HGNC:7067]			REACTOME_CYTOKINE_SIGNALING_IN_IMMUNE_SYSTEM							
4938	OAS1	2'-5'-oligoadenylate synthetase 1 [Source:HGNC Symbol;Acc:HGNC:8086]			REACTOME_CYTOKINE_SIGNALING_IN_IMMUNE_SYSTEM							
4939	OAS2	2'-5'-oligoadenylate synthetase 2 [Source:HGNC Symbol;Acc:HGNC:8087]			REACTOME_CYTOKINE_SIGNALING_IN_IMMUNE_SYSTEM							
4940	OAS3	2'-5'-oligoadenylate synthetase 3 [Source:HGNC Symbol;Acc:HGNC:8088]			REACTOME_CYTOKINE_SIGNALING_IN_IMMUNE_SYSTEM							
3433	IFIT2	interferon induced protein with tetratricopeptide repeats 2 [Source:HGNC Symbol;Acc:HGNC:5409]			REACTOME_CYTOKINE_SIGNALING_IN_IMMUNE_SYSTEM							
4599	MX1	MX dynamin like GTPase 1 [Source:HGNC Symbol;Acc:HGNC:7532]			REACTOME_CYTOKINE_SIGNALING_IN_IMMUNE_SYSTEM							
25939	SAMHD1	SAM and HD domain containing deoxynucleoside triphosphate triphosphohydrolase 1 [Source:HGNC Symbol;Acc:HGNC:15925]			REACTOME_CYTOKINE_SIGNALING_IN_IMMUNE_SYSTEM							
2537	IFI6	interferon alpha inducible protein 6 [Source:HGNC Symbol;Acc:HGNC:4054]			REACTOME_CYTOKINE_SIGNALING_IN_IMMUNE_SYSTEM							
3434	IFIT1	interferon induced protein with tetratricopeptide repeats 1 [Source:HGNC Symbol;Acc:HGNC:5407]			REACTOME_CYTOKINE_SIGNALING_IN_IMMUNE_SYSTEM							
91543	RSAD2	radical S-adenosyl methionine domain containing 2 [Source:HGNC Symbol;Acc:HGNC:30908]			REACTOME_CYTOKINE_SIGNALING_IN_IMMUNE_SYSTEM							
3429	IFI27	interferon alpha inducible protein 27 [Source:HGNC Symbol;Acc:HGNC:5397]			REACTOME_CYTOKINE_SIGNALING_IN_IMMUNE_SYSTEM							
54739	XAF1	XIAP associated factor 1 [Source:HGNC Symbol;Acc:HGNC:30932]			REACTOME_CYTOKINE_SIGNALING_IN_IMMUNE_SYSTEM							
24138	IFIT5	interferon induced protein with tetratricopeptide repeats 5 [Source:HGNC Symbol;Acc:HGNC:13328]			REACTOME_CYTOKINE_SIGNALING_IN_IMMUNE_SYSTEM							
2633	GBP1	guanylate binding protein 1 [Source:HGNC Symbol;Acc:HGNC:4182]			REACTOME_CYTOKINE_SIGNALING_IN_IMMUNE_SYSTEM							
115361	GBP4	guanylate binding protein 4 [Source:HGNC Symbol;Acc:HGNC:20480]			REACTOME_CYTOKINE_SIGNALING_IN_IMMUNE_SYSTEM							
115362	GBP5	guanylate binding protein 5 [Source:HGNC Symbol;Acc:HGNC:19895]			REACTOME_CYTOKINE_SIGNALING_IN_IMMUNE_SYSTEM							
55128	TRIM68	tripartite motif containing 68 [Source:HGNC Symbol;Acc:HGNC:21161]			REACTOME_CYTOKINE_SIGNALING_IN_IMMUNE_SYSTEM							
9830	TRIM14	tripartite motif containing 14 [Source:HGNC Symbol;Acc:HGNC:16283]			REACTOME_CYTOKINE_SIGNALING_IN_IMMUNE_SYSTEM							
2887	GRB10	growth factor receptor bound protein 10 [Source:HGNC Symbol;Acc:HGNC:4564]			REACTOME_CYTOKINE_SIGNALING_IN_IMMUNE_SYSTEM							
128178	EDARADD	EDAR associated death domain [Source:HGNC Symbol;Acc:HGNC:14341]			REACTOME_CYTOKINE_SIGNALING_IN_IMMUNE_SYSTEM							
8345	H2BC9	H2B clustered histone 9 [Source:HGNC Symbol;Acc:HGNC:4755]				REACTOME_POST_TRANSLATIONAL_PROTEIN_MODIFICATION		REACTOME_RNA_POLYMERASE_II_TRANSCRIPTION			KEGG_SYSTEMIC_LUPUS_ERYTHEMATOSUS	
8335	H2AC4	H2A clustered histone 4 [Source:HGNC Symbol;Acc:HGNC:4734]				REACTOME_POST_TRANSLATIONAL_PROTEIN_MODIFICATION		REACTOME_RNA_POLYMERASE_II_TRANSCRIPTION			KEGG_SYSTEMIC_LUPUS_ERYTHEMATOSUS	
3013	H2AC7	H2A clustered histone 7 [Source:HGNC Symbol;Acc:HGNC:4729]				REACTOME_POST_TRANSLATIONAL_PROTEIN_MODIFICATION		REACTOME_RNA_POLYMERASE_II_TRANSCRIPTION			KEGG_SYSTEMIC_LUPUS_ERYTHEMATOSUS	
723790	H2AC19	H2A clustered histone 19 [Source:HGNC Symbol;Acc:HGNC:29668]				REACTOME_POST_TRANSLATIONAL_PROTEIN_MODIFICATION		REACTOME_RNA_POLYMERASE_II_TRANSCRIPTION			KEGG_SYSTEMIC_LUPUS_ERYTHEMATOSUS	
8338	H2AC20	H2A clustered histone 20 [Source:HGNC Symbol;Acc:HGNC:4738]				REACTOME_POST_TRANSLATIONAL_PROTEIN_MODIFICATION		REACTOME_RNA_POLYMERASE_II_TRANSCRIPTION			KEGG_SYSTEMIC_LUPUS_ERYTHEMATOSUS	
3018	H2BC3	H2B clustered histone 3 [Source:HGNC Symbol;Acc:HGNC:4751]				REACTOME_POST_TRANSLATIONAL_PROTEIN_MODIFICATION		REACTOME_RNA_POLYMERASE_II_TRANSCRIPTION			KEGG_SYSTEMIC_LUPUS_ERYTHEMATOSUS	
8339	H2BC8	H2B clustered histone 8 [Source:HGNC Symbol;Acc:HGNC:4746]				REACTOME_POST_TRANSLATIONAL_PROTEIN_MODIFICATION		REACTOME_RNA_POLYMERASE_II_TRANSCRIPTION			KEGG_SYSTEMIC_LUPUS_ERYTHEMATOSUS	
3017	H2BC5	H2B clustered histone 5 [Source:HGNC Symbol;Acc:HGNC:4747]				REACTOME_POST_TRANSLATIONAL_PROTEIN_MODIFICATION		REACTOME_RNA_POLYMERASE_II_TRANSCRIPTION			KEGG_SYSTEMIC_LUPUS_ERYTHEMATOSUS	
8340	H2BC13	H2B clustered histone 13 [Source:HGNC Symbol;Acc:HGNC:4748]				REACTOME_POST_TRANSLATIONAL_PROTEIN_MODIFICATION		REACTOME_RNA_POLYMERASE_II_TRANSCRIPTION			KEGG_SYSTEMIC_LUPUS_ERYTHEMATOSUS	
8343	H2BC7	H2B clustered histone 7 [Source:HGNC Symbol;Acc:HGNC:4752]				REACTOME_POST_TRANSLATIONAL_PROTEIN_MODIFICATION		REACTOME_RNA_POLYMERASE_II_TRANSCRIPTION			KEGG_SYSTEMIC_LUPUS_ERYTHEMATOSUS	
8348	H2BC17	H2B clustered histone 17 [Source:HGNC Symbol;Acc:HGNC:4758]				REACTOME_POST_TRANSLATIONAL_PROTEIN_MODIFICATION		REACTOME_RNA_POLYMERASE_II_TRANSCRIPTION			KEGG_SYSTEMIC_LUPUS_ERYTHEMATOSUS	
8970	H2BC11	H2B clustered histone 11 [Source:HGNC Symbol;Acc:HGNC:4761]				REACTOME_POST_TRANSLATIONAL_PROTEIN_MODIFICATION		REACTOME_RNA_POLYMERASE_II_TRANSCRIPTION			KEGG_SYSTEMIC_LUPUS_ERYTHEMATOSUS	
8359	H4C1	H4 clustered histone 1 [Source:HGNC Symbol;Acc:HGNC:4781]				REACTOME_POST_TRANSLATIONAL_PROTEIN_MODIFICATION		REACTOME_RNA_POLYMERASE_II_TRANSCRIPTION			KEGG_SYSTEMIC_LUPUS_ERYTHEMATOSUS	
51616	TAF9B	TATA-box binding protein associated factor 9b [Source:HGNC Symbol;Acc:HGNC:17306]				REACTOME_POST_TRANSLATIONAL_PROTEIN_MODIFICATION		REACTOME_RNA_POLYMERASE_II_TRANSCRIPTION				
5468	PPARG	peroxisome proliferator activated receptor gamma [Source:HGNC Symbol;Acc:HGNC:9236]				REACTOME_POST_TRANSLATIONAL_PROTEIN_MODIFICATION		REACTOME_RNA_POLYMERASE_II_TRANSCRIPTION				
5465	PPARA	peroxisome proliferator activated receptor alpha [Source:HGNC Symbol;Acc:HGNC:9232]				REACTOME_POST_TRANSLATIONAL_PROTEIN_MODIFICATION		REACTOME_RNA_POLYMERASE_II_TRANSCRIPTION				
4193	MDM2	MDM2 proto-oncogene [Source:HGNC Symbol;Acc:HGNC:6973]				REACTOME_POST_TRANSLATIONAL_PROTEIN_MODIFICATION		REACTOME_RNA_POLYMERASE_II_TRANSCRIPTION				
4086	SMAD1	SMAD family member 1 [Source:HGNC Symbol;Acc:HGNC:6767]				REACTOME_POST_TRANSLATIONAL_PROTEIN_MODIFICATION		REACTOME_RNA_POLYMERASE_II_TRANSCRIPTION				
2516	NR5A1	nuclear receptor subfamily 5 group A member 1 [Source:HGNC Symbol;Acc:HGNC:7983]				REACTOME_POST_TRANSLATIONAL_PROTEIN_MODIFICATION		REACTOME_RNA_POLYMERASE_II_TRANSCRIPTION				
1912	PHC2	polyhomeotic homolog 2 [Source:HGNC Symbol;Acc:HGNC:3183]				REACTOME_POST_TRANSLATIONAL_PROTEIN_MODIFICATION		REACTOME_RNA_POLYMERASE_II_TRANSCRIPTION				
7020	TFAP2A	transcription factor AP-2 alpha [Source:HGNC Symbol;Acc:HGNC:11742]				REACTOME_POST_TRANSLATIONAL_PROTEIN_MODIFICATION		REACTOME_RNA_POLYMERASE_II_TRANSCRIPTION				
4306	NR3C2	nuclear receptor subfamily 3 group C member 2 [Source:HGNC Symbol;Acc:HGNC:7979]				REACTOME_POST_TRANSLATIONAL_PROTEIN_MODIFICATION		REACTOME_RNA_POLYMERASE_II_TRANSCRIPTION				
7181	NR2C1	nuclear receptor subfamily 2 group C member 1 [Source:HGNC Symbol;Acc:HGNC:7971]				REACTOME_POST_TRANSLATIONAL_PROTEIN_MODIFICATION		REACTOME_RNA_POLYMERASE_II_TRANSCRIPTION				
10381	TUBB3	tubulin beta 3 class III [Source:HGNC Symbol;Acc:HGNC:20772]				REACTOME_POST_TRANSLATIONAL_PROTEIN_MODIFICATION			REACTOME_HEMOSTASIS			
7280	TUBB2A	tubulin beta 2A class IIa [Source:HGNC Symbol;Acc:HGNC:12412]				REACTOME_POST_TRANSLATIONAL_PROTEIN_MODIFICATION			REACTOME_HEMOSTASIS			
81027	TUBB1	tubulin beta 1 class VI [Source:HGNC Symbol;Acc:HGNC:16257]				REACTOME_POST_TRANSLATIONAL_PROTEIN_MODIFICATION			REACTOME_HEMOSTASIS			
832	CAPZB	capping actin protein of muscle Z-line subunit beta [Source:HGNC Symbol;Acc:HGNC:1491]				REACTOME_POST_TRANSLATIONAL_PROTEIN_MODIFICATION			REACTOME_HEMOSTASIS			
462	SERPINC1	serpin family C member 1 [Source:HGNC Symbol;Acc:HGNC:775]				REACTOME_POST_TRANSLATIONAL_PROTEIN_MODIFICATION			REACTOME_HEMOSTASIS			
5874	RAB27B	RAB27B, member RAS oncogene family [Source:HGNC Symbol;Acc:HGNC:9767]				REACTOME_POST_TRANSLATIONAL_PROTEIN_MODIFICATION			REACTOME_HEMOSTASIS			
1048	CEACAM5	CEA cell adhesion molecule 5 [Source:HGNC Symbol;Acc:HGNC:1817]				REACTOME_POST_TRANSLATIONAL_PROTEIN_MODIFICATION			REACTOME_HEMOSTASIS			
8332	H2AC16	H2A clustered histone 16 [Source:HGNC Symbol;Acc:HGNC:4730]				REACTOME_POST_TRANSLATIONAL_PROTEIN_MODIFICATION					KEGG_SYSTEMIC_LUPUS_ERYTHEMATOSUS	
317772	H2AC21	H2A clustered histone 21 [Source:HGNC Symbol;Acc:HGNC:20508]				REACTOME_POST_TRANSLATIONAL_PROTEIN_MODIFICATION					KEGG_SYSTEMIC_LUPUS_ERYTHEMATOSUS	
8330	H2AC15	H2A clustered histone 15 [Source:HGNC Symbol;Acc:HGNC:4726]				REACTOME_POST_TRANSLATIONAL_PROTEIN_MODIFICATION					KEGG_SYSTEMIC_LUPUS_ERYTHEMATOSUS	
8336	H2AC17	H2A clustered histone 17 [Source:HGNC Symbol;Acc:HGNC:4735]				REACTOME_POST_TRANSLATIONAL_PROTEIN_MODIFICATION					KEGG_SYSTEMIC_LUPUS_ERYTHEMATOSUS	
8969	H2AC11	H2A clustered histone 11 [Source:HGNC Symbol;Acc:HGNC:4737]				REACTOME_POST_TRANSLATIONAL_PROTEIN_MODIFICATION					KEGG_SYSTEMIC_LUPUS_ERYTHEMATOSUS	
51128	SAR1B	secretion associated Ras related GTPase 1B [Source:HGNC Symbol;Acc:HGNC:10535]				REACTOME_POST_TRANSLATIONAL_PROTEIN_MODIFICATION						
8453	CUL2	cullin 2 [Source:HGNC Symbol;Acc:HGNC:2552]				REACTOME_POST_TRANSLATIONAL_PROTEIN_MODIFICATION						
10540	DCTN2	dynactin subunit 2 [Source:HGNC Symbol;Acc:HGNC:2712]				REACTOME_POST_TRANSLATIONAL_PROTEIN_MODIFICATION						
92912	UBE2Q2	ubiquitin conjugating enzyme E2 Q2 [Source:HGNC Symbol;Acc:HGNC:19248]				REACTOME_POST_TRANSLATIONAL_PROTEIN_MODIFICATION						
26190	FBXW2	F-box and WD repeat domain containing 2 [Source:HGNC Symbol;Acc:HGNC:13608]				REACTOME_POST_TRANSLATIONAL_PROTEIN_MODIFICATION						
146330	FBXL16	F-box and leucine rich repeat protein 16 [Source:HGNC Symbol;Acc:HGNC:14150]				REACTOME_POST_TRANSLATIONAL_PROTEIN_MODIFICATION						
150726	FBXO41	F-box protein 41 [Source:HGNC Symbol;Acc:HGNC:29409]				REACTOME_POST_TRANSLATIONAL_PROTEIN_MODIFICATION						
23014	FBXO21	F-box protein 21 [Source:HGNC Symbol;Acc:HGNC:13592]				REACTOME_POST_TRANSLATIONAL_PROTEIN_MODIFICATION						
26249	KLHL3	kelch like family member 3 [Source:HGNC Symbol;Acc:HGNC:6354]				REACTOME_POST_TRANSLATIONAL_PROTEIN_MODIFICATION						
51676	ASB2	ankyrin repeat and SOCS box containing 2 [Source:HGNC Symbol;Acc:HGNC:16012]				REACTOME_POST_TRANSLATIONAL_PROTEIN_MODIFICATION						
11096	ADAMTS5	ADAM metallopeptidase with thrombospondin type 1 motif 5 [Source:HGNC Symbol;Acc:HGNC:221]				REACTOME_POST_TRANSLATIONAL_PROTEIN_MODIFICATION						
9508	ADAMTS3	ADAM metallopeptidase with thrombospondin type 1 motif 3 [Source:HGNC Symbol;Acc:HGNC:219]				REACTOME_POST_TRANSLATIONAL_PROTEIN_MODIFICATION						
81794	ADAMTS10	ADAM metallopeptidase with thrombospondin type 1 motif 10 [Source:HGNC Symbol;Acc:HGNC:13201]				REACTOME_POST_TRANSLATIONAL_PROTEIN_MODIFICATION						
170689	ADAMTS15	ADAM metallopeptidase with thrombospondin type 1 motif 15 [Source:HGNC Symbol;Acc:HGNC:16305]				REACTOME_POST_TRANSLATIONAL_PROTEIN_MODIFICATION						
54757	FAM20A	FAM20A golgi associated secretory pathway pseudokinase [Source:HGNC Symbol;Acc:HGNC:23015]				REACTOME_POST_TRANSLATIONAL_PROTEIN_MODIFICATION						
10418	SPON1	spondin 1 [Source:HGNC Symbol;Acc:HGNC:11252]				REACTOME_POST_TRANSLATIONAL_PROTEIN_MODIFICATION						
3490	IGFBP7	insulin like growth factor binding protein 7 [Source:HGNC Symbol;Acc:HGNC:5476]				REACTOME_POST_TRANSLATIONAL_PROTEIN_MODIFICATION						
4052	LTBP1	latent transforming growth factor beta binding protein 1 [Source:HGNC Symbol;Acc:HGNC:6714]				REACTOME_POST_TRANSLATIONAL_PROTEIN_MODIFICATION						
10417	SPON2	spondin 2 [Source:HGNC Symbol;Acc:HGNC:11253]				REACTOME_POST_TRANSLATIONAL_PROTEIN_MODIFICATION						
6484	ST3GAL4	ST3 beta-galactoside alpha-2,3-sialyltransferase 4 [Source:HGNC Symbol;Acc:HGNC:10864]				REACTOME_POST_TRANSLATIONAL_PROTEIN_MODIFICATION						
256435	ST6GALNAC3	ST6 N-acetylgalactosaminide alpha-2,6-sialyltransferase 3 [Source:HGNC Symbol;Acc:HGNC:19343]				REACTOME_POST_TRANSLATIONAL_PROTEIN_MODIFICATION						
6480	ST6GAL1	ST6 beta-galactoside alpha-2,6-sialyltransferase 1 [Source:HGNC Symbol;Acc:HGNC:10860]				REACTOME_POST_TRANSLATIONAL_PROTEIN_MODIFICATION						
10610	ST6GALNAC2	ST6 N-acetylgalactosaminide alpha-2,6-sialyltransferase 2 [Source:HGNC Symbol;Acc:HGNC:10867]				REACTOME_POST_TRANSLATIONAL_PROTEIN_MODIFICATION						
5987	TRIM27	tripartite motif containing 27 [Source:HGNC Symbol;Acc:HGNC:9975]				REACTOME_POST_TRANSLATIONAL_PROTEIN_MODIFICATION						
336	APOA2	apolipoprotein A2 [Source:HGNC Symbol;Acc:HGNC:601]				REACTOME_POST_TRANSLATIONAL_PROTEIN_MODIFICATION						
285362	SUMF1	sulfatase modifying factor 1 [Source:HGNC Symbol;Acc:HGNC:20376]				REACTOME_POST_TRANSLATIONAL_PROTEIN_MODIFICATION						
51299	NRN1	neuritin 1 [Source:HGNC Symbol;Acc:HGNC:17972]				REACTOME_POST_TRANSLATIONAL_PROTEIN_MODIFICATION						
9100	USP10	ubiquitin specific peptidase 10 [Source:HGNC Symbol;Acc:HGNC:12608]				REACTOME_POST_TRANSLATIONAL_PROTEIN_MODIFICATION						
81847	RNF146	ring finger protein 146 [Source:HGNC Symbol;Acc:HGNC:21336]				REACTOME_POST_TRANSLATIONAL_PROTEIN_MODIFICATION						
8724	SNX3	sorting nexin 3 [Source:HGNC Symbol;Acc:HGNC:11174]				REACTOME_POST_TRANSLATIONAL_PROTEIN_MODIFICATION						
219333	USP12	ubiquitin specific peptidase 12 [Source:HGNC Symbol;Acc:HGNC:20485]				REACTOME_POST_TRANSLATIONAL_PROTEIN_MODIFICATION						
29761	USP25	ubiquitin specific peptidase 25 [Source:HGNC Symbol;Acc:HGNC:12624]				REACTOME_POST_TRANSLATIONAL_PROTEIN_MODIFICATION						
57646	USP28	ubiquitin specific peptidase 28 [Source:HGNC Symbol;Acc:HGNC:12625]				REACTOME_POST_TRANSLATIONAL_PROTEIN_MODIFICATION						
9097	USP14	ubiquitin specific peptidase 14 [Source:HGNC Symbol;Acc:HGNC:12612]				REACTOME_POST_TRANSLATIONAL_PROTEIN_MODIFICATION						
9577	BABAM2	BRISC and BRCA1 A complex member 2 [Source:HGNC Symbol;Acc:HGNC:1106]				REACTOME_POST_TRANSLATIONAL_PROTEIN_MODIFICATION						
79913	ACTR5	actin related protein 5 [Source:HGNC Symbol;Acc:HGNC:14671]				REACTOME_POST_TRANSLATIONAL_PROTEIN_MODIFICATION						
10318	TNIP1	TNFAIP3 interacting protein 1 [Source:HGNC Symbol;Acc:HGNC:16903]				REACTOME_POST_TRANSLATIONAL_PROTEIN_MODIFICATION						
126119	JOSD2	Josephin domain containing 2 [Source:HGNC Symbol;Acc:HGNC:28853]				REACTOME_POST_TRANSLATIONAL_PROTEIN_MODIFICATION						
9063	PIAS2	protein inhibitor of activated STAT 2 [Source:HGNC Symbol;Acc:HGNC:17311]				REACTOME_POST_TRANSLATIONAL_PROTEIN_MODIFICATION						
288	ANK3	ankyrin 3 [Source:HGNC Symbol;Acc:HGNC:494]				REACTOME_POST_TRANSLATIONAL_PROTEIN_MODIFICATION						
286	ANK1	ankyrin 1 [Source:HGNC Symbol;Acc:HGNC:492]				REACTOME_POST_TRANSLATIONAL_PROTEIN_MODIFICATION						
11041	B4GAT1	beta-1,4-glucuronyltransferase 1 [Source:HGNC Symbol;Acc:HGNC:15685]				REACTOME_POST_TRANSLATIONAL_PROTEIN_MODIFICATION						
2590	GALNT2	polypeptide N-acetylgalactosaminyltransferase 2 [Source:HGNC Symbol;Acc:HGNC:4124]				REACTOME_POST_TRANSLATIONAL_PROTEIN_MODIFICATION						
63917	GALNT11	polypeptide N-acetylgalactosaminyltransferase 11 [Source:HGNC Symbol;Acc:HGNC:19875]				REACTOME_POST_TRANSLATIONAL_PROTEIN_MODIFICATION						
11226	GALNT6	polypeptide N-acetylgalactosaminyltransferase 6 [Source:HGNC Symbol;Acc:HGNC:4128]				REACTOME_POST_TRANSLATIONAL_PROTEIN_MODIFICATION						
192134	B3GNT6	UDP-GlcNAc:betaGal beta-1,3-N-acetylglucosaminyltransferase 6 [Source:HGNC Symbol;Acc:HGNC:24141]				REACTOME_POST_TRANSLATIONAL_PROTEIN_MODIFICATION						
79623	GALNT14	polypeptide N-acetylgalactosaminyltransferase 14 [Source:HGNC Symbol;Acc:HGNC:22946]				REACTOME_POST_TRANSLATIONAL_PROTEIN_MODIFICATION						
146712	B3GNTL1	UDP-GlcNAc:betaGal beta-1,3-N-acetylglucosaminyltransferase like 1 [Source:HGNC Symbol;Acc:HGNC:21727]				REACTOME_POST_TRANSLATIONAL_PROTEIN_MODIFICATION						
374907	B3GNT8	UDP-GlcNAc:betaGal beta-1,3-N-acetylglucosaminyltransferase 8 [Source:HGNC Symbol;Acc:HGNC:24139]				REACTOME_POST_TRANSLATIONAL_PROTEIN_MODIFICATION						
55901	THSD1	thrombospondin type 1 domain containing 1 [Source:HGNC Symbol;Acc:HGNC:17754]				REACTOME_POST_TRANSLATIONAL_PROTEIN_MODIFICATION						
80731	THSD7B	thrombospondin type 1 domain containing 7B [Source:HGNC Symbol;Acc:HGNC:29348]				REACTOME_POST_TRANSLATIONAL_PROTEIN_MODIFICATION						
57719	ANO8	anoctamin 8 [Source:HGNC Symbol;Acc:HGNC:29329]				REACTOME_POST_TRANSLATIONAL_PROTEIN_MODIFICATION						
255738	PCSK9	proprotein convertase subtilisin/kexin type 9 [Source:HGNC Symbol;Acc:HGNC:20001]				REACTOME_POST_TRANSLATIONAL_PROTEIN_MODIFICATION						
1786	DNMT1	DNA methyltransferase 1 [Source:HGNC Symbol;Acc:HGNC:2976]				REACTOME_POST_TRANSLATIONAL_PROTEIN_MODIFICATION						
30815	ST6GALNAC6	ST6 N-acetylgalactosaminide alpha-2,6-sialyltransferase 6 [Source:HGNC Symbol;Acc:HGNC:23364]				REACTOME_POST_TRANSLATIONAL_PROTEIN_MODIFICATION						
4061	LY6E	lymphocyte antigen 6 family member E [Source:HGNC Symbol;Acc:HGNC:6727]				REACTOME_POST_TRANSLATIONAL_PROTEIN_MODIFICATION						
249	ALPL	alkaline phosphatase, biomineralization associated [Source:HGNC Symbol;Acc:HGNC:438]				REACTOME_POST_TRANSLATIONAL_PROTEIN_MODIFICATION						
29906	ST8SIA5	ST8 alpha-N-acetyl-neuraminide alpha-2,8-sialyltransferase 5 [Source:HGNC Symbol;Acc:HGNC:17827]				REACTOME_POST_TRANSLATIONAL_PROTEIN_MODIFICATION						
8869	ST3GAL5	ST3 beta-galactoside alpha-2,3-sialyltransferase 5 [Source:HGNC Symbol;Acc:HGNC:10872]				REACTOME_POST_TRANSLATIONAL_PROTEIN_MODIFICATION						
140838	NANP	N-acetylneuraminic acid phosphatase [Source:HGNC Symbol;Acc:HGNC:16140]				REACTOME_POST_TRANSLATIONAL_PROTEIN_MODIFICATION						
54187	NANS	N-acetylneuraminate synthase [Source:HGNC Symbol;Acc:HGNC:19237]				REACTOME_POST_TRANSLATIONAL_PROTEIN_MODIFICATION						
55907	CMAS	cytidine monophosphate N-acetylneuraminic acid synthetase [Source:HGNC Symbol;Acc:HGNC:18290]				REACTOME_POST_TRANSLATIONAL_PROTEIN_MODIFICATION						
126003	TRAPPC5	trafficking protein particle complex 5 [Source:HGNC Symbol;Acc:HGNC:23067]				REACTOME_POST_TRANSLATIONAL_PROTEIN_MODIFICATION						
27095	TRAPPC3	trafficking protein particle complex 3 [Source:HGNC Symbol;Acc:HGNC:19942]				REACTOME_POST_TRANSLATIONAL_PROTEIN_MODIFICATION						
55014	STX17	syntaxin 17 [Source:HGNC Symbol;Acc:HGNC:11432]				REACTOME_POST_TRANSLATIONAL_PROTEIN_MODIFICATION						
54732	TMED9	transmembrane p24 trafficking protein 9 [Source:HGNC Symbol;Acc:HGNC:24878]				REACTOME_POST_TRANSLATIONAL_PROTEIN_MODIFICATION						
57134	MAN1C1	mannosidase alpha class 1C member 1 [Source:HGNC Symbol;Acc:HGNC:19080]				REACTOME_POST_TRANSLATIONAL_PROTEIN_MODIFICATION						
9363	RAB33A	RAB33A, member RAS oncogene family [Source:HGNC Symbol;Acc:HGNC:9773]				REACTOME_POST_TRANSLATIONAL_PROTEIN_MODIFICATION						
10981	RAB32	RAB32, member RAS oncogene family [Source:HGNC Symbol;Acc:HGNC:9772]				REACTOME_POST_TRANSLATIONAL_PROTEIN_MODIFICATION						
79796	ALG9	ALG9 alpha-1,2-mannosyltransferase [Source:HGNC Symbol;Acc:HGNC:15672]				REACTOME_POST_TRANSLATIONAL_PROTEIN_MODIFICATION						
9945	GFPT2	glutamine-fructose-6-phosphate transaminase 2 [Source:HGNC Symbol;Acc:HGNC:4242]				REACTOME_POST_TRANSLATIONAL_PROTEIN_MODIFICATION						
144245	ALG10B	ALG10 alpha-1,2-glucosyltransferase B [Source:HGNC Symbol;Acc:HGNC:31088]				REACTOME_POST_TRANSLATIONAL_PROTEIN_MODIFICATION						
5828	PEX2	peroxisomal biogenesis factor 2 [Source:HGNC Symbol;Acc:HGNC:9717]				REACTOME_POST_TRANSLATIONAL_PROTEIN_MODIFICATION						
9781	RNF144A	ring finger protein 144A [Source:HGNC Symbol;Acc:HGNC:20457]				REACTOME_POST_TRANSLATIONAL_PROTEIN_MODIFICATION						
8459	TPST2	tyrosylprotein sulfotransferase 2 [Source:HGNC Symbol;Acc:HGNC:12021]				REACTOME_POST_TRANSLATIONAL_PROTEIN_MODIFICATION						
8460	TPST1	tyrosylprotein sulfotransferase 1 [Source:HGNC Symbol;Acc:HGNC:12020]				REACTOME_POST_TRANSLATIONAL_PROTEIN_MODIFICATION						
11098	PRSS23	serine protease 23 [Source:HGNC Symbol;Acc:HGNC:14370]				REACTOME_POST_TRANSLATIONAL_PROTEIN_MODIFICATION						
51280	GOLM1	golgi membrane protein 1 [Source:HGNC Symbol;Acc:HGNC:15451]				REACTOME_POST_TRANSLATIONAL_PROTEIN_MODIFICATION						
54587	MXRA8	matrix remodeling associated 8 [Source:HGNC Symbol;Acc:HGNC:7542]				REACTOME_POST_TRANSLATIONAL_PROTEIN_MODIFICATION						
1161	ERCC8	ERCC excision repair 8, CSA ubiquitin ligase complex subunit [Source:HGNC Symbol;Acc:HGNC:3439]				REACTOME_POST_TRANSLATIONAL_PROTEIN_MODIFICATION						
51602	NOP58	NOP58 ribonucleoprotein [Source:HGNC Symbol;Acc:HGNC:29926]				REACTOME_POST_TRANSLATIONAL_PROTEIN_MODIFICATION						
376267	RAB15	RAB15, member RAS oncogene family [Source:HGNC Symbol;Acc:HGNC:20150]				REACTOME_POST_TRANSLATIONAL_PROTEIN_MODIFICATION						
55647	RAB20	RAB20, member RAS oncogene family [Source:HGNC Symbol;Acc:HGNC:18260]				REACTOME_POST_TRANSLATIONAL_PROTEIN_MODIFICATION						
64284	RAB17	RAB17, member RAS oncogene family [Source:HGNC Symbol;Acc:HGNC:16523]				REACTOME_POST_TRANSLATIONAL_PROTEIN_MODIFICATION						
120526	DNAJC24	DnaJ heat shock protein family (Hsp40) member C24 [Source:HGNC Symbol;Acc:HGNC:26979]				REACTOME_POST_TRANSLATIONAL_PROTEIN_MODIFICATION						
285381	DPH3	diphthamide biosynthesis 3 [Source:HGNC Symbol;Acc:HGNC:27717]				REACTOME_POST_TRANSLATIONAL_PROTEIN_MODIFICATION						
122416	ANKRD9	ankyrin repeat domain 9 [Source:HGNC Symbol;Acc:HGNC:20096]				REACTOME_POST_TRANSLATIONAL_PROTEIN_MODIFICATION						
23142	DCUN1D4	defective in cullin neddylation 1 domain containing 4 [Source:HGNC Symbol;Acc:HGNC:28998]				REACTOME_POST_TRANSLATIONAL_PROTEIN_MODIFICATION						
51667	NUB1	negative regulator of ubiquitin like proteins 1 [Source:HGNC Symbol;Acc:HGNC:17623]				REACTOME_POST_TRANSLATIONAL_PROTEIN_MODIFICATION						
54876	DCAF16	DDB1 and CUL4 associated factor 16 [Source:HGNC Symbol;Acc:HGNC:25987]				REACTOME_POST_TRANSLATIONAL_PROTEIN_MODIFICATION						
55650	PIGV	phosphatidylinositol glycan anchor biosynthesis class V [Source:HGNC Symbol;Acc:HGNC:26031]				REACTOME_POST_TRANSLATIONAL_PROTEIN_MODIFICATION						

80055	PGAP1	post-GPI attachment to proteins inositol deacylase 1 [Source:HGNC Symbol;Acc:HGNC:25712]			REACTOME_POST_TRANSLATIONAL_PROTEIN_MODIFICATION						
5196	PF4	platelet factor 4 [Source:HGNC Symbol;Acc:HGNC:8861]				REACTOME_SIGNALING_BY_GPCR	REACTOME_RNA_POLYMERASE_II_TRANSCRIPTION	REACTOME_HEMOSTASIS			KEGG_CYTOKINE_CYTOKINE_RECEPTOR_INTERACTION
814	CAMK4	calcium/calmodulin dependent protein kinase IV [Source:HGNC Symbol;Acc:HGNC:1464]				REACTOME_SIGNALING_BY_GPCR	REACTOME_RNA_POLYMERASE_II_TRANSCRIPTION				
5739	PTGIR	prostaglandin I2 receptor [Source:HGNC Symbol;Acc:HGNC:9602]				REACTOME_SIGNALING_BY_GPCR		REACTOME_HEMOSTASIS			
64805	P2RY12	purinergic receptor P2Y12 [Source:HGNC Symbol;Acc:HGNC:18124]				REACTOME_SIGNALING_BY_GPCR		REACTOME_HEMOSTASIS			
2783	GNB2	G protein subunit beta 2 [Source:HGNC Symbol;Acc:HGNC:4398]				REACTOME_SIGNALING_BY_GPCR		REACTOME_HEMOSTASIS			
2787	GNG5	G protein subunit gamma 5 [Source:HGNC Symbol;Acc:HGNC:4408]				REACTOME_SIGNALING_BY_GPCR		REACTOME_HEMOSTASIS			
2791	GNG11	G protein subunit gamma 11 [Source:HGNC Symbol;Acc:HGNC:4403]				REACTOME_SIGNALING_BY_GPCR		REACTOME_HEMOSTASIS			
2790	GNG10	G protein subunit gamma 10 [Source:HGNC Symbol;Acc:HGNC:4402]				REACTOME_SIGNALING_BY_GPCR		REACTOME_HEMOSTASIS			
2793	GNGT2	G protein subunit gamma transducin 2 [Source:HGNC Symbol;Acc:HGNC:4412]				REACTOME_SIGNALING_BY_GPCR		REACTOME_HEMOSTASIS			
5577	PRKAR2B	protein kinase cAMP-dependent type II regulatory subunit beta [Source:HGNC Symbol;Acc:HGNC:9392]				REACTOME_SIGNALING_BY_GPCR		REACTOME_HEMOSTASIS			
388	RHOB	ras homolog family member B [Source:HGNC Symbol;Acc:HGNC:668]				REACTOME_SIGNALING_BY_GPCR		REACTOME_HEMOSTASIS			
2769	GNA15	G protein subunit alpha 15 [Source:HGNC Symbol;Acc:HGNC:4383]				REACTOME_SIGNALING_BY_GPCR		REACTOME_HEMOSTASIS			
747	DAGLA	diacylglycerol lipase alpha [Source:HGNC Symbol;Acc:HGNC:1165]				REACTOME_SIGNALING_BY_GPCR		REACTOME_HEMOSTASIS			
160851	DGKH	diacylglycerol kinase eta [Source:HGNC Symbol;Acc:HGNC:2854]				REACTOME_SIGNALING_BY_GPCR		REACTOME_HEMOSTASIS			
2768	GNA12	G protein subunit alpha 12 [Source:HGNC Symbol;Acc:HGNC:4380]				REACTOME_SIGNALING_BY_GPCR		REACTOME_HEMOSTASIS			
56477	CCL28	C-C motif chemokine ligand 28 [Source:HGNC Symbol;Acc:HGNC:17700]				REACTOME_SIGNALING_BY_GPCR					KEGG_CYTOKINE_CYTOKINE_RECEPTOR_INTERACTION
6375	XCL1	X-C motif chemokine ligand 1 [Source:HGNC Symbol;Acc:HGNC:10645]				REACTOME_SIGNALING_BY_GPCR					KEGG_CYTOKINE_CYTOKINE_RECEPTOR_INTERACTION
643	CXCR5	C-X-C motif chemokine receptor 5 [Source:HGNC Symbol;Acc:HGNC:1060]				REACTOME_SIGNALING_BY_GPCR					KEGG_CYTOKINE_CYTOKINE_RECEPTOR_INTERACTION
58191	CXCL16	C-X-C motif chemokine ligand 16 [Source:HGNC Symbol;Acc:HGNC:16642]				REACTOME_SIGNALING_BY_GPCR					KEGG_CYTOKINE_CYTOKINE_RECEPTOR_INTERACTION
2833	CXCR3	C-X-C motif chemokine receptor 3 [Source:HGNC Symbol;Acc:HGNC:4540]				REACTOME_SIGNALING_BY_GPCR					KEGG_CYTOKINE_CYTOKINE_RECEPTOR_INTERACTION
1236	CCR7	C-C motif chemokine receptor 7 [Source:HGNC Symbol;Acc:HGNC:1608]				REACTOME_SIGNALING_BY_GPCR					KEGG_CYTOKINE_CYTOKINE_RECEPTOR_INTERACTION
3640	INSL3	insulin like 3 [Source:HGNC Symbol;Acc:HGNC:6086]				REACTOME_SIGNALING_BY_GPCR					
3363	HTR7	5-hydroxytryptamine receptor 7 [Source:HGNC Symbol;Acc:HGNC:5302]				REACTOME_SIGNALING_BY_GPCR					
57105	CYSLTR2	cysteinyl leukotriene receptor 2 [Source:HGNC Symbol;Acc:HGNC:18274]				REACTOME_SIGNALING_BY_GPCR					
10800	CYSLTR1	cysteinyl leukotriene receptor 1 [Source:HGNC Symbol;Acc:HGNC:17451]				REACTOME_SIGNALING_BY_GPCR					
5732	PTGER2	prostaglandin E receptor 2 [Source:HGNC Symbol;Acc:HGNC:9594]				REACTOME_SIGNALING_BY_GPCR					
1816	DRD5	dopamine receptor D5 [Source:HGNC Symbol;Acc:HGNC:3026]				REACTOME_SIGNALING_BY_GPCR					
134864	TAAR1	trace amine associated receptor 1 [Source:HGNC Symbol;Acc:HGNC:17734]				REACTOME_SIGNALING_BY_GPCR					
8698	S1PR4	sphingosine-1-phosphate receptor 4 [Source:HGNC Symbol;Acc:HGNC:3170]				REACTOME_SIGNALING_BY_GPCR					
57121	LPAR5	lysophosphatidic acid receptor 5 [Source:HGNC Symbol;Acc:HGNC:13307]				REACTOME_SIGNALING_BY_GPCR					
10316	NMUR1	neuromedin U receptor 1 [Source:HGNC Symbol;Acc:HGNC:4518]				REACTOME_SIGNALING_BY_GPCR					
1269	CNR2	cannabinoid receptor 2 [Source:HGNC Symbol;Acc:HGNC:2160]				REACTOME_SIGNALING_BY_GPCR					
1815	DRD4	dopamine receptor D4 [Source:HGNC Symbol;Acc:HGNC:3025]				REACTOME_SIGNALING_BY_GPCR					
3355	HTR1F	5-hydroxytryptamine receptor 1F [Source:HGNC Symbol;Acc:HGNC:5292]				REACTOME_SIGNALING_BY_GPCR					
11251	PTGDR2	prostaglandin D2 receptor 2 [Source:HGNC Symbol;Acc:HGNC:4502]				REACTOME_SIGNALING_BY_GPCR					
9294	S1PR2	sphingosine-1-phosphate receptor 2 [Source:HGNC Symbol;Acc:HGNC:3169]				REACTOME_SIGNALING_BY_GPCR					
53637	S1PR5	sphingosine-1-phosphate receptor 5 [Source:HGNC Symbol;Acc:HGNC:14299]				REACTOME_SIGNALING_BY_GPCR					
50865	HEBP1	heme binding protein 1 [Source:HGNC Symbol;Acc:HGNC:17176]				REACTOME_SIGNALING_BY_GPCR					
9290	GPR55	G protein-coupled receptor 55 [Source:HGNC Symbol;Acc:HGNC:4511]				REACTOME_SIGNALING_BY_GPCR					
10161	LPAR6	lysophosphatidic acid receptor 6 [Source:HGNC Symbol;Acc:HGNC:15520]				REACTOME_SIGNALING_BY_GPCR					
5021	OXTR	oxytocin receptor [Source:HGNC Symbol;Acc:HGNC:8529]				REACTOME_SIGNALING_BY_GPCR					
1241	LTB4R	leukotriene B4 receptor [Source:HGNC Symbol;Acc:HGNC:6713]				REACTOME_SIGNALING_BY_GPCR					
4923	NTSR1	neurotensin receptor 1 [Source:HGNC Symbol;Acc:HGNC:8039]				REACTOME_SIGNALING_BY_GPCR					
5029	P2RY2	purinergic receptor P2Y2 [Source:HGNC Symbol;Acc:HGNC:8541]				REACTOME_SIGNALING_BY_GPCR					
5031	P2RY6	pyrimidinergic receptor P2Y6 [Source:HGNC Symbol;Acc:HGNC:8543]				REACTOME_SIGNALING_BY_GPCR					
27334	P2RY10	P2Y receptor family member 10 [Source:HGNC Symbol;Acc:HGNC:19906]				REACTOME_SIGNALING_BY_GPCR					
1131	CHRM3	cholinergic receptor muscarinic 3 [Source:HGNC Symbol;Acc:HGNC:1952]				REACTOME_SIGNALING_BY_GPCR					
8111	GPR68	G protein-coupled receptor 68 [Source:HGNC Symbol;Acc:HGNC:4519]				REACTOME_SIGNALING_BY_GPCR					
2865	FFAR3	free fatty acid receptor 3 [Source:HGNC Symbol;Acc:HGNC:4499]				REACTOME_SIGNALING_BY_GPCR					
2828	GPR4	G protein-coupled receptor 4 [Source:HGNC Symbol;Acc:HGNC:4497]				REACTOME_SIGNALING_BY_GPCR					
51738	GHRL	ghrelin and obestatin prepropeptide [Source:HGNC Symbol;Acc:HGNC:18129]				REACTOME_SIGNALING_BY_GPCR					
3060	HCRT	hypocretin neuropeptide precursor [Source:HGNC Symbol;Acc:HGNC:4847]				REACTOME_SIGNALING_BY_GPCR					
4935	GPR143	G protein-coupled receptor 143 [Source:HGNC Symbol;Acc:HGNC:20145]				REACTOME_SIGNALING_BY_GPCR					
1240	CMKLR1	chemerin chemokine-like receptor 1 [Source:HGNC Symbol;Acc:HGNC:2121]				REACTOME_SIGNALING_BY_GPCR					
7504	XK	X-linked Kx blood group [Source:HGNC Symbol;Acc:HGNC:12811]				REACTOME_SIGNALING_BY_GPCR					
79948	PLPPR3	phospholipid phosphatase related 3 [Source:HGNC Symbol;Acc:HGNC:23497]				REACTOME_SIGNALING_BY_GPCR					
133	ADM	adrenomedullin [Source:HGNC Symbol;Acc:HGNC:259]				REACTOME_SIGNALING_BY_GPCR					
259285	TAS2R39	taste 2 receptor member 39 [Source:HGNC Symbol;Acc:HGNC:18886]				REACTOME_SIGNALING_BY_GPCR					
80835	TAS1R1	taste 1 receptor member 1 [Source:HGNC Symbol;Acc:HGNC:14448]				REACTOME_SIGNALING_BY_GPCR					
259293	TAS2R30	taste 2 receptor member 30 [Source:HGNC Symbol;Acc:HGNC:19112]				REACTOME_SIGNALING_BY_GPCR					
8323	FZD6	frizzled class receptor 6 [Source:HGNC Symbol;Acc:HGNC:4044]				REACTOME_SIGNALING_BY_GPCR					
2015	ADGRE1	adhesion G protein-coupled receptor E1 [Source:HGNC Symbol;Acc:HGNC:3336]				REACTOME_SIGNALING_BY_GPCR					
114131	UCN3	urocortin 3 [Source:HGNC Symbol;Acc:HGNC:17781]				REACTOME_SIGNALING_BY_GPCR					
115	ADCY9	adenylate cyclase 9 [Source:HGNC Symbol;Acc:HGNC:240]				REACTOME_SIGNALING_BY_GPCR					
5613	PRKX	protein kinase X-linked [Source:HGNC Symbol;Acc:HGNC:9441]				REACTOME_SIGNALING_BY_GPCR					
2781	GNAZ	G protein subunit alpha z [Source:HGNC Symbol;Acc:HGNC:4395]				REACTOME_SIGNALING_BY_GPCR					
22899	ARHGEF15	Rho guanine nucleotide exchange factor 15 [Source:HGNC Symbol;Acc:HGNC:15590]				REACTOME_SIGNALING_BY_GPCR					
7074	TIAM1	TIAM Rac1 associated GEF 1 [Source:HGNC Symbol;Acc:HGNC:11805]				REACTOME_SIGNALING_BY_GPCR					
23365	ARHGEF12	Rho guanine nucleotide exchange factor 12 [Source:HGNC Symbol;Acc:HGNC:14193]				REACTOME_SIGNALING_BY_GPCR					
55160	ARHGEF10L	Rho guanine nucleotide exchange factor 10 like [Source:HGNC Symbol;Acc:HGNC:25540]				REACTOME_SIGNALING_BY_GPCR					
55701	ARHGEF40	Rho guanine nucleotide exchange factor 40 [Source:HGNC Symbol;Acc:HGNC:25516]				REACTOME_SIGNALING_BY_GPCR					
10645	CAMKK2	calcium/calmodulin dependent protein kinase kinase 2 [Source:HGNC Symbol;Acc:HGNC:1470]				REACTOME_SIGNALING_BY_GPCR					
5996	RGS1	regulator of G protein signaling 1 [Source:HGNC Symbol;Acc:HGNC:9991]				REACTOME_SIGNALING_BY_GPCR					
5998	RGS3	regulator of G protein signaling 3 [Source:HGNC Symbol;Acc:HGNC:9999]				REACTOME_SIGNALING_BY_GPCR					
5533	PPP3CC	protein phosphatase 3 catalytic subunit gamma [Source:HGNC Symbol;Acc:HGNC:9316]				REACTOME_SIGNALING_BY_GPCR					
8787	RGS9	regulator of G protein signaling 9 [Source:HGNC Symbol;Acc:HGNC:10004]				REACTOME_SIGNALING_BY_GPCR					
6001	RGS10	regulator of G protein signaling 10 [Source:HGNC Symbol;Acc:HGNC:9992]				REACTOME_SIGNALING_BY_GPCR					
10636	RGS14	regulator of G protein signaling 14 [Source:HGNC Symbol;Acc:HGNC:9996]				REACTOME_SIGNALING_BY_GPCR					
63940	GPSM3	G protein signaling modulator 3 [Source:HGNC Symbol;Acc:HGNC:13945]				REACTOME_SIGNALING_BY_GPCR					
3676	ITGA4	integrin subunit alpha 4 [Source:HGNC Symbol;Acc:HGNC:6140]					REACTOME_RNA_POLYMERASE_II_TRANSCRIPTION	REACTOME_HEMOSTASIS	KEGG_CELL_ADHESION_MOLECULES_CAMS		
2623	GATA1	GATA binding protein 1 [Source:HGNC Symbol;Acc:HGNC:4170]					REACTOME_RNA_POLYMERASE_II_TRANSCRIPTION	REACTOME_HEMOSTASIS			
4778	NFE2	nuclear factor, erythroid 2 [Source:HGNC Symbol;Acc:HGNC:7780]					REACTOME_RNA_POLYMERASE_II_TRANSCRIPTION	REACTOME_HEMOSTASIS			
1493	CTLA4	cytotoxic T-lymphocyte associated protein 4 [Source:HGNC Symbol;Acc:HGNC:2505]					REACTOME_RNA_POLYMERASE_II_TRANSCRIPTION		KEGG_CELL_ADHESION_MOLECULES_CAMS		
650	BMP2	bone morphogenetic protein 2 [Source:HGNC Symbol;Acc:HGNC:1069]					REACTOME_RNA_POLYMERASE_II_TRANSCRIPTION				KEGG_CYTOKINE_CYTOKINE_RECEPTOR_INTERACTION
23327	NEDD4L	NEDD4 like E3 ubiquitin protein ligase [Source:HGNC Symbol;Acc:HGNC:7728]					REACTOME_RNA_POLYMERASE_II_TRANSCRIPTION				
11059	WWP1	WW domain containing E3 ubiquitin protein ligase 1 [Source:HGNC Symbol;Acc:HGNC:17004]					REACTOME_RNA_POLYMERASE_II_TRANSCRIPTION				
55615	PRR5	proline rich 5 [Source:HGNC Symbol;Acc:HGNC:31682]					REACTOME_RNA_POLYMERASE_II_TRANSCRIPTION				
10769	PLK2	polo like kinase 2 [Source:HGNC Symbol;Acc:HGNC:19699]					REACTOME_RNA_POLYMERASE_II_TRANSCRIPTION				
22916	NCBP2	nuclear cap binding protein subunit 2 [Source:HGNC Symbol;Acc:HGNC:7659]					REACTOME_RNA_POLYMERASE_II_TRANSCRIPTION				
10398	MYL9	myosin light chain 9 [Source:HGNC Symbol;Acc:HGNC:15754]					REACTOME_RNA_POLYMERASE_II_TRANSCRIPTION				
995	CDC25C	cell division cycle 25C [Source:HGNC Symbol;Acc:HGNC:1727]					REACTOME_RNA_POLYMERASE_II_TRANSCRIPTION				
7533	YWHAH	tyrosine 3-monooxygenase/tryptophan 5-monooxygenase activation protein eta [Source:HGNC Symbol;Acc:HGNC:12853]					REACTOME_RNA_POLYMERASE_II_TRANSCRIPTION				
6604	SMARCD3	SWI/SNF related, matrix associated, actin dependent regulator of chromatin, subfamily d, member 3 [Source:HGNC Symbol;Acc:HGNC:11108]					REACTOME_RNA_POLYMERASE_II_TRANSCRIPTION				
5565	PRKAB2	protein kinase AMP-activated non-catalytic subunit beta 2 [Source:HGNC Symbol;Acc:HGNC:9379]					REACTOME_RNA_POLYMERASE_II_TRANSCRIPTION				
3164	NR4A1	nuclear receptor subfamily 4 group A member 1 [Source:HGNC Symbol;Acc:HGNC:7980]					REACTOME_RNA_POLYMERASE_II_TRANSCRIPTION				
637	BID	BH3 interacting domain death agonist [Source:HGNC Symbol;Acc:HGNC:1050]					REACTOME_RNA_POLYMERASE_II_TRANSCRIPTION				
4094	MAF	MAF bZIP transcription factor [Source:HGNC Symbol;Acc:HGNC:6776]					REACTOME_RNA_POLYMERASE_II_TRANSCRIPTION				
6886	TAL1	TAL bHLH transcription factor 1, erythroid differentiation factor [Source:HGNC Symbol;Acc:HGNC:11556]					REACTOME_RNA_POLYMERASE_II_TRANSCRIPTION				
4343	MOV10	Mov10 RISC complex RNA helicase [Source:HGNC Symbol;Acc:HGNC:7200]					REACTOME_RNA_POLYMERASE_II_TRANSCRIPTION				
8013	NR4A3	nuclear receptor subfamily 4 group A member 3 [Source:HGNC Symbol;Acc:HGNC:7982]					REACTOME_RNA_POLYMERASE_II_TRANSCRIPTION				
6929	TCF3	transcription factor 3 [Source:HGNC Symbol;Acc:HGNC:11633]					REACTOME_RNA_POLYMERASE_II_TRANSCRIPTION				
51176	LEF1	lymphoid enhancer binding factor 1 [Source:HGNC Symbol;Acc:HGNC:6551]					REACTOME_RNA_POLYMERASE_II_TRANSCRIPTION				
2744	GLS	glutaminase [Source:HGNC Symbol;Acc:HGNC:4331]					REACTOME_RNA_POLYMERASE_II_TRANSCRIPTION				
1021	CDK6	cyclin dependent kinase 6 [Source:HGNC Symbol;Acc:HGNC:1777]					REACTOME_RNA_POLYMERASE_II_TRANSCRIPTION				
27161	AGO2	argonaute RISC catalytic component 2 [Source:HGNC Symbol;Acc:HGNC:3263]					REACTOME_RNA_POLYMERASE_II_TRANSCRIPTION				
7082	TJP1	tight junction protein 1 [Source:HGNC Symbol;Acc:HGNC:11827]					REACTOME_RNA_POLYMERASE_II_TRANSCRIPTION				
26053	AUTS2	activator of transcription and developmental regulator AUTS2 [Source:HGNC Symbol;Acc:HGNC:14262]					REACTOME_RNA_POLYMERASE_II_TRANSCRIPTION				
84333	PCGF5	polycomb group ring finger 5 [Source:HGNC Symbol;Acc:HGNC:28264]					REACTOME_RNA_POLYMERASE_II_TRANSCRIPTION				
1030	CDKN2B	cyclin dependent kinase inhibitor 2B [Source:HGNC Symbol;Acc:HGNC:1788]					REACTOME_RNA_POLYMERASE_II_TRANSCRIPTION				
545	ATR	ATR serine/threonine kinase [Source:HGNC Symbol;Acc:HGNC:882]					REACTOME_RNA_POLYMERASE_II_TRANSCRIPTION				
9540	TP53I3	tumor protein p53 inducible protein 3 [Source:HGNC Symbol;Acc:HGNC:19373]					REACTOME_RNA_POLYMERASE_II_TRANSCRIPTION				
1111	CHEK1	checkpoint kinase 1 [Source:HGNC Symbol;Acc:HGNC:1925]					REACTOME_RNA_POLYMERASE_II_TRANSCRIPTION				
55835	CENPJ	centromere protein J [Source:HGNC Symbol;Acc:HGNC:17272]					REACTOME_RNA_POLYMERASE_II_TRANSCRIPTION				
2289	FKBP5	FKBP prolyl isomerase 5 [Source:HGNC Symbol;Acc:HGNC:3721]					REACTOME_RNA_POLYMERASE_II_TRANSCRIPTION				
1647	GADD45A	growth arrest and DNA damage inducible alpha [Source:HGNC Symbol;Acc:HGNC:4095]					REACTOME_RNA_POLYMERASE_II_TRANSCRIPTION				
58528	RRAGD	Ras related GTP binding D [Source:HGNC Symbol;Acc:HGNC:19903]					REACTOME_RNA_POLYMERASE_II_TRANSCRIPTION				
10542	LAMTOR5	late endosomal/lysosomal adaptor, MAPK and MTOR activator 5 [Source:HGNC Symbol;Acc:HGNC:17955]					REACTOME_RNA_POLYMERASE_II_TRANSCRIPTION				
25824	PRDX5	peroxiredoxin 5 [Source:HGNC Symbol;Acc:HGNC:9355]					REACTOME_RNA_POLYMERASE_II_TRANSCRIPTION				
6932	TCF7	transcription factor 7 [Source:HGNC Symbol;Acc:HGNC:11639]					REACTOME_RNA_POLYMERASE_II_TRANSCRIPTION				
864	RUNX3	RUNX family transcription factor 3 [Source:HGNC Symbol;Acc:HGNC:10473]					REACTOME_RNA_POLYMERASE_II_TRANSCRIPTION				
27113	BBC3	BCL2 binding component 3 [Source:HGNC Symbol;Acc:HGNC:17868]					REACTOME_RNA_POLYMERASE_II_TRANSCRIPTION				
9975	NR1D2	nuclear receptor subfamily 1 group D member 2 [Source:HGNC Symbol;Acc:HGNC:7963]					REACTOME_RNA_POLYMERASE_II_TRANSCRIPTION				
5458	POU4F2	POU class 4 homeobox 2 [Source:HGNC Symbol;Acc:HGNC:9219]					REACTOME_RNA_POLYMERASE_II_TRANSCRIPTION				
79370	BCL2L14	BCL2 like 14 [Source:HGNC Symbol;Acc:HGNC:16657]					REACTOME_RNA_POLYMERASE_II_TRANSCRIPTION				
28984	RGCC	regulator of cell cycle [Source:HGNC Symbol;Acc:HGNC:20369]					REACTOME_RNA_POLYMERASE_II_TRANSCRIPTION				
56950	SMYD2	SET and MYND domain containing 2 [Source:HGNC Symbol;Acc:HGNC:20982]					REACTOME_RNA_POLYMERASE_II_TRANSCRIPTION				
2176	FANCC	FA complementation group C [Source:HGNC Symbol;Acc:HGNC:3584]					REACTOME_RNA_POLYMERASE_II_TRANSCRIPTION				
133383	SETD9	SET domain containing 9 [Source:HGNC Symbol;Acc:HGNC:28508]					REACTOME_RNA_POLYMERASE_II_TRANSCRIPTION				
5816	PVALB	parvalbumin [Source:HGNC Symbol;Acc:HGNC:9704]					REACTOME_RNA_POLYMERASE_II_TRANSCRIPTION				
114787	GPRIN1	G protein regulated inducer of neurite outgrowth 1 [Source:HGNC Symbol;Acc:HGNC:24835]					REACTOME_RNA_POLYMERASE_II_TRANSCRIPTION				
79047	KCTD15	potassium channel tetramerization domain containing 15 [Source:HGNC Symbol;Acc:HGNC:23297]					REACTOME_RNA_POLYMERASE_II_TRANSCRIPTION				
25888	ZNF473	zinc finger protein 473 [Source:HGNC Symbol;Acc:HGNC:23239]					REACTOME_RNA_POLYMERASE_II_TRANSCRIPTION				
7561	ZNF14	zinc finger protein 14 [Source:HGNC Symbol;Acc:HGNC:12924]					REACTOME_RNA_POLYMERASE_II_TRANSCRIPTION				
9984	THOC1	THO complex 1 [Source:HGNC Symbol;Acc:HGNC:19070]					REACTOME_RNA_POLYMERASE_II_TRANSCRIPTION				
57508	INTS2	integrator complex subunit 2 [Source:HGNC Symbol;Acc:HGNC:29241]					REACTOME_RNA_POLYMERASE_II_TRANSCRIPTION				
80237	ELL3	elongation factor for RNA polymerase II 3 [Source:HGNC Symbol;Acc:HGNC:23113]					REACTOME_RNA_POLYMERASE_II_TRANSCRIPTION				
4300	MLLT3	MLLT3 super elongation complex subunit [Source:HGNC Symbol;Acc:HGNC:7136]					REACTOME_RNA_POLYMERASE_II_TRANSCRIPTION				
100129842	ZNF737	zinc finger protein 737 [Source:HGNC Symbol;Acc:HGNC:32468]					REACTOME_RNA_POLYMERASE_II_TRANSCRIPTION				
100287226	ZNF729	zinc finger protein 729 [Source:HGNC Symbol;Acc:HGNC:32464]					REACTOME_RNA_POLYMERASE_II_TRANSCRIPTION				
117608	ZNF354B	zinc finger protein 354B [Source:HGNC Symbol;Acc:HGNC:17197]					REACTOME_RNA_POLYMERASE_II_TRANSCRIPTION				
126231	ZNF573	zinc finger protein 573 [Source:HGNC Symbol;Acc:HGNC:26420]					REACTOME_RNA_POLYMERASE_II_TRANSCRIPTION				
146198	ZFP90	ZFP90 zinc finger protein [Source:HGNC Symbol;Acc:HGNC:23329]					REACTOME_RNA_POLYMERASE_II_TRANSCRIPTION				
155054	ZNF425	zinc finger protein 425 [Source:HGNC Symbol;Acc:HGNC:20690]					REACTOME_RNA_POLYMERASE_II_TRANSCRIPTION				
162239	ZFP1	ZFP1 zinc finger protein [Source:HGNC Symbol;Acc:HGNC:23328]					REACTOME_RNA_POLYMERASE_II_TRANSCRIPTION				
162966	ZNF600	zinc finger protein 600 [Source:HGNC Symbol;Acc:HGNC:30951]					REACTOME_RNA_POLYMERASE_II_TRANSCRIPTION				
162967	ZNF320	zinc finger protein 320 [Source:HGNC Symbol;Acc:HGNC:13842]					REACTOME_RNA_POLYMERASE_II_TRANSCRIPTION				
163081	ZNF567	zinc finger protein 567 [Source:HGNC Symbol;Acc:HGNC:28696]					REACTOME_RNA_POLYMERASE_II_TRANSCRIPTION				
163223	ZNF676	zinc finger protein 676 [Source:HGNC Symbol;Acc:HGNC:20429]					REACTOME_RNA_POLYMERASE_II_TRANSCRIPTION				
163255	ZNF540	zinc finger protein 540 [Source:HGNC Symbol;Acc:HGNC:25331]					REACTOME_RNA_POLYMERASE_II_TRANSCRIPTION				
168374	ZNF92	zinc finger protein 92 [Source:HGNC Symbol;Acc:HGNC:13168]					REACTOME_RNA_POLYMERASE_II_TRANSCRIPTION				
169270	ZNF596	zinc finger protein 596 [Source:HGNC Symbol;Acc:HGNC:27268]					REACTOME_RNA_POLYMERASE_II_TRANSCRIPTION				
26152	ZNF337	zinc finger protein 337 [Source:HGNC Symbol;Acc:HGNC:15809]					REACTOME_RNA_POLYMERASE_II_TRANSCRIPTION				
283337	ZNF740	zinc finger protein 740 [Source:HGNC Symbol;Acc:HGNC:27465]					REACTOME_RNA_POLYMERASE_II_TRANSCRIPTION				
284459	ZNF875	zinc finger protein 875 [Source:HGNC Symbol;Acc:HGNC:4928]					REACTOME_RNA_POLYMERASE_II_TRANSCRIPTION				
285971	ZNF775	zinc finger protein 775 [Source:HGNC Symbol;Acc:HGNC:28501]					REACTOME_RNA_POLYMERASE_II_TRANSCRIPTION				
339327	ZNF546	zinc finger protein 546 [Source:HGNC Symbol;Acc:HGNC:28671]					REACTOME_RNA_POLYMERASE_II_TRANSCRIPTION				
339559	ZFP69	ZFP69 zinc finger protein [Source:HGNC Symbol;Acc:HGNC:24708]					REACTOME_RNA_POLYMERASE_II_TRANSCRIPTION				
388567	ZNF749	zinc finger protein 749 [Source:HGNC Symbol;Acc:HGNC:32783]					REACTOME_RNA_POLYMERASE_II_TRANSCRIPTION				
51276	ZNF571	zinc finger protein 571 [Source:HGNC Symbol;Acc:HGNC:25000]					REACTOME_RNA_POLYMERASE_II_TRANSCRIPTION				
55422	ZNF331	zinc finger protein 331 [Source:HGNC Symbol;Acc:HGNC:15489]					REACTOME_RNA_POLYMERASE_II_TRANSCRIPTION				
55786	ZNF415	zinc finger protein 415 [Source:HGNC Symbol;Acc:HGNC:20636]					REACTOME_RNA_POLYMERASE_II_TRANSCRIPTION				
56242	ZNF253	zinc finger protein 253 [Source:HGNC Symbol;Acc:HGNC:13497]					REACTOME_RNA_POLYMERASE_II_TRANSCRIPTION				
57209	ZNF248	zinc finger protein 248 [Source:HGNC Symbol;Acc:HGNC:13041]					REACTOME_RNA_POLYMERASE_II_TRANSCRIPTION				
57343	ZNF304	zinc finger protein 304 [Source:HGNC Symbol;Acc:HGNC:13505]					REACTOME_RNA_POLYMERASE_II_TRANSCRIPTION				
654254	ZNF732	zinc finger protein 732 [Source:HGNC Symbol;Acc:HGNC:37138]					REACTOME_RNA_POLYMERASE_II_TRANSCRIPTION				
6940	ZNF354A	zinc finger protein 354A [Source:HGNC Symbol;Acc:HGNC:11628]					REACTOME_RNA_POLYMERASE_II_TRANSCRIPTION				
728927	ZNF736	zinc finger protein 736 [Source:HGNC Symbol;Acc:HGNC:32467]					REACTOME_RNA_POLYMERASE_II_TRANSCRIPTION				
7562	ZNF708	zinc finger protein 708 [Source:HGNC Symbol;Acc:HGNC:12945]					REACTOME_RNA_POLYMERASE_II_TRANSCRIPTION				
7571	ZNF23	zinc finger protein 23 [Source:HGNC Symbol;Acc:HGNC:13023]					REACTOME_RNA_POLYMERASE_II_TRANSCRIPTION				
7678	ZNF124	zinc finger protein 124 [Source:HGNC Symbol;Acc:HGNC:12907]					REACTOME_RNA_POLYMERASE_II_TRANSCRIPTION				
7695	ZNF136	zinc finger protein 136 [Source:HGNC Symbol;Acc:HGNC:12920]					REACTOME_RNA_POLYMERASE_II_TRANSCRIPTION				
7697	ZNF138	zinc finger protein 138 [Source:HGNC Symbol;Acc:HGNC:12922]					REACTOME_RNA_POLYMERASE_II_TRANSCRIPTION				
7748	ZNF195	zinc finger protein 195 [Source:HGNC Symbol;Acc:HGNC:12986]					REACTOME_RNA_POLYMERASE_II_TRANSCRIPTION				
7757	ZNF208	zinc finger protein 208 [Source:HGNC Symbol;Acc:HGNC:12999]					REACTOME_RNA_POLYMERASE_II_TRANSCRIPTION				
7768	ZNF225	zinc finger protein 225 [Source:HGNC Symbol;Acc:HGNC:13018]					REACTOME_RNA_POLYMERASE_II_TRANSCRIPTION				
80264	ZNF430	zinc finger protein 430 [Source:HGNC Symbol;Acc:HGNC:20808]					REACTOME_RNA_POLYMERASE_II_TRANSCRIPTION				
84436	ZNF528	zinc finger protein 528 [Source:HGNC Symbol;Acc:HGNC:29384]					REACTOME_RNA_POLYMERASE_II_TRANSCRIPTION				
84527	ZNF559	zinc finger protein 559 [Source:HGNC Symbol;Acc:HGNC:28197]					REACTOME_RNA_POLYMERASE_II_TRANSCRIPTION				
84671	ZNF347	zinc finger protein 347 [Source:HGNC Symbol;Acc:HGNC:16447]					REACTOME_RNA_POLYMERASE_II_TRANSCRIPTION				
90075	ZNF30	zinc finger protein 30 [Source:HGNC Symbol;Acc:HGNC:13090]					REACTOME_RNA_POLYMERASE_II_TRANSCRIPTION				
90827	ZNF479	zinc finger protein 479 [Source:HGNC Symbol;Acc:HGNC:23258]					REACTOME_RNA_POLYMERASE_II_TRANSCRIPTION				
9310	ZNF235	zinc finger protein 235 [Source:HGNC Symbol;Acc:HGNC:12866]					REACTOME_RNA_POLYMERASE_II_TRANSCRIPTION				
4267	CD99	CD99 molecule (Xg blood group) [Source:HGNC Symbol;Acc:HGNC:7082]						REACTOME_HEMOSTASIS	KEGG_CELL_ADHESION_MOLECULES_CAMS		
3655	ITGA6	integrin subunit alpha 6 [Source:HGNC Symbol;Acc:HGNC:6142]						REACTOME_HEMOSTASIS	KEGG_CELL_ADHESION_MOLECULES_CAMS		
914	CD2	CD2 molecule [Source:HGNC Symbol;Acc:HGNC:1639]						REACTOME_HEMOSTASIS	KEGG_CELL_ADHESION_MOLECULES_CAMS		
83700	JAM3	junctional adhesion molecule 3 [Source:HGNC Symbol;Acc:HGNC:15532]						REACTOME_HEMOSTASIS	KEGG_CELL_ADHESION_MOLECULES_CAMS		
6693	SPN	sialophorin [Source:HGNC Symbol;Acc:HGNC:11249]						REACTOME_HEMOSTASIS	KEGG_CELL_ADHESION_MOLECULES_CAMS		
5197	PF4V1	platelet factor 4 variant 1 [Source:HGNC Symbol;Acc:HGNC:8862]						REACTOME_HEMOSTASIS			KEGG_CYTOKINE_CYTOKINE_RECEPTOR_INTERACTION
4352	MPL	MPL proto-oncogene, thrombopoietin receptor [Source:HGNC Symbol;Acc:HGNC:7217]						REACTOME_HEMOSTASIS			KEGG_CYTOKINE_CYTOKINE_RECEPTOR_INTERACTION
22920	KIFAP3	kinesin associated protein 3 [Source:HGNC Symbol;Acc:HGNC:17060]						REACTOME_HEMOSTASIS			
56992	KIF15	kinesin family member 15 [Source:HGNC Symbol;Acc:HGNC:17273]						REACTOME_HEMOSTASIS			
3700	ITIH4	inter-alpha-trypsin inhibitor heavy chain 4 [Source:HGNC Symbol;Acc:HGNC:6169]						REACTOME_HEMOSTASIS			
5552	SRGN	serglycin [Source:HGNC Symbol;Acc:HGNC:9361]						REACTOME_HEMOSTASIS			
5271	SERPINB8	serpin family B member 8 [Source:HGNC Symbol;Acc:HGNC:8952]						REACTOME_HEMOSTASIS			
3690	ITGB3	integrin subunit beta 3 [Source:HGNC Symbol;Acc:HGNC:6156]						REACTOME_HEMOSTASIS			
2812	GP1BB	glycoprotein Ib platelet subunit beta [Source:HGNC Symbol;Acc:HGNC:4440]						REACTOME_HEMOSTASIS			
85440	DOCK7	dedicator of cytokinesis 7 [Source:HGNC Symbol;Acc:HGNC:19190]						REACTOME_HEMOSTASIS			
57572	DOCK6	dedicator of cytokinesis 6 [Source:HGNC Symbol;Acc:HGNC:19189]						REACTOME_HEMOSTASIS			
10257	ABCC4	ATP binding cassette subfamily C member 4 [Source:HGNC Symbol;Acc:HGNC:55]						REACTOME_HEMOSTASIS			
51206	GP6	glycoprotein VI platelet [Source:HGNC Symbol;Acc:HGNC:14388]						REACTOME_HEMOSTASIS			
23052	ENDOD1	endonuclease domain containing 1 [Source:HGNC Symbol;Acc:HGNC:29129]						REACTOME_HEMOSTASIS			
10184	LHFPL2	LHFPL tetraspan subfamily member 2 [Source:HGNC Symbol;Acc:HGNC:6588]						REACTOME_HEMOSTASIS			
10447	FAM3C	FAM3 metabolism regulating signaling molecule C [Source:HGNC Symbol;Acc:HGNC:18664]						REACTOME_HEMOSTASIS			
22927	HABP4	hyaluronan binding protein 4 [Source:HGNC Symbol;Acc:HGNC:17062]						REACTOME_HEMOSTASIS			
80739	MPIG6B	megakaryocyte and platelet inhibitory receptor G6b [Source:HGNC Symbol;Acc:HGNC:13937]						REACTOME_HEMOSTASIS			
9046	DOK2	docking protein 2 [Source:HGNC Symbol;Acc:HGNC:2991]						REACTOME_HEMOSTASIS			
490	ATP2B1	ATPase plasma membrane Ca2+ transporting 1 [Source:HGNC Symbol;Acc:HGNC:814]						REACTOME_HEMOSTASIS			
9123	SLC16A3	solute carrier family 16 member 3 [Source:HGNC Symbol;Acc:HGNC:10924]						REACTOME_HEMOSTASIS			
8165	AKAP1	A-kinase anchoring protein 1 [Source:HGNC Symbol;Acc:HGNC:367]						REACTOME_HEMOSTASIS			
10461	MERTK	MER proto-oncogene, tyrosine kinase [Source:HGNC Symbol;Acc:HGNC:7027]						REACTOME_HEMOSTASIS			
3778	KCNMA1	potassium calcium-activated channel subfamily M alpha 1 [Source:HGNC Symbol;Acc:HGNC:6284]						REACTOME_HEMOSTASIS			
2982	GUCY1A1	guanylate cyclase 1 soluble subunit alpha 1 [Source:HGNC Symbol;Acc:HGNC:4685]						REACTOME_HEMOSTASIS			

2983	GUCY1B1	guanylate cyclase 1 soluble subunit beta 1 [Source:HGNC Symbol;Acc:HGNC:4687]	REACTOME_HEMOSTASIS			
7056	THBD	thrombomodulin [Source:HGNC Symbol;Acc:HGNC:11784]	REACTOME_HEMOSTASIS			
962	CD48	CD48 molecule [Source:HGNC Symbol;Acc:HGNC:1683]	REACTOME_HEMOSTASIS			
51744	CD244	CD244 molecule [Source:HGNC Symbol;Acc:HGNC:18171]	REACTOME_HEMOSTASIS			
2888	GRB14	growth factor receptor bound protein 14 [Source:HGNC Symbol;Acc:HGNC:4565]	REACTOME_HEMOSTASIS			
4072	EPCAM	epithelial cell adhesion molecule [Source:HGNC Symbol;Acc:HGNC:11529]	REACTOME_HEMOSTASIS			
83692	CD99L2	CD99 molecule like 2 [Source:HGNC Symbol;Acc:HGNC:18237]	REACTOME_HEMOSTASIS			
124602	KIF19	kinesin family member 19 [Source:HGNC Symbol;Acc:HGNC:26735]	REACTOME_HEMOSTASIS			
221458	KIF6	kinesin family member 6 [Source:HGNC Symbol;Acc:HGNC:21202]	REACTOME_HEMOSTASIS			
3046	HBE1	hemoglobin subunit epsilon 1 [Source:HGNC Symbol;Acc:HGNC:4830]	REACTOME_HEMOSTASIS			
3109	HLA-DMB	major histocompatibility complex, class II, DM beta [Source:HGNC Symbol;Acc:HGNC:4935]		KEGG_CELL_ADHESION_MOLECULES_CAMS	KEGG_SYSTEMIC_LUPUS_ERYTHEMATOSUS	
3111	HLA-DOA	major histocompatibility complex, class II, DO alpha [Source:HGNC Symbol;Acc:HGNC:4936]		KEGG_CELL_ADHESION_MOLECULES_CAMS	KEGG_SYSTEMIC_LUPUS_ERYTHEMATOSUS	
925	CD8A	CD8a molecule [Source:HGNC Symbol;Acc:HGNC:1706]		KEGG_CELL_ADHESION_MOLECULES_CAMS		
3695	ITGB7	integrin subunit beta 7 [Source:HGNC Symbol;Acc:HGNC:6162]		KEGG_CELL_ADHESION_MOLECULES_CAMS		
8174	MADCAM1	mucosal vascular addressin cell adhesion molecule 1 [Source:HGNC Symbol;Acc:HGNC:6765]		KEGG_CELL_ADHESION_MOLECULES_CAMS		
29851	ICOS	inducible T cell costimulator [Source:HGNC Symbol;Acc:HGNC:5351]		KEGG_CELL_ADHESION_MOLECULES_CAMS		
6614	SIGLEC1	sialic acid binding Ig like lectin 1 [Source:HGNC Symbol;Acc:HGNC:11127]		KEGG_CELL_ADHESION_MOLECULES_CAMS		
8516	ITGA8	integrin subunit alpha 8 [Source:HGNC Symbol;Acc:HGNC:6144]		KEGG_CELL_ADHESION_MOLECULES_CAMS		
3696	ITGB8	integrin subunit beta 8 [Source:HGNC Symbol;Acc:HGNC:6163]		KEGG_CELL_ADHESION_MOLECULES_CAMS		
214	ALCAM	activated leukocyte cell adhesion molecule [Source:HGNC Symbol;Acc:HGNC:400]		KEGG_CELL_ADHESION_MOLECULES_CAMS		
4359	MPZ	myelin protein zero [Source:HGNC Symbol;Acc:HGNC:7225]		KEGG_CELL_ADHESION_MOLECULES_CAMS		
5010	CLDN11	claudin 11 [Source:HGNC Symbol;Acc:HGNC:8514]		KEGG_CELL_ADHESION_MOLECULES_CAMS		
49861	CLDN20	claudin 20 [Source:HGNC Symbol;Acc:HGNC:2042]		KEGG_CELL_ADHESION_MOLECULES_CAMS		
51208	CLDN18	claudin 18 [Source:HGNC Symbol;Acc:HGNC:2039]		KEGG_CELL_ADHESION_MOLECULES_CAMS		
25945	NECTIN3	nectin cell adhesion molecule 3 [Source:HGNC Symbol;Acc:HGNC:17664]		KEGG_CELL_ADHESION_MOLECULES_CAMS		
5797	PTPRM	protein tyrosine phosphatase receptor type M [Source:HGNC Symbol;Acc:HGNC:9675]		KEGG_CELL_ADHESION_MOLECULES_CAMS		
26047	CNTNAP2	contactin associated protein 2 [Source:HGNC Symbol;Acc:HGNC:13830]		KEGG_CELL_ADHESION_MOLECULES_CAMS		
923	CD6	CD6 molecule [Source:HGNC Symbol;Acc:HGNC:1691]		KEGG_CELL_ADHESION_MOLECULES_CAMS		
6741	SSB	small RNA binding exonuclease protection factor La [Source:HGNC Symbol;Acc:HGNC:11316]			KEGG_SYSTEMIC_LUPUS_ERYTHEMATOSUS	
83740	H2AB3	H2A.B variant histone 3 [Source:HGNC Symbol;Acc:HGNC:14455]			KEGG_SYSTEMIC_LUPUS_ERYTHEMATOSUS	
4254	KITLG	KIT ligand [Source:HGNC Symbol;Acc:HGNC:6343]				KEGG_CYTOKINE_CYTOKINE_RECEPTOR_INTERACTION
6362	CCL18	C-C motif chemokine ligand 18 [Source:HGNC Symbol;Acc:HGNC:10616]				KEGG_CYTOKINE_CYTOKINE_RECEPTOR_INTERACTION
56034	PDGFC	platelet derived growth factor C [Source:HGNC Symbol;Acc:HGNC:8801]				KEGG_CYTOKINE_CYTOKINE_RECEPTOR_INTERACTION
3467	IFNW1	interferon omega 1 [Source:HGNC Symbol;Acc:HGNC:5448]				KEGG_CYTOKINE_CYTOKINE_RECEPTOR_INTERACTION
3625	INHBB	inhibin subunit beta B [Source:HGNC Symbol;Acc:HGNC:6067]				KEGG_CYTOKINE_CYTOKINE_RECEPTOR_INTERACTION
2321	FLT1	fms related receptor tyrosine kinase 1 [Source:HGNC Symbol;Acc:HGNC:3763]				KEGG_CYTOKINE_CYTOKINE_RECEPTOR_INTERACTION
91	ACVR1B	activin A receptor type 1B [Source:HGNC Symbol;Acc:HGNC:172]				KEGG_CYTOKINE_CYTOKINE_RECEPTOR_INTERACTION
659	BMPR2	bone morphogenetic protein receptor type 2 [Source:HGNC Symbol;Acc:HGNC:1078]				KEGG_CYTOKINE_CYTOKINE_RECEPTOR_INTERACTION
1308	COL17A1	collagen type XVII alpha 1 chain [Source:HGNC Symbol;Acc:HGNC:2194]				
915	CD3D	CD3d molecule [Source:HGNC Symbol;Acc:HGNC:1673]				
6456	SH3GL2	SH3 domain containing GRB2 like 2, endophilin A1 [Source:HGNC Symbol;Acc:HGNC:10831]				
51466	EVL	Enah/Vasp-like [Source:HGNC Symbol;Acc:HGNC:20234]				
7535	ZAP70	zeta chain of T cell receptor associated protein kinase 70 [Source:HGNC Symbol;Acc:HGNC:12858]				
117145	THEM4	thioesterase superfamily member 4 [Source:HGNC Symbol;Acc:HGNC:17947]				
3821	KLRC1	killer cell lectin like receptor C1 [Source:HGNC Symbol;Acc:HGNC:6374]				
868	CBLB	Cbl proto-oncogene B [Source:HGNC Symbol;Acc:HGNC:1542]				
7220	TRPC1	transient receptor potential cation channel subfamily C member 1 [Source:HGNC Symbol;Acc:HGNC:12333]				
7087	ICAM5	intercellular adhesion molecule 5 [Source:HGNC Symbol;Acc:HGNC:5348]				
4068	SH2D1A	SH2 domain containing 1A [Source:HGNC Symbol;Acc:HGNC:10820]				
10219	KLRG1	killer cell lectin like receptor G1 [Source:HGNC Symbol;Acc:HGNC:6380]				
10225	CD96	CD96 molecule [Source:HGNC Symbol;Acc:HGNC:16892]				
131450	CD200R1	CD200 receptor 1 [Source:HGNC Symbol;Acc:HGNC:24235]				
340205	TREML1	triggering receptor expressed on myeloid cells like 1 [Source:HGNC Symbol;Acc:HGNC:20434]				
353514	LILRA5	leukocyte immunoglobulin like receptor A5 [Source:HGNC Symbol;Acc:HGNC:16309]				
3820	KLRB1	killer cell lectin like receptor B1 [Source:HGNC Symbol;Acc:HGNC:6373]				
51348	KLRF1	killer cell lectin like receptor F1 [Source:HGNC Symbol;Acc:HGNC:13342]				
56253	CRTAM	cytotoxic and regulatory T cell molecule [Source:HGNC Symbol;Acc:HGNC:24313]				
3902	LAG3	lymphocyte activating 3 [Source:HGNC Symbol;Acc:HGNC:6476]				
25780	RASGRP3	RAS guanyl releasing protein 3 [Source:HGNC Symbol;Acc:HGNC:14545]				
50852	TRAT1	T cell receptor associated transmembrane adaptor 1 [Source:HGNC Symbol;Acc:HGNC:30698]				
255488	RNF144B	ring finger protein 144B [Source:HGNC Symbol;Acc:HGNC:21578]				
10952	SEC61B	SEC61 translocon subunit beta [Source:HGNC Symbol;Acc:HGNC:16993]				
63893	UBE2O	ubiquitin conjugating enzyme E2 O [Source:HGNC Symbol;Acc:HGNC:29554]				
8916	HERC3	HECT and RLD domain containing E3 ubiquitin protein ligase 3 [Source:HGNC Symbol;Acc:HGNC:4876]				
10385	BTN2A2	butyrophilin subfamily 2 member A2 [Source:HGNC Symbol;Acc:HGNC:1137]				
11118	BTN3A2	butyrophilin subfamily 3 member A2 [Source:HGNC Symbol;Acc:HGNC:1139]				
79908	BTNL8	butyrophilin like 8 [Source:HGNC Symbol;Acc:HGNC:26131]				
221687	RNF182	ring finger protein 182 [Source:HGNC Symbol;Acc:HGNC:28522]				
55008	HERC6	HECT and RLD domain containing E3 ubiquitin protein ligase family member 6 [Source:HGNC Symbol;Acc:HGNC:26072]				
55905	RNF114	ring finger protein 114 [Source:HGNC Symbol;Acc:HGNC:13094]				
51752	ERAP1	endoplasmic reticulum aminopeptidase 1 [Source:HGNC Symbol;Acc:HGNC:18173]				
64167	ERAP2	endoplasmic reticulum aminopeptidase 2 [Source:HGNC Symbol;Acc:HGNC:29499]				
23129	PLXND1	plexin D1 [Source:HGNC Symbol;Acc:HGNC:9107]				
5361	PLXNA1	plexin A1 [Source:HGNC Symbol;Acc:HGNC:9099]				
222663	SCUBE3	signal peptide, CUB domain and EGF like domain containing 3 [Source:HGNC Symbol;Acc:HGNC:13655]				
4017	LOXL2	lysyl oxidase like 2 [Source:HGNC Symbol;Acc:HGNC:6666]				
8692	HYAL2	hyaluronidase 2 [Source:HGNC Symbol;Acc:HGNC:5321]				
2258	FGF13	fibroblast growth factor 13 [Source:HGNC Symbol;Acc:HGNC:3670]				
80310	PDGFD	platelet derived growth factor D [Source:HGNC Symbol;Acc:HGNC:30620]				
83888	FGFBP2	fibroblast growth factor binding protein 2 [Source:HGNC Symbol;Acc:HGNC:29451]				
2487	FRZB	frizzled related protein [Source:HGNC Symbol;Acc:HGNC:3959]				
387911	C1QTNF9B	C1q and TNF related 9B [Source:HGNC Symbol;Acc:HGNC:34072]				
306	ANXA3	annexin A3 [Source:HGNC Symbol;Acc:HGNC:541]				
23436	CELA3B	chymotrypsin like elastase 3B [Source:HGNC Symbol;Acc:HGNC:15945]				
145264	SERPINA12	serpin family A member 12 [Source:HGNC Symbol;Acc:HGNC:18359]				
3026	HABP2	hyaluronan binding protein 2 [Source:HGNC Symbol;Acc:HGNC:4798]				
8530	CST7	cystatin F [Source:HGNC Symbol;Acc:HGNC:2479]				
89778	SERPINB11	serpin family B member 11 [Source:HGNC Symbol;Acc:HGNC:14221]				
9917	FAM20B	FAM20B glycosaminoglycan xylosylkinase [Source:HGNC Symbol;Acc:HGNC:23017]				
23654	PLXNB2	plexin B2 [Source:HGNC Symbol;Acc:HGNC:9104]				
114899	C1QTNF3	C1q and TNF related 3 [Source:HGNC Symbol;Acc:HGNC:14326]				
338872	C1QTNF9	C1q and TNF related 9 [Source:HGNC Symbol;Acc:HGNC:28732]				
387837	CLEC12B	C-type lectin domain family 12 member B [Source:HGNC Symbol;Acc:HGNC:31966]				
389376	SFTA2	surfactant associated 2 [Source:HGNC Symbol;Acc:HGNC:18386]				
390664	C1QTNF8	C1q and TNF related 8 [Source:HGNC Symbol;Acc:HGNC:31374]				
3957	LGALS2	galectin 2 [Source:HGNC Symbol;Acc:HGNC:6562]				
85329	LGALS12	galectin 12 [Source:HGNC Symbol;Acc:HGNC:15788]				
375790	AGRN	agrin [Source:HGNC Symbol;Acc:HGNC:329]				
1299	COL9A3	collagen type IX alpha 3 chain [Source:HGNC Symbol;Acc:HGNC:2219]				
1298	COL9A2	collagen type IX alpha 2 chain [Source:HGNC Symbol;Acc:HGNC:2218]				
59277	NTN4	netrin 4 [Source:HGNC Symbol;Acc:HGNC:13658]				
3909	LAMA3	laminin subunit alpha 3 [Source:HGNC Symbol;Acc:HGNC:6483]				
80781	COL18A1	collagen type XVIII alpha 1 chain [Source:HGNC Symbol;Acc:HGNC:2195]				
2006	ELN	elastin [Source:HGNC Symbol;Acc:HGNC:3327]				
4811	NID1	nidogen 1 [Source:HGNC Symbol;Acc:HGNC:7821]				
30008	EFEMP2	EGF containing fibulin extracellular matrix protein 2 [Source:HGNC Symbol;Acc:HGNC:3219]				
26577	PCOLCE2	procollagen C-endopeptidase enhancer 2 [Source:HGNC Symbol;Acc:HGNC:8739]				
1834	DSPP	dentin sialophosphoprotein [Source:HGNC Symbol;Acc:HGNC:3054]				
9353	SLIT2	slit guidance ligand 2 [Source:HGNC Symbol;Acc:HGNC:11086]				
7045	TGFBI	transforming growth factor beta induced [Source:HGNC Symbol;Acc:HGNC:11771]				
7783	ZP2	zona pellucida glycoprotein 2 [Source:HGNC Symbol;Acc:HGNC:13188]				
1101	CHAD	chondroadherin [Source:HGNC Symbol;Acc:HGNC:1909]				
200403	VWA3B	von Willebrand factor A domain containing 3B [Source:HGNC Symbol;Acc:HGNC:28385]				
342035	GLDN	gliomedin [Source:HGNC Symbol;Acc:HGNC:29514]				
4013	VWA5A	von Willebrand factor A domain containing 5A [Source:HGNC Symbol;Acc:HGNC:6658]				
79625	NDNF	neuron derived neurotrophic factor [Source:HGNC Symbol;Acc:HGNC:26256]				
9806	SPOCK2	SPARC (osteonectin), cwcv and kazal like domains proteoglycan 2 [Source:HGNC Symbol;Acc:HGNC:13564]				
921	CD5	CD5 molecule [Source:HGNC Symbol;Acc:HGNC:1685]				
931	MS4A1	membrane spanning 4-domains A1 [Source:HGNC Symbol;Acc:HGNC:7315]				
2687	GGT5	gamma-glutamyltransferase 5 [Source:HGNC Symbol;Acc:HGNC:4260]				
27243	CHMP2A	charged multivesicular body protein 2A [Source:HGNC Symbol;Acc:HGNC:30216]				
128866	CHMP4B	charged multivesicular body protein 4B [Source:HGNC Symbol;Acc:HGNC:16171]				
84313	VPS25	vacuolar protein sorting 25 homolog [Source:HGNC Symbol;Acc:HGNC:28122]				
2023	ENO1	enolase 1 [Source:HGNC Symbol;Acc:HGNC:3350]				
5230	PGK 1.00	phosphoglycerate kinase 1 [Source:HGNC Symbol;Acc:HGNC:8896]				
9332	CD163	CD163 molecule [Source:HGNC Symbol;Acc:HGNC:1631]				
8819	SAP30	Sin3A associated protein 30 [Source:HGNC Symbol;Acc:HGNC:10532]				
54625	PARP14	poly(ADP-ribose) polymerase family member 14 [Source:HGNC Symbol;Acc:HGNC:29232]				
81631	MAP1LC3B	microtubule associated protein 1 light chain 3 beta [Source:HGNC Symbol;Acc:HGNC:13352]				
9397	NMT2	N-myristoyltransferase 2 [Source:HGNC Symbol;Acc:HGNC:7858]				
2984	GUCY2C	guanylate cyclase 2C [Source:HGNC Symbol;Acc:HGNC:4688]				
3843	IPO5	importin 5 [Source:HGNC Symbol;Acc:HGNC:6402]				
10226	PLIN3	perilipin 3 [Source:HGNC Symbol;Acc:HGNC:16893]				
23344	ESYT1	extended synaptotagmin 1 [Source:HGNC Symbol;Acc:HGNC:29534]				
9497	SLC4A7	solute carrier family 4 member 7 [Source:HGNC Symbol;Acc:HGNC:11033]				
9644	SH3PXD2A	SH3 and PX domains 2A [Source:HGNC Symbol;Acc:HGNC:23664]				
103910	MYL12B	myosin light chain 12B [Source:HGNC Symbol;Acc:HGNC:29827]				
4660	PPP1R12B	protein phosphatase 1 regulatory subunit 12B [Source:HGNC Symbol;Acc:HGNC:7619]				
80776	B9D2	B9 domain containing 2 [Source:HGNC Symbol;Acc:HGNC:28636]				
10403	NDC80	NDC80 kinetochore complex component [Source:HGNC Symbol;Acc:HGNC:16909]				
54821	ERCC6L	ERCC excision repair 6 like, spindle assembly checkpoint helicase [Source:HGNC Symbol;Acc:HGNC:20794]				
55920	RCC2	regulator of chromosome condensation 2 [Source:HGNC Symbol;Acc:HGNC:30297]				
57405	SPC25	SPC25 component of NDC80 kinetochore complex [Source:HGNC Symbol;Acc:HGNC:24031]				
4638	MYLK	myosin light chain kinase [Source:HGNC Symbol;Acc:HGNC:7590]				
2017	CTTN	cortactin [Source:HGNC Symbol;Acc:HGNC:3338]				
29984	RHOD	ras homolog family member D [Source:HGNC Symbol;Acc:HGNC:670]				
537	ATP6AP1	ATPase H+ transporting accessory protein 1 [Source:HGNC Symbol;Acc:HGNC:868]				
10211	FLOT1	flotillin 1 [Source:HGNC Symbol;Acc:HGNC:3757]				
79658	ARHGAP10	Rho GTPase activating protein 10 [Source:HGNC Symbol;Acc:HGNC:26099]				
54874	FNBP1L	formin binding protein 1 like [Source:HGNC Symbol;Acc:HGNC:20851]				
22836	RHOBTB3	Rho related BTB domain containing 3 [Source:HGNC Symbol;Acc:HGNC:18757]				
4548	MTR	5-methyltetrahydrofolate-homocysteine methyltransferase [Source:HGNC Symbol;Acc:HGNC:7468]				
94240	EPSTI1	epithelial stromal interaction 1 [Source:HGNC Symbol;Acc:HGNC:16465]				
4580	MTX1	metaxin 1 [Source:HGNC Symbol;Acc:HGNC:7504]				
4698	NDUFA5	NADH:ubiquinone oxidoreductase subunit A5 [Source:HGNC Symbol;Acc:HGNC:7688]				
4162	MCAM	melanoma cell adhesion molecule [Source:HGNC Symbol;Acc:HGNC:6934]				
10409	BASP1	brain abundant membrane attached signal protein 1 [Source:HGNC Symbol;Acc:HGNC:957]				
26049	FAM169A	family with sequence similarity 169 member A [Source:HGNC Symbol;Acc:HGNC:29138]				
10395	DLC1	DLC1 Rho GTPase activating protein [Source:HGNC Symbol;Acc:HGNC:2897]				
395	ARHGAP6	Rho GTPase activating protein 6 [Source:HGNC Symbol;Acc:HGNC:676]				
25894	PLEKHG4	pleckstrin homology and RhoGEF domain containing G4 [Source:HGNC Symbol;Acc:HGNC:24501]				
51479	ANKFY1	ankyrin repeat and FYVE domain containing 1 [Source:HGNC Symbol;Acc:HGNC:20763]				
11135	CDC42EP1	CDC42 effector protein 1 [Source:HGNC Symbol;Acc:HGNC:17014]				
1123	CHN1	chimerin 1 [Source:HGNC Symbol;Acc:HGNC:1943]				
9710	GARRE1	granule associated Rac and RHOG effector 1 [Source:HGNC Symbol;Acc:HGNC:29016]				
157285	PRAG1	PEAK1 related, kinase-activating pseudokinase 1 [Source:HGNC Symbol;Acc:HGNC:25438]				
9886	RHOBTB1	Rho related BTB domain containing 1 [Source:HGNC Symbol;Acc:HGNC:18738]				
57473	ZNF512B	zinc finger protein 512B [Source:HGNC Symbol;Acc:HGNC:29212]				
66008	TRAK2	trafficking kinesin protein 2 [Source:HGNC Symbol;Acc:HGNC:13206]				
3002	GZMB	granzyme B [Source:HGNC Symbol;Acc:HGNC:4709]				
55825	PECR	peroxisomal trans-2-enoyl-CoA reductase [Source:HGNC Symbol;Acc:HGNC:18281]				
5551	PRF1	perforin 1 [Source:HGNC Symbol;Acc:HGNC:9360]				
5243	ABCB1	ATP binding cassette subfamily B member 1 [Source:HGNC Symbol;Acc:HGNC:40]				
840	CASP7	caspase 7 [Source:HGNC Symbol;Acc:HGNC:1508]				
30009	TBX21	T-box transcription factor 21 [Source:HGNC Symbol;Acc:HGNC:11599]				
8320	EOMES	eomesodermin [Source:HGNC Symbol;Acc:HGNC:3372]				
3001	GZMA	granzyme A [Source:HGNC Symbol;Acc:HGNC:4708]				
9374	PPT2	palmitoyl-protein thioesterase 2 [Source:HGNC Symbol;Acc:HGNC:9326]				
660	BMX	BMX non-receptor tyrosine kinase [Source:HGNC Symbol;Acc:HGNC:1079]				
8821	INPP4B	inositol polyphosphate-4-phosphatase type II B [Source:HGNC Symbol;Acc:HGNC:6075]				
5730	PTGDS	prostaglandin D2 synthase [Source:HGNC Symbol;Acc:HGNC:9592]				
400569	MED11	mediator complex subunit 11 [Source:HGNC Symbol;Acc:HGNC:32687]				
28234	SLCO1B3	solute carrier organic anion transporter family member 1B3 [Source:HGNC Symbol;Acc:HGNC:10961]				
9256	TSPOAP1	TSPO associated protein 1 [Source:HGNC Symbol;Acc:HGNC:16831]				
9663	LPIN2	lipin 2 [Source:HGNC Symbol;Acc:HGNC:14450]				
873	CBR1	carbonyl reductase 1 [Source:HGNC Symbol;Acc:HGNC:1548]				
1545	CYP1B1	cytochrome P450 family 1 subfamily B member 1 [Source:HGNC Symbol;Acc:HGNC:2597]				
211	ALAS1	5'-aminolevulinate synthase 1 [Source:HGNC Symbol;Acc:HGNC:396]				
29923	HILPDA	hypoxia inducible lipid droplet associated [Source:HGNC Symbol;Acc:HGNC:28859]				
80339	PNPLA3	patatin like phospholipase domain containing 3 [Source:HGNC Symbol;Acc:HGNC:18590]				
3248	HPGD	15-hydroxyprostaglandin dehydrogenase [Source:HGNC Symbol;Acc:HGNC:5154]				
5742	PTGS1	prostaglandin-endoperoxide synthase 1 [Source:HGNC Symbol;Acc:HGNC:9604]				
5095	PCCA	propionyl-CoA carboxylase subunit alpha [Source:HGNC Symbol;Acc:HGNC:8653]				
239	ALOX12	arachidonate 12-lipoxygenase, 12S type [Source:HGNC Symbol;Acc:HGNC:429]				
84129	ACAD11	acyl-CoA dehydrogenase family member 11 [Source:HGNC Symbol;Acc:HGNC:30211]				
641371	ACOT1	acyl-CoA thioesterase 1 [Source:HGNC Symbol;Acc:HGNC:33128]				
284486	THEM5	thioesterase superfamily member 5 [Source:HGNC Symbol;Acc:HGNC:26755]				
55856	ACOT13	acyl-CoA thioesterase 13 [Source:HGNC Symbol;Acc:HGNC:20999]				
80221	ACSF2	acyl-CoA synthetase family member 2 [Source:HGNC Symbol;Acc:HGNC:26101]				
84320	ACBD6	acyl-CoA binding domain containing 6 [Source:HGNC Symbol;Acc:HGNC:23339]				
2053	EPHX2	epoxide hydrolase 2 [Source:HGNC Symbol;Acc:HGNC:3402]				
23305	ACSL6	acyl-CoA synthetase long chain family member 6 [Source:HGNC Symbol;Acc:HGNC:16496]				
54677	CROT	carnitine O-octanoyltransferase [Source:HGNC Symbol;Acc:HGNC:2366]				
79993	ELOVL7	ELOVL fatty acid elongase 7 [Source:HGNC Symbol;Acc:HGNC:26292]				
55301	OLAH	oleoyl-ACP hydrolase [Source:HGNC Symbol;Acc:HGNC:25625]				
58526	MID1IP1	MID1 interacting protein 1 [Source:HGNC Symbol;Acc:HGNC:20715]				
1645	AKR1C1	aldo-keto reductase family 1 member C1 [Source:HGNC Symbol;Acc:HGNC:384]				
1588	CYP19A1	cytochrome P450 family 19 subfamily A member 1 [Source:HGNC Symbol;Acc:HGNC:2594]				
706	TSPO	translocator protein [Source:HGNC Symbol;Acc:HGNC:1158]				
56623	INPP5E	inositol polyphosphate-5-phosphatase E [Source:HGNC Symbol;Acc:HGNC:21474]				
5287	PIK3C2B	phosphatidylinositol-4-phosphate 3-kinase catalytic subunit type 2 beta [Source:HGNC Symbol;Acc:HGNC:8972]				
151056	PLB1	phospholipase B1 [Source:HGNC Symbol;Acc:HGNC:30041]				
85465	SELENOI	selenoprotein I [Source:HGNC Symbol;Acc:HGNC:29361]				
23171	GPD1L	glycerol-3-phosphate dehydrogenase 1 like [Source:HGNC Symbol;Acc:HGNC:28956]				
10162	LPCAT3	lysophosphatidylcholine acyltransferase 3 [Source:HGNC Symbol;Acc:HGNC:30244]				

55326	AGPAT5	1-acylglycerol-3-phosphate O-acyltransferase 5 [Source:HGNC Symbol;Acc:HGNC:20886]
23259	DDHD2	DDHD domain containing 2 [Source:HGNC Symbol;Acc:HGNC:29106]
374986	MIGA1	mitoguardin 1 [Source:HGNC Symbol;Acc:HGNC:24741]
57110	PLAAT1	phospholipase A and acyltransferase 1 [Source:HGNC Symbol;Acc:HGNC:14922]
5920	PLAAT4	phospholipase A and acyltransferase 4 [Source:HGNC Symbol;Acc:HGNC:9869]
9489	PGS1	phosphatidylglycerophosphate synthase 1 [Source:HGNC Symbol;Acc:HGNC:30029]
114879	OSBPL5	oxysterol binding protein like 5 [Source:HGNC Symbol;Acc:HGNC:16392]
57605	PITPNM2	phosphatidylinositol transfer protein membrane associated 2 [Source:HGNC Symbol;Acc:HGNC:21044]
59338	PLEKHA1	pleckstrin homology domain containing A1 [Source:HGNC Symbol;Acc:HGNC:14335]
54477	PLEKHA5	pleckstrin homology domain containing A5 [Source:HGNC Symbol;Acc:HGNC:30036]
84725	PLEKHA8	pleckstrin homology domain containing A8 [Source:HGNC Symbol;Acc:HGNC:30037]
79626	TNFAIP8L2	TNF alpha induced protein 8 like 2 [Source:HGNC Symbol;Acc:HGNC:26277]
51302	CYP39A1	cytochrome P450 family 39 subfamily A member 1 [Source:HGNC Symbol;Acc:HGNC:17449]
130367	SGPP2	sphingosine-1-phosphate phosphatase 2 [Source:HGNC Symbol;Acc:HGNC:19953]
166929	SGMS2	sphingomyelin synthase 2 [Source:HGNC Symbol;Acc:HGNC:28395]
7357	UGCG	UDP-glucose ceramide glucosyltransferase [Source:HGNC Symbol;Acc:HGNC:12524]
51099	ABHD5	abhydrolase domain containing 5, lysophosphatidic acid acyltransferase [Source:HGNC Symbol;Acc:HGNC:21396]
55711	FAR2	fatty acyl-CoA reductase 2 [Source:HGNC Symbol;Acc:HGNC:25531]
8613	PLPP3	phospholipid phosphatase 3 [Source:HGNC Symbol;Acc:HGNC:9229]
79603	CERS4	ceramide synthase 4 [Source:HGNC Symbol;Acc:HGNC:23747]
80270	HSD3B7	hydroxy-delta-5-steroid dehydrogenase, 3 beta- and steroid delta-isomerase 7 [Source:HGNC Symbol;Acc:HGNC:18324]
55512	SMPD3	sphingomyelin phosphodiesterase 3 [Source:HGNC Symbol;Acc:HGNC:14240]
2169	FABP2	fatty acid binding protein 2 [Source:HGNC Symbol;Acc:HGNC:3556]
114881	OSBPL7	oxysterol binding protein like 7 [Source:HGNC Symbol;Acc:HGNC:16387]
6309	SC5D	sterol-C5-desaturase [Source:HGNC Symbol;Acc:HGNC:10547]
622	BDH1	3-hydroxybutyrate dehydrogenase 1 [Source:HGNC Symbol;Acc:HGNC:1027]
29841	GRHL1	grainyhead like transcription factor 1 [Source:HGNC Symbol;Acc:HGNC:17923]
57379	AICDA	activation induced cytidine deaminase [Source:HGNC Symbol;Acc:HGNC:13203]
2035	EPB41	erythrocyte membrane protein band 4.1 [Source:HGNC Symbol;Acc:HGNC:3377]
3315	HSPB1	heat shock protein family B (small) member 1 [Source:HGNC Symbol;Acc:HGNC:5246]
321	APBA2	amyloid beta precursor protein binding family A member 2 [Source:HGNC Symbol;Acc:HGNC:579]
9133	CCNB2	cyclin B2 [Source:HGNC Symbol;Acc:HGNC:1580]
8870	IER3	immediate early response 3 [Source:HGNC Symbol;Acc:HGNC:5392]
55858	TMEM165	transmembrane protein 165 [Source:HGNC Symbol;Acc:HGNC:30760]
1960	EGR3	early growth response 3 [Source:HGNC Symbol;Acc:HGNC:3240]
5796	PTPRK	protein tyrosine phosphatase receptor type K [Source:HGNC Symbol;Acc:HGNC:9674]
824	CAPN2	calpain 2 [Source:HGNC Symbol;Acc:HGNC:1479]
6541	SLC7A1	solute carrier family 7 member 1 [Source:HGNC Symbol;Acc:HGNC:11057]
2113	ETS1	ETS proto-oncogene 1, transcription factor [Source:HGNC Symbol;Acc:HGNC:3488]
5226	PGD	phosphogluconate dehydrogenase [Source:HGNC Symbol;Acc:HGNC:8891]
2597	GAPDH	glyceraldehyde-3-phosphate dehydrogenase [Source:HGNC Symbol;Acc:HGNC:4141]
7086	TKT	transketolase [Source:HGNC Symbol;Acc:HGNC:11834]
8569	MKNK1	MAPK interacting serine/threonine kinase 1 [Source:HGNC Symbol;Acc:HGNC:7110]
9365	KL	klotho [Source:HGNC Symbol;Acc:HGNC:6344]
9047	SH2D2A	SH2 domain containing 2A [Source:HGNC Symbol;Acc:HGNC:10821]
3939	LDHA	lactate dehydrogenase A [Source:HGNC Symbol;Acc:HGNC:6535]
4673	NAP1L1	nucleosome assembly protein 1 like 1 [Source:HGNC Symbol;Acc:HGNC:7637]
7111	TMOD1	tropomodulin 1 [Source:HGNC Symbol;Acc:HGNC:11871]
52	ACP1	acid phosphatase 1 [Source:HGNC Symbol;Acc:HGNC:122]
154796	AMOT	angiomotin [Source:HGNC Symbol;Acc:HGNC:17810]
1267	CNP	2',3'-cyclic nucleotide 3' phosphodiesterase [Source:HGNC Symbol;Acc:HGNC:2158]
1192	CLIC1	chloride intracellular channel 1 [Source:HGNC Symbol;Acc:HGNC:2062]
1397	CRIP2	cysteine rich protein 2 [Source:HGNC Symbol;Acc:HGNC:2361]
357	SHROOM2	shroom family member 2 [Source:HGNC Symbol;Acc:HGNC:630]
51278	IER5	immediate early response 5 [Source:HGNC Symbol;Acc:HGNC:5393]
51309	ARMCX1	armadillo repeat containing X-linked 1 [Source:HGNC Symbol;Acc:HGNC:18073]
91624	NEXN	nexilin F-actin binding protein [Source:HGNC Symbol;Acc:HGNC:29557]
3691	ITGB4	integrin subunit beta 4 [Source:HGNC Symbol;Acc:HGNC:6158]
3679	ITGA7	integrin subunit alpha 7 [Source:HGNC Symbol;Acc:HGNC:6143]
7177	TPSAB1	tryptase alpha/beta 1 [Source:HGNC Symbol;Acc:HGNC:12019]
726	CAPN5	calpain 5 [Source:HGNC Symbol;Acc:HGNC:1482]
92291	CAPN13	calpain 13 [Source:HGNC Symbol;Acc:HGNC:16663]
83464	APH1B	aph-1 homolog B, gamma-secretase subunit [Source:HGNC Symbol;Acc:HGNC:24080]
785	CACNB4	calcium voltage-gated channel auxiliary subunit beta 4 [Source:HGNC Symbol;Acc:HGNC:1404]
1741	DLG3	discs large MAGUK scaffold protein 3 [Source:HGNC Symbol;Acc:HGNC:2902]
2242	FES	FES proto-oncogene, tyrosine kinase [Source:HGNC Symbol;Acc:HGNC:3657]
5080	PAX6	paired box 6 [Source:HGNC Symbol;Acc:HGNC:8620]
8848	TSC22D1	TSC22 domain family member 1 [Source:HGNC Symbol;Acc:HGNC:16826]
1050	CEBPA	CCAAT enhancer binding protein alpha [Source:HGNC Symbol;Acc:HGNC:1833]
2043	EPHA4	EPH receptor A4 [Source:HGNC Symbol;Acc:HGNC:3388]
3983	ABLIM1	actin binding LIM protein 1 [Source:HGNC Symbol;Acc:HGNC:78]
22885	ABLIM3	actin binding LIM protein family member 3 [Source:HGNC Symbol;Acc:HGNC:29132]
1826	DSCAM	DS cell adhesion molecule [Source:HGNC Symbol;Acc:HGNC:3039]
1947	EFNB1	ephrin B1 [Source:HGNC Symbol;Acc:HGNC:3226]
9355	LHX2	LIM homeobox 2 [Source:HGNC Symbol;Acc:HGNC:6594]
5376	PMP22	peripheral myelin protein 22 [Source:HGNC Symbol;Acc:HGNC:9118]
100133941	CD24	CD24 molecule [Source:HGNC Symbol;Acc:HGNC:1645]
9260	PDLIM7	PDZ and LIM domain 7 [Source:HGNC Symbol;Acc:HGNC:22958]
23708	GSPT2	G1 to S phase transition 2 [Source:HGNC Symbol;Acc:HGNC:4622]
2935	GSPT1	G1 to S phase transition 1 [Source:HGNC Symbol;Acc:HGNC:4621]
65110	UPF3A	UPF3A regulator of nonsense mediated mRNA decay [Source:HGNC Symbol;Acc:HGNC:20332]
5087	PBX1	PBX homeobox 1 [Source:HGNC Symbol;Acc:HGNC:8632]
4211	MEIS1	Meis homeobox 1 [Source:HGNC Symbol;Acc:HGNC:7000]
3212	HOXB2	homeobox B2 [Source:HGNC Symbol;Acc:HGNC:5113]
6736	SRY	sex determining region Y [Source:HGNC Symbol;Acc:HGNC:11311]
100271849	MEF2B	myocyte enhancer factor 2B [Source:HGNC Symbol;Acc:HGNC:6995]
130399	ACVR1C	activin A receptor type 1C [Source:HGNC Symbol;Acc:HGNC:18123]
4656	MYOG	myogenin [Source:HGNC Symbol;Acc:HGNC:7612]
79727	LIN28A	lin-28 homolog A [Source:HGNC Symbol;Acc:HGNC:15986]
1825	DSC3	desmocollin 3 [Source:HGNC Symbol;Acc:HGNC:3037]
342574	KRT27	keratin 27 [Source:HGNC Symbol;Acc:HGNC:30841]
4014	LORICRIN	loricrin cornified envelope precursor protein [Source:HGNC Symbol;Acc:HGNC:6663]
643418	LIPN	lipase family member N [Source:HGNC Symbol;Acc:HGNC:23452]
6705	SPRR2F	small proline rich protein 2F [Source:HGNC Symbol;Acc:HGNC:11266]
337963	KRTAP23-1	keratin associated protein 23-1 [Source:HGNC Symbol;Acc:HGNC:18928]
337968	KRTAP6-3	keratin associated protein 6-3 [Source:HGNC Symbol;Acc:HGNC:18933]
6622	SNCA	synuclein alpha [Source:HGNC Symbol;Acc:HGNC:11138]
23062	GGA2	golgi associated, gamma adaptin ear containing, ARF binding protein 2 [Source:HGNC Symbol;Acc:HGNC:16064]
10098	TSPAN5	tetraspanin 5 [Source:HGNC Symbol;Acc:HGNC:17753]
54959	ODAM	odontogenic, ameloblast associated [Source:HGNC Symbol;Acc:HGNC:26043]
6507	SLC1A3	solute carrier family 1 member 3 [Source:HGNC Symbol;Acc:HGNC:10941]
1244	ABCC2	ATP binding cassette subfamily C member 2 [Source:HGNC Symbol;Acc:HGNC:53]
1831	TSC22D3	TSC22 domain family member 3 [Source:HGNC Symbol;Acc:HGNC:3051]
200010	SLC5A9	solute carrier family 5 member 9 [Source:HGNC Symbol;Acc:HGNC:22146]
66035	SLC2A11	solute carrier family 2 member 11 [Source:HGNC Symbol;Acc:HGNC:14239]
3949	LDLR	low density lipoprotein receptor [Source:HGNC Symbol;Acc:HGNC:6547]
26119	LDLRAP1	low density lipoprotein receptor adaptor protein 1 [Source:HGNC Symbol;Acc:HGNC:18640]
444	ASPH	aspartate beta-hydroxylase [Source:HGNC Symbol;Acc:HGNC:757]
2281	FKBP1B	FKBP prolyl isomerase 1B [Source:HGNC Symbol;Acc:HGNC:3712]
3988	LIPA	lipase A, lysosomal acid type [Source:HGNC Symbol;Acc:HGNC:6617]
255231	MCOLN2	mucolipin 2 [Source:HGNC Symbol;Acc:HGNC:13357]
53373	TPCN1	two pore segment channel 1 [Source:HGNC Symbol;Acc:HGNC:18182]
55129	ANO10	anoctamin 10 [Source:HGNC Symbol;Acc:HGNC:25519]
94015	TTYH2	tweety family member 2 [Source:HGNC Symbol;Acc:HGNC:13877]
3990	LIPC	lipase C, hepatic type [Source:HGNC Symbol;Acc:HGNC:6619]
57194	ATP10A	ATPase phospholipid transporting 10A (putative) [Source:HGNC Symbol;Acc:HGNC:13542]
123606	NIPA1	NIPA magnesium transporter 1 [Source:HGNC Symbol;Acc:HGNC:17043]
57552	NCEH1	neutral cholesterol ester hydrolase 1 [Source:HGNC Symbol;Acc:HGNC:29260]
225	ABCD2	ATP binding cassette subfamily D member 2 [Source:HGNC Symbol;Acc:HGNC:66]
23456	ABCB10	ATP binding cassette subfamily B member 10 [Source:HGNC Symbol;Acc:HGNC:41]
85320	ABCC11	ATP binding cassette subfamily C member 11 [Source:HGNC Symbol;Acc:HGNC:14639]
8671	SLC4A4	solute carrier family 4 member 4 [Source:HGNC Symbol;Acc:HGNC:11030]
10864	SLC22A7	solute carrier family 22 member 7 [Source:HGNC Symbol;Acc:HGNC:10971]
760	CA2	carbonic anhydrase 2 [Source:HGNC Symbol;Acc:HGNC:1373]
762	CA4	carbonic anhydrase 4 [Source:HGNC Symbol;Acc:HGNC:1375]
119	ADD2	adducin 2 [Source:HGNC Symbol;Acc:HGNC:244]
2039	DMTN	dematin actin binding protein [Source:HGNC Symbol;Acc:HGNC:3382]
56172	ANKH	ANKH inorganic pyrophosphate transport regulator [Source:HGNC Symbol;Acc:HGNC:15492]
57185	NIPAL3	NIPA like domain containing 3 [Source:HGNC Symbol;Acc:HGNC:25233]
83697	SLC4A9	solute carrier family 4 member 9 [Source:HGNC Symbol;Acc:HGNC:11035]
2030	SLC29A1	solute carrier family 29 member 1 (Augustine blood group) [Source:HGNC Symbol;Acc:HGNC:11003]
85414	SLC45A3	solute carrier family 45 member 3 [Source:HGNC Symbol;Acc:HGNC:8642]
79751	SLC25A22	solute carrier family 25 member 22 [Source:HGNC Symbol;Acc:HGNC:19954]
28231	SLCO4A1	solute carrier organic anion transporter family member 4A1 [Source:HGNC Symbol;Acc:HGNC:10953]
5002	SLC22A18	solute carrier family 22 member 18 [Source:HGNC Symbol;Acc:HGNC:10964]
84912	SLC35B4	solute carrier family 35 member B4 [Source:HGNC Symbol;Acc:HGNC:20584]
254428	SLC41A1	solute carrier family 41 member 1 [Source:HGNC Symbol;Acc:HGNC:19429]
6563	SLC14A1	solute carrier family 14 member 1 (Kidd blood group) [Source:HGNC Symbol;Acc:HGNC:10918]
48	ACO1	aconitase 1 [Source:HGNC Symbol;Acc:HGNC:117]
55013	MCUB	mitochondrial calcium uniporter dominant negative subunit beta [Source:HGNC Symbol;Acc:HGNC:26076]
9254	CACNA2D2	calcium voltage-gated channel auxiliary subunit alpha2delta 2 [Source:HGNC Symbol;Acc:HGNC:1400]
116444	GRIN3B	glutamate ionotropic receptor NMDA type subunit 3B [Source:HGNC Symbol;Acc:HGNC:16768]
4747	NEFL	neurofilament light [Source:HGNC Symbol;Acc:HGNC:7739]
8825	LIN7A	lin-7 homolog A, crumbs cell polarity complex component [Source:HGNC Symbol;Acc:HGNC:17787]
4900	NRGN	neurogranin [Source:HGNC Symbol;Acc:HGNC:8000]
2570	GABRR2	gamma-aminobutyric acid type A receptor subunit rho2 [Source:HGNC Symbol;Acc:HGNC:4091]
3761	KCNJ4	potassium inwardly rectifying channel subfamily J member 4 [Source:HGNC Symbol;Acc:HGNC:6265]
3765	KCNJ9	potassium inwardly rectifying channel subfamily J member 9 [Source:HGNC Symbol;Acc:HGNC:6270]
3773	KCNJ16	potassium inwardly rectifying channel subfamily J member 16 [Source:HGNC Symbol;Acc:HGNC:6262]
200909	HTR3D	5-hydroxytryptamine receptor 3D [Source:HGNC Symbol;Acc:HGNC:24004]
217	ALDH2	aldehyde dehydrogenase 2 family member [Source:HGNC Symbol;Acc:HGNC:404]
220074	LRTOMT	leucine rich transmembrane and O-methyltransferase domain containing [Source:HGNC Symbol;Acc:HGNC:25033]
7915	ALDH5A1	aldehyde dehydrogenase 5 family member A1 [Source:HGNC Symbol;Acc:HGNC:408]
6854	SYN2	synapsin II [Source:HGNC Symbol;Acc:HGNC:11495]
1031	CDKN2C	cyclin dependent kinase inhibitor 2C [Source:HGNC Symbol;Acc:HGNC:1789]
8631	SKAP1	src kinase associated phosphoprotein 1 [Source:HGNC Symbol;Acc:HGNC:15605]
994	CDC25B	cell division cycle 25B [Source:HGNC Symbol;Acc:HGNC:1726]
23136	EPB41L3	erythrocyte membrane protein band 4.1 like 3 [Source:HGNC Symbol;Acc:HGNC:3380]
3783	KCNN4	potassium calcium-activated channel subfamily N member 4 [Source:HGNC Symbol;Acc:HGNC:6293]
3737	KCNA2	potassium voltage-gated channel subfamily A member 2 [Source:HGNC Symbol;Acc:HGNC:6220]
23416	KCNH3	potassium voltage-gated channel subfamily H member 3 [Source:HGNC Symbol;Acc:HGNC:6252]
94030	LRRC4B	leucine rich repeat containing 4B [Source:HGNC Symbol;Acc:HGNC:25042]
3306	HSPA2	heat shock protein family A (Hsp70) member 2 [Source:HGNC Symbol;Acc:HGNC:5235]
9985	REC8	REC8 meiotic recombination protein [Source:HGNC Symbol;Acc:HGNC:16879]
23353	SUN1	Sad1 and UNC84 domain containing 1 [Source:HGNC Symbol;Acc:HGNC:18587]
644186	SYCE3	synaptonemal complex central element protein 3 [Source:HGNC Symbol;Acc:HGNC:35245]
3823	KLRC3	killer cell lectin like receptor C3 [Source:HGNC Symbol;Acc:HGNC:6376]
8302	KLRC4	killer cell lectin like receptor C4 [Source:HGNC Symbol;Acc:HGNC:6377]
27030	MLH3	mutL homolog 3 [Source:HGNC Symbol;Acc:HGNC:7128]
23626	SPO11	SPO11 initiator of meiotic double stranded breaks [Source:HGNC Symbol;Acc:HGNC:11250]
84057	MND1	meiotic nuclear divisions 1 [Source:HGNC Symbol;Acc:HGNC:24839]
117144	CATSPER1	cation channel sperm associated 1 [Source:HGNC Symbol;Acc:HGNC:17116]
117155	CATSPER2	cation channel sperm associated 2 [Source:HGNC Symbol;Acc:HGNC:18810]
120776	OR2D2	olfactory receptor family 2 subfamily D member 2 [Source:HGNC Symbol;Acc:HGNC:8244]
283297	OR10A4	olfactory receptor family 10 subfamily A member 4 [Source:HGNC Symbol;Acc:HGNC:15130]
266747	RGL4	ral guanine nucleotide dissociation stimulator like 4 [Source:HGNC Symbol;Acc:HGNC:31911]
29909	GPR171	G protein-coupled receptor 171 [Source:HGNC Symbol;Acc:HGNC:30057]
84636	GPR174	G protein-coupled receptor 174 [Source:HGNC Symbol;Acc:HGNC:30245]
4603	MYBL1	MYB proto-oncogene like 1 [Source:HGNC Symbol;Acc:HGNC:7547]
10526	IPO8	importin 8 [Source:HGNC Symbol;Acc:HGNC:9853]
143689	PIWIL4	piwi like RNA-mediated gene silencing 4 [Source:HGNC Symbol;Acc:HGNC:18444]
55124	PIWIL2	piwi like RNA-mediated gene silencing 2 [Source:HGNC Symbol;Acc:HGNC:17644]
29102	DROSHA	drosha ribonuclease III [Source:HGNC Symbol;Acc:HGNC:17904]
122402	TDRD9	tudor domain containing 9 [Source:HGNC Symbol;Acc:HGNC:20122]
7188	TRAF5	TNF receptor associated factor 5 [Source:HGNC Symbol;Acc:HGNC:12035]
2114	ETS2	ETS proto-oncogene 2, transcription factor [Source:HGNC Symbol;Acc:HGNC:3489]
3009	H1-5	H1.5 linker histone, cluster member [Source:HGNC Symbol;Acc:HGNC:4719]
10592	SMC2	structural maintenance of chromosomes 2 [Source:HGNC Symbol;Acc:HGNC:14011]
10133	OPTN	optineurin [Source:HGNC Symbol;Acc:HGNC:17142]
11339	OIP5	Opa interacting protein 5 [Source:HGNC Symbol;Acc:HGNC:20300]
84131	CEP78	centrosomal protein 78 [Source:HGNC Symbol;Acc:HGNC:25740]
54930	HAUS4	HAUS augmin like complex subunit 4 [Source:HGNC Symbol;Acc:HGNC:20163]
55755	CDK5RAP2	CDK5 regulatory subunit associated protein 2 [Source:HGNC Symbol;Acc:HGNC:18672]
23244	PDS5A	PDS5 cohesin associated factor A [Source:HGNC Symbol;Acc:HGNC:29088]
10714	POLD3	DNA polymerase delta 3, accessory subunit [Source:HGNC Symbol;Acc:HGNC:20932]
81620	CDT1	chromatin licensing and DNA replication factor 1 [Source:HGNC Symbol;Acc:HGNC:24576]
63943	FKBPL	FKBP prolyl isomerase like [Source:HGNC Symbol;Acc:HGNC:13949]
55505	NOP10	NOP10 ribonucleoprotein [Source:HGNC Symbol;Acc:HGNC:14378]
8518	ELP1	elongator complex protein 1 [Source:HGNC Symbol;Acc:HGNC:5959]
9913	SUPT7L	SPT7 like, STAGA complex subunit gamma [Source:HGNC Symbol;Acc:HGNC:30632]
84148	KAT8	lysine acetyltransferase 8 [Source:HGNC Symbol;Acc:HGNC:17933]
55257	MRGBP	MRG domain binding protein [Source:HGNC Symbol;Acc:HGNC:15866]
7167	TPI1	triosephosphate isomerase 1 [Source:HGNC Symbol;Acc:HGNC:12009]
22934	RPIA	ribose 5-phosphate isomerase A [Source:HGNC Symbol;Acc:HGNC:10297]
6448	SGSH	N-sulfoglucosamine sulfohydrolase [Source:HGNC Symbol;Acc:HGNC:10818]
2584	GALK1	galactokinase 1 [Source:HGNC Symbol;Acc:HGNC:4118]
2131	EXT1	exostosin glycosyltransferase 1 [Source:HGNC Symbol;Acc:HGNC:3512]
5208	PFKFB2	6-phosphofructo-2-kinase/fructose-2,6-biphosphatase 2 [Source:HGNC Symbol;Acc:HGNC:8873]
2026	ENO2	enolase 2 [Source:HGNC Symbol;Acc:HGNC:3353]
8705	B3GALT4	beta-1,3-galactosyltransferase 4 [Source:HGNC Symbol;Acc:HGNC:919]
8604	SLC25A12	solute carrier family 25 member 12 [Source:HGNC Symbol;Acc:HGNC:10982]
8707	B3GALT2	beta-1,3-galactosyltransferase 2 [Source:HGNC Symbol;Acc:HGNC:917]
2529	FUT7	fucosyltransferase 7 [Source:HGNC Symbol;Acc:HGNC:4018]
27087	B3GAT1	beta-1,3-glucuronyltransferase 1 [Source:HGNC Symbol;Acc:HGNC:921]
55790	CSGALNACT1	chondroitin sulfate N-acetylgalactosaminyltransferase 1 [Source:HGNC Symbol;Acc:HGNC:24290]

55454	CSGALNACT2	chondroitin sulfate N-acetylgalactosaminyltransferase 2 [Source:HGNC Symbol;Acc:HGNC:24292]
10090	UST	uronyl 2-sulfotransferase [Source:HGNC Symbol;Acc:HGNC:17223]
6652	SORD	sorbitol dehydrogenase [Source:HGNC Symbol;Acc:HGNC:11184]
8509	NDST2	N-deacetylase and N-sulfotransferase 2 [Source:HGNC Symbol;Acc:HGNC:7681]
23035	PHLPP2	PH domain and leucine rich repeat protein phosphatase 2 [Source:HGNC Symbol;Acc:HGNC:29149]
440193	CCDC88C	coiled-coil domain containing 88C [Source:HGNC Symbol;Acc:HGNC:19967]
166	TLE5	TLE family member 5, transcriptional modulator [Source:HGNC Symbol;Acc:HGNC:307]
152789	JAKMIP1	janus kinase and microtubule interacting protein 1 [Source:HGNC Symbol;Acc:HGNC:26460]
3148	HMGB2	high mobility group box 2 [Source:HGNC Symbol;Acc:HGNC:5000]
3975	LHX1	LIM homeobox 1 [Source:HGNC Symbol;Acc:HGNC:6593]
9924	PAN2	poly(A) specific ribonuclease subunit PAN2 [Source:HGNC Symbol;Acc:HGNC:20074]
57476	GRAMD1B	GRAM domain containing 1B [Source:HGNC Symbol;Acc:HGNC:29214]
59285	CACNG6	calcium voltage-gated channel auxiliary subunit gamma 6 [Source:HGNC Symbol;Acc:HGNC:13625]
27091	CACNG5	calcium voltage-gated channel auxiliary subunit gamma 5 [Source:HGNC Symbol;Acc:HGNC:1409]
22808	MRAS	muscle RAS oncogene homolog [Source:HGNC Symbol;Acc:HGNC:7227]
1843	DUSP1	dual specificity phosphatase 1 [Source:HGNC Symbol;Acc:HGNC:3064]
8491	MAP4K3	mitogen-activated protein kinase kinase kinase kinase 3 [Source:HGNC Symbol;Acc:HGNC:6865]
54538	ROBO4	roundabout guidance receptor 4 [Source:HGNC Symbol;Acc:HGNC:17985]
57475	PLEKHH1	pleckstrin homology, MyTH4 and FERM domain containing H1 [Source:HGNC Symbol;Acc:HGNC:17733]
6443	SGCB	sarcoglycan beta [Source:HGNC Symbol;Acc:HGNC:10806]
64854	USP46	ubiquitin specific peptidase 46 [Source:HGNC Symbol;Acc:HGNC:20075]
10808	HSPH1	heat shock protein family H (Hsp110) member 1 [Source:HGNC Symbol;Acc:HGNC:16969]
9531	BAG3	BAG cochaperone 3 [Source:HGNC Symbol;Acc:HGNC:939]
55466	DNAJA4	DnaJ heat shock protein family (Hsp40) member A4 [Source:HGNC Symbol;Acc:HGNC:14885]
23645	PPP1R15A	protein phosphatase 1 regulatory subunit 15A [Source:HGNC Symbol;Acc:HGNC:14375]
8994	LIMD1	LIM domains containing 1 [Source:HGNC Symbol;Acc:HGNC:6612]
162989	DEDD2	death effector domain containing 2 [Source:HGNC Symbol;Acc:HGNC:24450]
8131	NPRL3	NPR3 like, GATOR1 complex subunit [Source:HGNC Symbol;Acc:HGNC:14124]
7169	TPM2	tropomyosin 2 [Source:HGNC Symbol;Acc:HGNC:12011]
22806	IKZF3	IKAROS family zinc finger 3 [Source:HGNC Symbol;Acc:HGNC:13178]
10252	SPRY1	sprouty RTK signaling antagonist 1 [Source:HGNC Symbol;Acc:HGNC:11269]
4145	MATK	megakaryocyte-associated tyrosine kinase [Source:HGNC Symbol;Acc:HGNC:6906]
3249	HPN	hepsin [Source:HGNC Symbol;Acc:HGNC:5155]
171558	PTCRA	pre T cell antigen receptor alpha [Source:HGNC Symbol;Acc:HGNC:21290]
800	CALD1	caldesmon 1 [Source:HGNC Symbol;Acc:HGNC:1441]
140465	MYL6B	myosin light chain 6B [Source:HGNC Symbol;Acc:HGNC:29823]
11215	AKAP11	A-kinase anchoring protein 11 [Source:HGNC Symbol;Acc:HGNC:369]
2729	GCLC	glutamate-cysteine ligase catalytic subunit [Source:HGNC Symbol;Acc:HGNC:4311]
7049	TGFBR3	transforming growth factor beta receptor 3 [Source:HGNC Symbol;Acc:HGNC:11774]
29103	DNAJC15	DnaJ heat shock protein family (Hsp40) member C15 [Source:HGNC Symbol;Acc:HGNC:20325]
8436	CAVIN2	caveolae associated protein 2 [Source:HGNC Symbol;Acc:HGNC:10690]
92086	GGTLC1	gamma-glutamyltransferase light chain 1 [Source:HGNC Symbol;Acc:HGNC:16437]
114112	TXNRD3	thioredoxin reductase 3 [Source:HGNC Symbol;Acc:HGNC:20667]
874	CBR3	carbonyl reductase 3 [Source:HGNC Symbol;Acc:HGNC:1549]
117283	IP6K3	inositol hexakisphosphate kinase 3 [Source:HGNC Symbol;Acc:HGNC:17269]
79621	RNASEH2B	ribonuclease H2 subunit B [Source:HGNC Symbol;Acc:HGNC:25671]
84153	RNASEH2C	ribonuclease H2 subunit C [Source:HGNC Symbol;Acc:HGNC:24116]
875	CBS	cystathionine beta-synthase [Source:HGNC Symbol;Acc:HGNC:1550]
8853	ASAP2	ArfGAP with SH3 domain, ankyrin repeat and PH domain 2 [Source:HGNC Symbol;Acc:HGNC:2721]
126364	LRRC25	leucine rich repeat containing 25 [Source:HGNC Symbol;Acc:HGNC:29806]
22997	IGSF9B	immunoglobulin superfamily member 9B [Source:HGNC Symbol;Acc:HGNC:32326]
8609	KLF7	Kruppel like factor 7 [Source:HGNC Symbol;Acc:HGNC:6350]
4835	NQO2	N-ribosyldihydronicotinamide:quinone reductase 2 [Source:HGNC Symbol;Acc:HGNC:7856]
54700	RRN3	RRN3 homolog, RNA polymerase I transcription factor [Source:HGNC Symbol;Acc:HGNC:30346]
59352	LGR6	leucine rich repeat containing G protein-coupled receptor 6 [Source:HGNC Symbol;Acc:HGNC:19719]
50964	SOST	sclerostin [Source:HGNC Symbol;Acc:HGNC:13771]
950	SCARB2	scavenger receptor class B member 2 [Source:HGNC Symbol;Acc:HGNC:1665]
1777	DNASE2	deoxyribonuclease 2, lysosomal [Source:HGNC Symbol;Acc:HGNC:2960]
9829	DNAJC6	DnaJ heat shock protein family (Hsp40) member C6 [Source:HGNC Symbol;Acc:HGNC:15469]
22905	EPN2	epsin 2 [Source:HGNC Symbol;Acc:HGNC:18639]
9882	TBC1D4	TBC1 domain family member 4 [Source:HGNC Symbol;Acc:HGNC:19165]
273	AMPH	amphiphysin [Source:HGNC Symbol;Acc:HGNC:471]
3267	AGFG1	ArfGAP with FG repeats 1 [Source:HGNC Symbol;Acc:HGNC:5175]
29993	PACSIN1	protein kinase C and casein kinase substrate in neurons 1 [Source:HGNC Symbol;Acc:HGNC:8570]
2647	BLOC1S1	biogenesis of lysosomal organelles complex 1 subunit 1 [Source:HGNC Symbol;Acc:HGNC:4200]
54453	RIN2	Ras and Rab interactor 2 [Source:HGNC Symbol;Acc:HGNC:18750]
80271	ITPKC	inositol-trisphosphate 3-kinase C [Source:HGNC Symbol;Acc:HGNC:14897]
9538	EI24	EI24 autophagy associated transmembrane protein [Source:HGNC Symbol;Acc:HGNC:13276]
64782	AEN	apoptosis enhancing nuclease [Source:HGNC Symbol;Acc:HGNC:25722]
196513	DCP1B	decapping mRNA 1B [Source:HGNC Symbol;Acc:HGNC:24451]
91947	ARRDC4	arrestin domain containing 4 [Source:HGNC Symbol;Acc:HGNC:28087]
93408	MYL10	myosin light chain 10 [Source:HGNC Symbol;Acc:HGNC:29825]
326625	MMAB	metabolism of cobalamin associated B [Source:HGNC Symbol;Acc:HGNC:19331]
26873	OPLAH	5-oxoprolinase, ATP-hydrolysing [Source:HGNC Symbol;Acc:HGNC:8149]
56922	MCCC1	methylcrotonoyl-CoA carboxylase 1 [Source:HGNC Symbol;Acc:HGNC:6936]
2235	FECH	ferrochelatase [Source:HGNC Symbol;Acc:HGNC:3647]
7538	ZFP36	ZFP36 ring finger protein [Source:HGNC Symbol;Acc:HGNC:12862]
1618	DAZL	deleted in azoospermia like [Source:HGNC Symbol;Acc:HGNC:2685]
343171	OR2W3	olfactory receptor family 2 subfamily W member 3 [Source:HGNC Symbol;Acc:HGNC:15021]
55081	IFT57	intraflagellar transport 57 [Source:HGNC Symbol;Acc:HGNC:17367]
23059	CLUAP1	clusterin associated protein 1 [Source:HGNC Symbol;Acc:HGNC:19009]
79809	TTC21B	tetratricopeptide repeat domain 21B [Source:HGNC Symbol;Acc:HGNC:25660]
55212	BBS7	Bardet-Biedl syndrome 7 [Source:HGNC Symbol;Acc:HGNC:18758]
54585	LZTFL1	leucine zipper transcription factor like 1 [Source:HGNC Symbol;Acc:HGNC:6741]
9727	RAB11FIP3	RAB11 family interacting protein 3 [Source:HGNC Symbol;Acc:HGNC:17224]
90410	IFT20	intraflagellar transport 20 [Source:HGNC Symbol;Acc:HGNC:30989]
200132	TCTEX1D1	Tctex1 domain containing 1 [Source:HGNC Symbol;Acc:HGNC:26882]
23408	SIRT5	sirtuin 5 [Source:HGNC Symbol;Acc:HGNC:14933]
9249	DHRS3	dehydrogenase/reductase 3 [Source:HGNC Symbol;Acc:HGNC:17693]
10170	DHRS9	dehydrogenase/reductase 9 [Source:HGNC Symbol;Acc:HGNC:16888]
10964	IFI44L	interferon induced protein 44 like [Source:HGNC Symbol;Acc:HGNC:17817]
23541	SEC14L2	SEC14 like lipid binding 2 [Source:HGNC Symbol;Acc:HGNC:10699]
3250	HPR	haptoglobin-related protein [Source:HGNC Symbol;Acc:HGNC:5156]
3067	HDC	histidine decarboxylase [Source:HGNC Symbol;Acc:HGNC:4855]
8030	CCDC6	coiled-coil domain containing 6 [Source:HGNC Symbol;Acc:HGNC:18782]
285440	CYP4V2	cytochrome P450 family 4 subfamily V member 2 [Source:HGNC Symbol;Acc:HGNC:23198]
29967	LRP12	LDL receptor related protein 12 [Source:HGNC Symbol;Acc:HGNC:31708]
2986	GUCY2F	guanylate cyclase 2F, retinal [Source:HGNC Symbol;Acc:HGNC:4691]
5957	RCVRN	recoverin [Source:HGNC Symbol;Acc:HGNC:9937]
55183	RIF1	replication timing regulatory factor 1 [Source:HGNC Symbol;Acc:HGNC:23207]
6289	SAA2	serum amyloid A2 [Source:HGNC Symbol;Acc:HGNC:10514]
51734	MSRB1	methionine sulfoxide reductase B1 [Source:HGNC Symbol;Acc:HGNC:14133]
221002	RASGEF1A	RasGEF domain family member 1A [Source:HGNC Symbol;Acc:HGNC:24246]
4953	ODC1	ornithine decarboxylase 1 [Source:HGNC Symbol;Acc:HGNC:8109]
56911	MAP3K7CL	MAP3K7 C-terminal like [Source:HGNC Symbol;Acc:HGNC:16457]
30818	KCNIP3	potassium voltage-gated channel interacting protein 3 [Source:HGNC Symbol;Acc:HGNC:15523]
27018	BEX3	brain expressed X-linked 3 [Source:HGNC Symbol;Acc:HGNC:13388]
27347	STK39	serine/threonine kinase 39 [Source:HGNC Symbol;Acc:HGNC:17717]
23630	KCNE5	potassium voltage-gated channel subfamily E regulatory subunit 5 [Source:HGNC Symbol;Acc:HGNC:6241]
27134	TJP3	tight junction protein 3 [Source:HGNC Symbol;Acc:HGNC:11829]
3993	LLGL2	LLGL scribble cell polarity complex component 2 [Source:HGNC Symbol;Acc:HGNC:6629]
5447	POR	cytochrome p450 oxidoreductase [Source:HGNC Symbol;Acc:HGNC:9208]
4837	NNMT	nicotinamide N-methyltransferase [Source:HGNC Symbol;Acc:HGNC:7861]
219	ALDH1B1	aldehyde dehydrogenase 1 family member B1 [Source:HGNC Symbol;Acc:HGNC:407]
54498	SMOX	spermine oxidase [Source:HGNC Symbol;Acc:HGNC:15862]
670	BPHL	biphenyl hydrolase like [Source:HGNC Symbol;Acc:HGNC:1094]
91703	ACY3	aminoacylase 3 [Source:HGNC Symbol;Acc:HGNC:24104]
10941	UGT2A1	UDP glucuronosyltransferase family 2 member A1 complex locus [Source:HGNC Symbol;Acc:HGNC:12542]
27284	SULT1B1	sulfotransferase family 1B member 1 [Source:HGNC Symbol;Acc:HGNC:17845]
55347	ABHD10	abhydrolase domain containing 10, depalmitoylase [Source:HGNC Symbol;Acc:HGNC:25656]
8120	AP3B2	adaptor related protein complex 3 subunit beta 2 [Source:HGNC Symbol;Acc:HGNC:567]
10239	AP3S2	adaptor related protein complex 3 subunit sigma 2 [Source:HGNC Symbol;Acc:HGNC:571]
10947	AP3M2	adaptor related protein complex 3 subunit mu 2 [Source:HGNC Symbol;Acc:HGNC:570]
27074	LAMP3	lysosomal associated membrane protein 3 [Source:HGNC Symbol;Acc:HGNC:14582]
122481	AK7	adenylate kinase 7 [Source:HGNC Symbol;Acc:HGNC:20091]
1406	CRX	cone-rod homeobox [Source:HGNC Symbol;Acc:HGNC:2383]
65250	CPLANE1	ciliogenesis and planar polarity effector 1 [Source:HGNC Symbol;Acc:HGNC:25801]
84314	TMEM107	transmembrane protein 107 [Source:HGNC Symbol;Acc:HGNC:28128]
4117	MAK	male germ cell associated kinase [Source:HGNC Symbol;Acc:HGNC:6816]
221421	RSPH9	radial spoke head component 9 [Source:HGNC Symbol;Acc:HGNC:21057]
25886	POC1A	POC1 centriolar protein A [Source:HGNC Symbol;Acc:HGNC:24488]
7287	TULP1	TUB like protein 1 [Source:HGNC Symbol;Acc:HGNC:12423]
83538	TTC25	tetratricopeptide repeat domain 25 [Source:HGNC Symbol;Acc:HGNC:25280]
84662	GLIS2	GLIS family zinc finger 2 [Source:HGNC Symbol;Acc:HGNC:29450]
85478	CCDC65	coiled-coil domain containing 65 [Source:HGNC Symbol;Acc:HGNC:29937]
8437	RASAL1	RAS protein activator like 1 [Source:HGNC Symbol;Acc:HGNC:9873]
7494	XBP1	X-box binding protein 1 [Source:HGNC Symbol;Acc:HGNC:12801]
4704	NDUFA9	NADH:ubiquinone oxidoreductase subunit A9 [Source:HGNC Symbol;Acc:HGNC:7693]
6391	SDHC	succinate dehydrogenase complex subunit C [Source:HGNC Symbol;Acc:HGNC:10682]
1346	COX7A1	cytochrome c oxidase subunit 7A1 [Source:HGNC Symbol;Acc:HGNC:2287]
22877	MLXIP	MLX interacting protein [Source:HGNC Symbol;Acc:HGNC:17055]
1633	DCK	deoxycytidine kinase [Source:HGNC Symbol;Acc:HGNC:2704]
593	BCKDHA	branched chain keto acid dehydrogenase E1 subunit alpha [Source:HGNC Symbol;Acc:HGNC:986]
10295	BCKDK	branched chain keto acid dehydrogenase kinase [Source:HGNC Symbol;Acc:HGNC:16902]
586	BCAT1	branched chain amino acid transaminase 1 [Source:HGNC Symbol;Acc:HGNC:976]
4329	ALDH6A1	aldehyde dehydrogenase 6 family member A1 [Source:HGNC Symbol;Acc:HGNC:7179]
152926	PPM1K	protein phosphatase, Mg2+/Mn2+ dependent 1K [Source:HGNC Symbol;Acc:HGNC:25415]
2999	GZMH	granzyme H [Source:HGNC Symbol;Acc:HGNC:4710]
10693	CCT6B	chaperonin containing TCP1 subunit 6B [Source:HGNC Symbol;Acc:HGNC:1621]
3732	CD82	CD82 molecule [Source:HGNC Symbol;Acc:HGNC:6210]
27010	TPK1	thiamin pyrophosphokinase 1 [Source:HGNC Symbol;Acc:HGNC:17358]
10588	MTHFS	methenyltetrahydrofolate synthetase [Source:HGNC Symbol;Acc:HGNC:7437]
5169	ENPP3	ectonucleotide pyrophosphatase/phosphodiesterase 3 [Source:HGNC Symbol;Acc:HGNC:3358]
23057	NMNAT2	nicotinamide nucleotide adenylyltransferase 2 [Source:HGNC Symbol;Acc:HGNC:16789]
25902	MTHFD1L	methylenetetrahydrofolate dehydrogenase (NADP+ dependent) 1 like [Source:HGNC Symbol;Acc:HGNC:21055]
9962	SLC23A2	solute carrier family 23 member 2 [Source:HGNC Symbol;Acc:HGNC:10973]
7404	UTY	ubiquitously transcribed tetratricopeptide repeat containing, Y-linked [Source:HGNC Symbol;Acc:HGNC:12638]
64744	SMAP2	small ArfGAP2 [Source:HGNC Symbol;Acc:HGNC:25082]
50624	CUZD1	CUB and zona pellucida like domains 1 [Source:HGNC Symbol;Acc:HGNC:17937]
64919	BCL11B	BAF chromatin remodeling complex subunit BCL11B [Source:HGNC Symbol;Acc:HGNC:13222]
10771	ZMYND11	zinc finger MYND-type containing 11 [Source:HGNC Symbol;Acc:HGNC:16966]
51513	ETV7	ETS variant transcription factor 7 [Source:HGNC Symbol;Acc:HGNC:18160]
56953	NT5M	5',3'-nucleotidase, mitochondrial [Source:HGNC Symbol;Acc:HGNC:15769]
84618	NT5C1A	5'-nucleotidase, cytosolic IA [Source:HGNC Symbol;Acc:HGNC:17819]
955	ENTPD6	ectonucleoside triphosphate diphosphohydrolase 6 [Source:HGNC Symbol;Acc:HGNC:3368]
11164	NUDT5	nudix hydrolase 5 [Source:HGNC Symbol;Acc:HGNC:8052]
2766	GMPR	guanosine monophosphate reductase [Source:HGNC Symbol;Acc:HGNC:4376]
203	AK1	adenylate kinase 1 [Source:HGNC Symbol;Acc:HGNC:361]
87178	PNPT1	polyribonucleotide nucleotidyltransferase 1 [Source:HGNC Symbol;Acc:HGNC:23166]
5149	PDE6H	phosphodiesterase 6H [Source:HGNC Symbol;Acc:HGNC:8790]
54963	UCKL1	uridine-cytidine kinase 1 like 1 [Source:HGNC Symbol;Acc:HGNC:15938]
51727	CMPK1	cytidine/uridine monophosphate kinase 1 [Source:HGNC Symbol;Acc:HGNC:18170]
57089	ENTPD7	ectonucleoside triphosphate diphosphohydrolase 7 [Source:HGNC Symbol;Acc:HGNC:19745]
79039	DDX54	DEAD-box helicase 54 [Source:HGNC Symbol;Acc:HGNC:20084]
3298	HSF2	heat shock transcription factor 2 [Source:HGNC Symbol;Acc:HGNC:5225]
11076	TPPP	tubulin polymerization promoting protein [Source:HGNC Symbol;Acc:HGNC:24164]
11326	VSIG4	V-set and immunoglobulin domain containing 4 [Source:HGNC Symbol;Acc:HGNC:17032]
4774	NFIA	nuclear factor I A [Source:HGNC Symbol;Acc:HGNC:7784]
6638	SNRPN	small nuclear ribonucleoprotein polypeptide N [Source:HGNC Symbol;Acc:HGNC:11164]
347686	SNORD64	small nucleolar RNA, C/D box 64 [Source:HGNC Symbol;Acc:HGNC:32725]
11183	MAP4K5	mitogen-activated protein kinase kinase kinase kinase 5 [Source:HGNC Symbol;Acc:HGNC:6867]
94234	FOXQ1	forkhead box Q1 [Source:HGNC Symbol;Acc:HGNC:20951]
64388	GREM2	gremlin 2, DAN family BMP antagonist [Source:HGNC Symbol;Acc:HGNC:17655]
23514	SPIDR	scaffold protein involved in DNA repair [Source:HGNC Symbol;Acc:HGNC:28971]
10459	MAD2L2	mitotic arrest deficient 2 like 2 [Source:HGNC Symbol;Acc:HGNC:6764]
55120	FANCL	FA complementation group L [Source:HGNC Symbol;Acc:HGNC:20748]
22909	FAN1	FANCD2 and FANCI associated nuclease 1 [Source:HGNC Symbol;Acc:HGNC:29170]
85015	USP45	ubiquitin specific peptidase 45 [Source:HGNC Symbol;Acc:HGNC:20080]
60487	TRMT11	tRNA methyltransferase 11 homolog [Source:HGNC Symbol;Acc:HGNC:21080]
969	CD69	CD69 molecule [Source:HGNC Symbol;Acc:HGNC:1694]
9706	ULK2	unc-51 like autophagy activating kinase 2 [Source:HGNC Symbol;Acc:HGNC:13480]
55332	DRAM1	DNA damage regulated autophagy modulator 1 [Source:HGNC Symbol;Acc:HGNC:25645]
5509	PPP1R3D	protein phosphatase 1 regulatory subunit 3D [Source:HGNC Symbol;Acc:HGNC:9294]
129607	CMPK2	cytidine/uridine monophosphate kinase 2 [Source:HGNC Symbol;Acc:HGNC:27015]
245711	SPDYA	speedy/RINGO cell cycle regulator family member A [Source:HGNC Symbol;Acc:HGNC:30613]
22883	CLSTN1	calsyntenin 1 [Source:HGNC Symbol;Acc:HGNC:17447]
2296	FOXC1	forkhead box C1 [Source:HGNC Symbol;Acc:HGNC:3800]
92960	PEX11G	peroxisomal biogenesis factor 11 gamma [Source:HGNC Symbol;Acc:HGNC:20208]
58472	SQOR	sulfide quinone oxidoreductase [Source:HGNC Symbol;Acc:HGNC:20390]
27232	GNMT	glycine N-methyltransferase [Source:HGNC Symbol;Acc:HGNC:4415]
29969	MDFIC	MyoD family inhibitor domain containing [Source:HGNC Symbol;Acc:HGNC:28870]
9289	ADGRG1	adhesion G protein-coupled receptor G1 [Source:HGNC Symbol;Acc:HGNC:4512]
29904	EEF2K	eukaryotic elongation factor 2 kinase [Source:HGNC Symbol;Acc:HGNC:24615]
5393	EXOSC9	exosome component 9 [Source:HGNC Symbol;Acc:HGNC:9137]
54512	EXOSC4	exosome component 4 [Source:HGNC Symbol;Acc:HGNC:18189]
11044	TENT4A	terminal nucleotidyltransferase 4A [Source:HGNC Symbol;Acc:HGNC:16705]
11157	LSM 6.00	LSM6 homolog, U6 small nuclear RNA and mRNA degradation associated [Source:HGNC Symbol;Acc:HGNC:17017]
200931	SLC51A	solute carrier family 51 subunit alpha [Source:HGNC Symbol;Acc:HGNC:29955]
11344	TWF2	twinfilin actin binding protein 2 [Source:HGNC Symbol;Acc:HGNC:9621]
222662	LHFPL5	LHFPL tetraspan subfamily member 5 [Source:HGNC Symbol;Acc:HGNC:21253]
53405	CLIC5	chloride intracellular channel 5 [Source:HGNC Symbol;Acc:HGNC:13517]
9478	CABP1	calcium binding protein 1 [Source:HGNC Symbol;Acc:HGNC:1384]
120065	OR5P2	olfactory receptor family 5 subfamily P member 2 [Source:HGNC Symbol;Acc:HGNC:14783]
122740	OR4K14	olfactory receptor family 4 subfamily K member 14 [Source:HGNC Symbol;Acc:HGNC:15352]
124538	OR4D2	olfactory receptor family 4 subfamily D member 2 [Source:HGNC Symbol;Acc:HGNC:8294]
138799	OR13C5	olfactory receptor family 13 subfamily C member 5 [Source:HGNC Symbol;Acc:HGNC:15100]

256144	OR4C3	olfactory receptor family 4 subfamily C member 3 [Source:HGNC Symbol;Acc:HGNC:14697]
26689	OR4D1	olfactory receptor family 4 subfamily D member 1 [Source:HGNC Symbol;Acc:HGNC:8293]
283159	OR8D1	olfactory receptor family 8 subfamily D member 1 [Source:HGNC Symbol;Acc:HGNC:8481]
390061	OR51Q1	olfactory receptor family 51 subfamily Q member 1 [Source:HGNC Symbol;Acc:HGNC:14851]
390648	OR4F6	olfactory receptor family 4 subfamily F member 6 [Source:HGNC Symbol;Acc:HGNC:15372]
401994	OR14I1	olfactory receptor family 14 subfamily I member 1 [Source:HGNC Symbol;Acc:HGNC:19575]
79345	OR51B2	olfactory receptor family 51 subfamily B member 2 [Source:HGNC Symbol;Acc:HGNC:14703]
81470	OR2G2	olfactory receptor family 2 subfamily G member 2 [Source:HGNC Symbol;Acc:HGNC:15007]
51308	REEP2	receptor accessory protein 2 [Source:HGNC Symbol;Acc:HGNC:17975]
64108	RTP4	receptor transporter protein 4 [Source:HGNC Symbol;Acc:HGNC:23992]
729759	OR4F29	olfactory receptor family 4 subfamily F member 29 [Source:HGNC Symbol;Acc:HGNC:31275]
10567	RABAC1	Rab acceptor 1 [Source:HGNC Symbol;Acc:HGNC:9794]
64092	SAMSN1	SAM domain, SH3 domain and nuclear localization signals 1 [Source:HGNC Symbol;Acc:HGNC:10528]
57414	RHBDD2	rhomboid domain containing 2 [Source:HGNC Symbol;Acc:HGNC:23082]
51056	LAP3	leucine aminopeptidase 3 [Source:HGNC Symbol;Acc:HGNC:18449]
25825	BACE2	beta-secretase 2 [Source:HGNC Symbol;Acc:HGNC:934]
6449	SGTA	small glutamine rich tetratricopeptide repeat containing alpha [Source:HGNC Symbol;Acc:HGNC:10819]
54206	ERRFI1	ERBB receptor feedback inhibitor 1 [Source:HGNC Symbol;Acc:HGNC:18185]
23607	CD2AP	CD2 associated protein [Source:HGNC Symbol;Acc:HGNC:14258]
11346	SYNPO	synaptopodin [Source:HGNC Symbol;Acc:HGNC:30672]
161176	SYNE3	spectrin repeat containing nuclear envelope family member 3 [Source:HGNC Symbol;Acc:HGNC:19861]
1407	CRY1	cryptochrome circadian regulator 1 [Source:HGNC Symbol;Acc:HGNC:2384]
687	KLF9	Kruppel like factor 9 [Source:HGNC Symbol;Acc:HGNC:1123]
5813	PURA	purine rich element binding protein A [Source:HGNC Symbol;Acc:HGNC:9701]
219743	TYSND1	trypsin like peroxisomal matrix peptidase 1 [Source:HGNC Symbol;Acc:HGNC:28531]
26519	TIMM10	translocase of inner mitochondrial membrane 10 [Source:HGNC Symbol;Acc:HGNC:11814]
79145	CHCHD7	coiled-coil-helix-coiled-coil-helix domain containing 7 [Source:HGNC Symbol;Acc:HGNC:28314]
79899	PRR5L	proline rich 5 like [Source:HGNC Symbol;Acc:HGNC:25878]
26228	STAP1	signal transducing adaptor family member 1 [Source:HGNC Symbol;Acc:HGNC:24133]
3620	IDO1	indoleamine 2,3-dioxygenase 1 [Source:HGNC Symbol;Acc:HGNC:6059]
3613	IMPA2	inositol monophosphatase 2 [Source:HGNC Symbol;Acc:HGNC:6051]
55437	STRADB	STE20 related adaptor beta [Source:HGNC Symbol;Acc:HGNC:13205]
107075116	MIR3142HG	MIR3142 host gene [Source:HGNC Symbol;Acc:HGNC:51944]
51474	LIMA1	LIM domain and actin binding 1 [Source:HGNC Symbol;Acc:HGNC:24636]
4118	MAL	mal, T cell differentiation protein [Source:HGNC Symbol;Acc:HGNC:6817]
55062	WIPI1	WD repeat domain, phosphoinositide interacting 1 [Source:HGNC Symbol;Acc:HGNC:25471]
55226	NAT10	N-acetyltransferase 10 [Source:HGNC Symbol;Acc:HGNC:29830]
10885	WDR3	WD repeat domain 3 [Source:HGNC Symbol;Acc:HGNC:12755]
55623	THUMPD1	THUMP domain containing 1 [Source:HGNC Symbol;Acc:HGNC:23807]
9724	UTP14C	UTP14C small subunit processome component [Source:HGNC Symbol;Acc:HGNC:20321]
27350	APOBEC3C	apolipoprotein B mRNA editing enzyme catalytic subunit 3C [Source:HGNC Symbol;Acc:HGNC:17353]
104	ADARB1	adenosine deaminase RNA specific B1 [Source:HGNC Symbol;Acc:HGNC:226]
79746	ECHDC3	enoyl-CoA hydratase domain containing 3 [Source:HGNC Symbol;Acc:HGNC:23489]
23564	DDAH2	dimethylarginine dimethylaminohydrolase 2 [Source:HGNC Symbol;Acc:HGNC:2716]
80155	NAA15	N-alpha-acetyltransferase 15, NatA auxiliary subunit [Source:HGNC Symbol;Acc:HGNC:30782]
57085	AGTRAP	angiotensin II receptor associated protein [Source:HGNC Symbol;Acc:HGNC:13539]
55859	BEX1	brain expressed X-linked 1 [Source:HGNC Symbol;Acc:HGNC:1036]
8662	EIF3B	eukaryotic translation initiation factor 3 subunit B [Source:HGNC Symbol;Acc:HGNC:3280]
8669	EIF3J	eukaryotic translation initiation factor 3 subunit J [Source:HGNC Symbol;Acc:HGNC:3270]
1803	DPP4	dipeptidyl peptidase 4 [Source:HGNC Symbol;Acc:HGNC:3009]
56341	PRMT8	protein arginine methyltransferase 8 [Source:HGNC Symbol;Acc:HGNC:5188]
10873	ME3	malic enzyme 3 [Source:HGNC Symbol;Acc:HGNC:6985]
23119	HIC2	HIC ZBTB transcriptional repressor 2 [Source:HGNC Symbol;Acc:HGNC:18595]
421	ARVCF	ARVCF delta catenin family member [Source:HGNC Symbol;Acc:HGNC:728]
55752	SEPTIN11	septin 11 [Source:HGNC Symbol;Acc:HGNC:25589]
137872	ADHFE1	alcohol dehydrogenase iron containing 1 [Source:HGNC Symbol;Acc:HGNC:16354]
57732	ZFYVE28	zinc finger FYVE-type containing 28 [Source:HGNC Symbol;Acc:HGNC:29334]
9315	NREP	neuronal regeneration related protein [Source:HGNC Symbol;Acc:HGNC:16834]
3800	KIF5C	kinesin family member 5C [Source:HGNC Symbol;Acc:HGNC:6325]
283742	FAM98B	family with sequence similarity 98 member B [Source:HGNC Symbol;Acc:HGNC:26773]
85495	RPPH1	ribonuclease P RNA component H1 [Source:HGNC Symbol;Acc:HGNC:19273]
3735	KARS1	lysyl-tRNA synthetase 1 [Source:HGNC Symbol;Acc:HGNC:6215]
55217	TMLHE	trimethyllysine hydroxylase, epsilon [Source:HGNC Symbol;Acc:HGNC:18308]
347735	SERINC2	serine incorporator 2 [Source:HGNC Symbol;Acc:HGNC:23231]
54843	SYTL2	synaptotagmin like 2 [Source:HGNC Symbol;Acc:HGNC:15585]
127253	TYW3	tRNA-yW synthesizing protein 3 homolog [Source:HGNC Symbol;Acc:HGNC:24757]
55006	TRMT61B	tRNA methyltransferase 61B [Source:HGNC Symbol;Acc:HGNC:26070]
23500	DAAM2	dishevelled associated activator of morphogenesis 2 [Source:HGNC Symbol;Acc:HGNC:18143]
1318	SLC31A2	solute carrier family 31 member 2 [Source:HGNC Symbol;Acc:HGNC:11017]
57505	AARS2	alanyl-tRNA synthetase 2, mitochondrial [Source:HGNC Symbol;Acc:HGNC:21022]
51729	WBP11	WW domain binding protein 11 [Source:HGNC Symbol;Acc:HGNC:16461]
10915	TCERG1	transcription elongation regulator 1 [Source:HGNC Symbol;Acc:HGNC:15630]
10929	SRSF8	serine and arginine rich splicing factor 8 [Source:HGNC Symbol;Acc:HGNC:16988]
64061	TSPYL2	TSPY like 2 [Source:HGNC Symbol;Acc:HGNC:24358]
123283	TARS3	threonyl-tRNA synthetase 3 [Source:HGNC Symbol;Acc:HGNC:24728]
57662	CAMSAP3	calmodulin regulated spectrin associated protein family member 3 [Source:HGNC Symbol;Acc:HGNC:29307]
10149	ADGRG2	adhesion G protein-coupled receptor G2 [Source:HGNC Symbol;Acc:HGNC:4516]
57418	WDR18	WD repeat domain 18 [Source:HGNC Symbol;Acc:HGNC:17956]
10236	HNRNPR	heterogeneous nuclear ribonucleoprotein R [Source:HGNC Symbol;Acc:HGNC:5047]
10644	IGF2BP2	insulin like growth factor 2 mRNA binding protein 2 [Source:HGNC Symbol;Acc:HGNC:28867]
9788	MTSS1	MTSS I-BAR domain containing 1 [Source:HGNC Symbol;Acc:HGNC:20443]
93058	COQ10A	coenzyme Q10A [Source:HGNC Symbol;Acc:HGNC:26515]
8417	STX7	syntaxin 7 [Source:HGNC Symbol;Acc:HGNC:11442]
146225	CMTM2	CKLF like MARVEL transmembrane domain containing 2 [Source:HGNC Symbol;Acc:HGNC:19173]
10903	MTMR11	myotubularin related protein 11 [Source:HGNC Symbol;Acc:HGNC:24307]
10147	SUGP2	SURP and G-patch domain containing 2 [Source:HGNC Symbol;Acc:HGNC:18641]
219402	MTIF3	mitochondrial translational initiation factor 3 [Source:HGNC Symbol;Acc:HGNC:29788]
100033422	SNORD116-10	small nucleolar RNA, C/D box 116-10 [Source:HGNC Symbol;Acc:HGNC:33076]
100113407	TMEM170B	transmembrane protein 170B [Source:HGNC Symbol;Acc:HGNC:34244]
100128023	DPPA2P3	developmental pluripotency associated 2 pseudogene 3 [Source:HGNC Symbol;Acc:HGNC:44628]
100128420	LINC00892	long intergenic non-protein coding RNA 892 [Source:HGNC Symbol;Acc:HGNC:48578]
100128511	NEBL-AS1	NEBL antisense RNA 1 [Source:HGNC Symbol;Acc:HGNC:44899]
100128927	ZBTB42	zinc finger and BTB domain containing 42 [Source:HGNC Symbol;Acc:HGNC:32550]
100129662	FGF13-AS1	FGF13 antisense RNA 1 [Source:HGNC Symbol;Acc:HGNC:44264]
100130418	CECR7	cat eye syndrome chromosome region, candidate 7 [Source:HGNC Symbol;Acc:HGNC:1845]
100130581	LINC00910	long intergenic non-protein coding RNA 910 [Source:HGNC Symbol;Acc:HGNC:44361]
100130691	AC019080.1	novel transcript
100130733	LRRC70	leucine rich repeat containing 70 [Source:HGNC Symbol;Acc:HGNC:35155]
100130889	PSORS1C3	psoriasis susceptibility 1 candidate 3 [Source:HGNC Symbol;Acc:HGNC:17203]
100131176	WDR86-AS1	WDR86 antisense RNA 1 [Source:HGNC Symbol;Acc:HGNC:41186]
100132963	SMIM9	small integral membrane protein 9 [Source:HGNC Symbol;Acc:HGNC:41915]
100133957	UXT-AS1	UXT antisense RNA 1 [Source:HGNC Symbol;Acc:HGNC:49239]
100188949	LINC00426	long intergenic non-protein coding RNA 426 [Source:HGNC Symbol;Acc:HGNC:42761]
100190949	C5orf52	chromosome 5 open reading frame 52 [Source:HGNC Symbol;Acc:HGNC:35121]
100216479	FAR2P2	fatty acyl-CoA reductase 2 pseudogene 2 [Source:HGNC Symbol;Acc:HGNC:49279]
100288432	IL10RB-DT	IL10RB divergent transcript [Source:HGNC Symbol;Acc:HGNC:44303]
100289495	AL022069.3	novel transcript
100289678	ZNF783	zinc finger family member 783 [Source:HGNC Symbol;Acc:HGNC:27222]
100499483	CCDC180	coiled-coil domain containing 180 [Source:HGNC Symbol;Acc:HGNC:29303]
100505545	LINC01095	long intergenic non-protein coding RNA 1095 [Source:HGNC Symbol;Acc:HGNC:49220]
100505664	AL118508.1	novel transcript
100505985	AL138826.1	uncharacterized LOC100505985 [Source:NCBI gene (formerly Entrezgene);Acc:100505985]
100506211	MIR210HG	MIR210 host gene [Source:HGNC Symbol;Acc:HGNC:39524]
100506229	LINC01093	long intergenic non-protein coding RNA 1093 [Source:HGNC Symbol;Acc:HGNC:49218]
100506462	SRD5A3-AS1	SRD5A3 antisense RNA 1 [Source:HGNC Symbol;Acc:HGNC:44138]
100506736	SLFN12L	schlafen family member 12 like [Source:HGNC Symbol;Acc:HGNC:33920]
100506776	TRG-AS1	T cell receptor gamma locus antisense RNA 1 [Source:HGNC Symbol;Acc:HGNC:48974]
100506779	TSPOAP1-AS1	TSPOAP1, SUPT4H1 and RNF43 antisense RNA 1 [Source:HGNC Symbol;Acc:HGNC:44148]
100506810	LINC01132	long intergenic non-protein coding RNA 1132 [Source:HGNC Symbol;Acc:HGNC:49444]
100506994	PTPRG-AS1	PTPRG antisense RNA 1 [Source:HGNC Symbol;Acc:HGNC:44638]
100507261	MIR670HG	MIR670 host gene [Source:HGNC Symbol;Acc:HGNC:49204]
100507421	TMEM178B	transmembrane protein 178B [Source:HGNC Symbol;Acc:HGNC:44112]
100507629	LINC00658	long intergenic non-protein coding RNA 658 [Source:HGNC Symbol;Acc:HGNC:44315]
100507670	ZNF687-AS1	ZNF687 antisense RNA 1 [Source:HGNC Symbol;Acc:HGNC:54048]
100616668	TPTE2P5	transmembrane phosphoinositide 3-phosphatase and tensin homolog 2 pseudogene 5 [Source:HGNC Symbol;Acc:HGNC:42356]
100630918	PARTICL	promoter of MAT2A antisense radiation-induced circulating long non-coding RNA [Source:HGNC Symbol;Acc:HGNC:50886]
100652883	ALOX15P1	arachidonate 15-lipoxygenase pseudogene 1 [Source:HGNC Symbol;Acc:HGNC:13742]
100820829	MYZAP	myocardial zonula adherens protein [Source:HGNC Symbol;Acc:HGNC:43444]
10086	HHLA1	HERV-H LTR-associating 1 [Source:HGNC Symbol;Acc:HGNC:4904]
100862692	DIP2A-IT1	DIP2A intronic transcript 1 [Source:HGNC Symbol;Acc:HGNC:41430]
100873932	DPYD-AS1	DPYD antisense RNA 1 [Source:HGNC Symbol;Acc:HGNC:40195]
100873946	VAV3-AS1	VAV3 antisense RNA 1 [Source:HGNC Symbol;Acc:HGNC:40608]
100873989	GRM5-AS1	GRM5 antisense RNA 1 [Source:HGNC Symbol;Acc:HGNC:40265]
100874083	KCNAB1-AS2	KCNAB1 antisense RNA 2 [Source:HGNC Symbol;Acc:HGNC:40315]
100874151	LINC00381	long intergenic non-protein coding RNA 381 [Source:HGNC Symbol;Acc:HGNC:42708]
100874182	LINC00424	long intergenic non-protein coding RNA 424 [Source:HGNC Symbol;Acc:HGNC:42815]
100874212	MYCBP2-AS1	MYCBP2 antisense RNA 1 [Source:HGNC Symbol;Acc:HGNC:41023]
100874255	RNASEH2B-AS1	RNASEH2B antisense RNA 1 [Source:HGNC Symbol;Acc:HGNC:39967]
100874264	AOAH-IT1	AOAH intronic transcript 1 [Source:HGNC Symbol;Acc:HGNC:41307]
10150	MBNL2	muscleblind like splicing regulator 2 [Source:HGNC Symbol;Acc:HGNC:16746]
10158	PDZK1IP1	PDZK1 interacting protein 1 [Source:HGNC Symbol;Acc:HGNC:16887]
101927167	GATA2-AS1	GATA2 antisense RNA 1 [Source:HGNC Symbol;Acc:HGNC:51108]
101927590	LINC01166	long intergenic non-protein coding RNA 1166 [Source:HGNC Symbol;Acc:HGNC:49535]
10208	USPL1	ubiquitin specific peptidase like 1 [Source:HGNC Symbol;Acc:HGNC:20294]
10223	GPA33	glycoprotein A33 [Source:HGNC Symbol;Acc:HGNC:4445]
10314	LANCL1	LanC like 1 [Source:HGNC Symbol;Acc:HGNC:6508]
1033	CDKN3	cyclin dependent kinase inhibitor 3 [Source:HGNC Symbol;Acc:HGNC:1791]
10406	WFDC2	WAP four-disulfide core domain 2 [Source:HGNC Symbol;Acc:HGNC:15939]
10529	NEBL	nebulette [Source:HGNC Symbol;Acc:HGNC:16932]
105379444	FAM27C	family with sequence similarity 27 member C [Source:HGNC Symbol;Acc:HGNC:23668]
10561	IFI44	interferon induced protein 44 [Source:HGNC Symbol;Acc:HGNC:16938]
10609	P3H4	prolyl 3-hydroxylase family member 4 (inactive) [Source:HGNC Symbol;Acc:HGNC:16946]
10648	SCGB1D1	secretoglobin family 1D member 1 [Source:HGNC Symbol;Acc:HGNC:18395]
10732	TCFL5	transcription factor like 5 [Source:HGNC Symbol;Acc:HGNC:11646]
10742	RAI2	retinoic acid induced 2 [Source:HGNC Symbol;Acc:HGNC:9835]
10826	FAXDC2	fatty acid hydroxylase domain containing 2 [Source:HGNC Symbol;Acc:HGNC:1334]
10865	ARID5A	AT-rich interaction domain 5A [Source:HGNC Symbol;Acc:HGNC:17361]
10924	SMPDL3A	sphingomyelin phosphodiesterase acid like 3A [Source:HGNC Symbol;Acc:HGNC:17389]
11043	MID2	midline 2 [Source:HGNC Symbol;Acc:HGNC:7096]
11055	ZPBP	zona pellucida binding protein [Source:HGNC Symbol;Acc:HGNC:15662]
11123	RCAN3	RCAN family member 3 [Source:HGNC Symbol;Acc:HGNC:3042]
11138	TBC1D8	TBC1 domain family member 8 [Source:HGNC Symbol;Acc:HGNC:17791]
112267859	AQP7P3	aquaporin 7 pseudogene 3 [Source:HGNC Symbol;Acc:HGNC:31976]
11228	RASSF8	Ras association domain family member 8 [Source:HGNC Symbol;Acc:HGNC:13232]
112609	MRAP2	melanocortin 2 receptor accessory protein 2 [Source:HGNC Symbol;Acc:HGNC:21232]
11262	SP140	SP140 nuclear body protein [Source:HGNC Symbol;Acc:HGNC:17133]
11278	KLF12	Kruppel like factor 12 [Source:HGNC Symbol;Acc:HGNC:6346]
112840	WDR89	WD repeat domain 89 [Source:HGNC Symbol;Acc:HGNC:20489]
11341	SCRG1	stimulator of chondrogenesis 1 [Source:HGNC Symbol;Acc:HGNC:17036]
113419	TEX261	testis expressed 261 [Source:HGNC Symbol;Acc:HGNC:30712]
114783	LMTK3	lemur tyrosine kinase 3 [Source:HGNC Symbol;Acc:HGNC:19295]
114819	CROCCP3	CROCC pseudogene 3 [Source:HGNC Symbol;Acc:HGNC:29405]
114824	PNMA5	PNMA family member 5 [Source:HGNC Symbol;Acc:HGNC:18743]
114908	TMEM123	transmembrane protein 123 [Source:HGNC Symbol;Acc:HGNC:30138]
114928	GPRASP2	G protein-coupled receptor associated sorting protein 2 [Source:HGNC Symbol;Acc:HGNC:25169]
114991	ZNF618	zinc finger protein 618 [Source:HGNC Symbol;Acc:HGNC:29416]
116092	DNTTIP1	deoxynucleotidyltransferase terminal interacting protein 1 [Source:HGNC Symbol;Acc:HGNC:16160]
116135	LRRC3B	leucine rich repeat containing 3B [Source:HGNC Symbol;Acc:HGNC:28105]
116159	CYYR1	cysteine and tyrosine rich 1 [Source:HGNC Symbol;Acc:HGNC:16274]
116236	ABHD15	abhydrolase domain containing 15 [Source:HGNC Symbol;Acc:HGNC:26971]
116729	PPP1R27	protein phosphatase 1 regulatory subunit 27 [Source:HGNC Symbol;Acc:HGNC:16813]
116843	SLC18B1	solute carrier family 18 member B1 [Source:HGNC Symbol;Acc:HGNC:21573]
116933	CLRN1-AS1	CLRN1 antisense RNA 1 [Source:HGNC Symbol;Acc:HGNC:30895]
118426	BORCS5	BLOC-1 related complex subunit 5 [Source:HGNC Symbol;Acc:HGNC:17950]
118670	FAM24A	family with sequence similarity 24 member A [Source:HGNC Symbol;Acc:HGNC:23470]
119369	NUDT9P1	nudix hydrolase 9 pseudogene 1 [Source:HGNC Symbol;Acc:HGNC:28523]
119587	CPXM2	carboxypeptidase X, M14 family member 2 [Source:HGNC Symbol;Acc:HGNC:26977]
121793	TEX29	testis expressed 29 [Source:HGNC Symbol;Acc:HGNC:20370]
122664	TPPP2	tubulin polymerization promoting protein family member 2 [Source:HGNC Symbol;Acc:HGNC:19293]
122945	NOXRED1	NADP dependent oxidoreductase domain containing 1 [Source:HGNC Symbol;Acc:HGNC:20487]
123036	TC2N	tandem C2 domains, nuclear [Source:HGNC Symbol;Acc:HGNC:19859]
124936	CYB5D2	cytochrome b5 domain containing 2 [Source:HGNC Symbol;Acc:HGNC:28471]
125931	CEACAM20	CEA cell adhesion molecule 20 [Source:HGNC Symbol;Acc:HGNC:24879]
125972	CALR3	calreticulin 3 [Source:HGNC Symbol;Acc:HGNC:20407]
126917	IFFO2	intermediate filament family orphan 2 [Source:HGNC Symbol;Acc:HGNC:27006]
127687	C1orf122	chromosome 1 open reading frame 122 [Source:HGNC Symbol;Acc:HGNC:24789]
127733	UBXN10	UBX domain protein 10 [Source:HGNC Symbol;Acc:HGNC:26354]
128346	C1orf162	chromosome 1 open reading frame 162 [Source:HGNC Symbol;Acc:HGNC:28344]
128553	TSHZ2	teashirt zinc finger homeobox 2 [Source:HGNC Symbol;Acc:HGNC:13010]
128611	ZNF831	zinc finger protein 831 [Source:HGNC Symbol;Acc:HGNC:16167]
128646	SIRPD	signal regulatory protein delta [Source:HGNC Symbol;Acc:HGNC:16248]
128854	TSPY26P	testis specific protein Y-linked 26, pseudogene [Source:HGNC Symbol;Acc:HGNC:16256]
128872	HMGB3P1	high mobility group box 3 pseudogene 1 [Source:HGNC Symbol;Acc:HGNC:16240]
129852	C2orf73	chromosome 2 open reading frame 73 [Source:HGNC Symbol;Acc:HGNC:26861]
130814	SLC66A3	solute carrier family 66 member 3 [Source:HGNC Symbol;Acc:HGNC:28503]
130940	CCDC148	coiled-coil domain containing 148 [Source:HGNC Symbol;Acc:HGNC:25191]
131034	CPNE4	copine 4 [Source:HGNC Symbol;Acc:HGNC:2317]
131544	CRYBG3	crystallin beta-gamma domain containing 3 [Source:HGNC Symbol;Acc:HGNC:34427]
132001	TAMM41	TAM41 mitochondrial translocator assembly and maintenance homolog [Source:HGNC Symbol;Acc:HGNC:25187]
132989	C4orf36	chromosome 4 open reading frame 36 [Source:HGNC Symbol;Acc:HGNC:28386]
133923	ZNF474	zinc finger protein 474 [Source:HGNC Symbol;Acc:HGNC:23245]
135644	TRIM40	tripartite motif containing 40 [Source:HGNC Symbol;Acc:HGNC:18736]
1357	CPA1	carboxypeptidase A1 [Source:HGNC Symbol;Acc:HGNC:2296]
136259	KLF14	Kruppel like factor 14 [Source:HGNC Symbol;Acc:HGNC:23025]
136263	SSMEM1	serine rich single-pass membrane protein 1 [Source:HGNC Symbol;Acc:HGNC:29580]
138715	ARID3C	AT-rich interaction domain 3C [Source:HGNC Symbol;Acc:HGNC:21209]
139628	FOXR2	forkhead box R2 [Source:HGNC Symbol;Acc:HGNC:30469]
142940	TRUB1	TruB pseudouridine synthase family member 1 [Source:HGNC Symbol;Acc:HGNC:16060]
143630	UBQLNL	ubiquilin like [Source:HGNC Symbol;Acc:HGNC:28294]
143884	CWF19L2	CWF19 like cell cycle control factor 2 [Source:HGNC Symbol;Acc:HGNC:26508]
143888	POGLUT3	protein O-glucosyltransferase 3 [Source:HGNC Symbol;Acc:HGNC:28496]
144571	A2M-AS1	A2M antisense RNA 1 [Source:HGNC Symbol;Acc:HGNC:27057]
145270	PRIMA1	proline rich membrane anchor 1 [Source:HGNC Symbol;Acc:HGNC:18319]
145567	TTC7B	tetratricopeptide repeat domain 7B [Source:HGNC Symbol;Acc:HGNC:19858]

145853	C15orf61	chromosome 15 open reading frame 61 [Source:HGNC Symbol;Acc:HGNC:34453]
146050	ZSCAN29	zinc finger and SCAN domain containing 29 [Source:HGNC Symbol;Acc:HGNC:26673]
146857	SLFN13	schlafen family member 13 [Source:HGNC Symbol;Acc:HGNC:26481]
147015	DHRS13	dehydrogenase/reductase 13 [Source:HGNC Symbol;Acc:HGNC:28326]
147495	APCDD1	APC down-regulated 1 [Source:HGNC Symbol;Acc:HGNC:15718]
147699	PPM1N	protein phosphatase, Mg2+/Mn2+ dependent 1N (putative) [Source:HGNC Symbol;Acc:HGNC:26845]
147807	ZNF524	zinc finger protein 524 [Source:HGNC Symbol;Acc:HGNC:28322]
149473	CCDC24	coiled-coil domain containing 24 [Source:HGNC Symbol;Acc:HGNC:28688]
149478	BTBD19	BTB domain containing 19 [Source:HGNC Symbol;Acc:HGNC:27145]
149483	CCDC17	coiled-coil domain containing 17 [Source:HGNC Symbol;Acc:HGNC:26574]
149628	PYHIN1	pyrin and HIN domain family member 1 [Source:HGNC Symbol;Acc:HGNC:28894]
150468	CKAP2L	cytoskeleton associated protein 2 like [Source:HGNC Symbol;Acc:HGNC:26877]
150864	FAM117B	family with sequence similarity 117 member B [Source:HGNC Symbol;Acc:HGNC:14440]
151278	CCDC140	CCDC140 long non-coding RNA [Source:HGNC Symbol;Acc:HGNC:26514]
151651	EFHB	EF-hand domain family member B [Source:HGNC Symbol;Acc:HGNC:26330]
152024	LINC00691	long intergenic non-protein coding RNA 691 [Source:HGNC Symbol;Acc:HGNC:44514]
152195	NUDT16L2P	nudix hydrolase 16 like 2, pseudogene [Source:HGNC Symbol;Acc:HGNC:27189]
152579	SCFD2	sec1 family domain containing 2 [Source:HGNC Symbol;Acc:HGNC:30676]
153339	TMEM167A	transmembrane protein 167A [Source:HGNC Symbol;Acc:HGNC:28330]
1534	CYB561	cytochrome b561 [Source:HGNC Symbol;Acc:HGNC:2571]
153443	SRFBP1	serum response factor binding protein 1 [Source:HGNC Symbol;Acc:HGNC:26333]
153643	FAM81B	family with sequence similarity 81 member B [Source:HGNC Symbol;Acc:HGNC:26335]
153910	AL359313.1	novel transcript
154075	SAMD3	sterile alpha motif domain containing 3 [Source:HGNC Symbol;Acc:HGNC:21574]
154761	AC099548.2	family with sequence similarity 115, member C pseudogene [Source:NCBI gene (formerly Entrezgene);Acc:154761]
157273	AC022784.1	uncharacterized LOC157273 [Source:NCBI gene (formerly Entrezgene);Acc:157273]
157638	LRATD2	LRAT domain containing 2 [Source:HGNC Symbol;Acc:HGNC:24166]
158046	NXNL2	nucleoredoxin like 2 [Source:HGNC Symbol;Acc:HGNC:30482]
158219	TTC39B	tetratricopeptide repeat domain 39B [Source:HGNC Symbol;Acc:HGNC:23704]
158427	TSTD2	thiosulfate sulfurtransferase like domain containing 2 [Source:HGNC Symbol;Acc:HGNC:30087]
158586	ZXDB	zinc finger X-linked duplicated B [Source:HGNC Symbol;Acc:HGNC:13199]
159090	FAM122B	family with sequence similarity 122B [Source:HGNC Symbol;Acc:HGNC:30490]
159091	FAM122C	family with sequence similarity 122C [Source:HGNC Symbol;Acc:HGNC:25202]
160313	KRT19P2	keratin 19 pseudogene 2 [Source:HGNC Symbol;Acc:HGNC:33423]
160897	GPR180	G protein-coupled receptor 180 [Source:HGNC Symbol;Acc:HGNC:28899]
161291	TMEM30B	transmembrane protein 30B [Source:HGNC Symbol;Acc:HGNC:27254]
161394	SAMD15	sterile alpha motif domain containing 15 [Source:HGNC Symbol;Acc:HGNC:18631]
161424	NOP9	NOP9 nucleolar protein [Source:HGNC Symbol;Acc:HGNC:19826]
163778	SPRR4	small proline rich protein 4 [Source:HGNC Symbol;Acc:HGNC:23173]
165631	PARP15	poly(ADP-ribose) polymerase family member 15 [Source:HGNC Symbol;Acc:HGNC:26876]
1662	DDX10	DEAD-box helicase 10 [Source:HGNC Symbol;Acc:HGNC:2735]
169714	QSOX2	quiescin sulfhydryl oxidase 2 [Source:HGNC Symbol;Acc:HGNC:30249]
169834	ZNF883	zinc finger protein 883 [Source:HGNC Symbol;Acc:HGNC:27271]
170949	DEFT1P	defensin theta 1, pseudogene [Source:HGNC Symbol;Acc:HGNC:18339]
171389	NLRP6	NLR family pyrin domain containing 6 [Source:HGNC Symbol;Acc:HGNC:22944]
196383	RILPL2	Rab interacting lysosomal protein like 2 [Source:HGNC Symbol;Acc:HGNC:28787]
196472	FAM71C	family with sequence similarity 71 member C [Source:HGNC Symbol;Acc:HGNC:28594]
199223	TTC21A	tetratricopeptide repeat domain 21A [Source:HGNC Symbol;Acc:HGNC:30761]
200159	C1orf100	chromosome 1 open reading frame 100 [Source:HGNC Symbol;Acc:HGNC:30435]
200185	KRTCAP2	keratinocyte associated protein 2 [Source:HGNC Symbol;Acc:HGNC:28942]
200316	APOBEC3F	apolipoprotein B mRNA editing enzyme catalytic subunit 3F [Source:HGNC Symbol;Acc:HGNC:17356]
200420	ALMS1P1	ALMS1 pseudogene 1 [Source:HGNC Symbol;Acc:HGNC:29586]
200765	TIGD1	tigger transposable element derived 1 [Source:HGNC Symbol;Acc:HGNC:14523]
201161	CENPV	centromere protein V [Source:HGNC Symbol;Acc:HGNC:29920]
2012	EMP1	epithelial membrane protein 1 [Source:HGNC Symbol;Acc:HGNC:3333]
201305	SPNS3	sphingolipid transporter 3 (putative) [Source:HGNC Symbol;Acc:HGNC:28433]
201633	TIGIT	T cell immunoreceptor with Ig and ITIM domains [Source:HGNC Symbol;Acc:HGNC:26838]
201895	SMIM14	small integral membrane protein 14 [Source:HGNC Symbol;Acc:HGNC:27321]
202134	FAM153B	family with sequence similarity 153 member B [Source:HGNC Symbol;Acc:HGNC:27323]
203197	TMEM268	transmembrane protein 268 [Source:HGNC Symbol;Acc:HGNC:24513]
203427	SLC25A43	solute carrier family 25 member 43 [Source:HGNC Symbol;Acc:HGNC:30557]
203569	PAGE2	PAGE family member 2 [Source:HGNC Symbol;Acc:HGNC:31804]
219731	PROSER2-AS1	PROSER2 antisense RNA 1 [Source:HGNC Symbol;Acc:HGNC:27343]
219793	TBATA	thymus, brain and testes associated [Source:HGNC Symbol;Acc:HGNC:23511]
219899	TBCEL	tubulin folding cofactor E like [Source:HGNC Symbol;Acc:HGNC:28115]
220213	OTUD1	OTU deubiquitinase 1 [Source:HGNC Symbol;Acc:HGNC:27346]
220929	ZNF438	zinc finger protein 438 [Source:HGNC Symbol;Acc:HGNC:21029]
221476	PI16	peptidase inhibitor 16 [Source:HGNC Symbol;Acc:HGNC:21245]
221477	C6orf89	chromosome 6 open reading frame 89 [Source:HGNC Symbol;Acc:HGNC:21114]
221481	ARMC12	armadillo repeat containing 12 [Source:HGNC Symbol;Acc:HGNC:21099]
221883	HOXA11-AS	HOXA11 antisense RNA [Source:HGNC Symbol;Acc:HGNC:24957]
222223	ELAPOR2	endosome-lysosome associated apoptosis and autophagy regulator family member 2 [Source:HGNC Symbol;Acc:HGNC:21945]
22807	IKZF2	IKAROS family zinc finger 2 [Source:HGNC Symbol;Acc:HGNC:13177]
22844	FRMPD1	FERM and PDZ domain containing 1 [Source:HGNC Symbol;Acc:HGNC:29159]
22902	RUFY3	RUN and FYVE domain containing 3 [Source:HGNC Symbol;Acc:HGNC:30285]
22973	LAMB2P1	laminin subunit beta 2 pseudogene 1 [Source:HGNC Symbol;Acc:HGNC:6488]
2306	FOXD2	forkhead box D2 [Source:HGNC Symbol;Acc:HGNC:3803]
23070	CMTR1	cap methyltransferase 1 [Source:HGNC Symbol;Acc:HGNC:21077]
23094	SIPA1L3	signal induced proliferation associated 1 like 3 [Source:HGNC Symbol;Acc:HGNC:23801]
23111	SPART	spartin [Source:HGNC Symbol;Acc:HGNC:18514]
23180	RFTN1	raftlin, lipid raft linker 1 [Source:HGNC Symbol;Acc:HGNC:30278]
23184	MESD	mesoderm development LRP chaperone [Source:HGNC Symbol;Acc:HGNC:13520]
23199	GSE1	Gse1 coiled-coil protein [Source:HGNC Symbol;Acc:HGNC:28979]
23241	PACS2	phosphofurin acidic cluster sorting protein 2 [Source:HGNC Symbol;Acc:HGNC:23794]
23251	MINAR1	membrane integral NOTCH2 associated receptor 1 [Source:HGNC Symbol;Acc:HGNC:29172]
23264	ZC3H7B	zinc finger CCCH-type containing 7B [Source:HGNC Symbol;Acc:HGNC:30869]
23328	SASH1	SAM and SH3 domain containing 1 [Source:HGNC Symbol;Acc:HGNC:19182]
23333	DPY19L1	dpy-19 like C-mannosyltransferase 1 [Source:HGNC Symbol;Acc:HGNC:22205]
23336	SYNM	synemin [Source:HGNC Symbol;Acc:HGNC:24466]
23349	PHF24	PHD finger protein 24 [Source:HGNC Symbol;Acc:HGNC:29180]
23531	MMD	monocyte to macrophage differentiation associated [Source:HGNC Symbol;Acc:HGNC:7153]
23641	LDOC1	LDOC1 regulator of NFKB signaling [Source:HGNC Symbol;Acc:HGNC:6548]
23648	SSBP3	single stranded DNA binding protein 3 [Source:HGNC Symbol;Acc:HGNC:15674]
23731	TMEM245	transmembrane protein 245 [Source:HGNC Symbol;Acc:HGNC:1363]
23762	OSBP2	oxysterol binding protein 2 [Source:HGNC Symbol;Acc:HGNC:8504]
24141	LAMP5	lysosomal associated membrane protein family member 5 [Source:HGNC Symbol;Acc:HGNC:16097]
245	ALOX12P2	arachidonate 12-lipoxygenase pseudogene 2 [Source:HGNC Symbol;Acc:HGNC:432]
246126	TXLNGY	taxilin gamma pseudogene, Y-linked [Source:HGNC Symbol;Acc:HGNC:18473]
246777	SPESP1	sperm equatorial segment protein 1 [Source:HGNC Symbol;Acc:HGNC:15570]
252884	ZNF396	zinc finger protein 396 [Source:HGNC Symbol;Acc:HGNC:18824]
253152	EPHX4	epoxide hydrolase 4 [Source:HGNC Symbol;Acc:HGNC:23758]
254778	VXN	vexin [Source:HGNC Symbol;Acc:HGNC:28498]
254896	AC107959.4	novel transcript
255809	C19orf38	chromosome 19 open reading frame 38 [Source:HGNC Symbol;Acc:HGNC:34073]
256309	CCDC110	coiled-coil domain containing 110 [Source:HGNC Symbol;Acc:HGNC:28504]
256957	HEATR9	HEAT repeat containing 9 [Source:HGNC Symbol;Acc:HGNC:26548]
257019	FRMD3	FERM domain containing 3 [Source:HGNC Symbol;Acc:HGNC:24125]
257101	ZNF683	zinc finger protein 683 [Source:HGNC Symbol;Acc:HGNC:28495]
25822	DNAJB5	DnaJ heat shock protein family (Hsp40) member B5 [Source:HGNC Symbol;Acc:HGNC:14887]
25893	TRIM58	tripartite motif containing 58 [Source:HGNC Symbol;Acc:HGNC:24150]
25907	TMEM158	transmembrane protein 158 [Source:HGNC Symbol;Acc:HGNC:30293]
25911	DPCD	deleted in primary ciliary dyskinesia homolog (mouse) [Source:HGNC Symbol;Acc:HGNC:24542]
25914	RTTN	rotatin [Source:HGNC Symbol;Acc:HGNC:18654]
25923	ATL3	atlastin GTPase 3 [Source:HGNC Symbol;Acc:HGNC:24526]
259308	FAM205A	family with sequence similarity 205 member A [Source:HGNC Symbol;Acc:HGNC:41911]
25932	CLIC4	chloride intracellular channel 4 [Source:HGNC Symbol;Acc:HGNC:13518]
25976	TIPARP	TCDD inducible poly(ADP-ribose) polymerase [Source:HGNC Symbol;Acc:HGNC:23696]
26039	SS18L1	SS18L1 subunit of BAF chromatin remodeling complex [Source:HGNC Symbol;Acc:HGNC:15592]
26064	RAI14	retinoic acid induced 14 [Source:HGNC Symbol;Acc:HGNC:14873]
26083	TBC1D29P	TBC1 domain family member 29, pseudogene [Source:HGNC Symbol;Acc:HGNC:24509]
2615	LRRC32	leucine rich repeat containing 32 [Source:HGNC Symbol;Acc:HGNC:4161]
26499	PLEK2	pleckstrin 2 [Source:HGNC Symbol;Acc:HGNC:19238]
266697	POM121L4P	POM121 transmembrane nucleoporin like 4, pseudogene [Source:HGNC Symbol;Acc:HGNC:19326]
266812	NAP1L5	nucleosome assembly protein 1 like 5 [Source:HGNC Symbol;Acc:HGNC:19968]
267012	DAOA	D-amino acid oxidase activator [Source:HGNC Symbol;Acc:HGNC:21191]
26775	SNORA72	small nucleolar RNA, H/ACA box 72 [Source:HGNC Symbol;Acc:HGNC:10234]
26777	SNORA71A	small nucleolar RNA, H/ACA box 71A [Source:HGNC Symbol;Acc:HGNC:10232]
26996	GPR160	G protein-coupled receptor 160 [Source:HGNC Symbol;Acc:HGNC:23693]
27156	RSPH14	radial spoke head 14 homolog [Source:HGNC Symbol;Acc:HGNC:13437]
27201	GPR78	G protein-coupled receptor 78 [Source:HGNC Symbol;Acc:HGNC:4528]
283089	WDR11-AS1	WDR11 antisense RNA 1 [Source:HGNC Symbol;Acc:HGNC:27437]
283150	FOXR1	forkhead box R1 [Source:HGNC Symbol;Acc:HGNC:29980]
283219	KCTD21	potassium channel tetramerization domain containing 21 [Source:HGNC Symbol;Acc:HGNC:27452]
283248	RCOR2	REST corepressor 2 [Source:HGNC Symbol;Acc:HGNC:27455]
283316	CD163L1	CD163 molecule like 1 [Source:HGNC Symbol;Acc:HGNC:30375]
283507	SUGT1P3	SUGT1 pseudogene 3 [Source:HGNC Symbol;Acc:HGNC:20513]
283537	SLC46A3	solute carrier family 46 member 3 [Source:HGNC Symbol;Acc:HGNC:27501]
283578	TMED8	transmembrane p24 trafficking protein family member 8 [Source:HGNC Symbol;Acc:HGNC:18633]
283635	FAM177A1	family with sequence similarity 177 member A1 [Source:HGNC Symbol;Acc:HGNC:19829]
284323	ZNF780A	zinc finger protein 780A [Source:HGNC Symbol;Acc:HGNC:27603]
284576	LEMD1-AS1	LEMD1 antisense RNA 1 [Source:HGNC Symbol;Acc:HGNC:44132]
284649	SMG7-AS1	SMG7 antisense RNA 1 [Source:HGNC Symbol;Acc:HGNC:24518]
284688	LINC01142	long intergenic non-protein coding RNA 1142 [Source:HGNC Symbol;Acc:HGNC:49456]
284697	BTBD8	BTB domain containing 8 [Source:HGNC Symbol;Acc:HGNC:21019]
284751	LINC01270	long intergenic non-protein coding RNA 1270 [Source:HGNC Symbol;Acc:HGNC:27658]
284759	SIRPB2	signal regulatory protein beta 2 [Source:HGNC Symbol;Acc:HGNC:16247]
284805	C20orf203	chromosome 20 open reading frame 203 [Source:HGNC Symbol;Acc:HGNC:26592]
285016	ALKAL2	ALK and LTK ligand 2 [Source:HGNC Symbol;Acc:HGNC:27683]
285097	AC093642.2	TEC
285154	CYP1B1-AS1	CYP1B1 antisense RNA 1 [Source:HGNC Symbol;Acc:HGNC:28543]
285429	DCAF4L1	DDB1 and CUL4 associated factor 4 like 1 [Source:HGNC Symbol;Acc:HGNC:27723]
285590	SH3PXD2B	SH3 and PX domains 2B [Source:HGNC Symbol;Acc:HGNC:29242]
285848	PNPLA1	patatin like phospholipase domain containing 1 [Source:HGNC Symbol;Acc:HGNC:21246]
286016	TPI1P2	triosephosphate isomerase 1 pseudogene 2 [Source:HGNC Symbol;Acc:HGNC:38069]
286076	LINC02878	long intergenic non-protein coding RNA 2878 [Source:HGNC Symbol;Acc:HGNC:54524]
286135	LINC02209	long intergenic non-protein coding RNA 2209 [Source:HGNC Symbol;Acc:HGNC:27827]
286183	NKAIN3	sodium/potassium transporting ATPase interacting 3 [Source:HGNC Symbol;Acc:HGNC:26829]
28951	TRIB2	tribbles pseudokinase 2 [Source:HGNC Symbol;Acc:HGNC:30809]
28959	TMEM176B	transmembrane protein 176B [Source:HGNC Symbol;Acc:HGNC:29596]
28966	SNX24	sorting nexin 24 [Source:HGNC Symbol;Acc:HGNC:21533]
29018	FOXN3-AS2	FOXN3 antisense RNA 2 [Source:HGNC Symbol;Acc:HGNC:30119]
2907	GRINA	glutamate ionotropic receptor NMDA type subunit associated protein 1 [Source:HGNC Symbol;Acc:HGNC:4589]
29097	CNIH4	cornichon family AMPA receptor auxiliary protein 4 [Source:HGNC Symbol;Acc:HGNC:25013]
3003	GZMK	granzyme K [Source:HGNC Symbol;Acc:HGNC:4711]
3044	HBBP1	hemoglobin subunit beta pseudogene 1 [Source:HGNC Symbol;Acc:HGNC:4828]
313	AOAH	acyloxyacyl hydrolase [Source:HGNC Symbol;Acc:HGNC:548]
3131	HLF	HLF transcription factor, PAR bZIP family member [Source:HGNC Symbol;Acc:HGNC:4977]
317762	CCDC85C	coiled-coil domain containing 85C [Source:HGNC Symbol;Acc:HGNC:35459]
3208	HPCA	hippocalcin [Source:HGNC Symbol;Acc:HGNC:5144]
326342	ADGRE4P	adhesion G protein-coupled receptor E4, pseudogene [Source:HGNC Symbol;Acc:HGNC:19240]
3268	AGFG2	ArfGAP with FG repeats 2 [Source:HGNC Symbol;Acc:HGNC:5177]
338773	TMEM119	transmembrane protein 119 [Source:HGNC Symbol;Acc:HGNC:27884]
339512	CCDC190	coiled-coil domain containing 190 [Source:HGNC Symbol;Acc:HGNC:28736]
339906	PRSS42P	serine protease 42, pseudogene [Source:HGNC Symbol;Acc:HGNC:30716]
340508	GAS2L1P2	growth arrest specific 2 like 1 pseudogene 2 [Source:HGNC Symbol;Acc:HGNC:27986]
340526	RTL5	retrotransposon Gag like 5 [Source:HGNC Symbol;Acc:HGNC:29430]
340542	BEX5	brain expressed X-linked 5 [Source:HGNC Symbol;Acc:HGNC:27990]
340784	HMX3	H6 family homeobox 3 [Source:HGNC Symbol;Acc:HGNC:5019]
343071	PRAMEF10	PRAME family member 10 [Source:HGNC Symbol;Acc:HGNC:27997]
343263	MYBPHL	myosin binding protein H like [Source:HGNC Symbol;Acc:HGNC:30434]
343521	TCTEX1D4	Tctex1 domain containing 4 [Source:HGNC Symbol;Acc:HGNC:32315]
346157	ZNF391	zinc finger protein 391 [Source:HGNC Symbol;Acc:HGNC:18779]
348093	RBPMS2	RNA binding protein, mRNA processing factor 2 [Source:HGNC Symbol;Acc:HGNC:19098]
348645	C22orf34	chromosome 22 putative open reading frame 34 [Source:HGNC Symbol;Acc:HGNC:28010]
349152	DPY19L2P2	DPY19L2 pseudogene 2 [Source:HGNC Symbol;Acc:HGNC:21764]
352962	HLA-V	major histocompatibility complex, class I, V (pseudogene) [Source:HGNC Symbol;Acc:HGNC:23482]
353345	GPR141	G protein-coupled receptor 141 [Source:HGNC Symbol;Acc:HGNC:19997]
3580	CXCR2P1	C-X-C motif chemokine receptor 2 pseudogene 1 [Source:HGNC Symbol;Acc:HGNC:6028]
360023	ZBTB41	zinc finger and BTB domain containing 41 [Source:HGNC Symbol;Acc:HGNC:24819]
374882	TMEM205	transmembrane protein 205 [Source:HGNC Symbol;Acc:HGNC:29631]
375061	FAM89A	family with sequence similarity 89 member A [Source:HGNC Symbol;Acc:HGNC:25057]
375190	FAM228B	family with sequence similarity 228 member B [Source:HGNC Symbol;Acc:HGNC:24736]
375248	ANKRD36	ankyrin repeat domain 36 [Source:HGNC Symbol;Acc:HGNC:24079]
375346	STIMATE	STIM activating enhancer [Source:HGNC Symbol;Acc:HGNC:30526]
375757	SWI5	SWI5 homologous recombination repair protein [Source:HGNC Symbol;Acc:HGNC:31412]
379013	RNF138P1	ring finger protein 138 pseudogene 1 [Source:HGNC Symbol;Acc:HGNC:30342]
387104	SOGA3	SOGA family member 3 [Source:HGNC Symbol;Acc:HGNC:21494]
387357	THEMIS	thymocyte selection associated [Source:HGNC Symbol;Acc:HGNC:21569]
387751	GVINP1	GTPase, very large interferon inducible pseudogene 1 [Source:HGNC Symbol;Acc:HGNC:25813]
387763	C11orf96	chromosome 11 open reading frame 96 [Source:HGNC Symbol;Acc:HGNC:38675]
387882	C12orf75	chromosome 12 open reading frame 75 [Source:HGNC Symbol;Acc:HGNC:35164]
387923	SERP2	stress associated endoplasmic reticulum protein family member 2 [Source:HGNC Symbol;Acc:HGNC:20607]
388125	C2CD4B	C2 calcium dependent domain containing 4B [Source:HGNC Symbol;Acc:HGNC:33628]
388228	SBK1	SH3 domain binding kinase 1 [Source:HGNC Symbol;Acc:HGNC:17699]
388407	LINC02875	long intergenic non-protein coding RNA 2875 [Source:HGNC Symbol;Acc:HGNC:32699]
388965	FUNDC2P2	FUN14 domain containing 2 pseudogene 2 [Source:HGNC Symbol;Acc:HGNC:17247]
389125	MUSTN1	musculoskeletal, embryonic nuclear protein 1 [Source:HGNC Symbol;Acc:HGNC:22144]
389257	LRRC14B	leucine rich repeat containing 14B [Source:HGNC Symbol;Acc:HGNC:37268]
389384	BNIP5	BCL2 interacting protein 5 [Source:HGNC Symbol;Acc:HGNC:33769]
389634	LINC00937	long intergenic non-protein coding RNA 937 [Source:HGNC Symbol;Acc:HGNC:48629]
389813	AJM1	apical junction component 1 homolog [Source:HGNC Symbol;Acc:HGNC:37284]
389860	PAGE2B	PAGE family member 2B [Source:HGNC Symbol;Acc:HGNC:31805]
390226	GUCY2EP	guanylate cyclase 2E, pseudogene [Source:HGNC Symbol;Acc:HGNC:4690]
390245	KDM4E	lysine demethylase 4E [Source:HGNC Symbol;Acc:HGNC:37098]
391518	VENTXP7	VENT homeobox pseudogene 7 [Source:HGNC Symbol;Acc:HGNC:13638]
392288	MAP1LC3BP1	microtubule associated protein 1 light chain 3 beta pseudogene 1 [Source:HGNC Symbol;Acc:HGNC:49783]
392509	ARL13A	ADP ribosylation factor like GTPase 13A [Source:HGNC Symbol;Acc:HGNC:31709]
399665	FAM102A	family with sequence similarity 102 member A [Source:HGNC Symbol;Acc:HGNC:31419]
399972	GSEC	G-quadruplex forming sequence containing lncRNA [Source:HGNC Symbol;Acc:HGNC:48645]

400046	LINC02389	long intergenic non-protein coding RNA 2389 [Source:HGNC Symbol;Acc:HGNC:53316]
400242	DICER1-AS1	DICER1 antisense RNA 1 [Source:HGNC Symbol;Acc:HGNC:43017]
400322	HERC2P2	hect domain and RLD 2 pseudogene 2 [Source:HGNC Symbol;Acc:HGNC:4870]
400512	NSMCE1-DT	NSMCE1 divergent transcript [Source:HGNC Symbol;Acc:HGNC:51368]
401074	LINC00960	long intergenic non-protein coding RNA 960 [Source:HGNC Symbol;Acc:HGNC:48710]
401093	MBNL1-AS1	MBNL1 antisense RNA 1 [Source:HGNC Symbol;Acc:HGNC:44584]
401124	DTHD1	death domain containing 1 [Source:HGNC Symbol;Acc:HGNC:37261]
401152	C4orf3	chromosome 4 open reading frame 3 [Source:HGNC Symbol;Acc:HGNC:19225]
401190	RGS7BP	regulator of G protein signaling 7 binding protein [Source:HGNC Symbol;Acc:HGNC:23271]
401251	SAPCD1	suppressor APC domain containing 1 [Source:HGNC Symbol;Acc:HGNC:13938]
401262	CRIP3	cysteine rich protein 3 [Source:HGNC Symbol;Acc:HGNC:17751]
401285	TCP10L2	t-complex 10 like 2, pseudogene [Source:HGNC Symbol;Acc:HGNC:21254]
401289	LINC01624	long intergenic non-protein coding RNA 1624 [Source:HGNC Symbol;Acc:HGNC:52051]
401399	PRRT4	proline rich transmembrane protein 4 [Source:HGNC Symbol;Acc:HGNC:37280]
401474	SAMD12	sterile alpha motif domain containing 12 [Source:HGNC Symbol;Acc:HGNC:31750]
401546	C9orf152	chromosome 9 open reading frame 152 [Source:HGNC Symbol;Acc:HGNC:31455]
404201	WDFY3-AS2	WDFY3 antisense RNA 2 [Source:HGNC Symbol;Acc:HGNC:21603]
404734	ANKHD1-EIF4EBP3	ANKHD1-EIF4EBP3 readthrough [Source:HGNC Symbol;Acc:HGNC:33530]
4063	LY9	lymphocyte antigen 9 [Source:HGNC Symbol;Acc:HGNC:6730]
4070	TACSTD2	tumor associated calcium signal transducer 2 [Source:HGNC Symbol;Acc:HGNC:11530]
414328	IDNK	IDNK gluconokinase [Source:HGNC Symbol;Acc:HGNC:31367]
414765	HCG25	HLA complex group 25 [Source:HGNC Symbol;Acc:HGNC:20196]
415116	PIM3	Pim-3 proto-oncogene, serine/threonine kinase [Source:HGNC Symbol;Acc:HGNC:19310]
4356	MPP3	membrane palmitoylated protein 3 [Source:HGNC Symbol;Acc:HGNC:7221]
439921	MXRA7	matrix remodeling associated 7 [Source:HGNC Symbol;Acc:HGNC:7541]
439949	PRKCQ-AS1	PRKCQ antisense RNA 1 [Source:HGNC Symbol;Acc:HGNC:44689]
440503	PLIN5	perilipin 5 [Source:HGNC Symbol;Acc:HGNC:33196]
440799	P2RX6P	purinergic receptor P2X 6 pseudogene [Source:HGNC Symbol;Acc:HGNC:34076]
441116	LINC01951	long intergenic non-protein coding RNA 1951 [Source:HGNC Symbol;Acc:HGNC:52774]
441307	HRAT92	heart tissue-associated transcript 92 [Source:NCBI gene (formerly Entrezgene);Acc:441307]
441317	FAM90A7P	family with sequence similarity 90 member A7, pseudogene [Source:HGNC Symbol;Acc:HGNC:32255]
441374	FLJ42969	uncharacterized LOC441374 [Source:NCBI gene (formerly Entrezgene);Acc:441374]
441549	CDNF	cerebral dopamine neurotrophic factor [Source:HGNC Symbol;Acc:HGNC:24913]
441666	BX322639.1	zinc finger protein pseudogene
441951	ZFAS1	ZNFX1 antisense RNA 1 [Source:HGNC Symbol;Acc:HGNC:33101]
4430	MYO1B	myosin IB [Source:HGNC Symbol;Acc:HGNC:7596]
4642	MYO1D	myosin ID [Source:HGNC Symbol;Acc:HGNC:7598]
4648	MYO7B	myosin VIIB [Source:HGNC Symbol;Acc:HGNC:7607]
4675	NAP1L3	nucleosome assembly protein 1 like 3 [Source:HGNC Symbol;Acc:HGNC:7639]
493856	CISD2	CDGSH iron sulfur domain 2 [Source:HGNC Symbol;Acc:HGNC:24212]
494119	SPANXN2	SPANX family member N2 [Source:HGNC Symbol;Acc:HGNC:33175]
5003	SLC22A18AS	solute carrier family 22 member 18 antisense [Source:HGNC Symbol;Acc:HGNC:10965]
5075	PAX1	paired box 1 [Source:HGNC Symbol;Acc:HGNC:8615]
50853	VILL	villin like [Source:HGNC Symbol;Acc:HGNC:30906]
51018	RRP15	ribosomal RNA processing 15 homolog [Source:HGNC Symbol;Acc:HGNC:24255]
51063	CALHM2	calcium homeostasis modulator family member 2 [Source:HGNC Symbol;Acc:HGNC:23493]
51101	ZC2HC1A	zinc finger C2HC-type containing 1A [Source:HGNC Symbol;Acc:HGNC:24277]
51161	C3orf18	chromosome 3 open reading frame 18 [Source:HGNC Symbol;Acc:HGNC:24837]
51177	PLEKHO1	pleckstrin homology domain containing O1 [Source:HGNC Symbol;Acc:HGNC:24310]
5129	CDK18	cyclin dependent kinase 18 [Source:HGNC Symbol;Acc:HGNC:8751]
51338	MS4A4A	membrane spanning 4-domains A4A [Source:HGNC Symbol;Acc:HGNC:13371]
51480	VCX2	variable charge X-linked 2 [Source:HGNC Symbol;Acc:HGNC:18158]
51537	MTFP1	mitochondrial fission process 1 [Source:HGNC Symbol;Acc:HGNC:26945]
51617	NSG2	neuronal vesicle trafficking associated 2 [Source:HGNC Symbol;Acc:HGNC:24955]
51629	SLC25A39	solute carrier family 25 member 39 [Source:HGNC Symbol;Acc:HGNC:24279]
51635	DHRS7	dehydrogenase/reductase 7 [Source:HGNC Symbol;Acc:HGNC:21524]
51678	MPP6	membrane palmitoylated protein 6 [Source:HGNC Symbol;Acc:HGNC:18167]
51754	TMEM8B	transmembrane protein 8B [Source:HGNC Symbol;Acc:HGNC:21427]
5184	PEPD	peptidase D [Source:HGNC Symbol;Acc:HGNC:8840]
5355	PLP2	proteolipid protein 2 [Source:HGNC Symbol;Acc:HGNC:9087]
541473	AC211486.1	FK506 binding protein 6, 36kDa (FKBP6) pseudogene
54221	SNTG2	syntrophin gamma 2 [Source:HGNC Symbol;Acc:HGNC:13741]
54432	YIPF1	Yip1 domain family member 1 [Source:HGNC Symbol;Acc:HGNC:25231]
54491	OTULINL	OTU deubiquitinase with linear linkage specificity like [Source:HGNC Symbol;Acc:HGNC:25629]
54494	C11orf71	chromosome 11 open reading frame 71 [Source:HGNC Symbol;Acc:HGNC:25937]
54497	HEATR5B	HEAT repeat containing 5B [Source:HGNC Symbol;Acc:HGNC:29273]
54550	NECAB2	N-terminal EF-hand calcium binding protein 2 [Source:HGNC Symbol;Acc:HGNC:23746]
54602	NDFIP2	Nedd4 family interacting protein 2 [Source:HGNC Symbol;Acc:HGNC:18537]
54680	ZNHIT6	zinc finger HIT-type containing 6 [Source:HGNC Symbol;Acc:HGNC:26089]
54758	KLHDC4	kelch domain containing 4 [Source:HGNC Symbol;Acc:HGNC:25272]
54765	TRIM44	tripartite motif containing 44 [Source:HGNC Symbol;Acc:HGNC:19016]
54809	SAMD9	sterile alpha motif domain containing 9 [Source:HGNC Symbol;Acc:HGNC:1348]
54832	VPS13C	vacuolar protein sorting 13 homolog C [Source:HGNC Symbol;Acc:HGNC:23594]
54847	SIDT1	SID1 transmembrane family member 1 [Source:HGNC Symbol;Acc:HGNC:25967]
54855	TENT5C	terminal nucleotidyltransferase 5C [Source:HGNC Symbol;Acc:HGNC:24712]
54865	GPATCH4	G-patch domain containing 4 [Source:HGNC Symbol;Acc:HGNC:25982]
54877	ZCCHC2	zinc finger CCHC-type containing 2 [Source:HGNC Symbol;Acc:HGNC:22916]
54906	TASOR2	transcription activation suppressor family member 2 [Source:HGNC Symbol;Acc:HGNC:23484]
54977	SLC25A38	solute carrier family 25 member 38 [Source:HGNC Symbol;Acc:HGNC:26054]
55002	TMCO3	transmembrane and coiled-coil domains 3 [Source:HGNC Symbol;Acc:HGNC:20329]
55020	TTC38	tetratricopeptide repeat domain 38 [Source:HGNC Symbol;Acc:HGNC:26082]
55022	PID1	phosphotyrosine interaction domain containing 1 [Source:HGNC Symbol;Acc:HGNC:26084]
55024	BANK1	B cell scaffold protein with ankyrin repeats 1 [Source:HGNC Symbol;Acc:HGNC:18233]
55086	RADX	RPA1 related single stranded DNA binding protein, X-linked [Source:HGNC Symbol;Acc:HGNC:25486]
55092	TMEM51	transmembrane protein 51 [Source:HGNC Symbol;Acc:HGNC:25488]
55093	NTAQ1	N-terminal glutamine amidase 1 [Source:HGNC Symbol;Acc:HGNC:25490]
55165	CEP55	centrosomal protein 55 [Source:HGNC Symbol;Acc:HGNC:1161]
55177	RMDN3	regulator of microtubule dynamics 3 [Source:HGNC Symbol;Acc:HGNC:25550]
55188	RIC8B	RIC8 guanine nucleotide exchange factor B [Source:HGNC Symbol;Acc:HGNC:25555]
55201	MAP1S	microtubule associated protein 1S [Source:HGNC Symbol;Acc:HGNC:15715]
55248	PACC1	proton activated chloride channel 1 [Source:HGNC Symbol;Acc:HGNC:25593]
55259	CFAP94	cilia and flagella associated protein 94 [Source:HGNC Symbol;Acc:HGNC:29599]
55277	FGGY	FGGY carbohydrate kinase domain containing [Source:HGNC Symbol;Acc:HGNC:25610]
55278	QRSL1	glutaminyl-tRNA amidotransferase subunit QRSL1 [Source:HGNC Symbol;Acc:HGNC:21020]
55296	TBC1D19	TBC1 domain family member 19 [Source:HGNC Symbol;Acc:HGNC:25624]
55333	SYNJ2BP	synaptojanin 2 binding protein [Source:HGNC Symbol;Acc:HGNC:18955]
55335	NIPSNAP3B	nipsnap homolog 3B [Source:HGNC Symbol;Acc:HGNC:23641]
55363	HEMGN	hemogen [Source:HGNC Symbol;Acc:HGNC:17509]
55365	TMEM176A	transmembrane protein 176A [Source:HGNC Symbol;Acc:HGNC:24930]
55450	CAMK2N1	calcium/calmodulin dependent protein kinase II inhibitor 1 [Source:HGNC Symbol;Acc:HGNC:24190]
55508	SLC35E3	solute carrier family 35 member E3 [Source:HGNC Symbol;Acc:HGNC:20864]
55511	SAGE1	sarcoma antigen 1 [Source:HGNC Symbol;Acc:HGNC:30369]
55552	ZNF823	zinc finger protein 823 [Source:HGNC Symbol;Acc:HGNC:30936]
55601	DDX60	DExD/H-box helicase 60 [Source:HGNC Symbol;Acc:HGNC:25942]
55622	TTC27	tetratricopeptide repeat domain 27 [Source:HGNC Symbol;Acc:HGNC:25986]
55633	TBC1D22B	TBC1 domain family member 22B [Source:HGNC Symbol;Acc:HGNC:21602]
55705	IPO9	importin 9 [Source:HGNC Symbol;Acc:HGNC:19425]
55853	IDI2-AS1	IDI2 antisense RNA 1 [Source:HGNC Symbol;Acc:HGNC:30885]
55876	GSDMB	gasdermin B [Source:HGNC Symbol;Acc:HGNC:23690]
55906	ZC4H2	zinc finger C4H2-type containing [Source:HGNC Symbol;Acc:HGNC:24931]
55972	SLC25A40	solute carrier family 25 member 40 [Source:HGNC Symbol;Acc:HGNC:29680]
56061	UBFD1	ubiquitin family domain containing 1 [Source:HGNC Symbol;Acc:HGNC:30565]
56127	PCDHB9	protocadherin beta 9 [Source:HGNC Symbol;Acc:HGNC:8694]
5616	PRKY	protein kinase Y-linked (pseudogene) [Source:HGNC Symbol;Acc:HGNC:9444]
56203	LMOD3	leiomodin 3 [Source:HGNC Symbol;Acc:HGNC:6649]
56663	VTRNA1-2	vault RNA 1-2 [Source:HGNC Symbol;Acc:HGNC:12655]
56849	TCEAL7	transcription elongation factor A like 7 [Source:HGNC Symbol;Acc:HGNC:28336]
56884	FSTL5	follistatin like 5 [Source:HGNC Symbol;Acc:HGNC:21386]
56919	DHX33	DEAH-box helicase 33 [Source:HGNC Symbol;Acc:HGNC:16718]
56927	GPR108	G protein-coupled receptor 108 [Source:HGNC Symbol;Acc:HGNC:17829]
56935	SMCO4	single-pass membrane protein with coiled-coil domains 4 [Source:HGNC Symbol;Acc:HGNC:24810]
56944	OLFML3	olfactomedin like 3 [Source:HGNC Symbol;Acc:HGNC:24956]
56947	MFF	mitochondrial fission factor [Source:HGNC Symbol;Acc:HGNC:24858]
56952	PRTFDC1	phosphoribosyl transferase domain containing 1 [Source:HGNC Symbol;Acc:HGNC:23333]
56969	RPL23AP32	ribosomal protein L23a pseudogene 32 [Source:HGNC Symbol;Acc:HGNC:25345]
57095	PITHD1	PITH domain containing 1 [Source:HGNC Symbol;Acc:HGNC:25022]
57097	PARP11	poly(ADP-ribose) polymerase family member 11 [Source:HGNC Symbol;Acc:HGNC:1186]
57102	C12orf4	chromosome 12 open reading frame 4 [Source:HGNC Symbol;Acc:HGNC:1184]
57151	LYZL6	lysozyme like 6 [Source:HGNC Symbol;Acc:HGNC:29614]
57157	PHTF2	putative homeodomain transcription factor 2 [Source:HGNC Symbol;Acc:HGNC:13411]
57189	DENND11	DENN domain containing 11 [Source:HGNC Symbol;Acc:HGNC:29472]
57213	SPRYD7	SPRY domain containing 7 [Source:HGNC Symbol;Acc:HGNC:14297]
57493	HEG1	heart development protein with EGF like domains 1 [Source:HGNC Symbol;Acc:HGNC:29227]
57507	ZNF608	zinc finger protein 608 [Source:HGNC Symbol;Acc:HGNC:29238]
57583	TMEM181	transmembrane protein 181 [Source:HGNC Symbol;Acc:HGNC:20958]
57633	LRRN1	leucine rich repeat neuronal 1 [Source:HGNC Symbol;Acc:HGNC:20980]
57639	CCDC146	coiled-coil domain containing 146 [Source:HGNC Symbol;Acc:HGNC:29296]
57655	GRAMD1A	GRAM domain containing 1A [Source:HGNC Symbol;Acc:HGNC:29305]
57659	ZBTB4	zinc finger and BTB domain containing 4 [Source:HGNC Symbol;Acc:HGNC:23847]
57683	ZDBF2	zinc finger DBF-type containing 2 [Source:HGNC Symbol;Acc:HGNC:29313]
57699	CPNE5	copine 5 [Source:HGNC Symbol;Acc:HGNC:2318]
57713	SFMBT2	Scm like with four mbt domains 2 [Source:HGNC Symbol;Acc:HGNC:20256]
57730	ANKRD36B	ankyrin repeat domain 36B [Source:HGNC Symbol;Acc:HGNC:29333]
57801	HES4	hes family bHLH transcription factor 4 [Source:HGNC Symbol;Acc:HGNC:24149]
58160	NFE4	nuclear factor, erythroid 4 [Source:HGNC Symbol;Acc:HGNC:29902]
58473	PLEKHB1	pleckstrin homology domain containing B1 [Source:HGNC Symbol;Acc:HGNC:19079]
58475	MS4A7	membrane spanning 4-domains A7 [Source:HGNC Symbol;Acc:HGNC:13378]
58986	PGAP6	post-glycosylphosphatidylinositol attachment to proteins 6 [Source:HGNC Symbol;Acc:HGNC:17205]
5911	RAP2A	RAP2A, member of RAS oncogene family [Source:HGNC Symbol;Acc:HGNC:9861]
5988	RFPL1	ret finger protein like 1 [Source:HGNC Symbol;Acc:HGNC:9977]
5990	RFX2	regulatory factor X2 [Source:HGNC Symbol;Acc:HGNC:9983]
60468	BACH2	BTB domain and CNC homolog 2 [Source:HGNC Symbol;Acc:HGNC:14078]
6050	RNH1	ribonuclease/angiogenin inhibitor 1 [Source:HGNC Symbol;Acc:HGNC:10074]
6094	ROM1	retinal outer segment membrane protein 1 [Source:HGNC Symbol;Acc:HGNC:10254]
619564	SNORD72	small nucleolar RNA, C/D box 72 [Source:HGNC Symbol;Acc:HGNC:32733]
6253	RTN2	reticulon 2 [Source:HGNC Symbol;Acc:HGNC:10468]
631	BFSP1	beaded filament structural protein 1 [Source:HGNC Symbol;Acc:HGNC:1040]
63898	SH2D4A	SH2 domain containing 4A [Source:HGNC Symbol;Acc:HGNC:26102]
63901	FAM111A	family with sequence similarity 111 member A [Source:HGNC Symbol;Acc:HGNC:24725]
63947	DMRTC1	DMRT like family C1 [Source:HGNC Symbol;Acc:HGNC:13910]
64115	VSIR	V-set immunoregulatory receptor [Source:HGNC Symbol;Acc:HGNC:30085]
641649	TMEM91	transmembrane protein 91 [Source:HGNC Symbol;Acc:HGNC:32393]
64168	NECAB1	N-terminal EF-hand calcium binding protein 1 [Source:HGNC Symbol;Acc:HGNC:20983]
64207	IRF2BPL	interferon regulatory factor 2 binding protein like [Source:HGNC Symbol;Acc:HGNC:14282]
64231	MS4A6A	membrane spanning 4-domains A6A [Source:HGNC Symbol;Acc:HGNC:13375]
642569	TRIM53AP	tripartite motif containing 53A, pseudogene [Source:HGNC Symbol;Acc:HGNC:19025]
642623	UBTFL1	upstream binding transcription factor like 1 [Source:HGNC Symbol;Acc:HGNC:14533]
643008	SMIM5	small integral membrane protein 5 [Source:HGNC Symbol;Acc:HGNC:40030]
64318	NOC3L	NOC3 like DNA replication regulator [Source:HGNC Symbol;Acc:HGNC:24034]
64328	XPO4	exportin 4 [Source:HGNC Symbol;Acc:HGNC:17796]
643733	CASP4LP	caspase 4 like, pseudogene [Source:HGNC Symbol;Acc:HGNC:53657]
64417	TMEM267	transmembrane protein 267 [Source:HGNC Symbol;Acc:HGNC:26139]
64478	CSMD1	CUB and Sushi multiple domains 1 [Source:HGNC Symbol;Acc:HGNC:14026]
645104	CLRN2	clarin 2 [Source:HGNC Symbol;Acc:HGNC:33939]
646201	BASP1P1	BASP1 pseudogene 1 [Source:HGNC Symbol;Acc:HGNC:39418]
646424	SPINK8	serine peptidase inhibitor Kazal type 8 (putative) [Source:HGNC Symbol;Acc:HGNC:33160]
646862	RNF225	ring finger protein 225 [Source:HGNC Symbol;Acc:HGNC:51249]
647087	STMP1	short transmembrane mitochondrial protein 1 [Source:HGNC Symbol;Acc:HGNC:41909]
647310	TEX22	testis expressed 22 [Source:HGNC Symbol;Acc:HGNC:40026]
64761	PARP12	poly(ADP-ribose) polymerase family member 12 [Source:HGNC Symbol;Acc:HGNC:21919]
64770	CCDC14	coiled-coil domain containing 14 [Source:HGNC Symbol;Acc:HGNC:25766]
64843	ISL2	ISL LIM homeobox 2 [Source:HGNC Symbol;Acc:HGNC:18524]
64863	METTL4	methyltransferase like 4 [Source:HGNC Symbol;Acc:HGNC:24726]
652972	LRRC37A5P	leucine rich repeat containing 37 member A5, pseudogene [Source:HGNC Symbol;Acc:HGNC:23369]
653160	GPR199P	G protein-coupled receptor 199, pseudogene [Source:HGNC Symbol;Acc:HGNC:55170]

653316	FAM153CP	family with sequence similarity 153 member C, pseudogene [Source:HGNC Symbol;Acc:HGNC:33936]
653319	KIAA0895L	KIAA0895 like [Source:HGNC Symbol;Acc:HGNC:34408]
654434	SNHG20	small nucleolar RNA host gene 20 [Source:HGNC Symbol;Acc:HGNC:33099]
65981	CAPRIN2	caprin family member 2 [Source:HGNC Symbol;Acc:HGNC:21259]
65991	FUNDC2	FUN14 domain containing 2 [Source:HGNC Symbol;Acc:HGNC:24925]
6689	SPIB	Spi-B transcription factor [Source:HGNC Symbol;Acc:HGNC:11242]
677770	SCARNA22	small Cajal body-specific RNA 22 [Source:HGNC Symbol;Acc:HGNC:32580]
6894	TARBP1	TAR (HIV-1) RNA binding protein 1 [Source:HGNC Symbol;Acc:HGNC:11568]
6920	TCEA3	transcription elongation factor A3 [Source:HGNC Symbol;Acc:HGNC:11615]
692058	SNORD11	small nucleolar RNA, C/D box 11 [Source:HGNC Symbol;Acc:HGNC:32707]
692089	SNORD19	small nucleolar RNA, C/D box 19 [Source:HGNC Symbol;Acc:HGNC:32717]
692247	LMNTD2-AS1	LMNTD2 antisense RNA 1 [Source:HGNC Symbol;Acc:HGNC:41204]
7141	TNP1	transition protein 1 [Source:HGNC Symbol;Acc:HGNC:11951]
7162	TPBG	trophoblast glycoprotein [Source:HGNC Symbol;Acc:HGNC:12004]
7267	TTC3	tetratricopeptide repeat domain 3 [Source:HGNC Symbol;Acc:HGNC:12393]
728039	SSR4P1	signal sequence receptor subunit 4 pseudogene 1 [Source:HGNC Symbol;Acc:HGNC:23131]
728377	ARHGEF34P	Rho guanine nucleotide exchange factor 34, pseudogene [Source:HGNC Symbol;Acc:HGNC:38086]
728431	LINC01137	long intergenic non-protein coding RNA 1137 [Source:HGNC Symbol;Acc:HGNC:49453]
7288	TULP2	TUB like protein 2 [Source:HGNC Symbol;Acc:HGNC:12424]
729264	TP53TG3D	TP53 target 3D [Source:HGNC Symbol;Acc:HGNC:44657]
729288	ZNF286B	zinc finger protein 286B (pseudogene) [Source:HGNC Symbol;Acc:HGNC:33241]
729359	PLIN4	perilipin 4 [Source:HGNC Symbol;Acc:HGNC:29393]
729522	AACSP1	acetoacetyl-CoA synthetase pseudogene 1 [Source:HGNC Symbol;Acc:HGNC:18226]
729582	DIRC3	disrupted in renal carcinoma 3 [Source:HGNC Symbol;Acc:HGNC:17805]
729732	AC068587.4	novel transcript
729747	ZNF878	zinc finger protein 878 [Source:HGNC Symbol;Acc:HGNC:37246]
729911	ANKRD34C-AS1	ANKRD34C antisense RNA 1 [Source:HGNC Symbol;Acc:HGNC:48618]
731223	LINC01220	long intergenic non-protein coding RNA 1220 [Source:HGNC Symbol;Acc:HGNC:49664]
7379	UPK2	uroplakin 2 [Source:HGNC Symbol;Acc:HGNC:12579]
754	PTTG1IP	PTTG1 interacting protein [Source:HGNC Symbol;Acc:HGNC:13524]
7577	ZNF29P	zinc finger protein 29, pseudogene [Source:HGNC Symbol;Acc:HGNC:13080]
7639	ZNF85	zinc finger protein 85 [Source:HGNC Symbol;Acc:HGNC:13160]
7675	ZNF121	zinc finger protein 121 [Source:HGNC Symbol;Acc:HGNC:12904]
7718	ZNF165	zinc finger protein 165 [Source:HGNC Symbol;Acc:HGNC:12953]
7741	ZSCAN26	zinc finger and SCAN domain containing 26 [Source:HGNC Symbol;Acc:HGNC:12978]
7746	ZSCAN9	zinc finger and SCAN domain containing 9 [Source:HGNC Symbol;Acc:HGNC:12984]
7754	ZNF204P	zinc finger protein 204, pseudogene [Source:HGNC Symbol;Acc:HGNC:12995]
79012	CAMKV	CaM kinase like vesicle associated [Source:HGNC Symbol;Acc:HGNC:28788]
79020	C7orf25	chromosome 7 open reading frame 25 [Source:HGNC Symbol;Acc:HGNC:21703]
79026	AHNAK	AHNAK nucleoprotein [Source:HGNC Symbol;Acc:HGNC:347]
79037	PVRIG	PVR related immunoglobulin domain containing [Source:HGNC Symbol;Acc:HGNC:32190]
79149	ZSCAN5A	zinc finger and SCAN domain containing 5A [Source:HGNC Symbol;Acc:HGNC:23710]
79635	CCDC121	coiled-coil domain containing 121 [Source:HGNC Symbol;Acc:HGNC:25833]
79650	USB1	U6 snRNA biogenesis phosphodiesterase 1 [Source:HGNC Symbol;Acc:HGNC:25792]
79652	TMEM204	transmembrane protein 204 [Source:HGNC Symbol;Acc:HGNC:14158]
79710	MORC4	MORC family CW-type zinc finger 4 [Source:HGNC Symbol;Acc:HGNC:23485]
79722	ANKRD55	ankyrin repeat domain 55 [Source:HGNC Symbol;Acc:HGNC:25681]
79730	NSUN7	NOP2/Sun RNA methyltransferase family member 7 [Source:HGNC Symbol;Acc:HGNC:25857]
79745	CLIP4	CAP-Gly domain containing linker protein family member 4 [Source:HGNC Symbol;Acc:HGNC:26108]
79747	ADGB	androglobin [Source:HGNC Symbol;Acc:HGNC:21212]
79827	CLMP	CXADR like membrane protein [Source:HGNC Symbol;Acc:HGNC:24039]
79839	CCDC102B	coiled-coil domain containing 102B [Source:HGNC Symbol;Acc:HGNC:26295]
79843	FAM124B	family with sequence similarity 124 member B [Source:HGNC Symbol;Acc:HGNC:26224]
79856	SNX22	sorting nexin 22 [Source:HGNC Symbol;Acc:HGNC:16315]
79884	MAP9	microtubule associated protein 9 [Source:HGNC Symbol;Acc:HGNC:26118]
80003	PCNX2	pecanex 2 [Source:HGNC Symbol;Acc:HGNC:8736]
80008	TMEM156	transmembrane protein 156 [Source:HGNC Symbol;Acc:HGNC:26260]
80017	DGLUCY	D-glutamate cyclase [Source:HGNC Symbol;Acc:HGNC:20498]
80018	NAA25	N-alpha-acetyltransferase 25, NatB auxiliary subunit [Source:HGNC Symbol;Acc:HGNC:25783]
80019	UBTD1	ubiquitin domain containing 1 [Source:HGNC Symbol;Acc:HGNC:25683]
80111	C3orf36	chromosome 3 putative open reading frame 36 [Source:HGNC Symbol;Acc:HGNC:26170]
80127	BBOF1	basal body orientation factor 1 [Source:HGNC Symbol;Acc:HGNC:19855]
80212	CCDC92	coiled-coil domain containing 92 [Source:HGNC Symbol;Acc:HGNC:29563]
80714	PBX4	PBX homeobox 4 [Source:HGNC Symbol;Acc:HGNC:13403]
80763	SPX	spexin hormone [Source:HGNC Symbol;Acc:HGNC:28139]
80830	APOL6	apolipoprotein L6 [Source:HGNC Symbol;Acc:HGNC:14870]
80833	APOL3	apolipoprotein L3 [Source:HGNC Symbol;Acc:HGNC:14868]
81552	VOPP1	VOPP1 WW domain binding protein [Source:HGNC Symbol;Acc:HGNC:34518]
81553	CYRIA	CYFIP related Rac1 interactor A [Source:HGNC Symbol;Acc:HGNC:25373]
81563	C1orf21	chromosome 1 open reading frame 21 [Source:HGNC Symbol;Acc:HGNC:15494]
81606	LBH	LBH regulator of WNT signaling pathway [Source:HGNC Symbol;Acc:HGNC:29532]
81626	SHCBP1L	SHC binding and spindle associated 1 like [Source:HGNC Symbol;Acc:HGNC:16788]
81831	NETO2	neuropilin and tolloid like 2 [Source:HGNC Symbol;Acc:HGNC:14644]
8287	USP9Y	ubiquitin specific peptidase 9 Y-linked [Source:HGNC Symbol;Acc:HGNC:12633]
83448	PUS7L	pseudouridine synthase 7 like [Source:HGNC Symbol;Acc:HGNC:25276]
83450	DRC3	dynein regulatory complex subunit 3 [Source:HGNC Symbol;Acc:HGNC:25384]
83699	SH3BGRL2	SH3 domain binding glutamate rich protein like 2 [Source:HGNC Symbol;Acc:HGNC:15567]
83853	ROPN1L	rhophilin associated tail protein 1 like [Source:HGNC Symbol;Acc:HGNC:24060]
83862	TMEM120A	transmembrane protein 120A [Source:HGNC Symbol;Acc:HGNC:21697]
83886	PRSS27	serine protease 27 [Source:HGNC Symbol;Acc:HGNC:15475]
83891	SNX25	sorting nexin 25 [Source:HGNC Symbol;Acc:HGNC:21883]
83903	HASPIN	histone H3 associated protein kinase [Source:HGNC Symbol;Acc:HGNC:19682]
84067	FAM160A2	family with sequence similarity 160 member A2 [Source:HGNC Symbol;Acc:HGNC:25378]
84181	CHD6	chromodomain helicase DNA binding protein 6 [Source:HGNC Symbol;Acc:HGNC:19057]
84223	IQCG	IQ motif containing G [Source:HGNC Symbol;Acc:HGNC:25251]
84247	RTL6	retrotransposon Gag like 6 [Source:HGNC Symbol;Acc:HGNC:13343]
84281	C2orf88	chromosome 2 open reading frame 88 [Source:HGNC Symbol;Acc:HGNC:28191]
84304	NUDT22	nudix hydrolase 22 [Source:HGNC Symbol;Acc:HGNC:28189]
84417	ECRG4	ECRG4 augurin precursor [Source:HGNC Symbol;Acc:HGNC:24642]
84504	NKX6-2	NK6 homeobox 2 [Source:HGNC Symbol;Acc:HGNC:19321]
84519	ACRBP	acrosin binding protein [Source:HGNC Symbol;Acc:HGNC:17195]
84529	CDIN1	CDAN1 interacting nuclease 1 [Source:HGNC Symbol;Acc:HGNC:26929]
84569	LYZL1	lysozyme like 1 [Source:HGNC Symbol;Acc:HGNC:30502]
84672	TTTY6	testis-specific transcript, Y-linked 6 [Source:HGNC Symbol;Acc:HGNC:16483]
84690	SPATA22	spermatogenesis associated 22 [Source:HGNC Symbol;Acc:HGNC:30705]
84691	FAM71F1	family with sequence similarity 71 member F1 [Source:HGNC Symbol;Acc:HGNC:30704]
84698	CAPS2	calcyphosine 2 [Source:HGNC Symbol;Acc:HGNC:16471]
84707	BEX2	brain expressed X-linked 2 [Source:HGNC Symbol;Acc:HGNC:30933]
84793	FOXD2-AS1	FOXD2 adjacent opposite strand RNA 1 [Source:HGNC Symbol;Acc:HGNC:44256]
84809	CROCCP2	CROCC pseudogene 2 [Source:HGNC Symbol;Acc:HGNC:28170]
84886	C1orf198	chromosome 1 open reading frame 198 [Source:HGNC Symbol;Acc:HGNC:25900]
84935	MEDAG	mesenteric estrogen dependent adipogenesis [Source:HGNC Symbol;Acc:HGNC:25926]
84941	HSH2D	hematopoietic SH2 domain containing [Source:HGNC Symbol;Acc:HGNC:24920]
85446	ZFHX2	zinc finger homeobox 2 [Source:HGNC Symbol;Acc:HGNC:20152]
8723	SNX4	sorting nexin 4 [Source:HGNC Symbol;Acc:HGNC:11175]
8725	URI1	URI1 prefoldin like chaperone [Source:HGNC Symbol;Acc:HGNC:13236]
8872	CDC123	cell division cycle 123 [Source:HGNC Symbol;Acc:HGNC:16827]
8880	FUBP1	far upstream element binding protein 1 [Source:HGNC Symbol;Acc:HGNC:4004]
90024	FLJ20021	uncharacterized LOC90024 [Source:NCBI gene (formerly Entrezgene);Acc:90024]
90070	LACRT	lacritin [Source:HGNC Symbol;Acc:HGNC:16430]
90139	TSPAN18	tetraspanin 18 [Source:HGNC Symbol;Acc:HGNC:20660]
9022	CLIC3	chloride intracellular channel 3 [Source:HGNC Symbol;Acc:HGNC:2064]
90273	CEACAM21	CEA cell adhesion molecule 21 [Source:HGNC Symbol;Acc:HGNC:28834]
90313	TP53I13	tumor protein p53 inducible protein 13 [Source:HGNC Symbol;Acc:HGNC:25102]
90355	MACIR	macrophage immunometabolism regulator [Source:HGNC Symbol;Acc:HGNC:25052]
90362	FAM110B	family with sequence similarity 110 member B [Source:HGNC Symbol;Acc:HGNC:28587]
90488	TMEM263	transmembrane protein 263 [Source:HGNC Symbol;Acc:HGNC:28281]
90853	SPOCD1	SPOC domain containing 1 [Source:HGNC Symbol;Acc:HGNC:26338]
90987	ZNF251	zinc finger protein 251 [Source:HGNC Symbol;Acc:HGNC:13045]
91107	TRIM47	tripartite motif containing 47 [Source:HGNC Symbol;Acc:HGNC:19020]
9121	SLC16A5	solute carrier family 16 member 5 [Source:HGNC Symbol;Acc:HGNC:10926]
9202	ZMYM4	zinc finger MYM-type containing 4 [Source:HGNC Symbol;Acc:HGNC:13055]
92106	OXNAD1	oxidoreductase NAD binding domain containing 1 [Source:HGNC Symbol;Acc:HGNC:25128]
92154	MTSS2	MTSS I-BAR domain containing 2 [Source:HGNC Symbol;Acc:HGNC:25094]
92181	UBTD2	ubiquitin domain containing 2 [Source:HGNC Symbol;Acc:HGNC:24463]
92521	SPECC1	sperm antigen with calponin homology and coiled-coil domains 1 [Source:HGNC Symbol;Acc:HGNC:30615]
92689	FAM114A1	family with sequence similarity 114 member A1 [Source:HGNC Symbol;Acc:HGNC:25087]
92691	TMEM169	transmembrane protein 169 [Source:HGNC Symbol;Acc:HGNC:25130]
9317	PTER	phosphotriesterase related [Source:HGNC Symbol;Acc:HGNC:9590]
93349	SP140L	SP140 nuclear body protein like [Source:HGNC Symbol;Acc:HGNC:25105]
93594	TBC1D31	TBC1 domain family member 31 [Source:HGNC Symbol;Acc:HGNC:30888]
9406	ZRANB2	zinc finger RANBP2-type containing 2 [Source:HGNC Symbol;Acc:HGNC:13058]
94163	SNORD38B	small nucleolar RNA, C/D box 38B [Source:HGNC Symbol;Acc:HGNC:30356]
9452	ITM2A	integral membrane protein 2A [Source:HGNC Symbol;Acc:HGNC:6173]
9576	SPAG6	sperm associated antigen 6 [Source:HGNC Symbol;Acc:HGNC:11215]
9581	PREPL	prolyl endopeptidase like [Source:HGNC Symbol;Acc:HGNC:30228]
9699	RIMS2	regulating synaptic membrane exocytosis 2 [Source:HGNC Symbol;Acc:HGNC:17283]
9711	RUBCN	rubicon autophagy regulator [Source:HGNC Symbol;Acc:HGNC:28991]
9717	SEC14L5	SEC14 like lipid binding 5 [Source:HGNC Symbol;Acc:HGNC:29032]
9729	KIAA0408	KIAA0408 [Source:HGNC Symbol;Acc:HGNC:21636]
9737	GPRASP1	G protein-coupled receptor associated sorting protein 1 [Source:HGNC Symbol;Acc:HGNC:24834]
9805	SCRN1	secernin 1 [Source:HGNC Symbol;Acc:HGNC:22192]
9858	PPP1R26	protein phosphatase 1 regulatory subunit 26 [Source:HGNC Symbol;Acc:HGNC:29089]
987	LRBA	LPS responsive beige-like anchor protein [Source:HGNC Symbol;Acc:HGNC:1742]
9880	ZBTB39	zinc finger and BTB domain containing 39 [Source:HGNC Symbol;Acc:HGNC:29014]
9881	TRANK1	tetratricopeptide repeat and ankyrin repeat containing 1 [Source:HGNC Symbol;Acc:HGNC:29011]
9889	ZBED4	zinc finger BED-type containing 4 [Source:HGNC Symbol;Acc:HGNC:20721]
9910	RABGAP1L	RAB GTPase activating protein 1 like [Source:HGNC Symbol;Acc:HGNC:24663]
